# Resolving phylogenetic relationships of Delphacini and Tropidocephalini (Hemiptera: Delphacidae: Delphacinae) as inferred from four genetic loci

**DOI:** 10.1038/s41598-017-03624-w

**Published:** 2017-06-12

**Authors:** Yi-Xin Huang, Li-Fang Zheng, Charles R. Bartlett, Dao-Zheng Qin

**Affiliations:** 1 Key Laboratory of Plant Protection Resources and Pest Management of the Ministry of Education; Entomological Museum, Northwest A&F University, Yangling, Shaanxi 712100 China; 2 Department of Entomology and Wildlife Ecology, University of Delaware, Newark, DE 19716 USA

## Abstract

This paper explores the phylogeny of the delphacid subfamily Delphacinae based on nuclear ribosomal and mitochondrial DNA sequences of four genetic loci (16S rDNA, 28S rDNA, *Cytochrome oxidase I* and *Cytochrome b*). Maximum likelihood and Bayesian analyses yield robust phylogenetic trees. The topologies support the monophyly of Delphacinae and its basal split into three tribes, and provisionally support subdividing Delphacini into three clades, including a more broadly defined Numatina. The tribe Tropidocephalini is divided into two clades. In addition, *Paranectopia* is transferred from Tropidocephalini to Delphacini and *Harmalia* syn. nov. is regarded as a junior synonym of *Opiconsiva*. The genera *Bambusiphaga*, *Megadelphax* and *Muirodelphax* are found to be paraphyletic. The estimated time to the most recent common ancestor of Delphacinae is roughly at 90 million years ago in Late Cretaceous.

## Introduction

The planthopper subfamily Delphacinae Leach is the most speciose and economically important group in Delphacidae Leach^1, 2^. It comprises approximately 80% of all delphacid species. Members in this subfamily are terraneous and phloem-feeding, with 55 species known as pests on 25 crops, such as rice, maize, wheat, barley, sugarcane and bamboo^3^. Of these, 30 species are known as plant pathogen vectors, mostly viruses, except phytoplasmas in the Saccharosydnini Vilbaste^3–8^. Delphacids may damage plants directly by feeding and oviposition, or less directly by the introduction of saliva^9^, potentially leading to “hopperburn”, or by the introduction of plant pathogens^10, 11^. The delphacid planthoppers on rice, the “ricehoppers”, are among the most devastating pests on cultivated rice in southern China and northern Indochina. For example, the continuous outbreak of the white-backed planthopper, *Sogatella furcifera* (Horváth), has caused immeasurable rice yield loss in this region over the last decade^12, 13^, and extensive outbreaks of the brown planthopper, *Nilaparvata lugens* (Stål) and the small brown planthopper, *Laodelphax striatellus* (Fallén), in recent decades have caused significant losses in rice yields in China^14, 15^.

The early classifications of Delphacinae were diagnostic groupings based on morphology. Early works divided Delphacinae into three tribes (Tropidocephalini Muir, Alohini Muir and Delphacini Leach)^16–18^, or treated it as a subfamily without tribal subdivision^19^. Wagner recognized no tribes in Delphacinae^20^. Asche provided the first cladistic investigation of the Delphacidae^1, 21^, and revised it into seven subfamilies, with the largest subfamily, Delphacinae, including over 80% of described species. Asche’s Delphacinae was comprised of tribes Saccharosydnini, Tropidocephalini, and Delphacini. Asche’s Delphacini was expansively defined to include the Achorotilinae Wagner, Alohini Muir, Chlorioinae Wagner, Megamelinae Haupt and Stirominae Wagner of previous authors, putatively supported by an articulated suspensorium^1, 22^.

Thereafter, three other studies treated the higher classification of Delphacidae using morphological features^23–25^. Yang and colleagues explored the phylogeny of Asiracinae Motschulsky and Tropidocephalini using phenetic and cladistic methods, including *Ugyops* Guerin-Meneville (in Asiracinae) and nine Asian genera of Tropidocephalini^23^. These analyses showed that *Ugyops* was the most basal group, sister to Tropidocephalini, with the genus *Tropidocephala* Stål placed sister to the remainder of the tribe. Emeljanov utilized features of immatures to define a more broadly inclusive Delphacinae^24^. Emeljanov portrayed the Delphacinae as being a clade paraphyletic within the Asiracinae, and comprised seven tribes (Vizcayini Asche, Kelisiini Wagner, Stenocranini Wagner, Plesiodelphacini Asche, Delphacini, Tropidocephalini and Saccharosydnini), four of which were treated as subfamilies by Asche. Hamilton generally followed Emeljanov except he treated Kelisiini as a subtribe of Stenocranini, and Saccharosydnini as a subtribe of Tropidocephalini^25^.

Molecular investigations began with Dijkstra *et al*.^26, 27^, who examined delphacid phylogeny using mitochondrial DNA nucleotide sequence data from COI (*Cytochrome oxidase I*) and 12S rDNA, respectively. Although a limited number of delphacine taxa were included, Dijkstra *et al*. provided insight into resolving the phylogenetic relationships of Delphacinae. More recently, the more comprehensive phylogeny of Urban *et al*. presented the evolution of Delphacidae, based on DNA nucleotide sequence data from four genetic loci (18S rDNA, 28S rDNA, *wingless* and *Cytochrome oxidase I*) and 132 coded morphological characters^28^. Urban *et al*.’s Delphacinae comprised 93 species (89 species in Delphacini, two species in each of Saccharosydnini and Tropidocephalini) and the topology generally supported the higher classifications of Delphacidae proposed by Asche, Emeljanov and Hamilton, and suggested a rapid diversification of the Delphacini associated with host shifts to, and within, Poaceae, and specifically from C3 to C4 grasses.

Despite these studies, the phylogeny of the Tropidocephalini remains essentially unexplored. The tribe currently contains more than 180 species in 37 genera widely distributed throughout the Palaearctic, Afrotropical, Indomalayan, Australian, Neotropical and Pacific Regions, but most diverse in the Indomalayan Region^29–31^. The Tropidocephalini primarily feed on bamboos, with a few species feeding on other grasses^30, 31^. The Chinese Tropidocephalini, comprising about 90 species in 23 genera, represents the richest species diversity of this tribe worldwide, with most taxa restricted to south China. Hou & Chen explored the relationships among three *Belocera* Muir species, and subsequently among four genera of Tropidocephalini, as inferred from partial 16S rDNA gene sequences. These studies resulted in partly resolved relationships within the Tropidocephalini^32, 33^.

Within the Delphacini, Emeljanov placed 22 genera into his new subtribe Numatina Emeljanov based on the presence of an articulated suspensorium of the phallobase, implying that all other genera of Delphacini should be assigned in the nominal subtribe Delphacina Leach^34^. While not directly tested, this subtribal classification was considered doubtful by Urban *et al*. because Numatina (represented by a single taxon) was nested deeply within the Delphacina. In results presented by Urban *et al*., the Delphacini was “divided consistently into three major clades (plus some ‘intermediate’ taxa)”. However, the relationships were only partially resolved, just as it stated “the resolution of relationships within Delphacini will require the addition of new data sources, such as sequence data from other genes with a higher rate of mutation”.

At present, the fossil record for Delphacidae is very incomplete. Fossils of delphacid planthoppers explicitly reported so far show that the known fossils of Delphacidae are from the Paleogene and Neogene of North America and Europe, including four species (*Amagua fortis* Cockerell, *Chloriona stavropolitana* (Becker-Migdisova), *Delphax rhenana* Statz and *Delphax senilis* Scudder) in Delphacini of the Delphacinae and one species in Ugyopini (*Serafinana perperunae* Gebicki & Szwedo) of the Asiracinae^2, 35^. The estimation of divergence dates among living delphacid taxa could provide insights into the evolution of the Delphacinae.

The purpose of this study is to examine the phylogeny of the speciose subfamily Delphacinae with a wide range of taxonomic subsampling based on nucleotide sequences. To reconstruct the generic relationships, three mitochondrial genes were selected with a high rate of mutation, and one nuclear ribosomal gene to avoid the side effect of maternal inheritance. All genera in Yang *et al*. and Hou & Chen’s studies were included to evaluate the support for the classification of Tropidocephalini^23, 32, 33^. Redivision of the tribe Delphacini was tested by including 23 species from Urban *et al*. and expanded species sampling. In addition, the positions of several controversial genera (i.e. *Paranectopia* Ding et Tian, *Miranus* Chen et Ding, *Harmalia* Fennah) were investigated. The divergence time of Delphacinae was estimated based on the concatenated dataset.

## Results

### Sequence characterization

The multiple sequence alignments of protein-coding genes *cox1* (*Cytochrome oxidase I*) and *cytb* (*Cytochrome b*) were unambiguous, in which alignment of *cox1* gene contained gaps, but none of that interrupted or shifted the reading frame. Unlike the sequences of protein-coding genes, the sequences of ribosomal genes varied in length across the sampled taxa. Highly variable regions of 16S rDNA and 28S rDNA that differed in length were excluded from phylogenetic analyses due to extreme ambiguity of alignment.

For 28S rDNA, the result of sequence variation analyses showed the sequences were 718 bp in length, including 185 variable sites, 533 conservative sites and 138 parsimony-informative sites, the percentage of A+T was 47.1%. The 16S rDNA comprised a sequence of 492 bp with gaps. Failed DNA extractions or PCR amplifications contributed to the missing data. The protein-coding genes alignment consisted of 1125 sites, including 537 bp of *cox1*. The average content of A+T is much higher than the content of C+G in mitochondrial genes, which is in accordance with Simon *et al*.^36^. The detailed descriptive statistics for sequence segments are listed in Table 1. After aligning with outgroups, the length of 28S rDNA and 16S rDNA became longer by including gaps. The sequence alignments of each partition are listed in Supplementary Tables S1–S4.

**Table 1:** Descriptive statistics for data partitions.

Data Partition	28S rDNA	16S rDNA	*cox1*	*cytb*
Length (bp)	718	492	537	588
Conserved	533	208	269	255
Varied	185	284	268	333
Parsim-info	138	233	233	303
T%	22.5	40.6	36.2	40.6
C%	23.5	8.2	16.2	16.9
A%	24.6	36.5	33.1	32.6
G%	29.4	14.7	14.5	9.9

The results of chi-square tests for base compositional homogeneity of each gene showed homogeneity among taxa. Little saturation was detected in all four alignments both in assuming a symmetrical and an asymmetrical topology. All sequence segments are useful for tree reconstruction.

### Phylogenetic analyses

**Figure 1:**
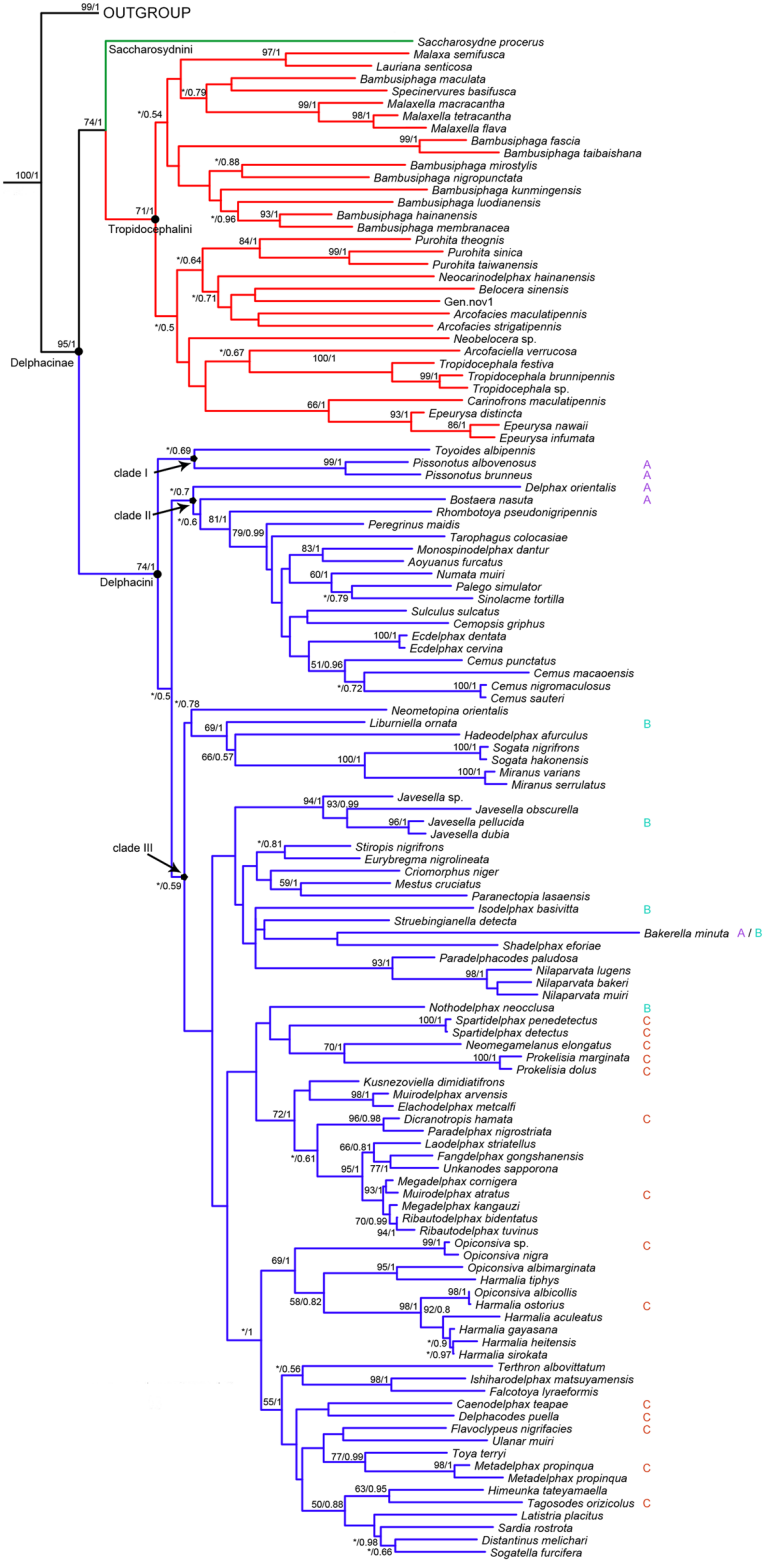
Phylogenetic tree of Delphacinae obtained from maximum likelihood (ML) analysis based on concatenated data of genes *cox1*, *cytb*, 16S rDNA and 28S rDNA. ML bootstrap values and Bayesian posterior probabilities are indicated at internal nodes. Bootstrap values under 50 are replaced by “*”. The species acquired from Urban *et al*. were indicated with A, B, C as belonging to clade 1, 2 and 3 in their research, respectively.

The combined data consist of nuclear ribosomal gene 28S rDNA and mitochondrial genes of 16S rDNA, *cox1*, *cytb*, by which we obtained a robust phylogenetic tree of Delphacinae (Fig. 1). Delphacinae was recovered as monophyletic (Bootstrap support values = 95, Posterior probability values = 1) and divided into three clades represented by Saccharosydnini, Tropidocephalini and Delphacini, respectively. Saccharosydnini and Tropidocephalini were recovered as sister groups.

Within Tropidocephalini, all genera except *Bambusiphaga* Huang & Ding were recovered as monophyletic. In the ML topology, *Belocera* and a new genus were recovered as sister groups, with the combined clade sister to *Arcofacies* Muir. However, the new genus was more closely related to *Arcofacies* in BI tree. Two sister groups, *Tropidocephala* and *Arcofaciella* Fennah; *Epeurysa* Matsumura and *Carinofrons* Chen & Li were recovered.

In the tribe Delphacini, three clades were recovered. Several genera within Delphacini were recovered as paraphyletic, including *Muirodelphax* Wagner, *Megadelphax* Wagner, *Harmalia* Fennah and *Opiconsiva* Distant. Sister groups that were recovered within Delphacini include *Cemus* Fennah and *Ecdelphax* Yang; *Sogata* Distant and *Miranus* Chen & Ding; *Paradelphacodes* Wagner and *Nilaparvata* Distant; *Ishiharodelphax* Kwon and *Falcotoya* Fennah; *Metadelphax* Wagner and *Toya* Distant; *Sogatella* Fennah and *Distantinus* (Distant) (as *Matutinus*)^37^. The genus *Paranectopia* (formerly assigned to Tropidocephalini) was grouped with *Mestus cruciatus* Ren & Qin in the Delphacini. The three Palearctic genera, *Megadelphax*, *Ribautodelphax* Wagner and *Muirodelphax* formed a clade, with *Ribautodelphax* derived within *Megadelphax*.

### Monophyletic tests

The log likelihood score of best ML tree was 66657.05. Ten taxa recognized as monophyletic groups were compared against the best ML tree by SH and AU tests. The results support the monophyly of Delphacidae, Delphacinae and Delphacini. Also, the paraphyly of *Opiconsiva* was corroborated. The monophyly of Tropidocephalini was statistically rejected by AU test (P < 0.05). However, after the transference of *Paranectopia* to Delphacini, the monophyly of Tropidocephalini and Delphacini were both supported. Both AU and SH tests failed to reject the monophyly of *Bambusiphaga*, *Megadelphax* and *Harmalia*, even though they were not recovered as monophyletic in the best ML topology.

### Divergence time estimation

**Figure 2:**
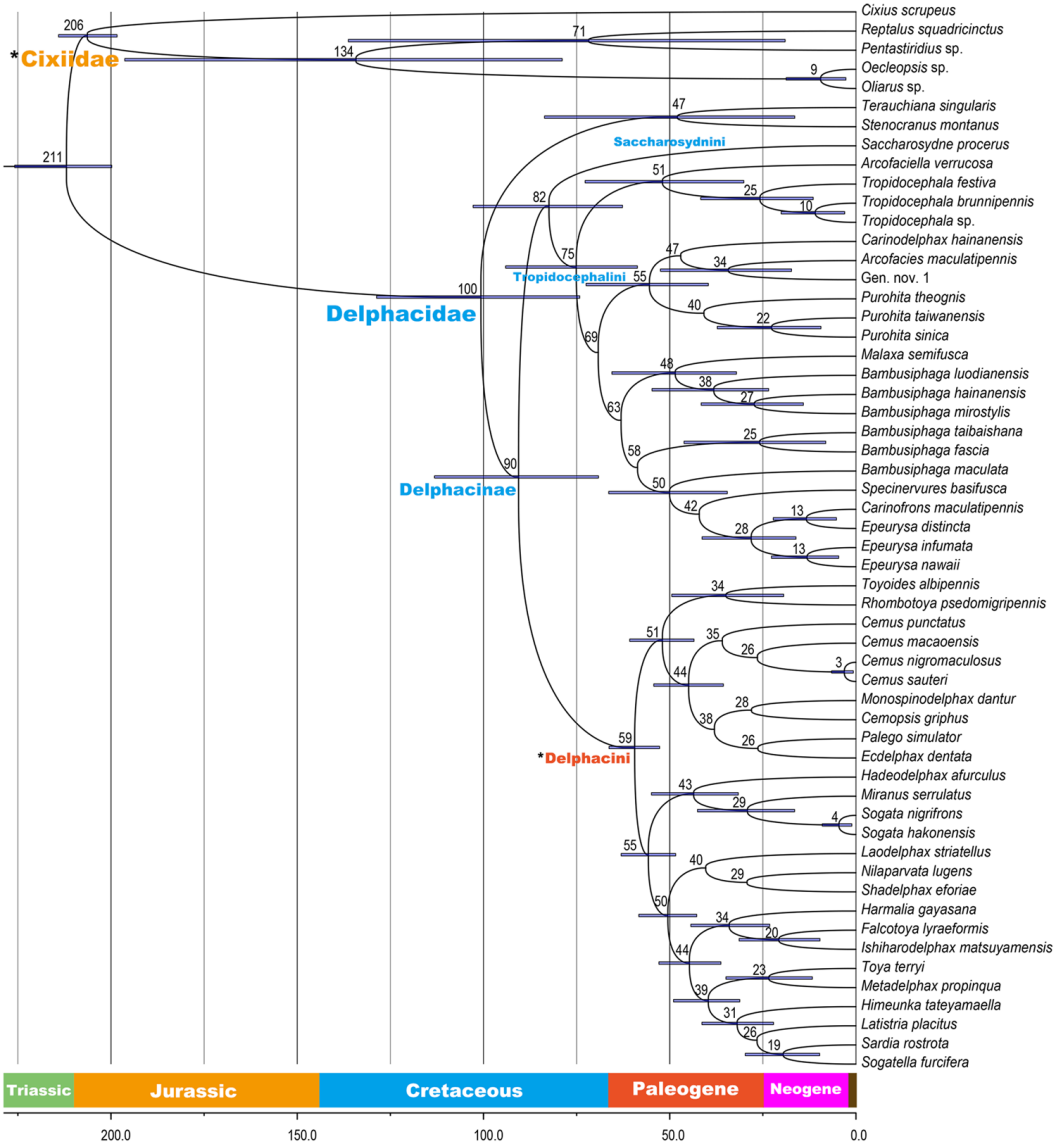
Chronogram of Delphacinae divergence time estimated from the BEAST analysis with a Bayesian relaxed lognormal clock. The numbers at nodes indicate the mean ages and blue bars represent 95% highest posterior density intervals for the node ages. Two calibration points are indicated as *Cixiidae and *Delphacini.

The divergence time chronogram of Delphacinae is presented in Fig. 2, with branch length as mean age. The divergence of Delphacidae from Cixiidae Spinola is approximately 211 Mya (million years ago). The estimated time to the most recent common ancestor of Delphacinae is roughly at 90 Mya. Saccharosydnini and Tropidocephalini split at some 82 Mya, slightly earlier than the origin of Tropidocephalini (75 Mya). The genus *Tropidocephala* originated at approximately 25 Mya.

## Discussion

The present study reconstructed a relatively comprehensive phylogenetic tree of Delphacinae based on mitochondrial and nuclear gene sequences, which divide this subfamily into three distinct clades, representing the three tribes (Delphacini, Saccharosydnini and Tropidocephalini) of Delphacinae. In Tropidocephalini, the genus *Lauriana* Ren & Qin grouped with *Malaxa* Melichar, and thence to a clade contained *Malaxella* Ding & Hu, supporting the previous morphological studies of Ren *et al*.^29^. *Belocera* and *Arcofacies* are closely related and in accordance with the study of Yang^23^. The results also support the status of the *Bambusiphaga fascia* group, but failed recognize the monophyly of four other species groups included in the present study (*B. nigropunctata* group, *B. citricolorata* group, *B. maculata* group and *B. mirostylis* group)^38, 39^. In addition, *B. maculata* Chen & Li separated from the other members of the genus, sister to *Specinervures basifusca* Chen & Li. However, *Bambusiphaga* and *Specinervures* Kuoh & Ding are distinctly different morphologically (e.g., in *Specinervures*, the forewing with crossveins just proximad of midlength and trailing margin produced in distal half; ventrolateral margins of male pygofer strongly incised and gonostyli with inner angles reflected mesoventral apically^40^), it is unreasonable to transfer *B. maculata* to *Specinervures* based on current classification criteria. Thus, while the paraphyly of *Bambusiphaga* is here indicated, and further review of included taxa is needed to obtain a satisfactory classification. Furthermore, this study consistently divided the tribe Tropidocephalini into two major lineages, the first clade includes *Malaxa*, *Lauriana*, *Specinervures*, *Bambusiphaga* and *Malaxella*, another one contains the remaining genera in the tribe except *Paranectopia*. However, morphological diagnoses of these two clades require further study.

Although the monotypic genus *Paranectopia* was originally established in Tropidocephalini based on the post-tibial spur not having fine teeth along the posterior margin^41^, features of the male genitalia of *Paranectopia lasaensis* Ding et Tian show the aedeagus and anal segment are not in close functional contact, the suspensorium is present, and the aedeagal base is not twisted^42^, which meet the definition of the tribe Delphacini *sensu* Asche^1^. The assignment of *Paranectopia* in Tropidocephalini caused the monophyly rejected of Tropidocephalini in the AU test, and is accepted after deletion of *Paranectopia*. Here *Paranectopia* is transferred into Delphacini.

The subtribal classification of Delphacini can be evaluated using the ninety-one genera in Delphacini sampled here, including 23 species representing the “three clades” hypothesis in Urban *et al*.’s phylogeny^28^. Our results support the redivision of this tribe into three clades, but they compositionally differ from Urban *et al*. in that clade I comprises two genera, clade II includes 14 genera, and clade III holds 46 genera (Fig. 1). Furthermore, clade II has four (*Bostaera* Ball, *Numata* Busck, *Cemus*, *Palego* Fennah) of the 22 genera in subtribe Numatina as defined by Emeljanov^34^. In contrast, clades I and III do not include any Numatina. Therefore our results support the general concept of subtribe Numatina, although more broadly defined. However, the presence of *Delphax* Fabricius (the name-bearing genus of subtribe Delphacina) at the base of clade II (including the four genera in Numatina) presents difficulties. Hence, the resolution of relationships within Delphacini still requires more gene and taxon sampling.

This study included 23 species in Urban *et al*.’s phylogeny^28^, indicated by the letters A, B and C (in Fig. 1) to designate the three clades of that study. A includes *Bakerella minuta* Beamer, *Pissonotus albovenosus* Osborn, *Pissonotus brunneus* Van Duzee, *Bostaera nasuta* Ball and *Delphax orientalis* (Linnavuori) of clade 1 in Urban *et al*.’s study. B includes *Liburniella ornata* (Stål), *Nothodelphax neocclusa* (Muir & Giffard), *Isodelphax basivitta* (Van Duzee) and *Javesella pellucida* (Fabricius) of Clade 2 of Urban *et al*.’s study. C includes *Caenodelphax teapae* (Fowler), *Muirodelphax arvensis* (Fitch), *Spartidelphax detecta* (Van Duzee), *Flavoclypeus nigrifacies* (Muir), *Spartidelphax penedetecta* (Beamer), *Delphacodes puella* (Van Duzee), *Dicranotropis hamata* (Boheman), *Harmalia ostorius* Kirkaldy, *Metadelphax propinqua* (Fieber), *Neomegamelanus elongatus* (Ball), *Opiconsiva* sp., *Prokelisia dolus* Wilson, *Prokelisia marginata* (Van Duzee), *Tagosodes orizicolus* (Muir) of Clade 3 in Urban *et al*.’s study. This study placed *Bakerella minuta* in Urban *et al*.’s clade 2, consistent with Urban *et al*.’s MP analysis. *Nothodelphax neocclusa* was placed in clade 2 in Urban *et al*.’s analysis, but in the present study is allied with the *Spartina*-feeding clade within clade 3, rendering the clades 2 and 3 of Urban *et al*. paraphyletic. Therefore, sister group clades 2 and 3 of Urban *et al*. should be merged together as a monophyletic lineage.

Chen *et al*. established the genus *Miranus* Chen & Ding, for *Stenocranus varians* (Kuoh), formerly in Stenocraninae^43^, but was thereafter included in Delphacini^44^. This study revealed that *M. varians* (Kuoh) and *M. serrulatus* Dong & Qin formed a monophyletic group sister with *Sogata* Distant, supporting the assignment of *Miranus* species in Delphacini rather than in Stenocraninae. The genera *Opiconsiva* and *Harmalia* share many morphological synapomorphies and formed a clade (although in *Opiconsiva* the aedeagal base bears a dorsal longitudinally grooved structure), but *Harmalia* is deeply nested within *Opiconsiva* making it paraphyletic. Both the AU and SH test of monophyly for *Opiconsiva* are rejected. *Harmalia* is placed here as a junior synonym of *Opiconsiva*.

The monophyly of three genera *Ribautodelphax*, *Muirodelphax* and *Megadelphax* all established by Wagner in 1963 were tested. Each of these included two representative species. The topology among these taxa (Fig. 1) is (*Megadelphax cornigera* + *Muirodelphax atratus*) + (*Megadelphax kangauzi* + (*Ribautodelphax bidentatus* + *Ribautodelphax tuvinus*)), *Muirodelphax arvensis* is placed outside this clade sister to *Elachodelphax metcalfi* (Kusnezov), casting doubt on the monophyly of *Muirodelphax*, at least as comprised by Hamilton and Kwon^45^. The species *R. bidentatus* was once transferred to *Megadelphax* by Vilbaste (because the processes on the anal tube are not cruciate, a diagnostic feature of *Ribautodelphax*) but its placement in *Ribautodelphax* is supported in this study. Our analyses suggest that *Ribautodelphax* may be derived within *Megadelphax*, which is plausible given the many similarities among these genera. Nevertheless, we feel that the six species included in this study are not sufficient to solve the phylogenetic interrelationships among these genera, Hence, additional evidence and more taxa are needed to clarify their phylogeny.

The relaxed molecular clock analysis shows that the Delphacinae can be traced into the Late Cretaceous (90 Mya), and that the tribe Tropidocephalini also originated in the Late Cretaceous (75 Mya), with Saccharosydnini diverging from Tropidocephalini 82 million years ago. Moreover, these results indicate that the family Delphacidae can be traced into the Cretaceous (100 Mya), much before the earliest fossil record of this family (in the Paleogene). These observations are consistent with the idea that molecular estimates should be earlier than fossil ages. Nevertheless, Delphacidae originated in this study later than that estimated by Song and Liang (Early Cretaceous, around 129 Mya)^46^. The possible reasons for the disagreement of estimated divergence times include the delphacid fossil used as a calibration point to provide the minimum age for Delphacidae, and the greater taxon sampling of Delphacidae in this study. Furthermore, Delphacinae feed mostly on Poaceae, with a few species in Delphacini known as sedge feeders^47^, and a recent study has shown that the estimated age of the Poaceae ranged from 107 Mya to 129 Mya (in Cretaceous)^48^, earlier than the time estimation of Delphacinae in this study (90 Mya), which implies that the Delphacinae have undergone a rapid diversification after Poaceae evolved, indirectly supporting Wilson *et al*.’s hypothesis of delphacid adaptive radiation following the development of the grassland biome^47^. However, our divergence date estimates should be regarded as provisional, as more evidences, including fossil, morphological and molecular are still needed to support these results, particularly new fossil cross-calibrations.

A robust molecular phylogeny of Delphacinae was reconstructed in this study. The monophyly of Delphacinae was supported along with its basal split into three monophyletic tribes. The redivision of Delphacini into three clades, including a more broadly defined Numatina is also supported here. In addition, *Paranectopia* is placed in Delphacini and *Harmalia* **syn. nov**. is regarded as a junior synonym of *Opiconsiva*. Although the phylogenetic analyses elucidated many main points in Delphacinae, expanded data and greater taxon sampling are still needed to better understand the evolution of Delphacinae.

## Materials and Methods

### Taxon sampling

Specimens collected for this study were identified morphologically by the corresponding author and preserved in 100% ethanol at −20 °C in the Key Laboratory of Plant Protection Resources and Pest Management of Ministry of Education, Entomological Museum, Northwest A&F University (NWAFU). Sampling includes 123 ingroups and eight outgroups taxa (Supplementary Table S5). Ingroup sampling represents all three recognized tribes of this subfamily (Saccharosydnini, Tropidocephalini and Delphacini). Fifty-two ingroups nucleotide sequences were acquired from Genbank. Eight other species were selected as outgroups, including five species in Cixiidae, two species in Stenocraninae Wagner and one species in Kelisiinae Wagner.

### Molecular data

DNA were extracted either from thoracic or leg muscle tissues using Qiagen DNEasy Kits (Qiagen, Inc., Valencia, CA, USA) or BioFlux Biospin Insect Genomic DNA Extraction Kit (Bioer, Inc., Hangzhou, China). The 28S rDNA, 16S rDNA, *cox1* and *cytb* were amplified by using the previously reported oligonucleotide primers as Dietrich *et al*.^49^, Clary & Wolstenholme^50^, Simon *et al*.^36^ (Table 2). All polymerase chain reactions (PCR) were performed in 25 μl reaction volumes with the following cycling protocol: an initial denaturation step of 5 min at 94 °C, followed by 20–60 s at 94 °C, 35 cycles of 1 min at 49–52 °C, 1 min at 72 °C, ending with 7 min incubation at 72 °C. The PCR products were inspected in 1% agarose gel electrophoresis with ethidium-bromide staining. Sequencing was carried out with the same primers used for amplification on both strands. New sequences were submitted to GenBank (Supplementary Table S5).

**Table 2:** The primers of 28S rDNA, 16S rDNA, *cox1* and *cytb* genes.

Locus Primer	Direction	Primer sequence	Reference
28S rDNA	Forward	5′-CCT CGG ACC TTG AAA ATC C-3′	Dietrich *et al*.
	Reverse	5′-TGT CTC CTT ACA GTG CCA GA-3′	
16S rDNA	Forward	5′-GCC TGT TTA TCA AAA ACA T-3′	Clary & Wolstenholm
	Reverse	5′-CCG GTC TGA ACT CAG ATC A-3′	
*cox1*	Forward	5′-TTGATTTTTTGGTCAYCCWGAAGT-3′	Simon *et al*.
	Reverse	5′-GGRAARAAWGTTAARTTWACTCC-3′	
*cytb*	Forward	5′-GTTCTACCTTGAGGTCAAATATC-3′	Simon *et al*.
	Reverse	5′-TTCTACTGGTCGTGCTCCAATTCA-3′	

### Alignment

New sequences were proofread and aligned into contigs in BioEdit^51^. To detect contamination, each sequence was searched under BLAST on GenBank. Alignment was performed using MUSCLE as implemented in MEGA 6^52^ with default options and checked manually.

The descriptive statistics for sequence segments were conducted in MEGA 6. Chi-Square test of homogeneity of each gene was performed in PAUP 4.0b10^53^. Substitution saturation of each sequence segment was tested using DAMBE 5^54^ by comparing the index of substitution saturation (Iss) with critical values (Iss.c).

### Phylogenetic analyses

One combined analysis of four segments was used for reconstructing phylogeny by using maximum likelihood (ML) and Bayesian inference (BI). Incomplete genes were included to increase the accuracy of phylogenetic analyses, especially for poorly supported nodes^55^, all gaps were treated as missing data.

ML analyses were implemented in PhyML 3.1^56^ and RaxmlGUI 1.3^57^ under the appropriate models. Tree topology search operations were conducted using SPR (Subtree Pruning Regrafting) moves. Bootstrap support values (BS) for nodes on the topology calculated were based on 1000 replicates.

Bayesian analyses were performed by simultaneously running two Monte Carlo Markov (MCMC) chains for 50 million generations in MrBayes 3.1.2^58^. Each run has four chains, with trees sampled every 1000 generations. The average standard deviation of split frequencies and Potential Scale Reduction Factor (PSRF) were used for examining convergence. When stationarity was reached, the first 25% trees were discarded as burn-in and a consensus tree was obtained from the rest trees in the chain. Posterior probability values (PP) were considered as node support values. All tree topologies were displayed in Figtree 1.4^59^, rooted using outgroups.

### Models

PartitionFinder^60^ was used to determine the best-fitting model for each gene partition. The Bayesian information criterion (BIC) indicated that the GTR+I+G model was the best-fitting model for 28S rDNA, 16S rDNA and the position 1 and 2 of protein-coding genes. The most appropriate nucleotide substitution model for the position 3 was GTR+G. A mixed model Bayesian analysis was performed in Mrbayes, and GTR+I+G was conducted under ML analyses.

### Monophyletic tests

Monophyletic constrained topologies of 10 taxa (Table 3) were reconstructed by the combined data using RaxmlGUI. Each topology was tested against the optimal ML tree calculated in RaxmlGUI respectively. The log-likelihoods of 10 topologies (shown in Table 3) were estimated with PAUP 4.0b10. The Shimodaira-Hasegawa (SH) test and the Approximately Unbiased (AU) test were done in the software CONSEL 0.1j^61^.

**Table 3:** The log-likelihood scores and monophyletic test.

Group Tested	Constraint tree score	Best ML tree (−ln = 66657.05)		Results
		AU test	SH test	
Delphacidae	66620.61	0.677	0.682	Fail to Reject
Delphacinae	66632.41	0.628	0.616	Fail to Reject
Delphacini (without *Paranectopia*)	66721.48	0.208	0.206	Fail to Reject
Delphacini (with *Paranectopia*)	66710.27	0.253	0.260	Fail to Reject
Tropidocephalini (without *Paranectopia*)	66797.40	0.059	0.055	Fail to Reject
Tropidocephalini (with *Paranectopia*)	66785.77	0.045	0.050	Reject
*Megadelphax*	66626.70	0.673	0.664	Fail to Reject
*Bambusiphaga*	66580.71	0.846	0.847	Fail to Reject
*Opiconsiva*	66790.63	0.047	0.049	Reject
*Harmalia*	66783.73	0.054	0.062	Fail to Reject

### Divergence date estimation

BEAST 1.8.3^62^ was used under an uncorrelated normal relaxed clock assumption and a speciation Yule process to estimate the divergence time. Two mitochondrial protein-coding genes were selected to ensure the equal mutation rates. Chains were run for 50 million generations, with sampling every 5000 generations. Tracer 1.6.0 was used to verify the posterior distribution and to ensure the effective sample sizes (ESSs) >200 from the Markov Chain Monte Carlo (MCMC) output. TreeAnnotator in the BEAST package was used to summarize tree data with ‘mean height’ and discarded the first 25% of trees as the “burn-in” period. The results were visualized in FigTree.

Fossils of Cixiidae and Delphacini were used as calibrations to estimate chronograms. A normal distribution at 210 ± 4 Mya was set for Cixiidae^46, 63^. In addition, the fossil of *Delphax senilis* dated from the lower Eocene Ypresian (56 Mya) to Lutetian (41.2 Mya) were used to calibrate the most recent possible origin of Delphacini, with a prior normally distributed around 48.6 ± 3.8 Mya^35^.

### Data availability

All data generated or analysed during this study are included in this published article (and its Supplementary Information files).

## Electronic supplementary material

Dataset S1-S5
