# Supplementary material for: Resolving phylogenetic relationships of Delphacini and Tropidocephalini (Hemiptera: Delphacidae: Delphacinae) as inferred from four genetic loci
Source: Sci Rep. 2017 Jun 12;7:3319. doi: 10.1038/s41598-017-03624-w (PMC5468274; doi:10.1038/s41598-017-03624-w)
Supplement: Supplementary file 1 — Dataset S1-S5 [file 41598_2017_3624_MOESM1_ESM.doc]

# Resolving phylogenetic relationships of Delphacini and Tropidocephalini (Hemiptera: Delphacidae: Delphacinae) as inferred from four genetic loci

# Yi-Xin Huang^1^, Li-Fang Zheng^1^, Charles R. Bartlett^2^, Dao-Zheng Qin^1^*

**1** Key Laboratory of Plant Protection Resources and Pest Management of the Ministry of Education; Entomological Museum, Northwest A&F University, Yangling, Shaanxi 712100, China

**2** Department of Entomology and Wildlife Ecology, University of Delaware, Newark, DE, 19716, U.S.A.

^*^Corresponding author: Dr. Dao-Zheng Qin

Key Laboratory of Plant Protection Resources and Pest Management of the Ministry of Education; Entomological Museum, Northwest A&F University, Yangling, Shaanxi 712100, China

Email: [qindaozh0426@aliyun.com](mailto:qindaozh0426@aliyun.com), Phone: 86-29-87092524

**Supplementary** **Table S1** The sequence alignments of 28S rDNA.

| Taxa | The sequence alignments of 28S rDNA |
| --- | --- |
| *Aoyuanus furcatus* | CAG-GTGAGGGCCACGCGGAAGTCGCGCCGGATCGTACCCATATCCGCAGCAGGTCTCCAAGGTTAAGAGCCTCTAGTCGATAGAATAATGTAGGTAAGGGAAGTCGGCAAATTGGATC-CGTAACTTCGGAACAAGGATTGGCTC-TGAGGATCAGGGTGTGTCGGGCTAGGTTGGGATGTGGGTCAGCGCCAACGAGCCTGGCCTGAG---CACGAG-GAATGGAA-A---GCA-------CTTTGG---------TGCA-ATC-CAGGAGCCGAGCT-CGGTCCTGC-GC--CTTGGCCTCCCACGGATCGGCCTTGCTGCGGGG--GTCTGTTG-AC-AGTTGCA-------CAT-GGACTT--GT-CTG---------TGTGTTAACGCAGCAGAATTCA--CCTTCGGCCACCATTTAATGGTCAACTCAGAACTGGCACGGACTGGGAGAATCCGACTGTCTAATTAAAGCAAAGCATTGCGATGGCTCTAGCTGAGTGCTTACGCAATGTGATTTCTGCCCAGTGCTCTGAATGTCAACGTGAAGAAATTCAAGC-AAGCGCGGGTAAACGGCGGGAGTAACTATGACTCTCTTAAGGTAGCCAAATGCCTCGTCATCTAATTAGTGACGCGCATGAATGGATTAACGAGATTCTCA-CTGTCCCTATCTACTATCTAGCGAAACCACTGCCAAGGGAACGGGCTTGGAAAAATTAGCGGGGAAAGAAGACCCTGTTGAGCTTGACTCTAGTCTGG |
| *Arcofaciella verrucosa* | CAG-GTGAGGGCCACGCGGAAGTCGCGCCGGATCGTACCCATATCCGCAGCAGGTCTCCAAGGTTAAGAGCCTCTAGTCGATAGAATAATGTAGGTAAGGGAAGTCGGCAAATTGGATC-CGTAACTTCGGAACAAGGATTGGCTC-TGAGGATCGGGGCGTGTCGGGCTAGGTCGGGATGTGGGTCGGTGCCAACGAGCCTGGCCTGAG---CACGAG-GAACGGAG-A---ACA-------CTTCTG---------TGGG-CTCTCGGGATCCGAGCT-CGGTCCTGC-GC--CTTGGCCTCCCACGGATCGGCCTTGCTGCGGGG--GTCTGTTG-AC-AGTTACA-------CAA-AGACAT--GTGCTG---------AGTGTGGACGG-GCAGAATCTATCCCTTCGGCCGCCATTCAACGGTCAACTCAGAACTGGCACGGACCGGGAGAATCCGACTGTCTAATTAAAGCAAAGCATTGCGATGGCTCTAGCTGAGTGTTTACGCAATGTGATTTCTGCCCAGTGCTCTGAATGTCAACGTGAAGAAATTCAAGC-AAGCGCGGGTAAACGGCGGGAGTAACTATGACTCTCTTAAGGTAGCCAAATGCCTCGTCATCTAATTAGTGACGCGCATGAATGGATTAACGAGATTCTCA-CTGTCCCTATCTACTATCTAGCGAAACCACTGCCAAGGGAACGGGCTTGGAAAAATTAGCGGGGAAAGAAGACCCTGTTGAGCTTGACTCTAGTCTGG |
| *Arcofacies maculatipennis* | CAG-GTGAGGGCCACGCGGAAGTCGCGCCGGATCGTACCCATATCCGCAGCAGGTCTCCAAGGTTAAGAGCCTCTAGTCGATAGAATAATGTAGGTAAGGGAAGTCGGCAAATTGGATC-CGTAACTTCGGAACAAGGATTGGCTC-TGAGGATCGGGGCGTGTCGGGCTAGGTCGGGATGTGGGTCGGCGCCAACGAGCCTGGCCTGAG---CACGAG-GAACGGAG-T---GCA-------TCTCGG---------TGTG-CTT-CGGGATCCGAGCT-CGGTCCTGC-GC--CTTGGCCTCCCACGGATCGGCCTTGCTGCGGGG--GTCTGCTG-AC-AGTTGCA-------CAC-AGACTT--GT-TTG---------AGTGTGGACGG-GCAGAATCTATCCCTTCGGCCGCCATTCAACGGTCAACTCAGAACTGGCACGGACCGGGAGAATCCGACTGTCTAATTAAAGCAAAGCATTGCGATGGCTCTAGCTGAGTGTTTACGCAATGTGATTTCTGCCCAGTGCTCTGAATGTCAACGTGAAGAAATTCAAGC-AAGCGCGGGTAAACGGCGGGAGTAACTATGACTCTCTTAAGGTAGCCAAATGCCTCGTCATCTAATTAGTGACGCGCATGAATGGATTAACGAGATTCTCA-CTGTCCCTATCTACTATCTAGCGAAACCACTGCCAAGGGAACGGGCTTGGAAAAATTAGCGGGGAAAGAAGACCCTGTTGAGCTTGACTCTAGTCTGG |
| *Arcofacies strigatipennis* | CAG-GTGAGGGCCACGCGGAAGTCGCGCCGGATCGTACCCATATCCGCAGCAGGTCTCCAAGGTTAAGAGCCTCTAGTCGATAGAATAATGTAGGTAAGGGAAGTCGGCAAATTGGATC-CGTAACTTCGGAACAAGGATTGGCTC-TGAGGATCGGGGCGTGTCGGGCTAGGTCGGGATGTGGGTCGGCGCCAACGAGCCTTGCCTGAG---CCTGAG-GAACGGAG-T---GCA-------TTTCGG---------TGTTGCTC-CGGAAATCGAGCT-CGGTCTTGC-GC--CTTGGCCTCCCACGGATCGGCCTTGCTGCGGGG--GTCTGTTG-AC-AGTTGCA-------CGC-AGACAT--GCGCTG---------AGTGCGGACGG-GCAGAATCTATCCCTTCGGCCGCCATTCAACGGTCAACTCAGAACTGGCACGGACCGGGAGAATCCGACTGTCTAATTAAAGCAAAGCATTGCGATGGCTCTAGCTGAGTGTTTACGCAATGTGATTTCTGCCCAGTGCTCTGAATGTCAACGTGAAGAAATTCAAGC-AAGCGCGGGTAAACGGCGGGAGTAACTATGACTCTCTTAAGGTAGCCAAATGCCTCGTCATCTAATTAGTGACGCGCATGAATGGATTAACGAGATTCTCA-CTGTCCCTATCTACTATCTAGCGAAACCACTGCCAAGGGAACGGGCTTGGAAAAATTAGCGGGGAAAGAAGACCCTGTTGAGCTTGACTCTAGTCTGG |
| *Bakerella minuta* | CAG-GTGAGGGCCACGCGGAAGTCGCGCCAGATCGTACCCATATCCGCAGCAGGTCTCCAAGGTTAAGAGCCTCTAGTCAATAGACTAATGTAGGTAAGGGAAGTCGGCAAATTGGATC-CGTAACTTCGGGACAAGGATTGGCTC-TGAGGAGCGGGGTGTGTCGGGCTTGGTCGGGATGTGGGTCCTGGTC-ACGAGCCTGGCCTGAG---CACGTGTGAATGAAG-A---CCACACACTCTGTGTG---------TGCG-ATT-CAGGATCCG-TCT-CGGTCCAGT-GC--CTTGGCCTCCCACGGATCGGCCTTGCTGCGGGC--GTCTGTTG-AT-AGTTGCA-------CACGAGACTTCGGT-CCG---------AGTGTATACCT-TCAGAAAACA--CCTTCGGCCACTATTTAACGTTCAACTCAGAACTGGCACGGACCGGGAGAATCCGACTGTCTAATTAAAGCAAAGCATTGCGATGGCTCTAGCTGAGTGCTTACGCAATGTGATTTCTGCCCAGTGCTCTGAATGTCAACGTGAAGAAATTCAAGC-AAGCGCGGGTAAACGGCGGGAGTAACTATGACTCTCTTAAGGTAGCCAAATGCCTCGTCATCTAATTAGTGACGCGCATGAATGGATTAACGAGATTCTCA-CTGTCCCTATCTACTATCTAGCGAAACCACTGCCAAGGGAACGGGCTTGGAAAAATTAGCGGGGAAAGAAGACCCTGTTGAGCTTGACTCTAGTCTGG |
| *Bambusiphaga fascia* | CAG-GTGAGGGCCACGCGGAAGTCGCGCCGGATCGTACCCATATCCGCAGCAGGTCTCCAAGGTGAAGAGCCTCTAGTCGATAGAATAATGTAGGTAAGGGAAGTCGGCAAATTTGATC-CGTAACTTCGGAACAAGGATTGGCTC-TGATGATCGAGGCGTGTCGGGCTTGGTCAGGATGTGGGTCAGTGTCAAGAAGTTCAGTAGTAGTATCACGAG-AACCGG-------GTA------------A---------TGC------TGGGATGCGTGCTAAAAACCTGG-GC--CTTGGCCTGCCACGGATCGGCCTTGCTGCGGGA-TATCTGTTG-AC-AGT-GCA-------CTG-AGACTT--GTGCTG---------AGTGTAAACGG-GCAGAGTCTATCCCATCGGCCGTCATTTAACGATCCATACAGAACTGGCACGGACTTGGAGAATCCGACTGTTTAATTAAAACAAAGCATTGTGATGGCTATAGCTCAGTGTTTACGCAATGTGATTTCTGCCCAGTGCTCTGAATGTCAAAGTGAAGAAATTCAAGCTAAGCGCGGGTAAACGGCGGGAGTAACTATGACTCTCTTAAGGTAGCCAAATGCCTCGTCATCTAATTAGTGACGTGCATGAATGGATTAACGAGATTCTCA-CTGTCCCTATCTACTATCTAGCGAAACCACTGCCAAGGGAACGGGCTTGGAAAAATCAGCGGGGAAAGAAGACCCTGTTGAGCTTGACTCTAGTCTGG |
| *Bambusiphaga hainanensis* | CAG-GTGAGGGCCACGCGAAAGTCGCGCCGGATCGTACCCATATCCGCAGCAGGTCTCCAAGGTTAAGAGCCTCTAGTCGATAGAATAATGTAGGTAAGGGAAGTCGGCAAATTGGATC-CGTAACTTCGGAACAAGGATTGGCTC-TGAGGATCGGGGCGTGTCGGGCTAGGTCGGGATGTGGGTCGGCGCCAACGAGCCTGGCCTGAG---CACGAG-GAACGGAG-T---GTG-------CTTCGG---------TACG-CTT-CGGGATCCGAGCT-CGGTCCTGC-GC--CTTGGCCTCCCACGGATCGGCCTTGCTGCGGGG--GTCTGCTG-ACAAGTTGCA-------CAC-AGACTT--GTGCTG---------AGTGTGGACGG-GCAGAATCTATCCCTTCGGCCGCCATTCAACGGTCAACTCAGAACTGGCACGGACCGGGAGAATCCGACTGTCTAATTAAAGCAAAGCATTGCGATGGCTCTAGCTGAGTGTTCACGCAATGTGATTTCTGCCCAGTGCTCTGAATGTCAACGTGAAGAAATTCAAGC-AAGCGCGGGTAAACGGCGGGAGTAACTATGACTCTCTTAAGGTAGCCAAATGCCTCGTCATCTAATTAGTGACGCGCATGAATGGATTAACGAGATTCTCA-CTGTCCCTATCTACTATCTAGCGAAACCACTGCCAAGGGAACGGGCTTGGAAAAATTAGCGGGGAAAGAAGACCCTGTTGAGCTTGACTCTAGTCTGG |
| *Bambusiphaga kunmingensis* | CAG-GTGAGGGCCACGCGGAAGTCGCGCCGGATCGTACCCATATCCGCAGCAGGTCTCCAAGGTTAAGAGCCTCTAGTCGATAGAATAATGTAGGTAAGGGAAGTCGGCAAATTGGATC-CGTAACTTCGGAACAAGGATTGGCTC-TGAGGATCGGGGCGTGTCGGGCTAGGTCGGGATGTGGGTTTGCGCCAACGAGCCTGGCCTGAG---CACGAG-GAACGGAG-T---GTG-------CTTCGG---------TACG-CTT-CGGGAATCGAGCT-CGGTCCTGC-GC--CTTGGCCTACCACGGATCGGCCTTGCTGCGGGG--GTCTGTTG-AC-AGTTGCA-------CAC-AGACTT--GTGCTG---------TGTGTGGACGG-GCAGAATCTATCCCTTCGGCCGCCATTCAACGGTCAACTCAGAACTGGCACGGACCGGGAGAATCCGACTGTCTAATTAAAGCAAAGCATTGCGATGGCTCTAGCTGAGTGCTTACGCAATGTGATTTCTGCCCAGTGCTCTGAATGTCAACGTGAAGAAATTCAAGC-AAGCGCGGGTAAACGGCGGGAGTAACTATGACTCTCTTAAGGTAGCCAAATGCCTCGTCATCTAATTAGTGACGCGCATGAATGGATTAACGAGATTCTCA-CTGTCCCTATCTACTATCTAGCGAAACCACTGCCAAGGGAACGGGCTTGGAAAAATTAGCGGGGAAAGAAGACCCTGTTGAGCTTGACTCTAGTCTGG |
| *Bambusiphaga luodianensis* | CAG-GTGAGGGCCACGCGAAAGTCGCGCCGGATCGTACCCATATCCGCAGCAGGTCTCCAAGGTTAAGAGCCTCTAGTCGATAGAATAATGTAGGTAAGGGAAGTCGGCAAATTGGATC-CGTAACTTCGGAACAAGGATTGGCTC-TGAGGATCGGGGCGTGTCGGGCTAGGTCGGGATGTGGGTCGGCGCCAACGAGCCTGGCCTGAG---CACGAG-GAATGGAG-T---GTG-------CTTCTG---------TATG-CTC-CTGGATCCGAGCT-CGGTCCTGC-GC--CTTGGCCTCCCACGGATCGGCCTTGCTGCGGGG--GTCTGCTG-AC-AGTTGCA-------CAT-AGACTT--GT-CTG---------TGTGTGGACGG-GCAGAATCTATCCCTTCGGCCGCCATTCAACGGTCAACTCAGAACTGGCACGGACCGGGAGAATCCGACTGTCTAATTAAAGCAAAGCATTGCGATGGCTCTAGCTGAGTGTTTACGCAATGTGATTTCTGCCCAGTGCTCTGAATGTCAACGTGAAGAAATTCAAGC-AAGCGCGGGTAAACGGCGGGAGTAACTATGACTCTCTTAAGGTAGCCAAATGCCTCGTCATCTAATTAGTGACGCGCATGAATGGATTAACGAGATTCTCA-CTGTCCCTATCTACTATCTAGCGAAACCACTGCCAAGGGAACGGGCTTGGAAAAATTAGCGGGGAAAGAAGACCCTGTTGAGCTTGACTCTAGTCTGG |
| *Bambusiphaga maculata* | CAG-GTGAGGGCCACGCGGAAGTCGCGCCGGATCGTACCCATATCCGCAGCAGGTCTCCAAGGTTAAGAGCCTCTAGTCGATAGAATAATGTAGGTAAGGGAAGTCGGCAAATTGGATC-CGTAACTTCGGAACAAGGATTGGCTC-TGAGGATCGGGGCGTGTCGGGCTAGGTCGGGATGTGGGTCAGCGCCAACGAGCCAGGCCTGAG---CACGAG-GAACGGAG-T---GTA-------CTTCGG---------TGCA-TTT-CGCGGAACGAGCT-CGGTCCTGC-GC--CTTGGCCTCCCACGGATCGGCCTTGCTGCGGGG--GTCTGCTG-AC-AGTTGCA-------CTT-AGACTT--GTGCTG---------AGTGTGGACGG-GTAGAATCTATCCCTTCGGCCGCCATTCAACGGTCAACTCAGAACTGGCACGGACCGGGAGAATCCGACTGTCTAATTAAAGCAAAGCATTGCGATGGCTCTAGCTGAGTGTTTACGCAATGTGATTTCTGCCCAGTGCTCTGAATGTCAACGTGAAGAAATTCAAGC-AAGCGCGGGTAAACGGCGGGAGTAACTATGACTCTCTTAAGGTAGCCAAATGCCTCGTCATCTAATTAGTGACGCGCATGAATGGATTAACGAGATTCTCA-CTGTCCCTATCTACTATCTAGCGAAACCACTGCCAAGGGAACGGGCTTGGAAAAATTAGCGGGGAAAGAAGACCCTGTTGAGCTTGACTCTAGTCTGG |
| *Bambusiphaga membranacea* | CAG-GTGAGGGCCACGCGAAAGTCGCGCCGGATCGTACCCATATCCGCAGCAGGTCTCCAAGGTTAAGAGCCTCTAGTCGATAGAATAATGTAGGTAAGGGAAGTCGGCAAATTGGATC-CGTAACTTCGGAACAAGGATTGGCTC-TGAGGATCGGGGCGTGTCGGGCTAGGTCGGGATGTGGGTCGGCGCCAACGAGCCTGGCCTGAG---CACGAG-GAACGGAG-T---GTG-------CTTCGG---------TACG-CTT-CGGGATCCGAGCT-CGGTCCTGC-GC--CTTGGCCTCCCACGGATCGGCCTTGCTGCGGGG--GTCTGCTG-ACAAGTTGCA-------CAC-AGACTT--GTGCTG---------AGTGTGGACGG-GCAGAATCTATCCCTTCGGCCGCCATTCAACGGTCAACTCAGAACTGGCACGGACCGGGAGAATCCGACTGTCTAATTAAAGCAAAGCATTGCGATGGCTCTAGCTGAGTGTTCACGCAATGTGATTTCTGCCCAGTGCTCTGAATGTCAACGTGAAGAAATTCAAGC-AAGCGCGGGTAAACGGCGGGAGTAACTATGACTCTCTTAAGGTAGCCAAATGCCTCGTCATCTAATTAGTGACGCGCATGAATGGATTAACGAGATTCTCA-CTGTCCCTATCTACTATCTAGCGAAACCACTGCCAAGGGAACGGGCTTGGAAAAATTAGCGGGGAAAGAAGACCCTGTTGAGCTTGACTCTAGTCTGG |
| *Bambusiphaga mirostylis* | CAG-GTGAGGGCCACGCGGAAGTCGCGCCGGATCGTACCCATATCCGCAGCAGGTCTCCAAGGTTAAGAGCCTCTAGTCGATAGAATAATGTAGGTAAGGGAAGTCGGCAAATTGGATC-CGTAACTTCGGAACAAGGATTGGCTC-TGAGGATCGGGGCGTGTCGGGCTAGGTCGGGATGTGGGTCGGCGCCAACGAGCCTGGCCTGAG---CACGAG-GAACGGAG-T---GCG-------CTTCTG---------TGCG-CTT-CGGGATCCGAGCT-CGGTCCTGC-GC--CTTGGCCTCCCACGGATCGGCCTTGCTGCGGGG--GTCTGTTG-AC-AGTTGCA-------CAC-AGACTT--GTGCTG---------AGTGTGGACGG-GCAGAATCTATCCCTTCGGCCGCCATTCAACGGTCAACTCAGAACTGGCACGGACCGGGAGAATCCGACTGTCTAATTAAAGCAAAGCATTGCGATGGCTCTAGCTGAGTGTTTACGCAATGTGATTTCTGCCCAGTGCTCTGAATGTCAACGTGAAGAAATTCAAGC-AAGCGCGGGTAAACGGCGGGAGTAACTATGACTCTCTTAAGGTAGCCAAATGCCTCGTCATCTAATTAGTGACGCGCATGAATGGATTAACGAGATTCTCA-CTGTCCCTATCTACTATCTAGCGAAACCACTGCCAAGGGAACGGGCTTGGAAAAATTAGCGGGGAAAGAAGACCCTGTTGAGCTTGACTCTAGTCTGG |
| *Bambusiphaga nigropunctata* | CAG-GTGAGGGCCACGCGAAAGTCGCGCCGGATCGTACCCATATCCGCAGCAGGTCTCCAAGGTTAAGAGCCTCTAGTCGATAGAATAATGTAGGTAAGGGAAGTCGGCAAATTGGATC-CGTAACTTCGGAACAAGGATTGGCTC-TGAGGATCGGGGCGTGTCGGGCTAGGTCGGGATGTGGGTCGGTGCCAACGAGCCTGGCCTGAG---CACGAG-GAACGGAG----------------TTCTG--------------CTT-CGGGATCCGAGCT-CGGTCCTGC-GC--CTTGGCCTCCCACGGAGCGGCCTTGCTGCGGGG--GTCTGCTG-ACAAGTTGCA-------CAC-AGACTT--GTGCTG---------AGTGTGGACGG-GCAGAATCTATCCCTTCGGCCGCCATTCAACGGTCAACTCAGAACTGGCACGGACCGGGAGAATCCGACTGTCTAATTAAAGCAAAGCATTGCGATGGCTCTAGCTGAGTGTTTACGCAATGTGATTTCTGCCCAGTGCTCTGAATGTCAACGTGAAGAAATTCAAGC-AAGCGCGGGTAAACGGCGGGAGTAACTATGACTCTCTTAAGGTAGCCAAATGCCTCGTCATCTAATTAGTGACGCGCATGAATGGATTAACGAGATTCTCA-CTGTCCCTATCTACTATCTAGCGAAACCACTGCCAAGGGAACGGGCTTGGAAAAATTAGCGGGGAAAGAAGACCCTGTTGAGCTTGACTCTAGTCTGG |
| *Bambusiphaga taibaishana* | CAG-GTGAGGGCCACGCGGAAGTCGCGCCGGATCGTACCCATATCCGCAGCAGGTCTCCAAGGTGAAGAGCCTCTAGTCGATAGAATAATGTAGGTAAGGGAAGTCGGCAAATTTGATC-CGTAACTTCGGAACAAGGATTGGCTC-TGATGATCGAGGCGTGTCGGGCTTGGTCAGGATGTGGGTCAGTGTCAAGAAGTTCAGTAGTAGTATCACGAG-AACCGG-------GTA------------A---------TGC------TGGGATGCGTGCTAAAAACCTGA-GC--CTTGGCCTGCCACGGATCGGCCTTGCTGCGGGG-TATCTGTTG-AC-AGT-GCA-------CTC-AGACTT--GTGCTG---------AGTGTAAACGG-GCAGAGTCTATCCCATCGGCCGTCATTTAACGATCCATACAGAACTGGCACGGACTTGGAGAATCCGACTGTTTAATTAAAACAAAGCATTGTGATGGCTATAGCTCAGTGTTCACGCAATGTGATTTCTGCCCAGTGCTCTGAATGTCAAAGTGAAGAAATTCAAGCTAAGCGCGGGTAAACGGCGGGAGTAACTATGACTCTCTTAAGGTAGCCAAATGCCTCGTCATCTAATTAGTGACGTGCATGAATGGATTAACGAGATTCTCA-CTGTCCCTATCTACTATCTAGCGAAACCACTGCCAAGGGAACGGGCTTGGAAAAATCAGCGGGGAAAGAAGACCCTGTTGAGCTTGACTCTAGTCTGG |
| *Bostaera nasuta* | ---------GGCCACGCAGTTGTCGCGCCGGATCGTACCCATATCCGCAGCAGGTCTCCAAGGTTAAGAGCCTCTAGTCGATAGAATAATGTAGGTAAGGGAAGTCGGCAAATTGGATC-CGTAACTTCGGAACAAGGATTGGCTC-TGAGGATCGGGGTGTGTCGGGCTAGGTTGGGATGTGGGTCAGCGCCAACGAGCCTGGCCTGAG---CACGAG-GAATGGAA-A---GCA-------TTTCGG---------TGCA-ATC-CAGGAGCCGAGCT-CGGTCCTGC-GC--CTTGGCCTCCCACGGATCGGCCTTGCTGCGGGG--GTCTGTTG-AT-AGTTGCG-------CAT-GGACTT--GT-TCG---------TGTGTCAACCA-GCAGAATTCA--CCTTCGGCCACCATTTAACGGTCAACTCAGAACTGGCACGGACCGGGAGAATCCGACTGTCTAATTAAAGCAAAGCATTGCGATGGCTCTAGCTGAGTGCTTACGCAATGTGATTTCTGCCCAGTGCTCTGAATGTCAACGTGAAGAAATTCAAGC-AAGCGCGGGTAAACGGCGGGAGTAACTATGACTCTCTTAAGGTAGCCAAATGCCTCGTCATCTAATTAGTGACGCGCATGAATGGATTAACGAGATTCTCA-CTGTCCCTATCTACTATCTAGCGAAACCACTGCCAAGGGAACGGGCTTGGAAAAATTAGCGGGGAAAGAAGACCCTGTTGAGCTTGACTCTAGTCTGG |
| *Caenodelphax teapae* | CAG-GTGAGGGCCACGCGGAAGTCGCGCCAGATCGTACCCATATCCGCAGCAGGTCTCCAAGGTTAAGAGCCTCTAGTCGATAGAATAATGTAGGTAAGGGAAGTCGGCAAATTGGATC-CGTAACTTCGGGACAAGGATTGGCTC-TGAGGATCGGGGTGTGTCGGGCTAGGTCGGGATGTGGGTCAGCGCCAACGAGCCTGGCCTGAG---CACGAG-GAATGGAG-A---GCA-------TCTCGG---------TGCA-ATC-CAGGATCCGAGCT-CGGTCCTGC-GC--CTTGGCCTCCCACGGATCGGCCTTGCTGCGGGG--GTCTGTTG-AC-AGTTGCA-------CAC-AGACTT--GT-CTG---------TGTGTTAACCA-GCAGAATTCA--CCTTCGGCCACCATTTAACGGTCAACTCAGAACTGGCACGGACCGGGAGAATCCGACTGTCTAATTAAAGCAAAGCATTGCGATGGCTCTAGCTGAGTGTTTACGCAATGTGATTTCTGCCCAGTGCTCTGAATGTCAACGTGAAGAAATTCAAGC-AAGCGCGGGTAAACGGCGGGAGTAACTATGACTCTCTTAAGGTAGCCAAATGCCTCGTCATCTAATTAGTGACGCGCATGAATGGATTAACGAGATTCTCA-CTGTCCCTATCTACTATCTAGCGAAACCACTGCCAAGGGAACGGGCTTGGAAAAATTAGCGGGGAAAGAAGACCCTGTTGAGCTTGACTCTAGTCTGG |
| *Carinofrons maculatipennis* | CAG-GTGAGGGCCACGCGGAAGTCGCGCCGGATCGTACCCATATCCGCAGCAGGTCTCCAAGGTTAAGAGCCTCTAGTCGATAGAATAATGTAGGTAAGGGAAGTCGGCAAATTGGATC-CGTAACTTCGGGACAAGGATTGGCTC-TGAGGATCGGGGTGTGTCGGGCTAGGTCAGGATGTGGGTCAGCGCCAACGAGCCAGGCCTGAG---CACGAG-GAATGGAG-A---GCA-------CTTCGG---------TGCA-ATC-CAGGATCCGAGCT-CGGTCCTGC-GC--CTTGGCCTCCCACGGATTGGCCTTGCTGCGGGG--GTCTGTTG-AC-AGTTGCG-------CAT-AGACTT--GT-CTG---------TGCGTTAACCA-GCAGAATTCA--CCTTCGGCCACCATTTAACGGTCAACTCAGAACTGGCACGGACCGGGAGAATCCGACTGTCTAATTAAAGCAAAGCATTGCGATGGCTCTAGCTGAGTGTTCACGCAATGTGATTTCTGCCCAGTGCTCTGAATGTCAACGTGAAGAAATTCAAGC-AAGCGCGGGTAAACGGCGGGAGTAACTATGACTCTCTTAAGGTAGCCAAATGCCTCGTCATCTAATTAGTGACGCGCATGAATGGATTAACGAGATTCTCA-CTGTCCCTATCTACTATCTAGCGAAACCACTGCCAAGGGAACGGGCTTGGAAAAATTAGCGGGGAAAGAAGACCCTGTTGAGCTTGACTCTAGTCTGG |
| *Cemopsis griphus* | CAG-GTGAGGGCCACGCGGAAGTCGCGCCGGATCGTACCCATATCCGCAGCAGGTCTCCAAGGTTAAGAGCCTCTAGTCGATAGAATAATGTAGGTAAGGGAAGTCGGCAAATTGGATC-CGTAACTTCGGAACAAGGATTGGCTC-TGAGGATCAGGGTGTGTCGGGCTAGGTTGGGATGTGGGTCAGCGCCAACGAGCCTGGCCTGAG---CACGAG-GAATGGAG-A---GCA-------TTTCGG---------TGCA-ATT-CAGGAGCCGAGCT-CGGTCCTGC-GC--CTTGGCCTCCCACGGATCGGCCTTGCTGCGGGG--GTCTGTTG-AC-AGTTGCA-------CGC-AGACTT--GT-TTG---------TGTGTTAACGCAGCAGAATTCA--CCTTCGGCCACCATTTAATGGTCAACTCAGAACTGGCACGGACTGGGAGAATCCGACTGTCTAATTAAAGCAAAGCATTGCGATGGCTCTAGCTGAGTGCTTACGCAATGTGATTTCTGCCCAGTGCTCTGAATGTCAACGTGAAGAAATTCAAGC-AAGCGCGGGTAAACGGCGGGAGTAACTATGACTCTCTTAAGGTAGCCAAATGCCTCGTCATCTAATTAGTGACGCGCATGAATGGATTAACGAGATTCTCA-CTGTCCCTATCTACTATCTAGCGAAACCACTGCCAAGGGAACGGGCTTGGAAAAATTAGCGGGGAAAGAAGACCCTGTTGAGCTTGACTCTAGTCTGG |
| *Cemus macaoensis* | CAG-GTGAGGGCCACGCGGAAGTCGCGCCGGATCGTACCCATATCCGCAGCAGGTCTCCAAGGTTAAGAGCCTCTAGTCGATAGAATAATGTAGGTAAGGGAAGTCGGCAAATTGGATC-CGTAACTTCGGAACAAGGATTGGCTC-TGAGGATCAGGGTGTGTCGGGCTAGGTTGGGATGTGGGTCAGCGCCAACGAGCCTGGCCTGAG---CACGAG-GAATGGAG-A---GCA-------TTTCGG---------TGCA-ATC-CAGGAGCCGAGCT-CGGTCCTGC-GC--CTTGGCCTCCCACGGATCGGCCTTGCTGCGGGG--GTCTGTTG-AC-AGTTGCA-------CAT-GGACTT--GT-CTG---------TGTGTTAACGCAGCAGAATTCA--CCTTCGGCCACCATTTAATGGTCAACTCAGAACTGGCACGGACTGGGAGAATCCGACTGTCTAATTAAAGCAAAGCATTGCGATGGCTCTAGCTGAGTGCTTACGCAATGTGATTTCTGCCCAGTGCTCTGAATGTCAACGTGAAGAAATTCAAGC-AAGCGCGGGTAAACGGCGGGAGTAACTATGACTCTCTTAAGGTAGCCAAATGCCTCGTCATCTAATTAGTGACGCGCATGAATGGATTAACGAGATTCTCA-CTGTCCCTATCTACTATCTAGCGAAACCACTGCCAAGGGAACGGGCTTGGAAAAATTAGCGGGGAAAGAAGACCCTGTTGAGCTTGACTCTAGTCTGG |
| *Cemus nigromaculosus* | CAG-GTGAGGGCCACGCGGAAGTCGCGCCGGATCGTACCCATATCCGCAGCAGGTCTCCAAGGTTAAGAGCCTCTAGTCGATAGAATAATGTAGGTAAGGGAAGTCGGCAAATTGGATC-CGTAACTTCGGAACAAGGATTGGCTC-TGAGGATCAGGGTGTGTCGGGCTAGGTTGGGATGTGGGTCAGCGCCAACGAGCCTGGCCTGAG---CACGAG-GAATGGAG-A---GCA-------TTTCGG---------TGCG-ATC-CAGGAGCCGAGCT-CGGTCCTGC-GC--CTTGGCCTCCCACGGATCGGCCTTGCTGCGGGG--GTCTGTTA-AC-AGTTGCA-------CAT-GGACTT--GT-CTG---------TGTGTTAACGTAGCAGAATTCA--CCTTCGGCCACCATTTAATGGTCAACTCAGAACTGGCACGGACTGGGAGAATCCGACTGTCTAATTAAAGCAAAGCATTGCGATGGCTCTAGCTGAGTGCTTACGCAATGTGATTTCTGCCCAGTGCTCTGAATGTCAACGTGAAGAAATTCAAGC-AAGCGCGGGTAAACGGCGGGAGTAACTATGACTCTCTTAAGGTAGCCAAATGCCTCGTCATCTAATTAGTGACGCGCATGAATGGATTAACGAGATTCTCA-CTGTCCCTATCTACTATCTAGCGAAACCACTGCCAAGGGAACGGGCTTGGAAAAATTAGCGGGGAAAGAAGACCCTGTTGAGCTTGACTCTAGTCTGG |
| *Cemus punctatus* | CAG-GTGAGGGCCACGCGGAAGTCGCGCCGGATCGTACCCATATCCGCAGCAGGTCTCCAAGGTTAAGAGCCTCTAGTCGATAGAATAATGTAGGTAAGGGAAGTCGGCAAATTGGATC-CGTAACTTCGGAACAAGGATTGGCTC-TGAGGATCAGGGTGTGTCGGGCTAGGTTGGGATGTGGGTCAGCGCCAACGAGCCTGGCCTGAG---CACGAG-GAATGGAG-A---GCA-------TTTCGG---------TGCG-ATC-CAGGAGCCGAGCT-CGGTCCTGC-GC--CTTGGCCTCCCACGGATCGGCCTTGCTGCGGGG--GTCTGTTG-AC-AGTTGCA-------CAT-GGACTT--GT-CTG---------TGTGTTAACGCAGCAGAATTCA--CCTTCGGCCACCATTTAATGGTCAACTCAGAACTGGCACGGACTGGGAGAATCCGACTGTCTAATTAAAGCAAAGCATTGCGATGGCTCTAGCTGAGTGCTTACGCAATGTGATTTCTGCCCAGTGCTCTGAATGTCAACGTGAAGAAATTCAAGC-AAGCGCGGGTAAACGGCGGGAGTAACTATGACTCTCTTAAGGTAGCCAAATGCCTCGTCATCTAATTAGTGACGCGCATGAATGGATTAACGAGATTCTCA-CTGTCCCTATCTACTATCTAGCGAAACCACTGCCAAGGGAACGGGCTTGGAAAAATTAGCGGGGAAAGAAGACCCTGTTGAGCTTGACTCTAGTCTGG |
| *Cemus sauteri* | CAG-GTGAGGGCCACGCGGAAGTCGCGCCGGATCGTACCCATATCCGCAGCAGGTCTCCAAGGTTAAGAGCCTCTAGTCGATAGAATAATGTAGGTAAGGGAAGTCGGCAAATTGGATC-CGTAACTTCGGAACAAGGATTGGCTC-TGAGGATCAGGGTGTGTCGGGCTAGGTTGGGATGTGGGTCAGCGCCAACGAGCCTGGCCTGAG---CACGAG-GAATGGAG-A---GCA-------TTTCGG---------TGCG-ATC-CAGGAGCCGAGCT-CGGTCCTGC-GC--CTTGGCCTCCCACGGATCGGCCTTGCTGCGGGG--GTCTGTTA-AC-AGTTGCA-------CAT-GGACTT--GT-CTG---------TGTGTTAACGTAGCAGAATTCA--CCTTCGGCCACCATTTAATGGTCAACTCAGAACTGGCACGGACTGGGAGAATCCGACTGTCTAATTAAAGCAAAGCATTGCGATGGCTCTAGCTGAGTGCTTACGCAATGTGATTTCTGCCCAGTGCTCTGAATGTCAACGTGAAGAAATTCAAGC-AAGCGCGGGTAAACGGCGGGAGTAACTATGACTCTCTTAAGGTAGCCAAATGCCTCGTCATCTAATTAGTGACGCGCATGAATGGATTAACGAGATTCTCA-CTGTCCCTATCTACTATCTAGCGAAACCACTGCCAAGGGAACGGGCTTGGAAAAATTAGCGGGGAAAGAAGACCCTGTTGAGCTTGACTCTAGTCTGG |
| *Cixius scrupeus* | CGG-GTGAGAGCGACGCGGTGATCGCGCCGGTTCGTATCCATATCCGCATCTGGTCTCCAAGGTTCAAAGCCTCTTGTCGATAGAATAATGTAGGTAAGGGAAGTCGGCAAATTGGATC-CGTAACTTCGGGACAAGGATTGGCTC-TGAGGATCGGGGCGTGTCGGGCTTGGTCGGGAAGCGGGTCAGTGCCAACGTGCCGGGCCTGGG---C------GAGTGTCGTGTTGGTA------TCCTTGGCAGCAATGTCGAGTTTCCCACGATCCGAACT-CGGTCCCGT-GCCACTTGGCCTCCCGCGGATCTTCCTTGCTGCGGGT-----TTTCG-ACCAA--GCA-------GACCAGGCTG--GG-CCGCTTCAGGCCCGCTTGGACGGCTTCGAATCAATCCCTTCGGCCGCCATTCAACGGTCAACTCAGAACTGGCACGGACAAGGAGAATCCGACTGCCTAATTAAAGCAAAGCATTGCGAGGGCTCTAGCCGAGTGCTTACGCAATGTGATTTCTGCCCAGTGCTCTGAATGTCAACGTGTAGAAATTCAAGC-AAGCGCGGGTAAACGGCGGGAGTAACTATGACTCTCTTAAGGTAGCCAAATGCCTCGTCATCCAATTAGTGACGCGCATGAATGGATTAACGAGATTCTCA-CTGTCCCTATCTACTATCTAGCGAAACCACAGCCAAGGGAACGGGCTTGGAAACATTAGCGGGGAAAGAAGACCCTGTTGAGCTTGACTCTAGTCTGG |
| *Criomorphus niger* | CAG-GTGAGGGCCACGCGGAAGTCGCGCCGGATCGTACCCATATCCGCAGCAGGTCTCCAAGGTTAAGAGCCTCTAGTCGATAGAATAATGTAGGTAAGGGAAGTCGGCAAATTGGATC-CGTAACTTCGGGACAAGGATTGGCTC-TGAGGATCGGGGTGTGTCGGGCTAGGTCGGGATGTGGGTCAGCGCCAACGAGCCTGGCCTGAG---CACGAG-GAATGGAG-A---GCA-------TTTCGG---------TGCG-ATC-CAGGATCCGAGCT-CGGTCCTGC-GC--CTTGGCCTCCCACGGATCGGCCTTGCTGCGGGG--GTCTGTTG-AC-AGTTGCA-------TAC-AGACTT--GT-CTG---------TGTGTTAACCA-GCAGAATTCA--CCTTCGGCCACCATTTAACGGTCAACTCAGAACTGGCACGGACCGGGAGAATCCGACTGTCTAATTAAAGCAAAGCATTGCGATGGCTCTAGCTGAGTGTTTACGCAATGTGATTTCTGCCCAGTGCTCTGAATGTCAACGTGAAGAAATTCAAGC-AAGCGCGGGTAAACGGCGGGAGTAACTATGACTCTCTTAAGGTAGCCAAATGCCTCGTCATCTAATTAGTGACGCGCATGAATGGATTAACGAGATTCTCA-CTGTCCCTATCTACTATCTAGCGAAACCACTGCCAAGGGAACGGGCTTGGAAAAATTAGCGGGGAAAGAAGACCCTGTTGAGCTTGACTCTAGTCTGG |
| *Delphacodes penedetecta* | CAG-GTGAGGGCCACGCGGAAGTCGCGCCGGATCGTACCCATATCCGCAGCAGGTCTCCAAGGTTAAGAGCCTCTAGTCGATAGAATAATGTAGGTAAGGGAAGTCGGCAAATTGGATC-CGTAACTTCGGGACAAGGATTGGCTC-TGAGGATCGGGGTGTGTCGGGCTAGGTCGGGATGTGGGTCAGCGCCAACGAGCCTGGCCTGAG---CACGAG-GAATGGAG-A---GCA-------TTTCGG---------TGCG-ATC-CAGGATCCGAGCT-CGGTCCTGC-GC--CTTGGCCTCCCACGGATCGGCCTTGCTGCGGGG--GTCTGTTG-AC-AGTTGCA-------CAT-GGATAC--GT-TCG---------TGTGTTAACCA-GCAGAATTCA--CCTTCGGCCACCATTTAACGGTCAACTCAGAACTGGCACGGACCGGGAGAATCCGACTGTCTAATTAAAGCAAAGCATTGCGATGGCTCTAGCTGAGTGTTTACGCAATGTGATTTCTGCCCAGTGCTCTGAATGTCAACGTGAAGAAATTCAAGC-AAGCGCGGGTAAACGGCGGGAGTAACTATGACTCTCTTAAGGTAGCCAAATGCCTCGTCATCTAATTAGTGACGCGCATGAATGGATTAACGAGATTCTCA-CTGTCCCTATCTACTATCTAGCGAAACCACTGCCAAGGGAACGGGCTTGGAAAAATTAGCGGGGAAAGAAGACCCTGTTGAGCTTGACTCTAGTCTGG |
| *Delphacodes puella* | CAG-GTGAGGGCCACGCGGAAGTCGCGCCAGATCGTACCCATATCCGCAGCAGGTCTCCAAGGTTAAGAGCCTCTAGTCGATAGAATAATGTAGGTAAGGGAAGTCGGCAAATTGGATC-CGTAACTTCGGGACAAGGATTGGCTC-TGAGGATCGGGGTGTGTCGGGCTAGGTCGGGATGTGGGTCAGCGCCAACGAGCCTGGCCTGAG---CACGAG-GAATGGAG-A---GCA-------TTTCGG---------TGC--ATC-CAGGATCCGAGCT-CGGTCCTGC-GC--CTTGGCCTCCCACGGATCGGCCTTGCTGCGGGG--GTCTGTTG-AC-AGTTGCA-------CAC-AGACTT--GT-CTG---------TGTGTTAACCA-GCAGAATTCA--CCTTCGGCCACCATTTAACGGTCAACTCAGAACTGGCACGGACCGGGAGAATCCGACTGTCTAATTAAAGCAAAGCATTGCGATGGCTCTAGCTGAGTGTTTACGCAATGTGATTTCTGCCCAGTGCTCTGAATGTCAACGTGAAGAAATTCAAGC-AAGCGCGGGTAAACGGCGGGAGTAACTATGACTCTCTTAAGGTAGCCAAATGCCTCGTCATCTAATTAGTGACGCGCATGAATGGATTAACGAGATTCTCA-CTGTCCCTATCTACTATCTAGCGAAACCACTGCCAAGGGAACGGGCTTGGAAAAATTAGCGGGGAAAGAAGACCCTGTTGAGCTTGACTCTAGTCTGG |
| *Delphax orientalis* | CAG-GTGAGGGCCACGCGGAAGTCGCGCCGGATCGTACCCATATCCGCAGCAGGTCTCCAAGGTTAAGAGCCTCTAGTCGATAGAATAATGTAGGTAAGGGAAGTCGGCAAATTGGATC-CGTAACTTCGGGACAAGGATTGGCTC-TGAGGATCGGGGTGTGTCGGGCTAGGTTGGGATGTGGGTCAGCGCCAACGAGCCTGGCCTGAG---CACGAG-GAATGGAG-A---GCA-------TTTCGG---------TGCA-ATC-CAGGAGCCGAGCT-CGGTCCTGC-GC--CTTGGCCTCCCACGGATCGGCCTTGCTGCGGGG--GTCTGTTG-AC-AGTTGCA-------TAC-AGATTT--AT-CTG---------TGTGTTAACCA-GCAGAATTCA--CCTTCGGCCACCATTTAACGGTCAACTCAGAACTGGCACGGACCGGGAGAATCCGACTGTCTAATTAAAGCAAAGCATTGCGATGGCTCTAGCTGAGTGCTTACGCAATGTGATTTCTGCCCAGTGCTCTGAATGTCAACGTGAAGAAATTCAAGC-AAGCGCGGGTAAACGGCGGGAGTAACTATGACTCTCTTAAGGTAGCCAAATGCCTCGTCATCTAATTAGTGACGCGCATGAATGGATTAACGAGATTCTCA-CTGTCCCTATCTACTATCTAGCGAAACCACTGCCAAGGGAACGGGCTTGGAAAAATTAGCGGGGAAAGAAGACCCTGTTGAGCTTGACTCTAGTCTGG |
| *Dicranotropis hamata* | CAG-GTGAGGGCCACGCGGAAGTCGCGCCGGATCGTACCCATATCCGCAGCAGGTCTCCAAGGTTAAGAGCCTCTAGTCGATAGAATAATGTAGGTAAGGGAAGTCGGCAAATTGGATC-CGTAACTTCGGGACAAGGATTGGCTC-TGAGGATCGGGGTGTGTCGGGCTAGGTCGGGATGTGGGTCAGCGCCAACGAGCCTGGCCTGAG---CACGAG-GAATGGAG-A---GCA-------TTTCGG---------TGCG-ATC-CAGGATCCGAGCT-CGGTCCTGC-GC--CTTGGCCTCCCACGGATCGGCCTTGCTGCGGGG--GTCTGTTG-AC-AGTTGCA-------CAT-GGACTT--GT-TTG---------TGTGTCAACCA-GCAGAATTCA--CCTTCGGCCACCATTTAACGGTCAACTCAGAACTGGCACGGACCGGGAGAATCCGACTGTCTAATTAAAGCAAAGCATTGCGATGGCTCTAGCTGAGTGTTTACGCAATGTGATTTCTGCCCAGTGCTCTGAATGTCAACGTGAAGAAATTCAAGC-AAGCGCGGGTAAACGGCGGGAGTAACTATGACTCTCTTAAGGTAGCCAAATGCCTCGTCATCTAATTAGTGACGCGCATGAATGGATTAACGAGATTCTCA-CTGTCCCTATCTACTATCTAGCGAAACCACTGCCAAGGGAACGGGCTTGGAAAAATTAGCGGGGAAAGAAGACCCTGTTGAGCTTGACTCTAGTCTGG |
| *Distantinus melichari* | CAG-GTGAGGGCCACGCGGAAGTCGCGCCAGATCGTACCCATATCCGCAGCAGGTCTCCAAGGTTAAGAGCCTCTAGTCGATAGAATAATGTAGGTAAGGGAAGTCGGCAAATTGGATC-CGTAACTTCGGGACAAGGATTGGCTC-TGAGGATCGGGGTGTGTCGGGCTAGGTCGGGATGTGGGTTAGCGCCAACGAGCCTGGCCTGAG---CACGAG-GAATGGAG-A---GCA-------TTTCGG---------TGCA-ATC-CAGGATCCGAGTT-CGGTCCTGC-GC--CTTGGCCTCCCACGGATCGGCCTTGCTGCGGGG--GTCTGTTG-AC-AGTTGCA-------CAC-GGACTT--GT-CTG---------TGTGTTAACCA-GCAGAATTCA--CCTTCGGCCACCATTTAACGGTCAACTCAGAACTGGCACGGACCGGGAGAATCCGACTGTCTAATTAAAGCAAAGCATTGCGATGGCTCTAGCTGAGTGTTTACGCAATGTGATTTCTGCCCAGTGCTCTGAATGTCAACGTGAAGAAATTCAAGC-AAGCGCGGGTAAACGGCGGGAGTAACTATGACTCTCTTAAGGTAGCCAAATGCCTCGTCATCTAATTAGTGACGCGCATGAATGGATTAACGAGATTCTCA-CTGTCCCTATCTACTATCTAGCGAAACCACTGCCAAGGGAACGGGCTTGGAAAAATTAGCGGGGAAAGAAGACCCTGTTGAGCTTGACTCTAGTCTGG |
| *Ecdelphax cervina* | CAG-GTGAGGGCCACGCGGAAGTCGCGCCGGATCGTACCCATATCCGCAGCAGGTCTCCAAGGTTAAGAGCCTCTAGTCGATAGAATAATGTAGGTAAGGGAAGTCGGCAAATTGGATC-CGTAACTTCGGAACAAGGATTGGCTC-TGAGGATCAGGGTGTGTCGGGCTAGGTTGGGATGTGGGTCAGCGCCAACGAGCCTGGCCTGAG---CACGAG-GAATGGGG-A---GCA-------TTTCGG---------TGCA-ATC-CAGGAGCCGAGCT-CGGTCCTGC-GC--CTTGGCCTCCCACGGATCGGCCTTGCTGCGGGG--GTCTGTTG-AC-AGTTGCA-------CAC-GGACTT--GT-CTG---------TGTGTTAACGCAGCAGAATTCA--CCTTCGGCCACCATTTAATGGTCAACTCAGAACTGGCACGGACTGGGAGAATCCGACTGTCTAATTAAAGCAAAGCATTGCGATGGCTCTAGCTGAGTGCTTACGCAATGTGATTTCTGCCCAGTGCTCTGAATGTCAACGTGAAGAAATTCAAGC-AAGCGCGGGTAAACGGCGGGAGTAACTATGACTCTCTTAAGGTAGCCAAATGCCTCGTCATCTAATTAGTGACGCGCATGAATGGATTAACGAGATTCTCA-CTGTCCCTATCTACTATCTAGCGAAACCACTGCCAAGGGAACGGGCTTGGAAAAATTAGCGGGGAAAGAAGACCCTGTTGAGCTTGACTCTAGTCTGG |
| *Ecdelphax dentata* | CAG-GTGAGGGCCACGCGGAAGTCGCGCCGGATCGTACCCATATCCGCAGCAGGTCTCCAAGGTTAAGAGCCTCTAGTCGATAGAATAATGTAGGTAAGGGAAGTCGGCAAATTGGATC-CGTAACTTCGGAACAAGGATTGGCTC-TGAGGATCAGGGTGTGTCGGGCTAGGTTGGGATGTGGGTCAGCGCCAACGAGCCTGGCCTGAG---CACGAG-GAATGGGG-A---GCA-------TTTCGG---------TGCA-ATC-CAGGAGCCGAGCT-CGGTCCTGC-GC--CTTGGCCTCCCACGGATCGGCCTTGCTGCGGGG--GTCTGTTG-AC-AGTTGCA-------CAC-GGACTT--GT-CTG---------TGTGTTAACGCAGCAGAATTCA--CCTTCGGCCACCATTTAATGGTCAACTCAGAACTGGCACGGACTGGGAGAATCCGACTGTCTAATTAAAGCAAAGCATTGCGATGGCTCTAGCTGAGTGCTTACGCAATGTGATTTCTGCCCAGTGCTCTGAATGTCAACGTGAAGAAATTCAAGC-AAGCGCGGGTAAACGGCGGGAGTAACTATGACTCTCTTAAGGTAGCCAAATGCCTCGTCATCTAATTAGTGACGCGCATGAATGGATTAACGAGATTCTCA-CTGTCCCTATCTACTATCTAGCGAAACCACTGCCAAGGGAACGGGCTTGGAAAAATTAGCGGGGAAAGAAGACCCTGTTGAGCTTGACTCTAGTCTGG |
| *Epeurysa distincta* | CAG-GTGAGGGCCACGCGGAAGTCGCGCCGGATCGTACCCATATCCGCAGCAGGTCTCCAAGGTTAAGAGCCTCTAGTCGATAGAATAATGTAGGTAAGGGAAGTCGGCAAATTGGATC-CGTAACTTCGGAACAAGGATTGGCTC-TGAGGATCGGGGCGTGTCGGGCTAGGTCGGGATGTGGGTCGGCGCCAACGAGCCTGGCCTGAG---CACGAG-GAACGGAG-T---GCA-------TTTCGG---------TGCG-CTT-CGGGATCCGAGCT-CGGTCCTGC-GC--CTTGGCCTCCCACGGATCGGCCTTGCTGCGGGG--GTCTGCTG-AC-AGTTGCA-------CAC-AGACTT--GT-CTG---------AGTGTGGACGG-GCAGAATCTTACCCTTCGGCCGCCATTCAACGGTCAACTCAGAACTGGCACGGACCGGGAGAATCCGACTGTCTAATTAAAGCAAAGCATTGCGATGGCTCCAGCTGAGTGTTCACGCAATGTGATTTCTGCCCAGTGCTCTGAATGTCAACGTGAAGAAATTCAAGC-AAGCGCGGGTAAACGGCGGGAGTAACTATGACTCTCTTAAGGTAGCCAAATGCCTCGTCATCTAATTAGTGACGCGCATGAATGGATTAACGAGATTCTCA-CTGTCCCTATCTACTATCTAGCGAAACCACTGCCAAGGGAACGGGCTTGGAAAAATTAGCGGGGAAAGAAGACCCTGTTGAGCTTGACTCTAGTCTGG |
| *Epeurysa infumata* | CAG-GTGAGGGCCACGCGGAAGTCGCGCCGGATCGTACCCATATCCGCAGCAGGTCTCCAAGGTTAAGAGCCTCTAGTCGATAGAATAATGTAGGTAAGGGAAGTCGGCAAATTGGATC-CGTAACTTCGGAACAAGGATTGGCTC-TGAGGATCGGGGCGTGTCGGGCTAGGTCGGGATGTGGGTCGGCGCCAACGAGCCTGGCCTGAG---CACGAG-GAACGGAG-T---GCA-------TTTCGG---------TGCG-CTT-CGGGATCCGAGCT-CGGTCCTGC-GC--CTTGGCCTCCCACGGATCGGCCTTGCTGCGGGG--GTCTGCTG-AC-AGTTGCA-------CAC-AGACTT--GT-CTG---------AGTGTGGACGG-GCAGAATCTTACCCTTCGGCCGCCATTCAACGGTCAACTCAGAACTGGCACGGACCGGGAGAATCCGACTGTCTAATTAAAGCAAAGCATTGCGATGGCTCCAGCTGAGTGTTCACGCAATGTGATTTCTGCCCAGTGCTCTGAATGTCAACGTGAAGAAATTCAAGC-AAGCGCGGGTAAACGGCGGGAGTAACTATGACTCTCTTAAGGTAGCCAAATGCCTCGTCATCTAATTAGTGACGCGCATGAATGGATTAACGAGATTCTCA-CTGTCCCTATCTACTATCTAGCGAAACCACTGCCAAGGGAACGGGCTTGGAAAAATTAGCGGGGAAAGAAGACCCTGTTGAGCTTGACTCTAGTCTGG |
| *Epeurysa nawaii* | CAG-GTGAGGGCCACGCGGAAGTCGCGCCGGATCGTACCCATATCCGCAGCAGGTCTCCAAGGTTAAGAGCCTCTAGTCGATAGAATAATGTAGGTAAGGGAAGTCGGCAAATTGGATC-CGTAACTTCGGAACAAGGATTGGCTC-TGAGGATCGGGGCGTGTCGGGCTAGGTCGGGATGTGGGTCGGCGCCAACGAGCCTGGCCTGAG---CACGAG-GAACGGAG-T---GCA-------TTTCGG---------TGCG-CTT-CGGGATCCGAGCT-CGGTCCTGC-GC--CTTGGCCTCCCACGGATCGGCCTTGCTGCGGGG--GTCTGCTG-AC-AGTTGCA-------CAC-AGACTT--GT-CTG---------AGTGTGGACGG-GCAGAATCTTACCCTTCGGCCGCCATTCAACGGTCAACTCAGAACTGGCACGGACCGGGAGAATCCGACTGTCTAATTAAAGCAAAGCATTGCGATGGCTCCAGCTGAGTGTTCACGCAATGTGATTTCTGCCCAGTGCTCTGAATGTCAACGTGAAGAAATTCAAGC-AAGCGCGGGTAAACGGCGGGAGTAACTATGACTCTCTTAAGGTAGCCAAATGCCTCGTCATCTAATTAGTGACGCGCATGAATGGATTAACGAGATTCTCA-CTGTCCCTATCTACTATCTAGCGAAACCACTGCCAAGGGAACGGGCTTGGAAAAATTAGCGGGGAAAGAAGACCCTGTTGAGCGTGACTCTAGTCTGG |
| *Eurybregma nigrolineata* | CAG-GTGAGGGCCACGCGGAAGTCGCGCCGGATCGTACCCATATCCGCAGCAGGTCTCCAAGGTTAAGAGCCTCTAGTCGATAGAATAATGTAGGTAAGGGAAGTCGGCAAATTGGATC-CGTAACTTCGGGACAAGGATTGGCTC-TGAGGATCGGGGTGTGTCGGGCTAGGTCGGGATGTGGGTCAGCGCCAACGAGCCTGGCCTGAG---CACGAG-GAATGGAG-A---GCA-------CTTCGG---------TGCA-ATC-CAGGATCCGAGCT-CGGTCCTGC-GC--CTTGGCCTCCCACGGATCGGCCTTGCTGCGGGG--GTCTGTTG-AC-AGTTGCA-------CAC-GGACTT--GT-TCG---------TGTGTTAACCA-GCAGAATTCA--CCTTCGGCCACCATTTAACGGTCAACTCAGAACTGGCACGGACCGGGAGAATCCGACTGTCTAATTAAAGCAAAGCATTGCGATGGCTCTAGCTGAGTGTTTACGCAATGTGATTTCTGCCCAGTGCTCTGAATGTCAACGTGAAGAAATTCAAGC-AAGCGCGGGTAAACGGCGGGAGTAACTATGACTCTCTTAAGGTAGCCAAATGCCTCGTCATCTAATTAGTGACGCGCATGAATGGATTAACGAGATTCTCA-CTGTCCCTATCTACTATCTAGCGAAACCACTGCCAAGGGAACGGGCTTGGAAAAATTAGCGGGGAAAGAAGACCCTGTTGAGCTTGACTCTAGTCTGG |
| *Falcotoya lyraeformis* | CAG-GTGAGGGCCACGCGGAAGTCGCGCCAGATCGTACCCATATCCGCAGCAGGTCTCCAAGGTTAAGAGCCTCTAGTCGATAGAATAATGTAGGTAAGGGAAGTCGGCAAATTGGATC-CGTAACTTCGGGACAAGGATTGGCTC-TGAGGATCGGGGTGTGTCGGGCTAGGTCGGGATGTGGGTTAGCGCCAACGAGCCTGGCCTGAG---CACGAG-GAATGGAG-A---GCA-------CTTCGG---------TGCG-ATC-CAGGATCCGAGCT-CGGTCCTGC-GC--CTTGGCCTCCCACGGATCGGCCTTGCTGCGGGG--GTCTGTTG-AC-AGTTGCA-------CAC-AGACTT--GT-CTG---------TGTGTTAACCA-GCAGAATTCA--CCTTCGGCCACCATTTAACGGTCAACTCAGAACTGGCACGGACCGGGAGAATCCGACTGTCTAATTAAAGCAAAGCATTGCGATGGCTCTAGCTGAGTGTTTACGCAATGTGATTTCTGCCCAGTGCTCTGAATGTCAACGTGAAGAAATTCAAGC-AAGCGCGGGTAAACGGCGGGAGTAACTATGACTCTCTTAAGGTAGCCAAATGCCTCGTCATCTAATTAGTGACGCGCATGAATGGATTAACGAGATTCTCA-CTGTCCCTATCTACTATCTAGCGAAACCACTGCCAAGGGAACGGGCTTGGAAAAATTAGCGGGGAAAGAAGACCCTGTTGAGCTTGACTCTAGTCTGG |
| *Fangdelphax gongshanensis* | CAG-GTGAGGGCCACGCGGAAGTCGCGCCAGATCGTACCCATATCCGCAGCAGGTCTCCAAGGTTAAGAGCCTCTAGTCGATAGAATAATGTAGGTAAGGGAAGTCGGCAAATTGGATC-CGTAACTTCGGGACAAGGATTGGCTC-TGAGGATCGGGGTGTGTCGGGCTAGATCGGGATGTGGGTCAGCGCCAACGAGCCTGGCCTGAG---CACGAG-GAATGGAG-A---GCA-------TCTCGG---------TGCG-ATC-CAGGATCCGAGCT-CGGTCCTGC-GC--CTTGGCCTCCCACGGATCGGTCTTGCTGCGGGG--GTCTGTTG-AC-AGTTGCA-------TAT-TGACTT--GT-CAG---------TGTGTCAACCA-GCAGAATTCA--CCTTCGGCCACCATTTAACGGTCAACTCAGAACTGGCACGGACCGGGAGAATCCGACTGTCTAATTAAAGCAAAGCATTGCGATGGCTCTAGCTGAGTGTTTACGCAATGTGATTTCTGCCCAGTGCTCTGAATGTCAACGTGAAGAAATTCAAGC-AAGCGCGGGTAAACGGCGGGAGTAACTATGACTCTCTTAAGGTAGCCAAATGCCTCGTCATCTAATTAGTGACGCGCATGAATGGATTAACGAGATTCTCA-CTGTCCCTATCTACTATCTAGCGAAACCACTGCCAAGGGAACGGGCTTGGAAAAATTAGCGGGGAAAGAAGACCCTGTTGAGCTTGACTCTAGTCTGG |
| *Flavoclypeus nigrifacies* | CAG-GTGAGGGCCACGCGGAAGTCGCGCCAGATCGTACCCATATCCGCAGCAGGTCTCCAAGGTTAAGAGCCTCTAGTCGATAGAATAATGTAGGTAAGGGAAGTCGGCAAATTGGATCGCGTAACTTCGGGACAAGGATTGGCTCGTGAGGATCGGGGTGTGTCGGGCTAGGTCGGGATGTGGGTCAGCGCCAACGAGCCTGGCCTGAG---CACGAG-GAATGGAG-A---GCA-------CTTCGG---------TGCA-ATC-CAGGATCCGAGCT-CGGTCCTGC-GC--CTTGGCCTCCCACGGATCGGCCTTGCTGCGGGG--GTCTGTTG-AC-AGTTGCA-------CAC-GGACTT--GT-CTG---------TGTGTTAACCA-GCAGAATTCA--CCTTCGGCCACCATTTAACGGTCAACTCAGAACTGGCACGGACCGGGAGAATCCGACTGTCTAATTAAAGCAAAGCATTGCGATGGCTCTAGCTGAGTGTTTACGCAATGTGATTTCTGCCCAGTGCTCTGAATGTCAACGTGAAGAAATTCAAGC-AAGCGCGGGTAAACGGCGGGAGTAACTATGACTCTCTTAAGGTAGCCAAATGCCTCGTCATCTAATTAGTGACGCGCATGAATGGATTAACGAGATTCTCA-CTGTCCCTATCTACTATCTAGCGAAACCACTGCCAAGGGAACGGGCTTGGAAAAATTAGCGGGGAAAGAAGACCCTGTTGAGCTTGACTCTAGTCTGG |
| Gen. nov. 1 | CAG-GTGAGGGCCACGCGGAAGTCGCGCCGGATCGTACCCATATCCGCAGCAGGTCTCCAAGGTTAAGAGCCTCTAGTCGATAGAATAATGTAGGTAAGGGAAGTCGGCAAATTGGATC-CGTAACTTCGGAACAAGGATTGGCTC-TGAGGATCGGGGCGTGTCGGGCTAGGTCGGGATGTGGGTCGGCGCCAACGAGCCTGGCCTGAG---CACGAG-GAACGGAG-T---GCA-------TTTCGG---------TGTG-CTT-CGGGATCCGAGCT-CGGGCCTGC-GC--CTTGGCCTCCCACGGATCGGCCTTGCTGCGGGG--GTCTGTTG-AC-AGTTGCG-------TGC-AGACTT--GTGCTG---------TGCGTGGACGG-GCAGAATCTATCCCTTCGGCCGCCATTCAACGGTCAACTCAGAACTGGCACGGACCGGGAGAATCCGACTGTCTAATTAAAGCAAAGCATTGCGATGGCTCTAGCTGAGTGTTTACGCAATGTGATTTCTGCCCAGTGCTCTGAATGTCAACGTGAAGAAATTCAAGC-AAGCGCGGGTAAACGGCGGGAGTAACTATGACTCTCTTAAGGTAGCCAAATGCCTCGTCATCTAATTAGTGACGCGCATGAATGGATTAACGAGATTCTCA-CTGTCCCTATCTACTATCTAGCGAAACCACTGCCAAGGGAACGGGCTTGGAAAAATTAGCGGGGAAAGAAGACCCTGTTGAGCTTGACTCTAGTCTGG |
| *Hadeodelphax afurculus* | CAG-GTGAGGGCCACGCGGAAGTCGCGCCGGATCGTACCCATATCCGCAGCAGGTCTCCAAGGTTAAGAGCCTCTAGTCGATAGAATAATGTAGGTAAGGGAAGTCGGCAAATTGGATC-CGTAACTTCGGGACAAGGATTGGCTC-TGAGGATCGGGGTGTGTCGGGCTAGGTCAGGATGTGGGTCGGCGCCAACGAGCCAGGCCTGAG---CACGAG-GAATGGAGGA---GCA-------TTTCGG---------TGTG-ATT-CAGGATCCGAGCT-CGGTCCTGC-GC--CTTGGCCTCCCACGGATTGGCCTTGCTGCGGGG--GTCTGTTG-AC-AGTTGCA-------CAT-AGACTT--GT-CTG---------TGTGTCAACCA-GCAGAATTCA--CCTTCGGCCACCATTTAACGGTCAACTCAGAACTGGCACGGACCGGGAGAATCCGACTGTCTAATTAAAGCAAAGCATTGCGATGGCTCTAGCTGAGTGTTCACGCAATGTGATTTCTGCCCAGTGCTCTGAATGTCAACGTGAAGAAATTCAAGC-AAGCGCGGGTAAACGGCGGGAGTAACTATGACTCTCTTAAGGTAGCCAAATGCCTCGTCATCTAATTAGTGACGCGCATGAATGGATTAACGAGATTCTCA-CTGTCCCTATCTACTATCTAGCGAAACCACTGCCAAGGGAACGGGCTTGGAAAAATTAGCGGGGAAAGAAGACCCTGTTGAGCTTGACTCTAGTCTGG |
| *Harmalia aculeatus* | CAG-GTGAGGGCCACGCGGAAGTCGCGCCGGATCGTACCCATATCCGCAGCAGGTCTCCAAGGTTAAGAGCCTCTAGTCGATAGAATAATGTAGGTAAGGGAAGTCGGCAAATTGGATC-CGTAACTTCGGGACAAGGATTGGCTC-TGGGGATCGGGGTGTGTCGGGCTAGGTCGGGATGTGGGTCAGCGCCAACGAGCCTGGCCTGAG---CACGAG-GAATGGAG-A---GCA-------CCTCGG---------TGCA-ATC-CAGGATCCGAGCT-CGGTCCTGC-GC--CTTGGCCTCCCACGGATCGGCCTTGCTGCGGGG--GTCTGTTG-AC-AGTTGCA-------CAC-GGACTT--GT-TCG---------TGTGTTAACGA-GCAGAATTCA--CCTTCGGCCACCATTTAACGGTCAACTCAGAACTGGCACGGACCGGGAGAATCCGACTGTCTAATTAAAGCAAAGCATTGCGATGGCTCTAGCTGAGTGTTTACGCAATGTGATTTCTGCCCAGTGCTCTGAATGTCAACGTGAAGAAATTCAAGC-AAGCGCGGGTAAACGGCGGGAGTAACTATGACTCTCTTAAGGTAGCCAAATGCCTCGTCATCTAATTAGTGACGCGCATGAATGGATTAACGAGATTCTCA-CTGTCCCTATCTACTATCTAGCGAAACCACTGCCAAGGGAACGGGCTTGGAAAAATTAGCGGGGAAAGAAGACCCTGTTGAGCTTGACTCTAGTCTGG |
| *Harmalia gayasana* | CAG-GTGAGGGCCACGCGGAAGTCGCGCCGGATCGTACCCATATCCGCAGCAGGTCTCCAAGGTTAAGAGCCTCTAGTCGATAGAATAATGTAGGTAAGGGAAGTCGGCAAATTGGATC-CGTAACTTCGGGACAAGGATTGGCTC-TGAGGATCGGGGTGTGTCGGGCTAGGTCGGGATGTGGGTCAGCGCCAACGAGCCTGGCCTGAG---CACGAG-GAATGGAG-A---GCA-------CTTCGG---------TGCA-ATC-CAGGATCCGAGCT-CGGTCCTGC-GC--CTTGGCCTCCCACGGATCGGCCTTGCTGCGGGG--GTCTGTTG-AC-AGTTGCA-------CAC-GGACTT--GT-TCG---------TGTGTTAACCA-GCAGAATTCA--CCTTCGGCCACCATTTAACGGTCAACTCAGAACTGGCACGGACCGGGAGAATCCGACTGTCTAATTAAAGCAAAGCATTGCGATGGCTCTAGCTGAGTGTTTACGCAATGTGATTTCTGCCCAGTGCTCTGAATGTCAACGTGAAGAAATTCAAGC-AAGCGCGGGTAAACGGCGGGAGTAACTATGACTCTCTTAAGGTAGCCAAATGCCTCGTCATCTAATTAGTGACGCGCATGAATGGATTAACGAGATTCTCA-CTGTCCCTATCTACTATCTAGCGAAACCACTGCCAAGGGAACGGGCTTGGAAAAATTAGCGGGGAAAGAAGACCCTGTTGAGCTTGACTCTAGTCTGG |
| *Harmalia heitensis* | CAG-GTGAGGGCCACGCGGAAGTCGCGCCGGATCGTACCCATATCCGCAGCAGGTCTCCAAGGTTAAGAGCCTCTAGTCGATAGAATAATGTAGGTAAGGGAAGTCGGCAAATTGGATC-CGTAACTTCGGGATAAGGATTGGCTC-TGGGGATCGGGGTGTGTCGGGCTTGGTCGGGATGTGGTTGAGCGATGTGCGATCTGGCCTGAG---CGAGAG-GAATGGAG-A-------------------------------G-ATC-CAGGATCCGAGTT-CGGTC-TGC-GC--CTTGGCC-CCCACGGATCGGCCTTGCTGCGGGG--GTCTGTTG-AC-TGTTGCA-------CAC-GGACTT--GT-TCG---------TGTGTTAACCA-GCAGAATTCA--CCTTCGGCCACCATTTAACGGTCAACTCAGAACTGGCACGGACTGGGAGAATCCGACTGTCTAATTAAAGCAAAGCATTGCGATGGCTCTAACTGAGTGTTTACGCAATGTGATTTCTGCCCAGTGCTCTGAATGTCAACGTGAAGAAATTCAAGC-AAGCGCGGGTAAACGGCGGGAGTAACTATGACTCTCTTAAGGTAGCCAAATGCCTCGTCATCTAATTAGTGACGCGCATGAATGGATTAACGAGATTCTCA-CTGTCCCTATCTACTATCTAGCGAAACCACTGCCAAGGGAACGGGCTTGGAAAAATTAGCGGGGAAAGAAGACCCTGTTGAGCTTGACTCTAGTCTGG |
| *Harmalia ostorius* | CAG-GTGAGGGCCACGCGGAAGTCGCGCCGGATCGTACCCATATCCGCAGCAGGTCTCCAAGGTTAAGAGCCTCTAGTCGATAGAATAATGTAGGTAAGGGAAGTCGGCAAATTGGATC-CGTAACTTCGGGACAAGGATTGGCTC-TGAGGATCGGGGTGTGTCGGGCTAGGTCGGGATGTGGGTCAGCGCCAACGAGCCTGGCCTGAG---CACGAG-GAATGGAG-A---GCA-------CTTCGG---------TGCG-ATC-CAGGATCCGAGCT-CGGTCCTGC-GC--CTTGGCCTCCCACGGATCGGCCTTGCTGCGGGG--GTCTGTTG-AC-AGTTGCA-------CAC-GGACTT--GT-TCG---------TGTGTTAACCA-GCAGAATTCA--CCTTCGGCCACCATTTAACGGTCAACTCAGAACTGGCACGGACCGGGAGAATCCGACTGTCTAATTAAAGCAAAGCATTGCGATGGCTCTAGCTGAGTGTTTACGCAATGTGATTTCTGCCCAGTGCTCTGAATGTCAACGTGAAGAAATTCAAGC-AAGCGCGGGTAAACGGCGGGAGTAACTATGACTCTCTTAAGGTAGCCAAATGCCTCGTCATCTAATTAGTGACGCGCATGAATGGATTAACGAGATTCTCA-CTGTCCCTATCTACTATCTAGCGAAACCACTGCCAAGGGAACGGGCTTGGAAAAATTAGCGGGGAAAGAAGACCCTGTTGAGCTTGACTCTAGTCTGG |
| *Harmalia sirokata* | CAG-GTGAGGGCCACGCGGAAGTCGCGCCGGATCGTACCCATATCCGCAGCAGGTCTCCAAGGTTAAGAGCCTCTAGTCGATAGAATAATGTAGGTAAGGGAAGTCGGCAAATTGGATC-CGTAACTTCGGGACAAGGATTGGCTC-TGAGGATCGGGGTGTGTCGGGCTAGGTCGGGATGTGGGTCAGCGCCAACGAGCCTGGCCTGAG---CACGAG-GAATGGAG-A---GCA-------CTTCGG---------TGCA-ATC-CAGGATCCGAGCT-CGGTCCTGC-GC--CTTGGCCTCCCACGGATCGGCCTTGCTGCGGGG--GTCTGTTG-AC-AGTTGCA-------CAC-GGACTT--GT-TCG---------TGTGTTAACCA-GCAGAATTCA--CCTTCGGCCACCATTTAACGGTCAACTCAGAACTGGCACGGACCGGGAGAATCCGACTGTCTAATTAAAGCAAAGCATTGCGATGGCTCTAGCTGAGTGTTTACGCAATGTGATTTCTGCCCAGTGCTCTGAATGTCAACGTGAAGAAATTCAAGC-AAGCGCGGGTAAACGGCGGGAGTAACTATGACTCTCTTAAGGTAGCCAAATGCCTCGTCATCTAATTAGTGACGCGCATGAATGGATTAACGAGATTCTCA-CTGTCCCTATCTACTATCTAGCGAAACCACTGCCAAGGGAACGGGCTTGGAAAAATTAGCGGGGAAAGAAGACCCTGTTGAGCTTGACTCTAGTCTGG |
| *Harmalia tiphys* | CAG-GTGAGGGCCACGCGGAAGTCGCGCCGGATCGTACCCATATCCGCAGCAGGTCTCCAAGGTTAAGAGCCTCTAGTCGATAGAATAATGTAGGTAAGGGAAGTCGGCAAATTGGATC-CGTAACTTCGGGACAAGGATTGGCTC-TGAGGATCGGGGTGTGTCGGGCTAGGTCGGGATGTGGGTCAGCGCCAACGAGCCTGGCCTGAG---CACGAG-GAATGGAG-A---GTG-------CTTCGG---------TGCA-ATC-CAGGATCCGAGCT-CGGTCCTGC-GC--CTTGGCCTCCCACGGATCGGCCTTGCTGCGGGG--GTCTGTTG--C-AGTTGCA-------CAT-GGACTT--GT-TCG---------TGTGTTAACCA-GCAGAATTCA--CCTTCGGCCACCATTTAACGGTCAACTCAGAACTGGCACGGACCGGGAGAATCCGACTGTCTAATTAAAGCAAAGCATTGCGATGGCTCTAGCTGAGTGTTTACGCAATGTGATTTCTGCCCAGTGCTCTGAATGTCAACGTGAAGAAATTCAAGC-AAGCGCGGGTAAACGGCGGGAGTAACTATGACTCTCTTAAGGTAGCCAAATGCCTCGTCATCTAATTAGTGACGCGCATGAATGGATTAACGAGATTCTCA-CTGTCCCTATCTACTATCTAGCGAAACCACTGCCAAGGGAACGGGCTTGGAAAAATTAGCGGGGAAAGAAGACCCTGTTGAGCTTGACTCTAGTCTGG |
| *Himeunka tateyamaella* | CAG-GTGAGGGCCACGCGGAAGTCGCGCCAGATCGTACCCATATCCGCAGCAGGTCTCCAAGGTTAAGAGCCTCTAGTCGATAGAATAATGTAGGTAAGGGAAGTCGGCAAATTGGATC-CGTAACTTCGGGACAAGGATTGGCTC-TGAGGATCGGGGTGTGTCGGGCTAGGTCGGGATGTGGGTTAGCGCCAACGAGCCTGGCCTGAG---CACGAG-GAATGGAA-A---GCA-------TCTCGG---------TGCA-ATC-CAGGATCCGAGCT-CGGTCCTGC-GC--CTTGGCCTCCCACGGATCGGCCTTGCTGCGGGG--GTCTGTTG-AC-GGCTGCA-------TAC-AGACTT--GT-CTG---------TGTGCAGACAAGGCAGAATTCA--CCTTCGGCCACCATTTAACGGTCAACTCAGAACTGGCACGGACCGGGAGAATCCGACTGTCTAATTAAAGCAAAGCATTGCGATGGCTCTAGCTGAGTGTTTACGCAATGTGATTTCTGCCCAGTGCTCTGAATGTCAACGTGAAGAAATTCAAGC-AAGCGCGGGTAAACGGCGGGAGTAACTATGACTCTCTTAAGGTAGCCAAATGCCTCGTCATCTAATTAGTGACGCGCATGAATGGATTAACGAGATTCTCA-CTGTCCCTATCTACTATCTAGCGAAACCACTGCCAAGGGAACGGGCTTGGAAAAATTAGCGGGGAAAGAAGACCCTGTTGAGCTTGACTCTAGTCTGG |
| *Ishiharodelphax matsuyamensis* | CAG-GTGAGGGCCACGCGGAAGTCGCGCCAGATCGTACCCATATCCGCAGCAGGTCTCCAAGGTTAAGAGCCTCTAGTCGATAGAATAATGTAGGTAAGGGAAGTCGGCAAATTGGATC-CGTAACTTCGGGACAAGGATTGGCTC-TGAGGATCGGGGTGTGTCGGGCTAGGTCGGGATGTGGGTCAGCGCCAACGAGCCTGGCCTGAG---CACGAG-GAATGGAG-A---GCA-------TTTCGG---------TGCA-ATC-CAGGATCCGAGCT-CGGTCCTGC-GC--CTTGGCCTCCCACGGATCGGCCTTGCTGCGGGG--GTCTGTTG-AC-AGTTGCA-------CAC-AGACTT--GT-CTG---------TGTGTTAACCA-GCAGAATTCA--CCTTCGGCCACCATTTAACGGTCAACTCAGAACTGGCACGGACCGGGAGAATCCGACTGTCTAATTAAAGCAAAGCATTGCGATGGCTCTAGCTGAGTGTTTACGCAATGTGATTTCTGCCCAGTGCTCTGAATGTCAACGTGAAGAAATTCAAGC-AAGCGCGGGTAAACGGCGGGAGTAACTATGACTCTCTTAAGGTAGCCAAATGCCTCGTCATCTAATTAGTGACGCGCATGAATGGATTAACGAGATTCTCA-CTGTCCCTATCTACTATCTAGCGAAACCACTGCCAAGGGAACGGGCTTGGAAAAATTAGCGGGGAAAGAAGACCCTGTTGAGCTTGACTCTAGTCTGG |
| *Isodelphax basivitta* | ----GTGAGGGCCACGCGGAAGTCGCGCCGGATCGTACCCATATCCGCAGCAGGTCTCCAAGGTTAAGAGCCTCTAGTCGATAGAATAATGTAGGTAAGGGAAGTCGGCAAATTGGATC-CGTAACTTCGGAACAAGGATTGGCTC-TGAGGATTGGGGTGTGTCGGGCTAGGTCGGGATGTGGTTCAGTGCCAACGAGCCTGGCCTGAG---CACGAG-GAATGGAG-A---GCA-------TTTCGG---------TGCT-ATT-CAGGATCCGAGCT-CGGTCTTGC-GC--CTTGGCCTCCCACGGATCGGCCTTGCTGCGGGG--GTCTGTTG-AC-AGTTGCA-------CAC--AACTACGGA--TG---------TGTGTTAACCA-GCAGAATTCA--CCTTCGGCCACCATTTAACGGTCAACTCAGAACTGGCACGGACCGGGAGAATCCGACTGTCTAATTAAAGCAAAGCATTGCGATGGCTCTAGCTGAGTGTTTACGCAATGTGATTTCTGCCCAGTGCTCTGAATGTCAACGTGAAGAAATTCAAGC-AAGCGCGGGTAAACGGCGGGAGTAACTATGACTCTCTTAAGGTAGCCAAATGCCTCGTCATCTAATTAGTGACGCGCATGAATGGATTAACGAGATTCTCA-CTGTCCCTATCTACTATCTAGCGAAACCACTGCCAAGGGAACGGGCTTGGAAAAATTAGCGGGGAAAGAAGACCCTGTTGAGCTTGACTCTAGTCTGG |
| *Javesella dubia* | CAG-GTGAGGGCCACGCGGAAGTCGCGCCGGATCGTACCCATATCCGCAGCAGGTCTCCAAGGTTAAGAGCCTCTAGTCGATAGAATAATGTAGGTAAGGGAAGTCGGCAAATTGGATC-CGTAACTTCGGGACAAGGATTGGCTC-TGAGGATCGGGGTGTGTCGGGCTAGGTTGGGATGTGGGTCAGCGCCAACGAGCCTGGCCTGAG---CACGAG-GAATGGAG-A---GTG-------TTTCGA---------CGCT-TTC-CAGGATCCGAGCT-CGGTCCTGC-GC--CTTGGCCTCCCACGGATCGGCCTTGCTGCGGGG--GTCTGTTG-AC-AGTTGCA-------CAT-AAACTT--GT-TTG---------TGTGTCAACCA-GCAGAATTCA--CCTTCGGCCACCATTTAACGGTCAACTCAGAACTGGCACGGACCGGGAGAATCCGACTGTCTAATTAAAGCAAAGCATTGCGATGGCTCTAGCTGAGTGTTTACGCAATGTGATTTCTGCCCAGTGCTCTGAATGTCAACGTGAAGAAATTCAAGC-AAGCGCGGGTAAACGGCGGGAGTAACTATGACTCTCTTAAGGTAGCCAAATGCCTCGTCATCTAATTAGTGACGCGCATGAATGGATTAACGAGATTCTCA-CTGTCCCTATCTACTATCTAGCGAAACCACTGCCAAGGGAACGGGCTTGGAAAAATTAGCGGGGAAAGAAGACCCTGTTGAGCTTGACTCTAGTCTGG |
| *Javesella obscurella* | CAG-GTAAGGGCCACGCGGAAGTCGCGCCGGATCGTACCCATATCCGCAGCAGGTCTCCAAGGTTAAGAGCCTCTAGTCGATAGAATAATGTAGGTAAGGGAAGTCGGCAAATTGGATC-CGTAACTTCGGGACAAGGATTGGCTC-TGGGGATCGAGGTGTGTCGGGCTTGGTTGGGATGTGGGTCAGCGCCAACGAGCCTGGCCTGAG---CACGGT-GAATGGAG------CA-------CTTCGG---------TGAC-GTT-CAGGATCCGAGTT-CGGTC-TGC-GC--CTTGGCCTCCCACGGATCGGCCTTGCTACGGTGTTGTCTGTTG-ACTGTTTGCT-------CCC-CTATTT--AT--AG---------GGTGTGAACCA-GCAGAGTTCCG-CCTTCGGCCACCATTTAACGGTCAACTCAGAACTGGCACGGACTGGGAGAATCCGACTGTCTAATTAAAGCAAAGCATTGCGATGGCTCCAACTGAGTGTTTACGCAATGTGATTTCTGCCCAGTGCTCTGAATGTCAACGTGAAGAAATTCAAGC-AAGCGCGGGTAAACGGCGGGAGTAACTATGACTCTCTTAAGGTAGCCAAATGCCTCGTCATCTAATTAGTGACGCGCATGAATGGATTAACGAGATTCTCA-CTGTCCCTATCTACTATCTAGCGAAACCACAGCCAAGGGAACGGGCTTGGAAAAATTAGCGGGGAAAGAAGACCCTGTTGAGCTTGACTCTAGTCTGG |
| *Javesella pellucida* | --------------------------------------------------------------------------------------------------------------------------------------------------------------------------------------------------------------------------------------------------------------------------------------------------------------------------------------------------------------------------------------------------------------------------------------------------------------------------------------------------------------------------------------------------------------------------------------------------------------------------------------------------TAATTAGTGACGCGCATGAATGGATTAACGAGATTCTCA-CTGTCCCTATCTACTATCTAGCGAAACCACAGCCAAGGGAACGGGCTTGGAAAAATTAGCGGGGAAAGAAGACCCTGTTGAGCTTGACTCTAGTTTGG |
| *Javesella* sp*.* | CAG-GTGAGGGCCACGCGGAAGTCGCGCCGGATCGTACCCATATCCGCAGCAGGTCTCCAAGGTTAAGAGCCTCTAGTCGATAGAATAATGTAGGTAAGGGAAGTCGGCAAATTGGATC-CGTAACTTCGGGACAAGGATTGGCTC-TGAGGATCGGGGTGTGTCGGGCTAGGTCGGGATGTGGGTCAAAGCCGATGAGCCTGGCCTGAG---CACGAG-GAATGGAG-A---GCA-------TTTCGG---------TGCG-ATC-CAGGATCCGAGCT-CGGTCCTGTAGC--CTTGGCCTCCCACGGATCGGCCTTGCTGCGGGG--GTCTGTTG-AC-AGTTGCA-------CAC-AGACTT--GT-CTG---------TGTGTTAACCA-GCAGAATTCA--CCTTCGGCCACCATTTAACGGTCAACTCAGAACTGGCACGGACCGGGAGAATCCGACTGTCTAATTAAAGCAAAGCATTGCGATGGCTCTAGCTGAGTGTTCACGCAATGTGATTTCTGCCCAGTGCTCTGAATGTCAACGTGAAGAAATTCAAGC-AAGCGCGGGTAAACGGCGGGAGTAACTATGACTCTCTTAAGGTAGCCAAATGCCTCGTCATCTAATTAGTGACGCGCATGAATGGATTAACGAGATTCTCA-CTGTCCCTATCTACTATCTAGCGAAACCACTGCCAAGGGAACGGGCTTGGAAAAATTAGCGGGGAAAGAAGACCCTGTTGAGCTTGACTCTAGTCTGG |
| *Kelisia xiphura* | CGG-GTGAGGGCCACGCGGAAGTCGCGCCGGATCGTACCCATATCCGCAGCAGGTCTCCAAGGTTAAGAGCCTCTAGTCGATAGAATAATGTAGGTAAGGGAAGTCGGCAAATTGGATC-CGTAACTTCGGGACAAGGATTGGCTC-TGAGGATCGGGGCGTGTCGGGCTAGGTTGGGATGTGGGTCAGCGCCAACGAGCCAGGCCTGGA---CGAGGG-GAACGGAT-G---TCA------CTTTCGG--------GTGAG-ATC-CGGGATCCGAGCT-CGGTCCTGT-GC--CTTGGCCTCCCACGGATCGGCCTTGCTGCGGGG-TGTCTGTCG-AC-AGTTGCA-------CAC-AGACTT--GCGCTG---------AGTGTGGACGG-GCAGAATTTATCCCTTCGGCCGCCATTCAACGGTCAACTCAGAACTGGCACGGACCGGGAGAATCCGACTGTCTAATTAAAGCAAAGCATTGCGATGGCTCTAGCTGAGTGTTTACGCAATGTGATTTCTGCCCAGTGCTCTGAATGTCAACGTGAAGAAATTCAAGC-AAGCGCGGGTAAACGGCGGGAGTAACTATGACTCTCTTAAGGTAGCCAAATGCCTCGTCATCTAATTAGTGACGCGCATGAATGGATTAACGAGATTCTCA-CTGTCCCTATCTACTATCTAGCGAAACCACTGCCAAGGGAACGGGCTTGGAAAAATTAGCGGGGAAAGAAGACCCTGTTGAGCTTGACTCTAGTCTGG |
| *Kusnezoviella dimidiatifrons* | CAG-GTGAGGGCCACGCGGAAGTCGCGCCGGATCGTACCCATATCCGCAGCAGGTCTCCAAGGTTAAGAGCCTCTAGTCGATAGAATAATGTAGGTAAGGGAAGTCGGCAAATTGGATC-CGTAACTTCGGGACAAGGATTGGCTC-TGAGGATCGGGGTGTGTCGGGCTAGGTCGGGATGTGGGTCAGCGCCAACGAGCCTGGCCTGAG---CACGAG-GAATGGAA-A---GCA-------CTTCGG---------TGCG-ATC-CAGGATCCGAGCT-CGGTCCTGC-GC--CTTGGCCTCCCACGGATCGGCCTTGCTGCGGGG--GTCTGTTG--C-AGTTGCA-------CAT-GGACTT--GT-CTG---------TGTGTTAACCA-GCAGAATTCA--CCTTCGGCCACCATTTAACGGTCAACTCAGAACTGGCACGGACCGGGAGAATCCGACTGTCTAATTAAAGCAAAGCATTGCGATGGCTCTAGCTGAGTGTTTACGCAATGTGATTTCTGCCCAGTGCTCTGAATGTCAACGTGAAGAAATTCAAGC-AAGCGCGGGTAAACGGCGGGAGTAACTATGACTCTCTTAAGGTAGCCAAATGCCTCGTCATCTAATTAGTGACGCGCATGAATGGATTAACGAGATTCTCA-CTGTCCCTATCTACTATCTAGCGAAACCACTGCCAAGGGAACGGGCTTGGAAAAATTAGCGGGGAAAGAAGACCCTGTTGAGCTTGACTCTAGTCTGG |
| *Laodelphax striatellus* | CAG-GTGAGGGCCACGCGGAAGTCGCGCCGGATCGTACCCATATCCGCAGCAGGTCTCCAAGGTTAAGAGCCTCTAGTCGATAGAATAATGTAGGTAAGGGAAGTCGGCAAATTGGATC-CGTAACTTCGGGACAAGGATTGGCTC-TGAGGATCGGGGTGTGTCGGGCTAGGTCGGGATGTGGGTCAGCGCCAACGAGCCTGGCCTGAG---CACGAG-GAATGGAG-A---GCA-------TCTCGG---------TGCG-ATC-CAGGATCCGAGCT-CGGTCCTGC-GC--CTTGGCCTCCCACGGATCGGCCTTGCTGCGGGG--GTCTGTTG-AC-AGTTGCA-------TAT-TGACTT--GT-CAG---------TGTGTCAACCA-GCAGAATTCA--CCTTCGGCCACCATTTAACGGTCAACTCAGAACTGGCACGGACCGGGAGAATCCGACTGTCTAATTAAAGCAAAGCATTGCGATGGCTCTAGCTGAGTGTTTACGCAATGTGATTTCTGCCCAGTGCTCTGAATGTCAACGTGAAGAAATTCAAGC-AAGCGCGGGTAAACGGCGGGAGTAACTATGACTCTCTTAAGGTAGCCAAATGCCTCGTCATCTAATTAGTGACGCGCATGAATGGATTAACGAGATTCTCA-CTGTCCCTATCTACTATCTAGCGAAACCACTGCCAAGGGAACGGGCTTGGAAAAATTAGCGGGGAAAGAAGACCCTGTTGAGCTTGACTCTAGTCTGG |
| *Latistria placitus* | CAG-GTGAGGGCTACGCGGAAATCGCGCCAGATCGTACCCATATCCGCAGCAGGTCTCCAAGGTTAAGAGCCTCTAGTCGATAGAATAATGTAGGTAAGGGAAGTCGGCAAATTGGATC-CGTAACTTCGGAACAAGGATTGGCTC-TGAGGATTGGGGTGTGTCGGGCTTGGTCGGGATGTGGGTTAGTGCCAACAAGCCTGGCCTGAG---CGAGAG-GAATGGAG-A---GCA-------TTTCGG---------TGTA-ATC-CAGGAACCGAGTT-CGGTCTTGC-GC--CTTGGCCTACCACGGATCGGCCTTGCAGCGGGG--GTCTGTTG-AC-AGTTGCA-------CAC---------AC-------------AGTGTAAACGA-GCAGAATTCA--CCTTCGGCCACCATTTAACGGTCAACTCAGAACTGGCACGGACCAGGAGAATCCGACTGTCTAATTAAAGCAAAGCATTGCGATGGCTCCAGCTGAGTGTTGGCGCAATGTGATTTCTGCCCAGTGCTCTGAATGTCAACGTGAAGAAATTCAAGC-AAGCGCGGGTAAACGGCGGGAGTAACTATGACTCTCTTAAGGTAGCCAAATGCCTCGTCATCTAATTAGTGACGCGCATGAATGGATTAACGAGATTCTCA-CTGTCCCTATCTACTATCTAGCGAAACCACAGCCAAGGGAACGGGCTTGGAAAAATTAGCGGGGAAAGAAGACCCTGTTGAGCTTGACTCTAGTCTGG |
| *Lauriana senticosa* | CAG-GTGAGGGCCACGCGGAAGTCGCGCCGGATCGTACCCATATCCGCAGCAGGTCTCCAAGGTTAAGAGCCTCTAGTCGATAGAATAATGTAGGTAAGGGAAGTCGGCAAATTGGATC-CGTAACTTCGGAACAAGGATTGGCTC-TGAGGATCGGGGCGTGTCGGGCTAGGTCGGGATGTGGGTCGGCGCCAACGAGCCTGGCCTGAG---CACGAG-GAACGGAG-T---GCA-------TTTCGG---------TGCG-CTT-CGGGATCCGAGCT-CGGTCCTGC-GC--CTTGGCCTCCCACGGATCGGCCTTGCTGCGGGG--GTCTGCTG-AC-AGTTGCA-------CAC-AGACTT--GTGCTG---------AGTGTGGACGG-GCAGAATCTATCCCTTCGGCCGCCATTCAACGGTCAACTCAGAACTGGCACGGACCGGGAGAATCCGACTGTCTAATTAAAGCAAAGCATTGCGATGGCTCTAGCTGAGTGTTTACGCAATGTGATTTCTGCCCAGTGCTCTGAATGTCAACGTGAAGAAATTCAAGC-AAGCGCGGGTAAACGGCGGGAGTAACTATGACTCTCTTAAGGTAGCCAAATGCCTCGTCATCTAATTAGTGACGCGCATGAATGGATTAACGAGATTCTCA-CTGTCCCTATCTACTATCTAGCGAAACCACTGCCAAGGGAACGGGCTTGGAAAAATTAGCGGGGAAAGAAGACCCTGTTGAGCTTGACTCTAGTCTGG |
| *Liburniella ornata* | CAG-GTGAGGGCCACGCGGAAGTCGCGCCGGATCGTACCCATATCCGCAGCAGGTCTCCAAGGTTAAGAGCCTCTAGTCGATAGAATAATGTAGGTAAGGGAAGTCGGCAAATTGGATC-CGTAACTTCGGGACAAGGATTGGCTC-TGAGGATCGGGGTGTGTCGGGCTAGGTCAGGATGTGGGTCAGCGCCAACGAGCCAGGCCTGAG---CACGAG-GAATGGAG-A---GCA-------TTTCGG---------TGCA-ATC-CAGGATCCGAGCT-CGGTCCTGC-GC--CTTGGCCTCCCACGGATTGGCCTTGCTGCGGGG--GTCTGTTG-AC-TGTTGCG-------CAT-GGACTT--GT-CTG---------TGCGTTAACCA-GCAGAATTCA--CCTTCGGCCACCATTTAACGGTCAACTCAGAACTGGCACGGACCGGGAGAATCCGACTGTCTAATTAAAGCAAAGCATTGCGATGGCTCTAGCTGAGTGTTCACGCAATGTGATTTCTGCCCAGTGCTCTGAATGTCAACGTGAAGAAATTCAAGC-AAGCGCGGGTAAACGGCGGGAGTAACTATGACTCTCTTAAGGTAGCCAAATGCCTCGTCATCTAATTAGTGACGCGCATGAATGGATTAACGAGATTCTCA-CTGTCCCTATCTACTATCTAGCGAAACCACTGCCAAGGGAACGGGCTTGGAAAAATTAGCGGGGAAAGAAGACCCTGTTGAGCTTGACTCTAGTCTGG |
| *Malaxa semifusca* | CAG-GTGAGGGCCACGCGGAAGTCGCGCCGGATCGTACCCATATCCGCAGCAGGTCTCCAAGGTTAAGAGCCTCTAGTCGATAGAATAATGTAGGTAAGGGAAGTCGGCAAATTGGATC-CGTAACTTCGGAACAAGGATTGGCTC-TGAGGATCGGGGCGTGTCGGGCTAGGTCGGGATGTGGGTCGGCGCCAACGAGCCTGGCCTGAG---CACGAG-GAACGGAG-C---GCA-------TTTCGG---------TGTG-CTT-CGGGATCCGAGCT-CGGTCCTGC-GC--CTTGGCCTCCCACGGATCGGCCTTGCTGCGGGG--GTCTGCTG-AC-AGTTGCA-------TCC-AGACTT--GTGCTG---------GGTGTGGACGG-GCAGAATCTATCCCTTCGGCCGCCATTCAACGGTCAACTCAGAACTGGCACGGACCGGGAGAATCCGACTGTCTAATTAAAGCAAAGCATTGCGATGGCTCTAGCTGAGTGTTTACGCAATGTGATTTCTGCCCAGTGCTCTGAATGTCAACGTGAAGAAATTCAAGC-AAGCGCGGGTAAACGGCGGGAGTAACTATGACTCTCTTAAGGTAGCCAAATGCCTCGTCATCTAATTAGTGACGCGCATGAATGGATTAACGAGATTCTCA-CTGTCCCTATCTACTATCTAGCGAAACCACTGCCAAGGGAACGGGCTTGGAAAAATTAGCGGGGAAAGAAGACCCTGTTGAGCTTGACTCTAGTCTGG |
| *Malaxella macracantha* | CAG-GTGAGGGCCACGCGGAAGTCGCGCCGGATCGTACCCATATCCGCAGCAGGTCTCCAAGGTTAAGAGCCTCTAGTCGATAGAATAATGTAGGTAAGGGAAGTCGGCAAATTGGATC-CGTAACTTCGGAACAAGGATTGGCTC-TGAGGATCGGGGCGTGTCGGGCTAGGTCGGGATGTGGGTCGGCGCCAACGAGCCTGGCCTGAG---CACGAG-GAACGGAG-T---GCA-------TTTCGG---------TGTG-CTT-CGGGATCCGAGCT-CGGTCCTGC-GC--CTTGGCCTCCCACGGATCGGCCTTGCTGCGGGG--GTCTGTTG-AC-AGTTGCA-------CAC-AGACTT--GTGCTG---------TGTGTGGACGG-GCAGAATCTATCCCTTCGGCCGCCATTCAACGGTCAACTCAGAACTGGCACGGACCGGGAGAATCCGACTGTCTAATTAAAGCAAAGCATTGCGATGGCTCTAGCTGAGTGTTTACGCAATGTGATTTCTGCCCAGTGCTCTGAATGTCAACGTGAAGAAATTCAAGC-AAGCGCGGGTAAACGGCGGGAGTAACTATGACTCTCTTAAGGTAGCCAAATGCCTCGTCATCTAATTAGTGACGCGCATGAATGGATTAACGAGATTCTCA-CTGTCCCTATCTACTATCTAGCGAAACCACTGCCAAGGGAACGGGCTTGGAAAAATTAGCGGGGAAAGAAGACCCTGTTGAGCTTGACTCTAGTCTGG |
| *Malaxella tetracantha* | CAG-GTGAGGGCCACGCGGAAGTCGCGCCGGATCGTACCCATATCCGCAGCAGGTCTCCAAGGTTAAGAGCCTCTAGTCGATAGAATAATGTAGGTAAGGGAAGTCGGCAAATTGGATC-CGTAACTTCGGAACAAGGATTGGCTC-TGAGGATCGGGGCGTGTCGGGCTAGGTCGGGATGTGGGTCGGCGCCAACGAGCCTGGCCTGAG---CACGAG-GAACGGAG-T---GCA-------TTTTGG---------TGTG-CTT-CGGGATCCGAGCT-CGGTCCTGC-GC--CTTGGCCTCCCACGGATCGGCCTTGCTGCGGGG--GTCTGTTG-AC-AGTTGCA-------CAT-AGACTT--GTGCTG---------TGTGTGGACGG-GCAGAATCTATCCCTTCGGCCGCCATTCAACGGTCAACTCAGAACTGGCACGGACCGGGAGAATCCGACTGTCTAATTAAAGCAAAGCATTGCGATGGCTCTAGCTGAGTGTTTACGCAATGTGATTTCTGCCCAGTGCTCTGAATGTCAACGTGAAGAAATTCAAGC-AAGCGCGGGTAAACGGCGGGAGTAACTATGACTCTCTTAAGGTAGCCAAATGCCTCGTCATCTAATTAGTGACGCGCATGAATGGATTAACGAGATTCTCA-CTGTCCCTATCTACTATCTAGCGAAACCACTGCCAAGGGAACGGGCTTGGAAAAATTAGCGGGGAAAGAAGACCCTGTTGAGCTTGACTCTAGTCTGG |
| *Megadephax kangauzi* | CAG-GTGAGGGCCACGCGGAAGTCGCGCCGGATCGTACCCATATCCGCAGCAGGTCTCCAAGGTTAAGAGCCTCTAGTCGATAGAATAATGTAGGTAAGGGAAGTCGGCAAATTGGATC-CGTAACTTCGGGACAAGGATTGGCTC-TGAGGATCGGGGTGTGTCGGGCTAGGTCGGGATGTGGGTCAGCGCCAACGAGCCTGGCCTGAG---CACGAG-GAATGGAG-A---GCA-------TTTCGG---------TGCA-ATC-CAGGATCCGAGCT-CGGTCCTGC-GC--CTTGGCCTCCCACGGATCGGCCTTGCTGCGGGG--GTCTGTTG-AC-AGTTGCA-------CAT-TGACTT--GT-CAG---------TGTGTCAACCA-GCAGAATTCA--CCTTCGGCCACCATTTAACGGTCAACTCAGAACTGGCACGGACCGGGAGAATCCGACTGTCTAATTAAAGCAAAGCATTGCGATGGCTCTAGCTGAGTGTTTACGCAATGTGATTTCTGCCCAGTGCTCTGAATGTCAACGTGAAGAAATTCAAGC-AAGCGCGGGTAAACGGCGGGAGTAACTATGACTCTCTTAAGGTAGCCAAATGCCTCGTCATCTAATTAGTGACGCGCATGAATGGATTAACGAGATTCTCA-CTGTCCCTATCTACTATCTAGCGAAACCACTGCCAAGGGAACGGGCTTGGAAAAATTAGCGGGGAAAGAAGACCCTGTTGAGCTTGACTCTAGTCTGG |
| *Mestus cruciatus* | CAG-GTGAGGGCCACGCGGAAGTCGCGCCAGACCGTACCCATATCCGCAGCAGGTCTCCAAGGTTAAGAGCCTCTAGTCGATAGAATAATGTAGGTAAGGGAAGTCGGCAAATTGGATC-CGTAACTTCGGGACAAGGATTGGCTC-TGAGGATCGGGGTGTGTCGGGCTAGGTTGGGATGTGGGTCAGCGCCAACGAGCCTGGCCTGAG---CACGAG-GAATGGAG-A---GCA-------TTTCGG---------TGTA-ATC-CAGGATCCGAGCT-CGGTCCTGC-GC--CTTGGCCTCCCACGGATCGGCCTTGCTGCGGGG--GTCTGTTG-AC-AGTTGCA-------CAC-AGACTT--GT-CTG---------TGTGTTAACCA-GCAGAATTCA--CCTTCGGCCACCATTTAACGGTCAACTCAGAACTGGCACGGACTGGGAGAATCCGACTGTCTAATTAAAGCAAAGCATTGCGATGGCTCTTGCTGAGTGTTTACGCAATGTGATTTCTGCCCAGTGCTCTGAATGTCAACGTGAAGAAATTCAAGC-AAGCGCGGGTAAACGGCGGGAGTAACTATGACTCTCTTAAGGTAGCCAAATGCCTCGTCATCTAATTAGTGACGCGCATGAATGGATTAACGAGATTCTCA-CTGTCCCTATCTACTATCTAGCGAAACCACTGCCAAGGGAACGGGCTTGGAAAAATTAGCGGGGAAAGAAGACCCTGTTGAGCTTGACTCTAGTCTGG |
| *Metadelphax propinqua* | CAG-GTGAGGGCCACGCGGAAGTCGCGCCAGATCGTACCCATATCCGCAGCAGGTCTCCAAGGTTAAGAGCCTCTAGTCGATAGAATAATGTAGGTAAGGGAAGTCGGCAAATTGGATC-CGTAACTTCGGGACAAGGATTGGCTC-TGAGGATCGGGGTGTGTCGGGCTAGGTCGGGATGTGGGTCAGCGCCAACGAGCCTGGCCTGAG---CACGAG-GAATGGAG-A---GCA-------TTTCGG---------TGCA-ATC-CAGGATCCGAGCT-CGGTCCTGC-GC--CTTGGCCTCCCACGGATCGGCCTTGCTGCGGGG--GTCTGTTG-AC-AGTTGCA-------CAC-GGACTT--GT-CTG---------TGTGTTAACCA-GCAGAATTCA--CCTTCGGCCACCATTTAACGGTCAACTCAGAACTGGCACGGACCGGGAGAATCCGACTGTCTAATTAAAGCAAAGCATTGCGATGGCTCTAGCTGAGTGTTTACGCAATGTGATTTCTGCCCAGTGCTCTGAATGTCAACGTGAAGAAATTCAAGC-AAGCGCGGGTAAACGGCGGGAGTAACTATGACTCTCTTAAGGTAGCCAAATGCCTCGTCATCTAATTAGTGACGCGCATGAATGGATTAACGAGATTCTCA-CTGTCCCTATCTACTATCTAGCGAAACCACTGCCAAGGGAACGGGCTTGGAAAAATTAGCGGGGAAAGAAGACCCTGTTGAGCTTGACTCTAGTCTGG |
| *Metadelphax propinqua* | CAG-GTGAGGGCCACGCGGAAGTCGCGCCAGATCGTACCCATATCCGCAGCAGGTCTCCAAGGTTAAGAGCCTCTAGTCGATAGAATAATGTAGGTAAGGGAAGTCGGCAAATTGGATC-CGTAACTTCGGGACAAGGATTGGCTC-TGAGGATCGGGGTGTGTCGGGCTAGGTCGGGATGTGGGTCAGCGCCAACGAGCCTGGCCTGAG---CACGAG-GAATGGAA-A---GCA-------TTTCGG---------TGCA-ATC-CAGGATCCGAGCT-CGGTCCTGC-GC--CTTGGCCTCCCACGGATCGGCCTTGCTGCGGGG--GTCTGTTG-AC-AGTTGCA-------CAC-AGACTT--GT-CTG---------TGTGTTAACCA-GCAGAATTCA--CCTTCGGCCACCATTTAACGGTCAACTCAGAACTGGCACGGACCGGGAGAATCCGACTGTCTAATTAAAGCAAAGCATTGCGATGGCTCTAGCTGAGTGTTTACGCAATGTGATTTCTGCCCAGTGCTCTGAATGTCAACGTGAAGAAATTCAAGC-AAGCGCGGGTAAACGGCGGGAGTAACTATGACTCTCTTAAGGTAGCCAAATGCCTCGTCATCTAATTAGTGACGCGCATGAATGGATTAACGAGATTCTCA-CTGTCCCTATCTACTATCTAGCGAAACCACTGCCAAGGGAACGGGCTTGGAAAAATTAGCGGGGAAAGAAGACCCTGTTGAGCTTGACTCTAGTCTGG |
| *Miranus serrulatus* | ----------------------TCGCGCCAGATCGTACCCATATCCGCAGCAGGTCTCCAAGGTTAAGAGCCTCTAGTCGATAGAATAATGTAGGTAAGGGAAGTCGGCAAATTGGATC-CGTAACTTCGGGACAAGGATTGGCTC-TGAGGATCGGGGTGTGTCGGGCTTGGTCAGGATGTGTGTCAGCGCCAACGAGCCAGGCCTGAG---CACGAG-GAATGGAG-C---GCA-------TCTTGA---------TGTG-TTT-CAGGATCCGAGCT-CGGTCCTGC-GC--CTTGGCCTTCCACGGATTGGCCTTGCTGCGGGT-GTTCTGTTT-GT--GTTCCA-------CAT-AGACTT--GT-CTG---------TGTGTCAACCG-GCAGGATTCA--CCTTCGGCCACCATTTAACGGTCAACTCAGAACTGGCACGGACCGGGAGAATCCGACTGTCTAATTAAAGCAAAGCATTGCGATGGCTCCAACTGAGTGTTCACGCAATGTGATTTCTGCCCAGTGCTCTGAATGTCAACGTGAAGAAATTCAAGC-AAGCGCGGGTAAACGGCGGGAGTAACTATGACTCTCTTAAGGTAGCCAAATGCCTCGTCATCTAATTAGTGACGCGCATGAATGGATTAACGAGATTCTCA-CTGTCCCTATCTACTATCTAGCGAAACCACAGCCAAGGGAACGGGCTTGGAAAAATTAGCGGGGAAAGAAGA-------------------------- |
| *Miranus varians* | CAG-GTGAGGGCCACGCGGAAATCGCGCCAGATCGTACCCATATCCGCAGCAGGTCTCCAAGGTTAAGAGCCTCTAGTCGATAGAATAATGTAGGTAAGGGAAGTCGGCAAATTGGATC-CGTAACTTCGGGACAAGGATTGGCTC-TGAGGATCGGGGTGTGTCGGGCTTGGTCAGGATGTGTGTCAGCGCCAACGAGCCAGGCCTGAG---CACGAG-GAATGGAG-C---GCA-------TCTTGA---------TGTG-TTT-CAGGATCCGAGCT-CGGTCCTGC-GC--CTTGGCCTTCCACGGATTGGCCTTGCTGCGGGT-GTTCTGTTT-GT--GTTCCA-------CAT-AGACTT--GT-CTG---------TGTGTCAACCG-GCAGAATTCA--CCTTCGGCCACCATTTAACGGTCAACTCAGAACTGGCACGGACCGGGAGAATCCGACTGTCTAATTAAAGCAAAGCATTGCGATGGCTCCAACTGAGTGTTCACGCAATGTGATTTCTGCCCAGTGCTCTGAATGTCAACGTGAAGAAATTCAAGC-AAGCGCGGGTAAACGGCGGGAGTAACTATGACTCTCTTAAGGTAGCCAAATGCCTCGTCATCTAATTAGTGACGCGCATGAATGGATTAACGAGATTCTCA-CTGTCCCTATCTACTATCTAGCGAAACCACAGCCAAGGGAACGGGCTTGGAAAAATTAGCGGGGAAAGAAGACCCTGTTGAGCTTGACTCTAGTCTGG |
| *Monospinodelphax dantur* | CAG-GTGAGGGCCACGCGGAAGTCGCGCCGGATCGTACCCATATCCGCAGCAGGTCTCCAAGGTTAAGAGCCTCTAGTCGATAGAATAATGTAGGTAAGGGAAGTCGGCAAATTGGATC-CGTAACTTCGGAACAAGGATTGGCTC-TGAGGATCAGGGTGTGTCGGGCTAGGTTGGGATGTGGGTCAGCGCCAACGAGCCTGGCCTGAG---CACGAG-GAATGGAA-A---GCA-------CTTCGG---------TGCA-ATC-CAGGAGCCGAGCT-CGGTCCTGC-GC--CTTGGCCTCCCACGGATCGGCCTTGCTGCGGGG--GTCTGTTG-AC-AGTTGCA-------CAT-GGACTT--GT-CTG---------TGTGTTAACGCAGCAGAATTCA--CCTTCGGCCACCATTTAATGGTCAACTCAGAACTGGCACGGACTGGGAGAATCCGACTGTCTAATTAAAGCAAAGCATTGCGATGGCTCTAGCTGAGTGCTTACGCAATGTGATTTCTGCCCAGTGCTCTGAATGTCAACGTGAAGAAATTCAAGC-AAGCGCGGGTAAACGGCGGGAGTAACTATGACTCTCTTAAGGTAGCCAAATGCCTCGTCATCTAATTAGTGACGCGCATGAATGGATTAACGAGATTCTCA-CTGTCCCTATCTACTATCTAGCGAAACCACTGCCAAGGGAACGGGCTTGGAAAAATTAGCGGGGAAAGAAGACCCTGTTGAGCTTGACTCTAGTCTGG |
| *Muirodelphax arvensis* | -AG-GTGAGGGCCACGCGGAAGTCGCGCCAGATCGTACCCATATCCGCAGCAGGTCTCCAAGGTTAAGAGCCTCTAGTCGATAGAATAATGTAGGTAAGGGAAGTCGGCAAATTGGATC-CGTAACTTCGGGACAAGGATTGGCTC-TGAGGATCGGGGTGTGTCGGGCTAGGTCGGGATGTGGGTCAGCGCCAACGAGCCTGGCCTGAG---CACGAG-GAATGGAG-A---GCA-------CTTCGG---------TGCG-ATC-CAGGATCCGAGCT-CGGTCCTGC-GC--CTTGGCCTCCCACGGATCGGCCTTGCTGCGGGG--GTCTGTTG-AC-AGTTGCA-------CAT-GGACTT--GT-CTG---------TGTGTCAACCA-GCAGAATTCA--CCTTCGGCCACCATTTAACGGTCAACTCAGAACTGGCACGGACCGGGAGAATCCGACTGTCTAATTAAAGCAAAGCATTGCGATGGCTCTAGCTGAGTGTTTACGCAATGTGATTTCTGCCCAGTGCTCTGAATGTCAACGTGAAGAAATTCAAGC-AAGCGCGGGTAAACGGCGGGAGTAACTATGACTCTCTTAAGGTAGCCAAATGCCTCGTCATCTAATTAGTGACGCGCATGAATGGATTAACGAGATTCTCA-CTGTCCCTATCTACTATCTAGCGAAACCACTGCCAAGGGAACGGGCTTGGAAAAATTAGCGGGGAAAGAAGACCCTGTTGAGCTTGACTCTAGTCTGG |
| *Neocarinodelphax hainanensis* | CAG-GTGAGGGCCACGCGGAAGTCGCGCCGGATCGTACCCATATCCGCAGCAGGTCTCCAAGGTTAAGAGCCTCTAGTCGATAGAATAATGTAGGTAAGGGAAGTCGGCAAATTGGATC-CGTAACTTCGGAACAAGGATTGGCTC-TGAGGATCGGGGCGTGTCGGGCTAGGTCGGGATGTGGGTCGGCGCCAACGAGCCTGGCCTGAG---CACGAG-GAACGGAG-TGTGGCA-------TTTCGG---------TGCTCCTC-CGGGATCCGAGCT-CGGTCCTGC-GC--CTTGGCCTCCCACGGATCGGCCTTGCTGCGGGG--GTCTGCTG-AC-AGTTGCATTCATAGCAC-ACTTGT--GTGCTG---------AGTGTGGACGG-GTAGAATCTATCCCTTCGGCCGCCATTCAACGGTCAACTCAGAACTGGCACGGACCGGGAGAATCCGACTGTCTAATTAAAGCAAAGCATTGCGATGGCTCTAGCTGAGTGTTTACGCAATGTGATTTCTGCCCAGTGCTCTGAATGTCAAAGTGAAGAAATTCAAGC-AAGCGCGGGTAAACGGCGGGAGTAACTATGACTCTCTTAAGGTAGCCAAATGCCTCGTCATCTAATTAGTGACGCGCATGAATGGATTAACGAGATTCTCA-CTGTCCCTATCTACTATCTAGCGAAACCACTGCCAAGGGAACGGGCTTGGAAAAATTAGCGGGGAAAGAAGACCCTGTTGAGCTTGACTCTAGTCTGG |
| *Neomegamelanus elongatus* | CAGCGTGAGGGCCACGCGGAAGTCGCGCCGGATCGTACCCATATCCGCAGCAGGTCTCCAAGGTTAAGAGCCTCTAGTCGATAGAATAATGTAGGTAAGGGAAGTCGGCAAATTGGATC-CGTAACTTCGGGACAAGGATTGGCTC-TGAGGATCGGGGTGTGTCGGGCTAGGTCGGGATGTGGGTCAGCGCCAACGAGCCTGGCCTGAG---CACGAG-GAATGGAA-A---GCA-------TTTCGG---------TGCA-ATC-CAGGATCCGAGCT-CGGTCCTGC-GC--CTTGGCCTCCCACGGATCGGCCTTGCTGCGGGG--GTCTGTTG-AC-AGTTGCA-------CAT-GGACTT--GT-CTG---------TGTGTTAACCA-GCAGAATTCA--CCTTCGGCCACCGCTCAACGGTCAACTCAGAACTGGCACGGACCGGGAGAATCCGACTGTCTAATTAAAGCAAAGCATTGCGATGGCTCTAGCTGAGTGTTTACGCAATGTGATTTCTGCCCAGTGCTCTGAATGTCAACGTGAAGAAATTCAAGC-AAGCGCGGGTAAACGGCGGGAGTAACTATGACTCTCTTAAGGTAGCCAAATGCCTCGTCATCTAATTAGTGACGCGCATGAATGGATTAACGAGATTCTCA-CTGTCCCTATCTACTATCTAGCGAAACCACTGCCAAGGGAACGGGCTTGGAAAAATTAGCGGGGAAAGAAGACCCTGTTGAGCTTGACTCTAGTCTGG |
| *Neometopina orientalis* | ----------------------TCGCGCCAGATCGTACCCATATCCGCAGCAGGTCTCCAAGGTTAAGAGCCTCTAGTCGATAGAATAATGTAGGTAAGGGAAGTCGGCAAATTGGATC-CGTAACTTCGGGACAAGGATTGGCTC-TGAGGATCGGGGTGTGTCGGGCTTGGTCGGGATGTGGGTTAGCGCCAACGAGCCTGGCCTGAG---CACGAG-GAATGGAG-A---GCA-------TTTCGG---------TGCT-CTC-CAGGATCCGAGTT-CGGTCCTGC-GC--CTTGGCCTCCCACGGATCGGCCTTGCTGCGGGG--GTCTGGTG-AC-AGTTGCA-------CAC-GGACTT--GT-CTG---------TGTGTTAACCA-GCAGAATTCA--CCTTCGGCCACCATTTAACGGTCAACTCAGAACTGGCACGGACCGGGAGAATCCGACTGTCTAATTAAAGCAAAGCATTGCGATGGCTCTAGCTGAGTGTTTACGCAATGTGATTTCTGCCCAGTGCTCTGAATGTCAACGTGAAGAAATTCAAGC-AAGCGCGGGTAAACGGCGGGAGTAACTATGACTCTCTTAAGGTAGCCAAATGCCTCGTCATCTAATTAGTGACGCGCATGAATGGATTAACGAGATTCTCA-CTGTCCCTATCTACTATCTAGCGAAACCACTGCCAAGGGAACGGGCTTGGAAAAATTAGCGGGGAAAGAAGACCCTGTTGAGCTTGACTCTAGTCTGG |
| *Nilaparvata bakeri* | CAG-GTGAGGGCCACGCGGAAGTCGCGCCAGATCGTACCCATATCCGCAGCAGGTCTCCAAGGTTAAGAGCCTCTAGTCGATAGAATAATGTAGGTAAGGGAAGTCGGCAAATTGGATC-CGTAACTTCGGGACAAGGATTGGCTC-TGAGGATCGGGGTGTGTCGGGCTAGGTCGGGATGTGGGTCAGCGCCAACGAGCCTGGCCTGAG---CACGAG-GAATGGAA-A---GCG-------CTTCGG---------TGTG-ATC-CAGGATTCGAGCT-CGGTCCTGC-GC--CTTGGCCTCCCACGGATCGGCCTTGCTGCGGGG--GTCTGTTG-AC-AGTTGCA-------TAC-AGACTT--GT-CTG---------TGTGTCAACCA-GCAGAATTCA--CCTTCGGCCACCATTTAACGGTCAACTCAGAACTGGCACGGACTGGGAGAATCCGACTGTCTAATTAAAGCAAAGCATTGCGATGGCTCTAGCTGAGTGTTTACGCAATGTGATTTCTGCCCAGTGCTCTGAATGTCAACGTGAAGAAATTCAAGC-AAGCGCGGGTAAACGGCGGGAGTAACTATGACTCTCTTAAGGTAGCCAAATGCCTCGTCATCTAATTAGTGACGCGCATGAATGGATTAACGAGATTCTCA-CTGTCCCTATCTACTATCTAGCGAAACCACTGCCAAGGGAACGGGCTTGGAAAAATTAGCGGGGAAAGAAGACCCTGTTGAGCTTGACTCTAGTCTGG |
| *Nilaparvata lugens* | CAG-GTGAGGGCCACGCGGAAGTCGCGCCAGATCGTACCCATATCCGCAGCAGGTCTCCAAGGTTAAGAGCCTCTAGTCGATAGAATAATGTAGGTAAGGGAAGTCGGCAAATTGGATC-CGTAACTTCGGGACAAGGATTGGCTC-TGAGGATCGGGGTGTGTCGGGCTAGGTCGGGATGTGGGTCAGCGCCAACGAGCCTGGCCTGAG---CACGAG-GAATGGAA-A---GCA-------CTTCGG---------TGTG-ATC-CAGGATTCGAGCT-CGGTCCTGC-GC--CTTGGCCTCCCACGGATCGGCCTTGCTGCGGGG--GTCTGTTG-AC-AGTTGCA-------TAC-AGACTT--GT-CTG---------TGTGTCAACCA-GCAGAATTCA--CCTTCGGCCACCATTTAACGGTCAACTCAGAACTGGCACGGACTGGGAGAATCCGACTGTCTAATTAAAGCAAAGCATTGCGATGGCTCTAGCTGAGTGTTTACGCAATGTGATTTCTGCCCAGTGCTCTGAATGTCAACGTGAAGAAATTCAAGC-AAGCGCGGGTAAACGGCGGGAGTAACTATGACTCTCTTAAGGTAGCCAAATGCCTCGTCATCTAATTAGTGACGCGCATGAATGGATTAACGAGATTCTCA-CTGTCCCTATCTACTATCTAGCGAAACCACTGCCAAGGGAACGGGCTTGGAAAAATTAGCGGGGAAAGAAGACCCTGTTGAGCTTGACTCTAGTCTGG |
| *Nilaparvata muiri* | CAG-GTGAGGGCCACGCGGAAGTCGCGCCAGATCGTACCCATATCCGCAGCAGGTCTCCAAGGTTAAGAGCCTCTAGTCGATAGAATAATGTAGGTAAGGGAAGTCGGCAAATTGGATC-CGTAACTTCGGGACAAGGATTGGCTC-TGAGGATCGGGGTGTGTCGGGCTAGGTCGGGATGTGGGTCAGCGCCAACGAGCCTGGCCTGAG---CACGAG-GAATGGAA-A---GCG-------CTTCGG---------TGTG-ATC-CAGGATTCGAGCT-CGGTCCTGC-GC--CTTGGCCTCCCACGGATCGGCCTTGCTGCGGGG--GTCTGTTG-AC-AGTTGCA-------TAC-AGACTT--GT-CTG---------TGTGTCAACCA-GCAGAATTCA--CCTTCGGCCACCATTTAACGGTCAACTCAGAACTGGCACGGACTGGGAGAATCCGACTGTCTAATTAAAGCAAAGCATTGCGATGGCTCTAGCTGAGTGTTTACGCAATGTGATTTCTGCCCAGTGCTCTGAATGTCAACGTGAAGAAATTCAAGC-AAGCGCGGGTAAACGGCGGGAGTAACTATGACTCTCTTAAGGTAGCCAAATGCCTCGTCATCTAATTAGTGACGCGCATGAATGGATTAACGAGATTCTCA-CTGTCCCTATCTACTATCTAGCGAAACCACTGCCAAGGGAACGGGCTTGGAAAAATTAGCGGGGAAAGAAGACCCTGTTGAGCTTGACTCTAGTCTGG |
| *Nothodelphax neocclusa* | CAG-GTGAGGGCCACGCGGAAGTCGCGCCGGATCGTACCCATATCCGCAGCAGGTCTCCAAGGTTAAGAGCCTCTAGTCGATAGAATAATGTAGGTAAGGGAAGTCGGCAAATTGGATC-CGTAACTTCGGGACAAGGATTGGCTC-TGAGGATCGGGGTGTGTCGGGCTAGGTCGGGATGTGGGTCAGCGCCAACGAGCCTGGCCTGAG---CACGAG-GAATGGAG-A---GCA-------CTTCGG---------TGCA-ATC-CAGGATCCGAGCT-CGGTCCTGC-GC--CTTGGCCTCCCACGGATCGGCCTTGCTGCGGGG--GTCTGTTG-AC-AGTTGCA-------CAT-GGACTT--GT-CTG---------TGTGTTAACCA-GCAGAATTCA--CCTTCGGCCACCATTTAACGGTCAACTCAGAACTGGCACGGACCGGGAGAATCCGACTGTCTAATTAAAGCAAAGCATTGCGATGGCTCTAGCTGAGTGTTTACGCAATGTGATTTCTGCCCAGTGCTCTGAATGTCAACGTGAAGAAATTCAAGC-AAGCGCGGGTAAACGGCGGGAGTAACTATGACTCTCTTAAGGTAGCCAAATGCCTCGTCATCTAATTAGTGACGCGCATGAATGGATTAACGAGATTCTCA-CTGTCCCTATCTACTATCTAGCGAAACCACTGCCAAGGGAACGGGCTTGGAAAAATTAGCGGGGAAAGAAGACCCTGTTGAGCTTGACTCTAGTCTGG |
| *Numata muiri* | CAG-GTGAGGGCCACGCGGAAGTCGCGCCGGATCGTACCCATATCCGCAGCAGGTCTCCAAGGTTAAGAGCCTCTAGTCGATAGAATAATGTAGGTAAGGGAAGTCGGCAAATTGGATC-CGTAACTTCGGAACAAGGATTGGCTC-TGAGGATCAGGGTGTGTCGGGCTAGGTTGGGATGTGGGTCAGCGCCAACGAGCCTGGCCTGAG---CACGAG-GAATGGAG-A---GCA-------TTTCGG---------TGCA-ATC-CAGGAGCCGAGCT-CGGTCCTGC-GC--CTTGGCCTCCCACGGATCGGCCTTGCTGCGGGG--GTCTGTTG-AC-AGTTGCA-------CAT-AGACTT--GT-CTG---------TGTGTTAACGCAGCAGAATTCA--CCTTCGGCCACCATTTAATGGTCAACTCAGAACTGGCACGGACTGGGAGAATCCGACTGTCTAATTAAAGCAAAGCATTGCGATGGCTCTAGCTGAGTGCTTACGCAATGTGATTTCTGCCCAGTGCTCTGAATGTCAACGTGAAGAAATTCAAGC-AAGCGCGGGTAAACGGCGGGAGTAACTATGACTCTCTTAAGGTAGCCAAATGCCTCGTCATCTAATTAGTGACGCGCATGAATGGATTAACGAGATTCTCA-CTGTCCCTATCTACTATCTAGCGAAACCACTGCCAAGGGAACGGGCTTGGAAAAATTAGCGGGGAAAGAAGACCCTGTTGAGCTTGACTCTAGTCTGG |
| *Oecleopsis* sp*.* | CGG-GTGCGAGCTACGCGGAAATCGCGCCGGATCGTACCCATATCCGCAGCAGGTCTCCAAGGTTAAGAGCCTCTAGTCGATAGAATAATGTAGGTAAGGGAAGTCGGCAAATTGGATC-CGTAACTTCGGGACAAGGATTGGCTC-TGAGGATCGGGGCGTGTCGGGCTTGGTCGGGAAGCGGGTCAGCGCCAACGTGCCGGGCCTGGG---CGAGGG-GAATGAGGCGTTTCCG--AGTAATCTGGA---------CGCT-TTC-TGTGATCCGAGCT-CGGTCCCGT-GC--CTTGGCCTCCCGCGGATCTTCCTTGCTGCGGGG-TGTCGGCTGTTCTGGTCGCG--------GCTGGGCTTCTGC-CCG----------CCGTGGACGG-GTCGAATTTGTCCCTTCGGCCGCCATTCAACGATCAACTCAGAACTGGCACGGACTGGGAGAATCCGACTGTCTAATTAAAGCAAAGCATTGCGATGGCTCTAGCTGAGTGTTTACGCAATGTGATTTCTGCCCAGTGCTCTGAATGTCAACGTGAAGAAATTCAAGC-AAGCGCGGGTAAACGGCGGGAGTAACTATGACTCTCTTAAGGTAGCCAAATGCCTCGTCATCTAATTAGTGACGCGCATGAATGGATTAACGAGATTCTCA-CTGTCCCTATCTACTATCTAGCGAAACCACTGCCAAGGGAACGGGCTTGGAAAAATTAGCGGGGAAAGAAGACCCTGTTGAGCTTGACTCTAGTCTGG |
| *Oliarus* sp*.* | CGG-GTGCGAGCTACGCGGAAATCGCGCCGGATCGTACCCATATCCGCAGCAGGTCTCCAAGGTTAAGAGCCTCTAGTCGATAGAATAATGTAGGTAAGGGAAGTCGGCAAATTGGATC-CGTAACTTCGGGACAAGGATTGGCTC-TGAGGATCGGGGCGTGTCGGGCTTGGTCGGGAAGCGGGTCAGCGCCAACGTGCCGGGCCTGGG---CGAGGG-GAATGAGGCGTTTCCG--AGTAATCTGGA---------CGCT-TTC-TGTGATCCGAGCT-CGGTCCCGT-GC--CTTGGCCTCCCGCGGATCTTCCTTGCTGCGGGG-TGTCGGCTGTTCTGGTCGCG--------GCTGGGCTTCTGC-CCG----------CCGTGGACGG-GTCGAATTTGTCCCTTCGGCCGCCATTCAACGATCAACTCAGAACTGGCACGGACTGGGAGAATCCGACTGTCTAATTAAAGCAAAGCATTGCGATGGCTCTAGCTGAGTGTTTACGCAATGTGATTTCTGCCCAGTGCTCTGAATGTCAACGTGAAGAAATTCAAGC-AAGCGCGGGTAAACGGCGGGAGTAACTATGACTCTCTTAAGGTAGCCAAATGCCTCGTCATCTAATTAGTGACGCGCATGAATGGATTAACGAGATTCTCA-CTGTCCCTATCTACTATCTAGCGAAACCACTGCCAAGGGAACGGGCTTGGAAAAATTAGCGGGGAAAGAAGACCCTGTTGAGCTTGACTCTAGTCTGG |
| *Opiconsiva albicollis* | CAG-GTGAGGGCCACGCGGAAGTCGCGCCGGATCGTACCCATATCCGCAGCAGGTCTCCAAGGTTAAGAGCCTCTAGTCGATAGAATAATGTAGGTAAGGGAAGTCGGCAAATTGGATC-CGTAACTTCGGGACAAGGATTGGCTC-TGAGGATCGGGGTGTGTCGGGCTAGGTCGGGATGTGGGTCAGCGCCAACGAGCCTGGCCTGAG---CACGAG-GAATGGAG-A---GCA-------CTTCGG---------TGCG-ATC-CAGGATCCGAGCT-CGGTCCTGC-GC--CTTGGCCTCCCACGGATCGGCCTTGCTGCGGGG--GTCTGTTG-AC-AGTTGCA-------CAC-GGACTT--GT-TCG---------TGTGTTAACCA-GCAGAATTCA--CCTTCGGCCACCATTTAACGGTCAACTCAGAACTGGCACGGACCGGGAGAATCCGACTGTCTAATTAAAGCAAAGCATTGCGATGGCTCTAGCTGAGTGTTTACGCAATGTGATTTCTGCCCAGTGCTCTGAATGTCAACGTGAAGAAATTCAAGC-AAGCGCGGGTAAACGGCGGGAGTAACTATGACTCTCTTAAGGTAGCCAAATGCCTCGTCATCTAATTAGTGACGCGCATGAATGGATTAACGAGATTCTCA-CTGTCCCTATCTACTATCTAGCGAAACCACTGCCAAGGGAACGGGCTTGGAAAAATTAGCGGGGAAAGAAGACCCTGTTGAGCTTGACTCTAGTCTGG |
| *Opiconsiva albimarginata* | CAG-GTGAGGGCCACGCGGAAGTCGCGCCGGATCGTACCCATATCCGCAGCAGGTCTCCAAGGTTAAGAGCCTCTAGTCGATAGAATAATGTAGGTAAGGGAAGTCGGCAAATTGGATC-CGTAACTTCGGGACAAGGATTGGCTC-TGAGGATCGGGGTGTGTCGGGCTAGGTCGGGATGTGGGTCAGCGCCAACGAGCCTGGCCTGAG---CACGAG-GAATGGAG-A---GCG-------CTTCGG---------TGCA-ATC-CAGGATCCGAGCT-CGGTCCTGC-GC--CTTGGCCTCCCACGGATCGGCCTTGCTGCGGGG--GTCTGTTG--T-AGTTGCA-------CAT-GGACTT--GT-TCG---------TGTGTTAACCA-GCAGAATTCA--CCTTCGGCCACCATTTAACGGTCAACTCAGAACTGGCACGGACCGGGAGAATCCGACTGTCTAATTAAAGCAAAGCATTGCGATGGCTCTAGCTGAGTGTTTACGCAATGTGATTTCTGCCCAGTGCTCTGAATGTCAACGTGAAGAAATTCAAGC-AAGCGCGGGTAAACGGCGGGAGTAACTATGACTCTCTTAAGGTAGCCAAATGCCTCGTCATCTAATTAGTGACGCGCATGAATGGATTAACGAGATTCTCA-CTGTCCCTATCTACTATCTAGCGAAACCACTGCCAAGGGAACGGGCTTGGAAAAATTAGCGGGGAAAGAAGACCCTGTTGAGCTTGACTCTAGTCTGG |
| *Opiconsiva nigra* | CAG-GTGAGGGCCACGCGGAAGTCGCGCCGGATCGTACCCATATCCGCAGCAGGTCTCCAAGGTTAAGAGCCTCTAGTCGATAGAATAATGTAGGTAAGGGAAGTCGGCAAATTGGATC-CGTAACTTCGGGACAAGGATTGGCTC-TGAGGATCGGGGTGTGTCGGGCTAGGTCGGGATGTGGGTCAGCGCCAACGAGCCTGGCCTGAG---CACGAG-GAATGGAG-A---GCA-------TTTCGG---------TGCG-ATC-CAGGATCCGAGCT-CGGTCCTGC-GC--CTTGGCCTCCCACGGATCGGCCTTGCTGCGGGG--GTCTGTTG-AC-AGTTGCA-------CAT-GGACTT--GT-TCA---------TGTGTTAACCA-GCAGAATTCA--CCTTCGGCCACCATTTAACGGTCAACTCAGAACTGGCACGGACCGGGAGAATCCGACTGTCTAATTAAAGCAAAGCATTGCGATGGCTCTAGCTGAGTGTTTACGCAATGTGATTTCTGCCCAGTGCTCTGAATGTCAACGTGAAGAAATTCAAGC-AAGCGCGGGTAAACGGCGGGAGTAACTATGACTCTCTTAAGGTAGCCAAATGCCTCGTCATCTAATTAGTGACGCGCATGAATGGATTAACGAGATTCTCA-CTGTCCCTATCTACTATCTAGCGAAACCACTGCCAAGGGAACGGGCTTGGAAAAATTAGCGGGGAAAGAAGACCCTGTTGAGCTTGACTCTAGTCTGG |
| *Opiconsiva* sp*.* | CAG-GTGAGGGCCACGCGGAAGTCGCGCCGGATCGTACCCATATCCGCAGCAGGTCTCCAAGGTTAAGAGCCTCTAGTCGATAGAATAATGTAGGTAAGGGAAGTCGGCAAATTGGATC-CGTAACTTCGGGACAAGGATTGGCTC-TGAGGATCGGGGTGTGTCGGGCTAGGTCGGGATGTGGGTCAGCGCCAACGAGCCTGGCCTGAG---CACGAG-GAATGGAG-A---GCA-------TTTCGG---------TGCG-ATC-CAGGATCCGAGCT-CGGTCCTGC-GC--CTTGGCCTCCCACGGATCGGCCTTGCTGCGGGG--GTCTGTTG-AC-AGTTGCA-------CAT-GGACTT--GT-TCA---------TGTGTTAACCA-GCAGAATTCA--CCTTCGGCCACCATTTAACGGTCAACTCAGAACTGGCACGGACCGGGAGAATCCGACTGTCTAATTAAAGCAAAGCATTGCGATGGCTCTAGCTGAGTGTTTACGCAATGTGATTTCTGCCCAGTGCTCTGAATGTCAACGTGAAGAAATTCAAGC-AAGCGCGGGTAAACGGCGGGAGTAACTATGACTCTCTTAAGGTAGCCAAATGCCTCGTCATCTAATTAGTGACGCGCATGAATGGATTAACGAGATTCTCA-CTGTCCCTATCTACTATCTAGCGAAACCACTGCCAAGGGAACGGGCTTGGAAAAATTAGCGGGGAAAGAAGACCCTGTTGAGCTTGACTCTAGTCTGG |
| *Palego simulator* | CAG-GTGAGGGCCACGCGGAAGTCGCGCCGGATCGTACCCATATCCGCAGCAGGTCTCCAAGGTTAAGAGCCTCTAGTCGATAGAATAATGTAGGTAAGGGAAGTCGGCAAATTGGATC-CGTAACTTCGGAACAAGGATTGGCTC-TGAGGATCAGGGTGTGTCGGGCTAGGTTGGGATGTGGGTCAGCGCCAACGAGCCTGGCCTGAG---CACGAG-GAATGGAG-A---GCA-------TTTCGG---------TGCA-ATC-CAGGAGCCGAGCT-CGGTCCTGC-GC--CTTGGCCTCCCACGGATCGGCCTTGCTGCGGGG--GTCTGTTG-AC-AGTTGCA-------CAC-GGACTT--GT-CTG---------TGTGTTAACGCAGCAGAATTCA--CCTTCGGCCACCATTTAATGGTCAACTCAGAACTGGCACGGACTGGGAGAATCCGACTGTCTAATTAAAGCAAAGCATTGCGATGGCTCTAGCTGAGTGCTTACGCAATGTGATTTCTGCCCAGTGCTCTGAATGTCAACGTGAAGAAATTCAAGC-AAGCGCGGGTAAACGGCGGGAGTAACTATGACTCTCTTAAGGTAGCCAAATGCCTCGTCATCTAATTAGTGACGCGCATGAATGGATTAACGAGATTCTCA-CTGTCCCTATCTACTATCTAGCGAAACCACTGCCAAGGGAACGGGCTTGGAAAAATTAGCGGGGAAAGAAGACCCTGTTGAGCTTGACTCTAGTCTGG |
| *Paradelphacodes paludosa* | CAG-GTGAGGGCCACGCGGAAGTCGCGCCAGATCGTACCCATATCCGCAGCAGGTCTCCAAGGTTAAGAGCCTCTAGTCGATAGAATAATGTAGGTAAGGGAAGTCGGCAAATTGGATC-CGTAACTTCGGGACAAGGATTGGCTC-TGAGGATCGGGGTGTGTCGGGCTAGGTCGGGATGTGGGTCAGCGCCAACGAGCCTGGCCTGAG---CACGAG-GAATGGAA-A---GCG-------CTTCGG---------TGTG-ATC-CAGGATTCGAGCT-CGGTCCTGC-GC--CTTGGCCTCCCACGGATCGGCCTTGCTGCGGGG--GTCTGTTG-AC-AGTTGCA-------TAC-AGACTT--GT-CTG---------TGTGTCAACCA-GCAGAATTCA--CCTTCGGCCACCATTTAACGGTCAACTCAGAACTGGCACGGACTGGGAGAATCCGACTGTCTAATTAAAGCAAAGCATTGCGATGGCTCTAGCTGAGTGTTTACGCAATGTGATTTCTGCCCAGTGCTCTGAATGTCAACGTGAAGAAATTCAAGC-AAGCGCGGGTAAACGGCGGGAGTAACTATGACTCTCTTAAGGTAGCCAAATGCCTCGTCATCTAATTAGTGACGCGCATGAATGGATTAACGAGATTCTCA-CTGTCCCTATCTACTATCTAGCGAAACCACTGCCAAGGGAACGGGCTTGGAAAAATTAGCGGGGAAAGAAGACCCTGTTGAGCTTGACTCTAGTCTGG |
| *Paradelphax nigrostriata* | CAG-GTGAGGGCCACGCCGAAGTCGCGCCGGATCGTACCCATATCCGCAGCAGGTCTCCAAGGTTAAGAGCCTCTAGTCGATAGAATAATGTAGGTAAGGGAAGTCGGCAAATTGGATC-CGTAACTTCGGGACAAGGATTGGCTC-TGAGGATCGGGGTGTGTCGGGCTAGGTCGGGATGTGGGTCAGCGCCAACGAGCCTGGCCTGAG---CACGAG-GAATGGAG-A---GCG-------TTTCGG---------TGCA-ATC-CAGGATTCGAGCT-CGGTCCTGC-GC--CTTGGCCTCCCACGGATCGGCCTTGCTGCGGGG--GTCTGTTG-AC-AGTTGCA-------CAT-GGACTT--GT-CTG---------TGTGTCAACCA-GCAGAATTCA--CCTTCGGCCACCATTTAACGGTCAACTCAGAACTGGCACGGACCGGGAGAATCCGACTGTCTAATTAAAGCAAAGCATTGCGATGGCTCTAGCTGAGTGTTTACGCAATGTGATTTCTGCCCAGTGCTCTGAATGTCAACGTGAAGAAATTCAAGC-AAGCGCGGGTAAACGGCGGGAGTAACTATGACTCTCTTAAGGTAGCCAAATGCCTCGTCATCTAATTAGTGACGCGCATGAATGGATTAACGAGATTCTCA-CTGTCCCTATCTACTATCTAGCGAAACCACTGCCAAGGGAACGGGCTTGGAAAAATTAGCGGGGAAAGAAGACCCTGTTGAGCTTGACTCTAGTCTGG |
| *Paranectopia lasaensis* | CAG-GTGAGGGCCACGCGGAAGTCGCGCCAGATCGTACCCATATCCGCAGCAGGTCTCCAAGGTTAAGAGCCTCTAGTCGATAGAATAATGTAGGTAAGGGAAGTCGGCAAATTGGATC-CGTAACTTCGGGACAAGGATTGGCTC-TGAGGATCGGGGTGTGTCGGGCTAGGTTGGGATGTGGGTCAGCGCCAACGAGCCTGGCCTGAG---CACGAG-GAATGGAG-A---GCA-------TATATG---------TGTA-ATC-CAGGATCCGAGCT-CGGTCCTGC-GC--CTTGGCCTCCCACGGATCGGCCTTGCTGCGGGG--GTCTGCTT-TG-TATTGCT-------CAC-AGACTT--GT-CTG---------TGTGTCAACCA-GCAGAATTCA--CCTTCGGCCACCATTTAACGGTCAACTCAGAACTGGCACGGACCGGGAGAATCCGACTGTCTAATTAAAGCAAAGCATTGCGATGGCTCTAGCTGAGTGTTTACGCAATGTGATTTCTGCCCAGTGCTCTGAATGTCAACGTGAAGAAATTCAAGC-AAGCGCGGGTAAACGGCGGGAGTAACTATGACTCTCTTAAGGTAGCCAAATGCCTCGTCATCTAATTAGTGACGCGCATGAATGGATTAACGAGATTCTCA-CTGTCCCTATCTACTATCTAGCGAAACCACTGCCAAGGGAACGGGCTTGGAAAAATTAGCGGGGAAAGAAGACCCTGTTGAGCTTGACTCTAGTCTGG |
| *Pentastiridius* sp*.* | CGG-GTGAGGGCTACGCGGAAGTCGCGCCGGGTCGTACCCATATCCGCAGCAGGTCTCCAAGGTTAAGAGCCTCTAGTCGATAGAATAATGTAGGTAAGGGAAGTCGGCAAATTGGATC-CGTAACTTCGGGACAAGGATTGGCTC-TGAGGATCGGGGCGTGTCGGGCTTGGTCGGGAAGCGGGTCAGCGCCAACGAGCCGGGCCTGGG---CGAGGG-GAAGGTGA-CGTTCCA-GAGAAATCTGGG---------CGTC-TAC-TGTGATCCGAGCT-CGGTCCCGT-GC--CTTGGCCTCCCGCGGATCTTCCTTGCTGCGGGG-TCTTGGCTGTTCTAGTTGCG-------GCTGGGCCTTCGGGCCCG----------CCGTGGACGG-GTCGAATTTGTCCCTTCGTCCGCCATTCAACGATCAACTCAGAACTGGCACGGACCGGGAGAATCCGACTGTCTAATTAAAGCAAAGCATTGCGATGGCTCTAACTGAGTGTTTACGCAATGTGATTTCTGCCCAGTGCTCTGAATGTCAACGTGAAGAAATTCAAGC-AAGCGCGGGTAAACGGCGGGAGTAACTATGACTCTCTTAAGGTAGCCAAATGCCTCGTCATCTAATTAGTGACGCGCATGAATGGATTAACGAGATTCTCA-CTGTCCCTATCTACTATCTAGCGAAACCACTGCCAAGGGAACGGGCTTGGAAAAATTAGCGGGGAAAGAAGACCCTGTTGAGCTTGACTCTAGTCTGG |
| *Peregrinus maidis* | CAG-GTGAGGGCCACGCGGAAGTCGCGCCGGATCGTACCCATATCCGCAGCAGGTCTCCAAGGTTAAGAGCCTCTAGTCGATAGAATAATGTAGGTAAGGGAAGTCGGCAAATTGGATC-CGTAACTTCGGAACAAGGATTGGCTC-TGAGGATCAGGGTGTGTCGGGCTAGGTTGGGATGTGGGTCAGCGCCAACGAGCCTGGCCTGAG---CACGAG-GAATGGAG-A---GCA-------TTTCGG---------TGCT-ATC-CAGGAGCCGAGCT-CGGTCCTGC-GC--CTTGGCCTCCCACGGATCGGCCTTGCTGCGGGG--GTCTGTTG-AC-AGTTGCA-------TGA-GGACTT--GT-CTT---------TGTGTTAACACAGCAGAATTCA--CCTTCGGCCACCATTTAATGGTCAACTCAGAACTGGCACGGACTGGGAGAATCCGACTGTCTAATTAAAGCAAAGCATTGCGATGGCTCTAGCTGAGTGCTTACGCAATGTGATTTCTGCCCAGTGCTCTGAATGTCAACGTGAAGAAATTCAAGC-AAGCGCGGGTAAACGGCGGGAGTAACTATGACTCTCTTAAGGTAGCCAAATGCCTCGTCATCTAATTAGTGACGCGCATGAATGGATTAACGAGATTCTCA-CTGTCCCTATCTACTATCTAGCGAAACCACTGCCAAGGGAACGGGCTTGGAAAAATTAGCGGGGAAAGAAGACCCTGTTGAGCTTGACTCTAGTCTGG |
| *Pissonotus albovenosus* | CAG-GTGAGGGCCACGCGGAAGTCGCGCCGGATCGTACCCATATCCGCAGCAGGTCTCCAAGGTTAAGAGCCTCTAGTCGATAGAATAATGTAGGTAAGGGAAGTCGGCAAATTGGATC-CGTAACTTCGGGACAAGGATTGGCTC-TGAGGATCGGGGTGTGTCGGGCTAGGTCGGGATGTGGGTCAGTGCCAACGAGCCTGGCCTGAG---CACAAG-GAATGGAG--------------------------------CA-ATC-CAGGATCTGAGCT-CGGTCCTGC-GC--CTTGGCCTCCCACGGATCGGCCTTGCTGCGGGG--GTCTGTTG-AC-AGTTGCA-------TGC-AGACTT--GT-CTG---------TGTGTTAACCA-GCAGAATTCA--CCTTCGGCCACCATTTAACGGTCAACTCAGAACTGGCACGGACCGGGAGAATCCGACTGTCTAATTAAAGCAAAGCATTGCGATGGCTCTAGCTGAGTGTTTACGCAATGTGATTTCTGCCCAGTGCTCTGAATGTCAACGTGAAAAAATTCAAGC-AAGCGCGGGTAAACGGCGGGAGTAACTATGACTCTCTTAAGGTAGCCAAATGCCTCGTCATCTAATTAGTGACGCGCATGAATGGATTAACGAGATTCTCA-CTGTCCCTATCTACTATCTAGCGAAACCACTGCCAAGGGAACGGGCTTGGAAAAATTAGCGGGGAAAGAAGACCCTGTTGAGCTTGACTCTAGTCTGG |
| *Pissonotus brunneus* | CAG-GTGAGGGCCACGCGGAAGTCGCGCCGGATCGTACCCATATCCGCAGCAGGTCTCCAAGGTTAAGAGCCTCTAGTCGATAGAATAATGTAGGTAAGGGAAGTCGGCAAATTGGATC-CGTAACTTCGGGACAAGGATTGGCTC-TGAGGATCGGGGTGTGTCGGGCTAGGTCGGGATGTGGGTCAGTGCCAACGAGCCTGGCCTGAG---CACAAG-GAATGGAG--------------------------------CA-ATC-CAGGATCTGAGCT-CGGTCCTGC-GC--CTTGGCCTCCCACGGATCGGCCTTGCTGCGGGG--GTCTGTTG-AC-AGTTGCA-------TGC-AGACTT--GT-CTG---------TGTGTTAACCA-GCAGAATTCA--CCTTCGGCCACCATTTAACGGTCAACTCAGAACTGGCACGGACCGGGAGAATCCGACTGTCTAATTAAAGCAAAGCATTGCGATGGCTCTAGCTGAGTGTTTACGCAATGTGATTTCTGCCCAGTGCTCTGAATGTCAACGTGAAGAAATTCAAGC-AAGCGCGGGTAAACGGCGGGAGTAACTATGACTCTCTTAAGGTAGCCAAATGCCTCGTCATCTAATTAGTGACGCGCATGAATGGATTAACGAGATTCTCA-CTGTCCCTATCTACTATCTAGCGAAACCACTGCCAAGGGAACGGGCTTGGAAAAATTAGCGGGGAAAGAAGACCCTGTTGAGCTTGACTCTAGTCTGG |
| *Prokelisia dolus* | CAG-GTGAGGGCCACGCGGAAGTCGCGCCGGATCGTACCCATATCCGCAGCAGGTCTCCAAGGTTAAGAGCCTCTAGTCGATAGAATAATGTAGGTAAGGGAAGTCGGCAAATTGGATC-CGTAACTTCGGGACAAGGATTGGCTC-TGAGGATCGGGGTGTGTCGGGCTAGGTCGGGATGTGGGTCAGCGCCAACGAGCCTGGCCTGAG---CACGAG-GAATGGAG-A---GCA-------CTCCGG---------TGTC-ATC-CAGGATCCGAGCT-CGGTCCTGC-GC--CTTGGCCTCCCACGGATCGGCCTTGCTGCGGGG--GTCTGTTG-AC-AGTTGCA-------CAC-GGACTT--GT-CTG---------TGTGTCAACCA-GCAGAATTCA--CCTTCGGCCACCGCTCAACGGTCAACTCAGAACTGGCACGGACCGGGAGAATCCGACTGTCTAATTAAAGCAAAGCATTGCGATGGCTCTAGCTGAGTGTTTACGCAATGTGATTTCTGCCCAGTGCTCTGAATGTCAACGTGAAGAAATTCAAGC-AAGCGCGGGTAAACGGCGGGAGTAACTATGACTCTCTTAAGGTAGCCAAATGCCTCGTCATCTAATTAGTGACGCGCATGAATGGATTAACGAGATTCTCA-CTGTCCCTATCTACTATCTAGCGAAACCACTGCCAAGGGAACGGGCTTGGAAAAATTAGCGGGGAAAGAAGACCCTGTTGAGCTTGACTCTAGTCTGG |
| *Prokelisia marginata* | CAG-GTGAGGGCCACGCGGAAGTCGCGCCGGATCGTACCCATATCCGCAGCAGGTCTCCAAGGTTAAGAGCCTCTAGTCGATAGAATAATGTAGGTAAGGGAAGTCGGCAAATTGGATC-CGTAACTTCGGGACAAGGATTGGCTC-TGAGGATCGGGGTGTGTCGGGCTAGGTCGGGATGTGGGTCAGCGCCAACGAGCCTGGCCTGAG---CACGAG-GAATGGAG-G---GCA-------CTCCGG---------TGTC-TTT-CAGGATCCGAGCT-CGGTCCTGC-GC--CTTGGCCTCCCACGGATCGGCCTTGCTGCGGGG--GTCTGTTG-AC-AGTTGCA-------CAT-GGACTT--GT-CTG---------TGTGTCAACCA-GCAGAATTCA--CCTTCGGCCACCGCTCAACGGTCAACTCAGAACTGGCACGGACCGGGAGAATCCGACTGTCTAATTAAAGCAAAGCATTGCGATGGCTCTAGCTGAGTGTTTACGCAATGTGATTTCTGCCCAGTGCTCTGAATGTCAACGTGAAGAAATTCAAGC-AAGCGCGGGTAAACGGCGGGAGTAACTATGACTCTCTTAAGGTAGCCAAATGCCTCGTCATCTAATTAGTGACGCGCATGAATGGATTAACGAGATTCTCA-CTGTCCCTATCTACTATCTAGCGAAACCACTGCCAAGGGAACGGGCTTGGAAAAATTAGCGGGGAAAGAAGACCCTGTTGAGCTTGACTCTAGTCTGG |
| *Purohita sinica* | CAG-GTGAGGGCCACGCGGAAGTCGCGCCGGATCGTACCCATATCCGCAGCAGGTCTCCAAGGTTAAGAGCCTCTAGTCGATAGAATAATGTAGGTAAGGGAAGTCGGCAAATTGGATC-CGTAACTTCGGAACAAGGATTGGCTC-TGAGGATCGGGGCGTGTCGGGCTAGGTCGGGATGTGGGTCGGCGCCAACGAGCCTGGCCTGAG---CACGAG-GAACGGAG-T---GCA-------TTTCGG---------TGCG-CTT-CGGGATCCGAGCT-CGGTCCTGC-GC--CTTGGCCTCCCACGGATCGGCCTTGCTGCGGGG--GTCTGTTG-AC-AGTTGCA-------CAC-AGACTT--GTGCTG---------AGTGTGGACGG-GCAGAATCTATCCCTTCGGCCGCCATTCAACGGTCAACTCAGAACTGGCACGGACCGGGAGAATCCGACTGTCTAATTAAAGCAAAGCATTGCGATGGCTCTAGCTGAGTGTTTACGCAATGTGATTTCTGCCCAGTGCTCTGAATGTCAACGTGAAGAAATTCAAGC-AAGCGCGGGTAAACGGCGGGAGTAACTATGACTCTCTTAAGGTAGCCAAATGCCTCGTCATCTAATTAGTGACGCGCATGAATGGATTAACGAGATTCTCA-CTGTCCCTATCTACTATCTAGCGAAACCACTGCCAAGGGAACGGGCTTGGAAAAATTAGCGGGGAAAGAAGACCCTGTTGAGCTTGACTCTAGTCTGG |
| *Purohita taiwanensis* | CAG-GTGAGGGCCACGCGGAAGTCGCGCCGGATCGTACCCATATCCGCAGCAGGTCTCCAAGGTTAAGAGCCTCTAGTCGATAGAATAATGTAGGTAAGGGAAGTCGGCAAATTGGATC-CGTAACTTCGGAACAAGGATTGGCTC-TGAGGATCGGGGCGTGTCGGGCTAGGTCGGGATGTGGGTCGGCGCCAACGAGCCTGGCCTGAG---CACGAG-GAACGGAG-T---GCA-------TTTCGG---------TGCG-CTT-CGGGATCCGAGCT-CGGTCCTGC-GC--CTTGGCCTCCCACGGATCGGCCTTGCTGCGGGG--GTCTGTTG-AC-AGTTGCA-------CAC-AGACTT--GTGCTG---------AGTGTGGACGG-GCAGAATCTATCCCTTCGGCCGCCATTCAACGGTCAACTCAGAACTGGCACGGACCGGGAGAATCCGACTGTCTAATTAAAGCAAAGCATTGCGATGGCTCTAGCTGAGTGTTTACGCAATGTGATTTCTGCCCAGTGCTCTGAATGTCAACGTGAAGAAATTCAAGC-AAGCGCGGGTAAACGGCGGGAGTAACTATGACTCTCTTAAGGTAGCCAAATGCCTCGTCATCTAATTAGTGACGCGCATGAATGGATTAACGAGATTCTCA-CTGTCCCTATCTACTATCTAGCGAAACCACTGCCAAGGGAACGGGCTTGGAAAAATTAGCGGGGAAAGAAGACCCTGTTGAGCTTGACTCTAGTCTGG |
| *Purohita theognis* | CAG-GTGAGGGCCACGCGAAAGTCGCGCCGGATCGTACCCATATCCGCAGCAGGTCTCCAAGGTTAAGAGCCTCTAGTCGATAGAATAATGTAGGTAAGGGAAGTCGGCAAATTGGATC-CGTAACTTCGGAACAAGGATTGGCTC-TGAGGATCGGGGCGTGTCGGGCTAGGTCGGGATGTGAGTCGGCGCCAACGAGCCTGGCCTGAG---CACGAG-GAACGGAG-T---GTG-------TTTCGA---------TATG-CTC-CGGGATCCGAGCT-CGGTCCTGC-GC--CTTGGCCTCTCACGGAGCGGCCTTGCTGCGGGG--GTCTGCAG-AC-AGTTGCA-------CAC-AGACTC--GTGCTG---------AGTGTGGACGA-GCAGAATCTATCCCTTCGGCCGCCATTCAACGGTCAACTCAGAACTGGCACGGACCGGGAGAATCCGACTGTCTAATTAAAGCAAAGCATTGCGATGGCTCTAGCTGAGTGTTTACGCAATGTGATTTCTGCCCAGTGCTCTGAATGTCAACGTGAAGAAATTCAAGC-AAGCGCGGGTAAACGGCGGGAGTAACTATGACTCTCTTAAGGTAGCCAAATGCCTCGTCATCTAATTAGTGACGCGCATGAATGGATTAACGAGATTCTCA-CTGTCCCTATCTACTATCTAGCGAAACCACTGCCAAGGGAACGGGCTTGGAAAAATTAGCGGGGAAAGAAGACCCTGTTGAGCTTGACTCTAGTCTGG |
| *Reptalus squadricinctus* | CGG-GTGAGGGCTACGCGGAAGTCGCGCCGGGTCGTACCCATATCCGCAGCAGGTCTCCAAGGTTAAGAGCCTCTAGTCGATAGAATAATGTAGGTAAGGGAAGTCGGCAAATTGGATC-CGTAACTTCGGGACAAGGATTGGCTC-TGAGGATCGGGGCGTGTCGGGCTTGGTCGGGAAGCGGGTCAGCGCCAACGAGCCGGGCCTGGG---CGAGGG-GAAGGTAG-CGTTCCA-GAGAAATCTGGG---------CGTC-TTC-TGTGATCCGAGCT-CGGTCCCGT-GC--CTTGGCCTCCCGCGGATCTTCCTTGCTGCGGGG-TCTTGGCTGTTCTAGTTGCG-------GCTGGGCCTTCGGGCCCG----------CCGTGGACGG-GTCGAATTTGTCCCTTCGTCCGCCATTCAACGATCAACTCAGAACTGGCACGGACCGGGAGAATCCGACTGTCTAATTAAAGCAAAGCATTGCGATGGCTCTAACTGAGTGTTTACGCAATGTGATTTCTGCCCAGTGCTCTGAATGTCAACGTGAAGAAATTCAAGC-AAGCGCGGGTAAACGGCGGGAGTAACTATGACTCTCTTAAGGTAGCCAAATGCCTCGTCATCTAATTAGTGACGCGCATGAATGGATTAACGAGATTCTCA-CTGTCCCTATCTACTATCTAGCGAAACCACTGCCAAGGGAACGGGCTTGGAAAAATTAGCGGGGAAAGAAGACCCTGTTGAGCTTGACTCTAGTCTGG |
| *Rhombotoya psedomigripennis* | CAG-GTGAGGGCCACGCGGAAGTCGCGCCGGATCGTACCCATATCCGCAGCAGGTCTCCAAGGTTAAGAGCCTCTAGTCGATAGAATAATGTAGGTAAGGGAAGTCGGCAAATTGGATC-CGTAACTTCGGAACAAGGATTGGCTC-TGAGGATCAGGGTGTGTCGGGCTAGGTTGGGATGTGGGTCAGCGCCAACGAGCCTGGCCTGAG---CACGAG-GAATGGAG-A---GCA-------TTTCGG---------TGCA-ATC-CAGGAGCCGAGCT-CGGTCCTGC-GC--CTTGGCCTCCCACGGATCGGCCTTGCTGCGGGG--GTCTGTTG-AC-AGTTGCA-------CAT-GGACTT--GT-CTG---------TGTGTTAACGCAGCAGAATTCA--CCTTCGGCCACCATTTAATGGTCAACTCAGAACTGGCACGGACTGGGAGAATCCGACTGTCTAATTAAAGCAAAGCATTGCGATGGCTCTAGCTGAGTGCTTACGCAATGTGATTTCTGCCCAGTGCTCTGAATGTCAACGTGAAGAAATTCAAGC-AAGCGCGGGTAAACGGCGGGAGTAACTATGACTCTCTTAAGGTAGCCAAATGCCTCGTCATCTAATTAGTGACGCGCATGAATGGATTAACGAGATTCTCA-CTGTCCCTATCTACTATCTAGCGAAACCACTGCCAAGGGAACGGGCTTGGAAAAATTAGCGGGGAAAGAAGACCCTGTTGAGCTTGACTCTAGTCTGG |
| *Ribautodelphax bidentatus* | CAG-GTGAGGGCCACGCGGAAGTCGCGCCGGATCGTACCCATATCCGCAGCAGGTCTCCAAGGTTAAGAGCCTCTAGTCGATAGAATAATGTAGGTAAGGGAAGTCGGCAAATTGGATC-CGTAACTTCGGGACAAGGATTGGCTC-TGAGGATCGGGGTGTGTCGGGCTAGGTCGGGATGTGGGTCAGCGCCAACGAGCCTGGCCTGAG---CACGAG-GAATGGAG-A---GCA-------TTTCGG---------TGCG-ATC-CAGGATCCGAGCT-CGGTCCTGC-GC--CTTGGCCTCCCACGGATCGGCCTTGCTGCGGGG--GTCTGTTG-AC-AGTTGCA-------CAT-TGACTT--GT-CAG---------TGTGTCAACCA-GCAGAATTCA--CCTTCGGCCACCATTTAACGGTCAACTCAGAACTGGCACGGACCGGGAGAATCCGACTGTCTAATTAAAGCAAAGCATTGCGATGGCTCTAGCTGAGTGTTTACGCAATGTGATTTCTGCCCAGTGCTCTGAATGTCAACGTGAAGAAATTCAAGC-AAGCGCGGGTAAACGGCGGGAGTAACTATGACTCTCTTAAGGTAGCCAAATGCCTCGTCATCTAATTAGTGACGCGCATGAATGGATTAACGAGATTCTCA-CTGTCCCTATCTACTATCTAGCGAAACCACTGCCAAGGGAACGGGCTTGGAAAAATTAGCGGGGAAAGAAGACCCTGTTGAGCTTGACTCTAGTCTGG |
| *Ribautodelphax tuvinus* | CAG-GTGAGGGCCACGCGGAAGTCGCGCCAGATCGTACCCATATCCGCAGCAGGTCTCCAAGGTTAAGAGCCTCTAGTCGATAGAATAATGTAGGTAAGGGAAGTCGGCAAATTGGATC-CGTAACTTCGGGACAAGGATTGGCTC-TGAGGATCGGGGTGTGTCGGGCTAGGTCGGGATGTGGGTTAGCGCCAACGAGCCTGGCCTGAG---CACGAG-GAATGGAG-A---GCA-------TTTCGG---------TGCT-CTC-CAGGATCCGAGTT-CGGTCCTGC-GC--CTTGGCCTCCCACGGATCGGCCTTGCTGCGGGG--GTCTGTTG-AC-AGTTGCA-------CAC-GGACTT--GT-CTG---------TGTGTTAACCA-GCAGAATTCA--CCTTCGGCCACCATTTAACGGTCAACTCAGAACTGGCACGGACCGGGAGAATCCGACTGTCTAATTAAAGCAAAGCATTGCGATGGCTCTAGCTGAGTGTTTACGCAATGTGATTTCTGCCCAGTGCTCTGAATGTCAACGTGAAGAAATTCAAGC-AAGCGCGGGTAAACGGCGGGAGTAACTATGACTCTCTTAAGGTAGCCAAATGCCTCGTCATCTAATTAGTGACGCGCATGAATGGATTAACGAGATTCTCA-CTGTCCCTATCTACTATCTAGCGAAACCACTGCCAAGGGAACGGGCTTGGAAAAATTAGCGGGGAAAGAAGACCCTGTTGAGCTTGACTCTAGTCTGG |
| *Saccharosydne procerus* | CAG-GTGAGGGCCACGCGGAAGTCGCGCCGGATCGTACCCATATCCGCAGCAGGTCTCCAAGGTTAAGAGCCTCTAGTCGATAGAATAATGTAGGTAAGGGAAGTCGGCAAATTGGATC-CGTAACTTCGGGACAAGGATTGGCTC-TGAGGATCGGGGCGTGTCGGGCTAGGTTGGGATGTGGGTCAGCGCCAACGAGCCTGGCCTGAG---CACGAG-GAACGGAGCA---------------------------------ATC-CGGGATCCGAGCT-CGGTCCTGC-GC--CTTGGCCTCCCACGGATCAGCCTTGCTGCGGGG--GTCTGTTG-AC-AGTCGCA-------CAC-GGACTT--GT-TCG---------AGTGTGGACGG-GCAGAATCTATCCCTTCGGCCGCCATTCAACGGTCAACTCAGAACTGGCACGGACCGGGAGAATCCGACTGTCTAATTAAAGCAAAGCATTGCGATGGCTCTAACTGAGTTTTTACGCAATGTGATTTCTGCCCAGTGCTCTGAATGTCAACGTGAAGAAATTCAAGC-AAGCGCGGGTAAACGGCGGGAGTAACTATGACTCTCTTAAGGTAGCCAAATGCCTCGTCATCTAATTAGTGACGCGCATGAATGGATTAACGAGATTCTCA-CTGTCCCTATCTACTATCTAGCGAAACCACTGCCAAGGGAACGGGCTTGGAAAAATTAGCGGGGAAAGAAGACCCTGTTGAGCTTGACTCTAGTCTGG |
| *Sardia rostrota* | CAG-GTGAGGGCCACGCGGAAGTCGCGCCAGATCGTACCCATATCCGCAGCAGGTCTCCAAGGTTAAGAGCCTCTAGTCGATAGAATAATGTAGGTAAGGGAAGTCGGCAAATTGGATC-CGTAACTTCGGGACAAGGATTGGCTC-TGAGGATCGGGGTGTGTCGGGCTAGGTCGGGATGTGGGTTAGCGCCAACGAGCCTGGCCTGAG---CACGAG-GAATGGAG-A---GCA-------TTTCGG---------TGCA-ATC-CAGGATCCGAGTT-CGGTCCTGC-GC--CTTGGCCTCCCACGGATCGGCCTTGCTGCGGGG--GTCTGTTG-AC-AGTTGCA-------CAT-AGACTT--GT-CTG---------TGTGTTAACCA-GCAGAATTCA--CCTTCGGCCACCATTTAACGGTCAACTCAGAACTGGCACGGACCGGGAGAATCCGACTGTCTAATTAAAGCAAAGCATTGCGATGGCTCTAGCTGAGTGTTTACGCAATGTGATTTCTGCCCAGTGCTCTGAATGTCAACGTGAAGAAATTCAAGC-AAGCGCGGGTAAACGGCGGGAGTAACTATGACTCTCTTAAGGTAGCCAAATGCCTCGTCATCTAATTAGTGACGCGCATGAATGGATTAACGAGATTCTCA-CTGTCCCTATCTACTATCTAGCGAAACCACTGCCAAGGGAACGGGCTTGGAAAAATTAGCGGGGAAAGAAGACCCTGTTGAGCTTGACTCTAGTCTGG |
| *Sinolacme tortilla* | CAG-GTGAGGGCCACGCGGAAGTCGCGCCGGATCGTACCCATATCCGCAGCAGGTCTCCAAGGTTAAGAGCCTCTAGTCGATAGAATAATGTAGGTAAGGGAAGTCGGCAAATTGGATC-CGTAACTTCGGAACAAGGATTGGCTC-TGAGGATCAGGGTGTGTCGGGCTAGGTTGGGATGTGGGTCAGCGCCAACGAGCCTGGCCTGAG---CACGAG-GAATGGAG-A---GCA-------TTTCGG---------TGCA-ATC-CAGGAGCCGAGCT-CGGTCCTGC-GC--CTTGGCCTCCCACGGATCGGCCTTGCTGCGGGG--GTCTGTTG-AC-AGTTGCA-------CAC-AATCTT--GT--------------GTGTTAACGCAGCAGAATTCA--CCTTCGGCCACCATTTAATGGTCAACTCAGAACTGGCACGGACTGGGAGAATCCGACTGTCTAATTAAAGCAAAGCATTGCGATGGCTCTAGCTGAGTGCTTACGCAATGTGATTTCTGCCCAGTGCTCTGAATGTCAACGTGAAGAAATTCAAGC-AAGCGCGGGTAAACGGCGGGAGTAACTATGACTCTCTTAAGGTAGCCAAATGCCTCGTCATCTAATTAGTGACGCGCATGAATGGATTAACGAGATTCTCA-CTGTCCCTATCTACTATCTAGCGAAACCACTGCCAAGGGAACGGGCTTGGAAAAATTAGCGGGGAAAGAAGACCCTGTTGAGCTTGACTCTAGTCTGG |
| *Sogata hakonensis* | CAG-GTGAGGGCCACGCGGAAATCGCGCCAGATCGTACCCATATCCGCAGCAGGTCTCCAAGGTTAAGAGCCTCTAGTCGATAGAATAATGTAGGTAAGGGAAGTCGGCAAATTGGATC-CGTAACTTCGGGACAAGGATTGGCTC-TGAGGATCGGGGTGTGTCGGGCTTGGTCAGGATGTGTGTCAGCGCCAACGAGCCAGGCCTGAG---CACGAG-GAATGGAA-T---GCA-------TCTTGA---------TGTG-TTT-CAGGATCTGAGCT-CGGTCCTGC-GC--CTTGGCCTTCCACGGATTGGCCTTGCTGCGGGT-GTTCTGTTT-TG-TGTTCCA-------CAT-AGACTT--GT-CTG---------TGTGTTAACCA-GCAGGATCCA--CCTTCGGCCACCATTTAACGGTCAACTCAGAACTGGCACGGACCGGGAGAATCCGACTGTCTAATTAAAGCAAAGCATTGCGATGGCTCCAACTGAGTGTTCACGCAATGTGATTTCTGCCCAGTGCTCTGAATGTCAACGTGAAGAAATTCAAGC-AAGCGCGGGTAAACGGCGGGAGTAACTATGACTCTCTTAAGGTAGCCAAATGCCTCGTCATCTAATTAGTGACGCGCATGAATGGATTAACGAGATTCTCA-CTGTCCCTATCTACTATCTAGCGAAACCACAGCCAAGGGAACGGGCTTGGAAAAATTAGCGGGGAAAGAAGACCCTGTTGAGCTTGACTCTAGTCTGG |
| *Sogata nigrifrons* | CAG-GTGAGGGCCACGCGGAAATCGCGCCAGATCGTACCCATATCCGCAGCAGGTCTCCAAGGTTAAGAGCCTCTAGTCGATAGAATAATGTAGGTAAGGGAAGTCGGCAAATTGGATC-CGTAACTTCGGGACAAGGATTGGCTC-TGAGGATCGGGGTGTGTCGGGCTTGGTCAGGATGTGTGTCAGCGCCAACGAGCCAGGCCTGAG---CACGAG-GAATGGAA-T---GCA-------TCTTGA---------TGTG-TTT-CAGGATCTGAGCT-CGGTCCTGC-GC--CTTGGCCTTCCACGGATTGGCCTTGCTGCGGGT-GTTCTGTTT-TG-TGTTCCA-------CAT-AGACTT--GT-CTG---------TGTGTTAACCA-GCAGGATCCA--CCTTCGGCCACCATTTAACGGTCAACTCAGAACTGGCACGGACCGGGAGAATCCGACTGTCTAATTAAAGCAAAGCATTGCGATGGCTCCAACTGAGTGTTCACGCAATGTGATTTCTGCCCAGTGCTCTGAATGTCAACGTGAAGAAATTCAAGC-AAGCGCGGGTAAACGGCGGGAGTAACTATGACTCTCTTAAGGTAGCCAAATGCCTCGTCATCTAATTAGTGACGCGCATGAATGGATTAACGAGATTCTCA-CTGTCCCTATCTACTATCTAGCGAAACCACAGCCAAGGGAACGGGCTTGGAAAAATTAGCGGGGAAAGAAGACCCTGTTGAGCTTGACTCTAGTCTGG |
| *Sogatella furcifera* | CAG-GTGAGGGCCACGCGGAAGTCGCGCCAGATCGTACCCATATCCGCAGCAGGTCTCCAAGGTTAAGAGCCTCTAGTCGATAGAATAATGTAGGTAAGGGAAGTCGGCAAATTGGATC-CGTAACTTCGGGACAAGGATTGGCTC-TGAGGATCGGGGTGTGTCGGGCTAGGTCGGGATGTGGGTTAGCGCCAACGAGCCTGGCCTGAG---CACGAG-GAATGGAG-A---GCA-------TTTCGG---------TGCA-ATC-CAGGATCCGAGTT-CGGTCCTGC-GC--CTTGGCCTCCCACGGATCGGCCTTGCTGCGGGG--GTCTGTTG--T-AGTTGCA-------CAC-GGACTT--GT-CTG---------TGTGTTAACCA-GCAGAATTCA--CCTTCGGCCACCATTTAACGGTCAACTCAGAACTGGCACGGACCGGGAGAATCCGACTGTCTAATTAAAGCAAAGCATTGCGATGGCTCTAGCTGAGTGTTTACGCAATGTGATTTCTGCCCAGTGCTCTGAATGTCAACGTGAAGAAATTCAAGC-AAGCGCGGGTAAACGGCGGGAGTAACTATGACTCTCTTAAGGTAGCCAAATGCCTCGTCATCTAATTAGTGACGCGCATGAATGGATTAACGAGATTCTCA-CTGTCCCTATCTACTATCTAGCGAAACCACTGCCAAGGGAACGGGCTTGGAAAAATTAGCGGGGAAAGAAGACCCTGTTGAGCTTGACTCTAGTCTGG |
| *Spartidelphax detectus* | CAG-GTGAGGGCCACGCGGAAGTCGCGCCGGATCGTACCCATATCCGCAGCAGGTCTCCAAGGTTAAGAGCCTCTAGTCGATAGAATAATGTAGGTAAGGGAAGTCGGCAAATTGGATC-CGTAACTTCGGGACAAGGATTGGCTC-TGAGGATCGGGGTGTGTCGGGCTAGGTCGGGATGTGGGTCAGCGCCAACGAGCCTGGCCTGAG---CACGAG-GAATGGAG-A---GCA-------TTTCGG---------TGCG-ATC-CAGGATCCGAGCT-CGGTCCTGC-GC--CTTGGCCTCCCACGGATCGGCCTTGCTGCGGGG--GTCTGTTG-AC-AGTTGCA-------CAT-GGATAC--GT-TCG---------TGTGTTAACCA-GCAGAATTCA--CCTTCGGCCACCATTTAACGGTCAACTCAGAACTGGCACGGACCGGGAGAATCCGACTGTCTAATTAAAGCAAAGCATTGCGATGGCTCTAGCTGAGTGTTTACGCAATGTGATTTCTGCCCAGTGCTCTGAATGTCAACGTGAAGAAATTCAAGC-AAGCGCGGGTAAACGGCGGGAGTAACTATGACTCTCTTAAGGTAGCCAAATGCCTCGTCATCTAATTAGTGACGCGCATGAATGGATTAACGAGATTCTCA-CTGTCCCTATCTACTATCTAGCGAAACCACTGCCAAGGGAACGGGCTTGGAAAAATTAGCGGGGAAAGAAGACCCTGTTGAGCTTGACTCTAGTCTGG |
| *Specinervures basifusca* | CAG-GTGAGGGCCACGCGGAAGTCGCGCCGGATCGTACCCATATCCGCAGCAGGTCTCCAAGGTTAAGAGCCTCTAGTCGATAGAATAATGTAGGTAAGGGAAGTCGGCAAATTGGATC-CGTAACTTCGGAACAAGGATTGGCTC-TGAGGATCGGGGCGTGTCGGGCTAGGTCGGGATGTGGGTCGGCGCCAACGAGCCTGGCCTGAG---CACGAG-GAACGGAGTT---GCA-------CTTCGG---------TGCG-CTC-CGGGATCCGAGCT-CGGTCCTGC-GC--CTTGGCCTCCCACGGATCGGCCTTGCTGCGGGG--GTCTGTTG-AC-AGTTGCA-------TGC-AGACTT--GTGCTG---------TGTGTGGACGG-GCAGAATCTATCCCTTCGGCCGCCATTCAACGGTCAACTCAGAACTGGCACGGACCGGGAGAATCCGACTGTCTAATTAAAGCAAAGCATTGCGATGGCTCTAGCTGAGTGTTTACGCAATGTGATTTCTGCCCAGTGCTCTGAATGTCAACGTGAAGAAATTCAAGC-AAGCGCGGGTAAACGGCGGGAGTAACTATGACTCTCTTAAGGTAGCCAAATGCCTCGTCATCTAATTAGTGACGCGCATGAATGGATTAACGAGATTCTCA-CTGTCCCTATCTACTATCTAGCGAAACCACTGCCAAGGGAACGGGCTTGGAAAAATTAGCGGGGAAAGAAGACCCTGTTGAGCTTGACTCTAGTCTGG |
| *Stenocranus montanus* | CGG-GTGAGGGCCACGCGGAAGTCGCGCCGGATCGTACCCATATCCGCAGCAGGTCTCCAAGGTTAAGAGCCTCTAGTCGATAGAATAATGTAGGTAAGGGAAGTCGGCAAATTGGATC-CGTAACTTCGGAACAAGGATTGGCTC-TGAGGATCGGGGTGTGTCGGGCTAGGTCGGGATGTGGGTCAGCGCCAACGAGCCTGGCCTGGG---CGAGAG-GAACGGGT-G---TCA------CTCTCGG--------GTGAA-ATC-CGGGATCCAAGCT-TGGTCCTGT-GC--CTTGGCCTCCCACGGATCGGCCTTGCTGCGGGG-TGTCTGTTG-AC-AGTTGCA-------CAC-AGACTT--GTGCTG---------AGTGTGGACGG-GCAGAATTTATCCCTTCGGCCACCATTCAACGGTCAACTCAGAACTGGCACGGACCGGGAGAATCCGACTGTCTAATTAAAGCAAAGCATTGCGAGGGCTCTAGCTGAGTGTTCACGCAATGTGATTTCTGCCCAGTGCTCTGAATGTCAACGTGAAGAAATTCAAGC-AAGCGCGGGTAAACGGCGGGAGTAACTATGACTCTCTTAAGGTAGCCAAATGCCTCGTCATCTAATTAGTGACGCGCATGAATGGATTAACGAGATTCTCA-CTGTCCCTATCTACTATCTAGCGAAACCACTGCCAAGGGAACGGGCTTGGAAAAATTAGCGGGGAAAGAAGACCCTGTTGAGCTTGACTCTAGTCTGG |
| *Stiropis nigrifrons* | CAG-GTGAGGGCCACGCGGAAGTCGCGCCGGATCGTACCCATATCCGCAGCAGGTCTCCAAGGTTAAGAGCCTCTAGTCGATAGAATAATGTAGGTAAGGGAAGTCGGCAAATTGGATC-CGTAACTTCGGGACAAGGATTGGCTC-TGAGGATCGGGGTGTGTCGGGCTAGGTCGGGATGTGGGTCAGCGCCAACGAGCCTGGCCTGAG---CACGAG-GAATGGAG-A---GCA-------TTTCGA---------TGCA-ATC-CAGGATCCGAGCT-CGGTCCTGC-GC--CTTGGCCTCCCACGGATCGGCCTTGCTGCGGGG--GTCTGTTG-AC-AGTTGCA-------CAC-AGACTT--GT-TTG---------TGTGTTAACCA-GCAGAATTCA--CCTTCGGCCACCATTTAACGGTCAACTCAGAACTGGCACGGACCGGGAGAATCCGACTGTCTAATTAAAGCAAAGCATTGCGATGGCTCTAGCTGAGTGTTTACGCAATGTGATTTCTGCCCAGTGCTCTGAATGTCAACGTGAAGAAATTCAAGC-AAGCGCGGGTAAACGGCGGGAGTAACTATGACTCTCTTAAGGTAGCCAAATGCCTCGTCATCTAATTAGTGACGCGCATGAATGGATTAACGAGATTCTCA-CTGTCCCTATCTACTATCTAGCGAAACCACTGCCAAGGGAACGGGCTTGGAAAAATTAGCGGGGAAAGAAGACCCTGTTGAGCTTGACTCTAGTCTGG |
| *Struebingianella detecta* | CAG-GTGAGGGCCACGCGGAAGTCGCGCCGGATCGTACCCATATCCGCAGCAGGTCTCCAAGGTTAAGAGCCTCTAGTCGATAGAATAATGTAGGTAAGGGAAGTCGGCAAATTGGATC-CGTAACTTCGGGACAAGGATTGGCTC-TGAGGATCGGGGTGTGTCGGGCTAGGTCGGGATGTGGGTCAAAGCCGATGAGCCTGGCCTGAG---CACGAG-GAATGGAG-A---GCA-------TTTCGG---------TGCG-ATC-CAGGATCCGAGCT-CGGTCCTGTAGC--CTTGGCCTCCCACGGATCGGCCTTGCTGCGGGG--GTCTGTTG-AC-AGTTGCA-------CAC-AGACTT--GT-CTG---------TGTGTTAACCA-GCAGAATTCA--CCTTCGGCCACCATTTAACGGTCAACTCAGAACTGGCACGGACCGGGAGAATCCGACTGTCTAATTAAAGCAAAGCATTGCGATGGCTCTAGCTGAGTGTTCACGCAATGTGATTTCTGCCCAGTGCTCTGAATGTCAACGTGAAGAAATTCAAGC-AAGCGCGGGTAAACGGCGGGAGTAACTATGACTCTCTTAAGGTAGCCAAATGCCTCGTCATCTAATTAGTGACGCGCATGAATGGATTAACGAGATTCTCA-CTGTCCCTATCTACTATCTAGCGAAACCACTGCCAAGGGAACGGGCTTGGAAAAATTAGCGGGGAAAGAAGACCCTGTTGAGCTTGACTCTAGTCTGG |
| *Sulculus sulcatus* | CAG-GTGAGGGCCACGCGGAAGTCGCGCCGGATCGTACCCATATCCGCAGCAGGTCTCCAAGGTTAAGAGCCTCTAGTCGATAGAATAATGTAGGTAAGGGAAGTCGGCAAATTGGATC-CGTAACTTCGGAACAAGGATTGGCTC-TGAGGATCAGGGTGTGTCGGGCTAGGTTGGGATGTGGGTCAGTGCCAACGAGCCTGGCCTGAG---CACGAG-GAATGGAG-A---GCA-------TTTCGG---------TGCA-ATC-CAGGAGCCGAGCT-CGGTCCTGC-GC--CTTGGCCTCCCACGGATCGGCCTTGCTGCGGGG--GTCTGTTG-AC-AGTTGCA-------TAC-GGACTT--GT-CTG---------TGTGTTAACGCAGCAGAATTCA--CCTTCGGCCACCATTTAATGGTCAACTCAGAACTGGCACGGACTGGGAGAATCCGACTGTCTAATTAAAGCAAAGCATTGCGATGGCTCTAGCTGAGTGCTTACGCAATGTGATTTCTGCCCAGTGCTCTGAATGTCAACGTGAAGAAATTCAAGC-AAGCGCGGGTAAACGGCGGGAGTAACTATGACTCTCTTAAGGTAGCCAAATGCCTCGTCATCTAATTAGTGACGCGCATGAATGGATTAACGAGATTCTCA-CTGTCCCTATCTACTATCTAGCGAAACCACTGCCAAGGGAACGGGCTTGGAAAAATTAGCGGGGAAAGAAGACCCTGTTGAGCTTGACTCTAGTCTGG |
| *Tagosodes orizicolus* | ---------------------------------------------------------------------------------------------------------------------------------------------------------------------------------------------------------------------------------------------------------------------------------------------------------------------------------------------------------------------------------------------------------------------------------------AACGGTCAACTCAGAACTGGCACGGACCGGGAGAATCCGACTGTCTAATTAAAGCAAAGCATTGCGATGGCTCTAGCTGAGTGTTTACGCAATGTGATTTCTGCCCAGTGCTCTGAATGTCAACGTGAAGAAATTCAAGC-AAGCGCGGGTAAACGGCGGGAGTAACTATGACTCTCTTAAGGTAGCCAAATGCCTCGTCATCTAATTAGTGACGCGCATGAATGGATTAACGAGATTCTCACCTGTCCCTATCTACTATCTAGCGAAACCACTGCCAAGGGAACGGGCTTGGAAAAATTAGCGGGGAAAGAAGACCCTGTTGAGCTTGACTCTAGTCTGG |
| *Tarophagus colocasiae* | CAG-GTGAGGGCCACGCGGAAGTCGCGCCGGATCGTACCCATATCCGCAGCAGGTCTCCAAGGTTAAGAGCCTCTAGTCGATAGAATAATGTAGGTAAGGGAAGTCGGCAAATTGGATC-CGTAACTTCGGAACAAGGATTGGCTC-TGAGGATCAGGGTGTGTCGGGCTAGGTTGGGATGTGGGTCAGCGCCAACGAGCCTGGCCTGAG---CACGAG-GAATGGAG-A---GCA-------TTTCGG---------TGCT-ATC-CAGGAGCCGAGCT-CGGTCCTGC-GC--CTTGGCCTCCCACGGATCGGCCTTGCTGCGGGG--GTCTGTTG-AC-AGTTGCA-------CAT-AGATTC--GT-CTG---------TGTGTTAACGCAGCAGAATTCA--CCTTCGGCCACCATTTAATGGTCAACTCAGAACTGGCACGGACTGGGAGAATCCGACTGTCTAATTAAAGCAAAGCATTGCGATGGCTCTAGCTGAGTGCTTACGCAATGTGATTTCTGCCCAGTGCTCTGAATGTCAACGTGAAGAAATTCAAGC-AAGCGCGGGTAAACGGCGGGAGTAACTATGACTCTCTTAAGGTAGCCAAATGCCTCGTCATCTAATTAGTGACGCGCATGAATGGATTAACGAGATTCTCA-CTGTCCCTATCTACTATCTAGCGAAACCACTGCCAAGGGAACGGGCTTGGAAAAATTAGCGGGGAAAGAAGACCCTGTTGAGCTTGACTCTAGTCTGG |
| *Terauchiana singularis* | CGG-GTGAGGGCCACGCGGAAGTCGCGCCGGATCGTACCCATATCCGCAGCAGGTCTCCAAGGTTAAGAGCCTCTAGTCGATAGAATAATGTAGGTAAGGGAAGTCGGCAAATTGGATC-CGTAACTTCGGAACAAGGATTGGCTC-TGAGGATCGGGGTGCGTCGGGCTAGGTCGGGATGTGGGTCAGCGCCAACGAGCCTGGCCTGGG---CGAGGG-GAACGGGT-G---TCA------CTTTCGG--------GTGAA-ATC-CGGGATCCGAGCT-TGGTCCTGT-GC--CTTGGCCTCCCACGGATCGGCCTTGCTGCGGGG-TGTCTGTTG-AC-AGTTGCA-------CGC-AGACTT--GTGCTG---------AGTGTGGACGG-GCAGAATTTATCCCTTCGGCCACCATTCAACGGTCAACTCAGAACTGGCACGGACCGGGAGAATCCGACTGTCTAATTAAAGCAAAGCATTGCGAGGGCTCTAGCTGAGTGTTCACGCAATGTGATTTCTGCCCAGTGCTCTGAATGTCAACGTGAAGAAATTCAAGC-AAGCGCGGGTAAACGGCGGGAGTAACTATGACTCTCTTAAGGTAGCCAAATGCCTCGTCATCTAATTAGTGACGCGCATGAATGGATTAACGAGATTCTCA-CTGTCCCTATCTACTATCTAGCGAAACCACTGCCAAGGGAACGGGCTTGGAAAAATTAGCGGGGAAAGAAGACCCTGTTGAGCTTGACTCTAGTCTGG |
| *Terthron albovittatum* | CAG-GTGAGGGCCACGCGGAAGTCGCGCCAGATCGTACCCATATCCGCAGCAGGTCTCCAAGGTTAAGAGCCTCTAGTCGATAGAATAATGTAGGTAAGGGAAGTCGGCAAATTGGATC-CGTAACTTCGGGACAAGGATTGGCTC-TGAGGATCGGGGTGTGTCGGGCTAGGTCGGGATGTGGGTCAGCGCCAACGAGCCTGGCCTGAG---CACGAG-GAATGGAG-A---GCA-------TTTCGG---------TGCG-ATC-CAGGATCCGAGCT-CGGTCCTGC-GC--CTTGGCCTCCCACGGATCGGCCTTGCTGCGGGG--GTCTGTTG-AC-AGTTGCA-------CAC-GGACTT--GT-CTG---------TGTGTTAACCA-GCAGAATTCA--CCTTCGGCCACCATTTAACGGTCAACTCAGAACTGGCACGGACCGGGAGAATCCGACTGTCTAATTAAAGCAAAGCATTGCGATGGCTCTAGCTGAGTGTTTACGCAATGTGATTTCTGCCCAGTGCTCTGAATGTCAACGTGAAGAAATTCAAGC-AAGCGCGGGTAAACGGCGGGAGTAACTATGACTCTCTTAAGGTAGCCAAATGCCTCGTCATCTAATTAGTGACGCGCATGAATGGATTAACGAGATTCTCA-CTGTCCCTATCTACTATCTAGCGAAACCACTGCCAAGGGAACGGGCTTGGAAAAATCAGCGGGGAAAGAAGACCCTGTTGAGCTTGACTCTAGTCTGG |
| *Toya terryi* | CAG-GTGAGGGCCACGCGGAAGTCGCGCCAGATCGTACCCATATCCGCAGCAGGTCTCCAAGGTTAAGAGCCTCTAGTCGATAGAATAATGTAGGTAAGGGAAGTCGGCAAATTGGATC-CGTAACTTCGGGACAAGGATTGGCTC-TGAGGATCGGGGTGTGTCGGGCTAGGTCGGGATGTGGGTCAGCGCCAACGAGCCTGGCCTGAG---CACGAG-GAATGGAG-A---GCA-------TTTCGG---------TGCA-ATC-CAGGATCCGAGCT-CGGTCCTGC-GC--CTTGGCCTCCCACGGATCGGCCTTGCTGCGGGG--GTCTGTTG-AC-AGTTGCA-------CAC-AGACTT--GT-CTG---------TGTGTTAACCA-GCAGAATTCA--CCTTCGGCCACCATTTAACGGTCAACTCAGAACTGGCACGGACCGGGAGAATCCGACTGTCTAATTAAAGCAAAGCATTGCGATGGCTCTAGCTGAGTGTTTACGCAATGTGATTTCTGCCCAGTGCTCTGAATGTCAACGTGAAGAAATTCAAGC-AAGCGCGGGTAAACGGCGGGAGTAACTATGACTCTCTTAAGGTAGCCAAATGCCTCGTCATCTAATTAGTGACGCGCATGAATGGATTAACGAGATTCTCA-CTGTCCCTATCTACTATCTAGCGAAACCACTGCCAAGGGAACGGGCTTGGAAAAATTAGCGGGGAAAGAAGACCCTGTTGAGCTTGACTCTAGTCTGG |
| *Toyoides albipennis* | CAG-GTGAGGGCCACGCGGAAGTCGCGCCGGATCGTACCCATATCCGCAGCAGGTCTCCAAGGTTAAGAGCCTCTAGTCGATAGAATAATGTAGGTAAGGGAAGTCGGCAAATTGGATC-CGTAACTTCGGGACAAGGATTGGCTC-TGAGGATCGGGGTGTGTCGGGCTAGGTCAGGATGTGGGTTAGTGCCAACGTGCCTGGCCTGAG---CACGAG-GAATGGAG-A---GCA-------TTTCGG---------TGCA-ATC-CAGGATCCGAGCT-CGGTCCTGA-GC--CTTGGCCTCCCACGGATTGGCCTTGCTGCGGGG--GTCTGTTG-AC-AGTTGCA-------TAT-AGACTT--GT-CTG---------TGTGTTAACCA-GCAGAATTCA--CCTTCGGCCACCATTTAACGGTCAACTCAGAACTGGCACGGACCGGGAGAATCCGACTGTCTAATTAAAGCAAAGCATTGCGATGGCTCTAGCTGAGTGTTTACGCAATGTGATTTCTGCCCAGTGCTCTGAATGTCAACGTGAAGAAATTCAAGC-AAGCGCGGGTAAACGGCGGGAGTAACTATGACTCTCTTAAGGTAGCCAAATGCCTCGTCATCTAATTAGTGACGCGCATGAATGGATTAACGAGATTCTCA-CTGTCCCTATCTACTATCTAGCGAAACCACTGCCAAGGGAACGGGCTTGGAAAAATTAGCGGGGAAAGAAGACCCTGTTGAGCTTGACTCTAGTCTGG |
| *Tropidocephala brunnipennis* | CAG-GTGAGGGCCACGCGGAAGTCGCGCCAGATCGTACCCATATCCGCAGCAGGTCTCCAAGGTTAAGAGCCTCTAGTCGATAGAATAATGTAGGTAAGGGAAGTCGGCAAATTGGATC-CGTAACTTCGGAACAAGGATTGGCTC-TGAGGATCGGGGCGTGTCGGGCTAGGTCGGGATGTGGGTCTGCGCCAACGAGCCTGGCCTGAG---CACGAG-GAACGGAG-T---ACA-------TTTCGG---------TGTG-CTT-CGGGATCCGAGCT-CGGTCTTGC-GC--CTTGGCCTCCCACGGATCGGCCTTGCTGCGGGG--GTCTGCTG-ACAAGTTGCA-------CAC-GGACTT--GTGCTG---------TGTGTGGACGG-GTAGAATCTATCCCTTCGGCCGCCATTCAACGGTCAACTCAGAACTGGCACGGACCGGGAGAATCCGACTGTCTAATTAAAGCAAAGCATTGCGATGGCTCTAGCTGAGTGTTTACGCAATGTGATTTCTGCCCAGTGCTCTGAATGTCAACGTGAAGAAATTCAAGC-AAGCGCGGGTAAACGGCGGGAGTAACTATGACTCTCTTAAGGTAGCCAAATGCCTCGTCATCTAATTAGTGACGCGCATGAATGGATTAACGAGATTCTCA-CTGTCCCTATCTACTATCTAGCGAAACCACTGCCAAGGGAACGGGCTTGGAAAAATTAGCGGGGAAAGAAGACCCTGTTGAGCTTGACTCTAGTCTGG |
| *Tropidocephala festiva* | CAG-GTGAGGGCCACGCGGAAGTCGCGCCGGATCGTACCCATATCCGCAGCAGGTCTCCAAGGTTAAGAGCCTCTAGTCGATAGAATAATGTAGGTAAGGGAAGTCGGCAAATTGGATC-CGTAACTTCGGAACAAGGATTGGCTC-TGAGGATCGGGGCGTGTCGGGCTAGGTCGGGATGTGGGTCTGCGCCAACGAGCCTGGCCTGAG---CACGAG-GAACGGAG-T---ACA-------TTTCGG---------TGTG-CTT-CGGGATCCGAGCT-CGGTCTTGC-GC--CTTGGCCTCCCACGGATCGGCCTTGCTGCGGGG--GTCTGCTG-ACAAGTTGCA-------CAC-AGACTT--GTGCTG---------TGTGTGGACGG-GTAGAATCTATCCCTTCGGCCGCCATTCAACGGTCAACTCAGAACTGGCACGGACCGGGAGAATCCGACTGTCTAATTAAAGCAAAGCATTGCGATGGCTCTAGCTGAGTGTTTACGCAATGTGATTTCTGCCCAGTGCTCTGAATGTCAACGTGAAGAAATTCAAGC-AAGCGCGGGTAAACGGCGGGAGTAACTATGACTCTCTTAAGGTAGCCAAATGCCTCGTCATCTAATTAGTGACGCGCATGAATGGATTAACGAGATTCTCA-CTGTCCCTATCTACTATCTAGCGAAACCACTGCCAAGGGAACGGGCTTGGAAAAATTAGCGGGGAAAGAAGACCCTGTTGAGCTTGACTCTAGTCTGG |
| *Tropidocephala* sp*.* | CAG-GTGAGGGCCACGCGGAAGTCGCGCCAGATCGTACCCATATCCGCAGCAGGTCTCCAAGGTTAAGAGCCTCTAGTCGATAGAATAATGTAGGTAAGGGAAGTCGGCAAATTGGATC-CGTAACTTCGGAACAAGGATTGGCTC-TGAGGATCGGGGCGTGTCGGGCTAGGTCGGGATGTGGGTCTGCGCCAACGAGCCTGGCCTGAG---CACGAG-GAACGGAG-T---ACA-------TTTCGG---------TGTG-CTT-CGGGATCCGAGCT-CGGTCTTGC-GC--CTTGGCCTCCCACGGATCGGCCTTGCTGCGGGG--GTCTGCTG-ACAAGTTGCA-------CAC-GGACTT--GTGCTG---------TGTGTGGACGG-GTAGAATCTATCCCTTCGGCCGCCATTCAACGGTCAACTCAGAACTGGCACGGACCGGGAGAATCCGACTGTCTAATTAAAGCAAAGCATTGCGATGGCTCTAGCTGAGTGTTTACGCAATGTGATTTCTGCCCAGTGCTCTGAATGTCAACGTGAAGAAATTCAAGC-AAGCGCGGGTAAACGGCGGGAGTAACTATGACTCTCTTAAGGTAGCCAAATGCCTCGTCATCTAATTAGTGACGCGCATGAATGGATTAACGAGATTCTCA-CTGTCCCTATCTACTATCTAGCGAAACCACTGCCAAGGGAACGGGCTTGGAAAAATTAGCGGGGAAAGAAGACCCTGTTGAGCTTGACTCTAGTCTGG |
| *Ulanar muiri* | CAG-GTGAGGGCCACGCGGAAGTCGCGCCAGATCGTACCCATATCCGCAGCAGGTCTCCAAGGTTAAGAGCCTCTAGTCGATAGAATAATGTAGGTAAGGGAAGTCGGCAAATTGGATC-CGTAACTTCGGGACAAGGATTGGCTC-TGAGGATCGGGGTGTGTCGGGCTAGGTCGGGATGTGGGTTAGCGCCAACGAGCCTGGCCTGAG---CACGAG-GAATGGAG-A---GCA-------TTTCGG---------TGCT-CTC-CAGGATCCGAGTT-CGGTCCTGC-GC--CTTGGCCTCCCACGGATCGGCCTTGCTGCGGGG--GTCTGTTG-AC-AGTTGCA-------CAC-GGACTT--GT-CTG---------TGTGTTAACCA-GCAGAATTCA--CCTTCGGCCACCATTTAACGGTCAACTCAGAACTGGCACGGACCGGGAGAATCCGACTGTCTAATTAAAGCAAAGCATTGCGATGGCTCTAGCTGAGTGTTTACGCAATGTGATTTCTGCCCAGTGCTCTGAATGTCAACGTGAAGAAATTCAAGC-AAGCGCGGGTAAACGGCGGGAGTAACTATGACTCTCTTAAGGTAGCCAAATGCCTCGTCATCTAATTAGTGACGCGCATGAATGGATTAACGAGATTCTCA-CTGTCCCTATCTACTATCTAGCGAAACCACTGCCAAGGGAACGGGCTTGGAAAAATTAGCGGGGAAAGAAGACCCTGTTGAGCTTGACTCTAGTCTGG |
| *Unkanodes sapporona* | CAG-GTGAGGGCCACGCGGAAGTCGCGCCAGATCGTACCCATATCCGCAGCAGGTCTCCAAGGTTAAGAGCCTCTAGTCGATAGAATAATGTAGGTAAGGGAAGTCGGCAAATTGGATC-CGTAACTTCGGGACAAGGATTGGCTC-TGAGGATCGGGGTGTGTCGGGCTAGGTCGGGATGTGGGTCAGCGCCAACGAGCCTGGCCTGAG---CACGAG-GAATGGAA-A---GCA-------CTTCGG---------TGTG-ATC-CAGGATTCGAGCT-CGGTCCTGC-GC--CTTGGCCTCCCACGGATCGGCCTTGCTGCGGGG--GTCTGTTG-AC-AGTTGCA-------TAC-AGACTT--GT-CTG---------TGTGTCAACCA-GCAGAATTCA--CCTTCGGCCACCATTTAACGGTCAACTCAGAACTGGCACGGACTGGGAGAATCCGACTGTCTAATTAAAGCAAAGCATTGCGATGGCTCTAGCTGAGTGTTTACGCAATGTGATTTCTGCCCAGTGCTCTGAATGTCAACGTGAAGAAATTCAAGC-AAGCGCGGGTAAACGGCGGGAGTAACTATGACTCTCTTAAGGTAGCCAAATGCCTCGTCATCTAATTAGTGACGCGCATGAATGGATTAACGAGATTCTCA-CTGTCCCTATCTACTATCTAGCGAAACCACTGCCAAGGGAACGGGCTTGGAAAAATTAGCGGGGAAAGAAGACCCTGTTGAGCTTGACTCTAGTCTGG |

**Supplementary** **Table S2** The sequence alignments of 16S rDNA.

| Taxa | The sequence alignments of 16S rDNA |
| --- | --- |
| *Aoyuanus furcatus* | TTTAAATAGCTGCAGTAATTTAACTGTACAAAGGTAGCATAATAATTAGTCTTTTAATTGAGGTCTAGAATGAATGGTTTAACAAGAAAAAAACTTTATTTAATTAATTTTT---TTGAATTTAATTTTTTAGTTAAAAAGCTAAAATTTTTAAATGGGACGATAAGACCCTTTAGATCTT--TAAATTTTTATGAT---TTAATATATT--TTTGGTTAG--ATAG--GATTTTAAGT-------AAAAATT---TTTTGTTGGGGTGACAAGTAAATTTTTAAAACTTTATTCT-TTTCTAACA-TA--TATT-TATGTTTA-----GTTGATCCTAA-ATTTTGGATTTTTAAAATAAGATACCTAAGGGATAACAGCGTAATAAATTTGTAAAGTTCATATAGATAAA--TTTGTTTACGACCTCGATGTTGAATTAATTAA-AATTTTTGGAGTAGACATTAAA-ATTTTAGGTCTGTTCGACCTTTAAAATATTACA |
| *Arcofaciella verrucosa* | TTTAAATAGCTGCAGTAATTTAACTGTACAAAGGTAGCATAGTAATTAGTCTTTTAATTGAGGTCTGGAATGAATGGTTCAACAAGATACAAACTTTATTATTTAAATAGTA---TTTAATTTAATTTTTTAGTAAAAAAACTAAAATTGTTAAATGGGACGATAAGACCCTATAGATCTT--AAATTTTTT-----TTTTAAAATTATTAATTGGTAAAC--TTGT--AATGTTTTAT----CAAGATAAAT---TTTAGTTGGGGTGATTTTAAAAATTTTTAAACTTTTAATT-ATTTTAACA-TTAATATT-AATGGTTT-----AATGATCTTAA-AATTTAGATTAAAAAAATAAGATACCTTAGGGATAACAGCGTAATAAATTTGAATAGTTCATATTGATAAA--TTTGTTTACGACCTCGATGTTGAATTAATTTA-AATTTTTGAAGTAGCCATTAAAATATTTTGGTCTGTTCGACCTTTAATAAATTACA |
| *Arcofacies maculatipennis* | TTTAAATAGCTGCAGTAATTTAACTGTACAAAGGTAGCATAATAATTAGTCTTTTAATTGAGGTCTAGAATGAACGGTTTAACATAATGGAAACTTTATTATTTTAATAAAA---TTAAATTTAATTTTTTAGTTAAAAAGCTAAAATTTTTAAATGGGACGATAAGACCCTATAGATCTT--TAATTTTGTTTATTTGATTTAAAATTG--GTTGTTTAA--TTTA--CATTTATATA------AGGAAAAT---TTTTGTTGGGGTGACATTAAAATTTTTAAAACTTTTAATT-TTTTTTACA-TT--TATT-AGTGATTT-----ATAGATCTTAA-ATTTTAGACTATTAAATTAAGATACCTTAGGGATAACAGCGTGATAAATTTGGATAGTTCATATTGATAAA--TTTGTTTACGACCTCGATGTTGAATTAAATAA-AAATTTTGAAGTAGAAATTAAA-ATTTTAGGTCTGTTCGACCTTTAAATATTTACA |
| *Arcofacies strigatipennis* | TTTAAATAGCTGCAGTAATTTAACTGTACAAAGGTAGCATAATAATTAGTCTTTTAATTGGGGTCTGGTATGAACGGTTTAACAAAGTAAAATCTTTATTATTTTAATAAAA---TTGAATTTAATTTTTTAGTTAAAAAGCTAAAATTTTTAAGTGGGACGATAAGACCCTATAGATCTT--TAATTTTTATACAT----AAAATGAAT--TTAGTAAAT--TTAA--AGATTTTATA----TAATAAAAAT---TTTCGTTGGGGTGACGTTAAAAATTTTAAAACTTTTAATT-ATTTTTACA-TA--AATT-TATGGTTA-----AATGATCTTAA-ATTTTAGATTATAAAATTAAGATACCTTAGGGATAACAGCGTAATAAATTTGGATAGTGCATATTGATAAA-ATTTGTTTACGACCTCGATGTTGAATTAATTTA-AAATTTTGAAGCAGAAATCAAA-AATTTAGGTCTGTTCGACCTTTAAAAATTTACA |
| *Bambusiphaga fascia* | TTTAAATAGCTGCAGTAATTTAACTGTACAAAGGTAGCATAATAATTAGTCTTTTAATTGAGGTCTAGAATGAATGGTTTAACAAGAAAACAACTTTATTAATTTAAAAAAA--TTTTAATTTAATTTTTTAGTTAAAAAGCTAAAATTTTTAAGTGGGACGATAAGACCCTATAGATCTT--AAATTTTTTAATTT---TTTTTTTTTT--TTAGATA-A--TTAA--AATTTTAATT----ATTAAAAAAT---TTTAGTTGGGGTGACTTTAAAATTTTTAAAACTTTTATTT-ATTTTTACA-TT--TATT-TATGAATA-----TTTGATCTAAA-ATTTTTGATTGAAAGAATAAGATACCTTAGGGATAACAGCGTAATAAATTTGGATAGTTCATATTAATAAA-TTTTGTTTACGACCTCGATGTTGAATTAATTTA-AAAATTTGAAGCAGAAATTAAA-TATTTGGGTCTGTTCGACCTTTAATTAATTACA |
| *Bambusiphaga hainanensis* | TTTAAATAGCTGCAGTAATTTAACTGTACAAAGGTAGCATAATAATTAGTCTTTTAATTGAGGTCTAGAATGAATGGTTTAACAAGAAGATTACTTTATTAGTTTAATTAAG---TTTAATTTAATTTTTTAGTTAAAAAGCTAAAATAATTAATTGGGACGATAAGACCCTATAGATCTT--AAATTTTTTTTTTAAATTTAATGTATT---TAGTTAAT--TTAACTAATTTAAATT--------AAAAAT---TTTAGTTGGGGTGATTTGAAAATTTTTAAAACTTTTTAATATTATTTACA-TT--TATA-ATTGAATTTTTT-TTTGATCTTTA-TTTTTTGATTATTAGATTAAGATACCTTAGGGATAACAGCGTGATAAATTTGGATAGTTCATATTGATAAA-TTTTGATTACGACCTCGATGTTGAATTAATTTA-AAGTTTTGAAGGAGAAATTGAATATTTTAGGTCTGTTCGACCTTTAATTAATTACA |
| *Bambusiphaga luodianensis* | TTTTAATAGCTGCAGTAATTTAACTGTACAAAGGTAGCATAATAATTAGTCTTTTAATTGAGGTCTAGAATGAATGGTTCAACAAGAAGAAAACTTTATTTTTAAAATAGAA--TTTTAATTTAATTTTTTGGTTAAAAAGCTAAAATATTTAATTGGGACGATAAGACCCTATAGATCTT--AAATTTTTTGTTGA---TTAAACATTT--TTAGTAAAA--TTAA--TATTTTTTAA------TTGAAAAT---TTTAGTTGGGGTGATTTAAAAATTTTTAAAACTTTTTAAT-ATTTTTACA-TT-------TATGGATGTTTATTTTGATCTTTA-ATTTTTGATTGTTAGATTAAGATACCTTAGGGATAACAGCGTAATATATTTGGATAGTTCATATTGATAAA-TTGTGATTACGACCTCGATGTTGAATTAATTTA-AATTTTTGAAGCAGAAATTAAAAGTTTTAGGTCTGTTCGACCTTTAATTAATTACA |
| *Bambusiphaga maculata* | TTTAAATAGCTGCAGTAATTTAACTGTACAAAGGTAGCATAATAATTAGTCTTTTAATTGAGGTCTAGAATGAATGGTTTAACAAGAGAAAAACTTTATTAATTTAATTAAA--TTTTAATTTAATTTTTTAGTTAAAAAGCTAAAATTTTTAAGTGGGACGATAAGACCCTATAGATCTT--TAATTTTTAAAATT------ATTTTTTATTTGGGTAAT--TATT--AATTATAATG---TGAAAAAAAAT---TTTGGTTGGGGTGACATTAAAATTTTTAAAACTTTTAATT-ATTTTATCA-TA--AATTAAATGAATA-----TTTGATCTAAA-ATTTTTGATTAAAAGATTAAGATACCTTAGGGATAACAGCGTAATAAATTTGGATAGTTCATATTAATAAA-TTTTGTTTACGACCTCGATGTTGAATTAATTTA-AAATTTTGAAGCAGAAATTAAATTTTTTAGGTCTGTTCGACCTTTAATTAATTACA |
| *Bambusiphaga membranacea* | TTTAAATAGCTGCAGTAATTTAACTGTACAAAGGTAGCATAATAATTAGTCTTTTAATTGAGGTCTCGAATGAATGGTTTAACAAGAAGAATACTTTATTAATTTAATTAAG---TTTAATTTAATTTTTTAGTTAAAAAGCTAAAATAATTAATTGGGACGATAAGACCCTATAGATCTT---AAATTTTTTTTTTAATTATACACGTT--TGGTTAATC--TAAT--TAATTTAATT-------TTAAAAT---TTTAATTGGGGTGATTTAAAAATTTTTAAAACTTTTTAATATTATTTACA-TT--TATA-GTTGTA--TTCATTTTGATCTTTACATTTTTGATTATTAGATTAAGATACCTTAGGGATAACAGCGTAATAAATTTGGATAGTTCATATTGATAAA-TTTTGATTACGACCTCGATGTTGAATTAATTTA-AAGTTTTGAAGAAGAAGTTAAA-TATTTGGGTCTGTTCGACCTTTAATTAATTACA |
| *Bambusiphaga mirostylis* | TTTAAATAGCTGCAGTAATTTAACTGTACAAAGGTAGCATAATAATTAGTCTTTTAATTGAGGTCTGGAATGAATGGTTTAACAAGAGATAAACTTTATTAATTTAATAAAA--TTTGAATTTAGTTTTTTGGTTAAAAAACCAGAATTTTTAATTGGGACGATAAGACCCTATAGATCTT---AAATTTTTTTATTAATTTTCTATTTT--TAGTTGTTT--TTAT--AAATTTAA------AAATTAAAAT---TTTGATTGGGGTGATTTAAAAATTTTTAAAACTTTTTAAT-AAATTTACA-TT--TATA-ATTGAA--TTGA-ATTGATCTTGA-ATTTTTGATTATTAGATTAAGATACCTTAGGGATAACAGCGTAATAAATTTGGATAGTTCATATTGATAAA-TTTTGTTTACGACCTCGATGTTGAATTAATTAA-AATATTTAAAGTAGAAATTAAGTTTATTAGGTCTGTTCGACCTTTAATTGATTACA |
| *Bambusiphaga nigropunctata* | TTTAAATAGCTGCAGTAATTTAACTGTACAAAGGTAGCATAATAATTAGTCTTTTAATTGAGGTCTAGAATGAATGGTTTAACAAAAAAATAACTTTATTAATTTATTTAAA--TTTTAATTTAATTTTTTAGTTAAAAAGCTAGAATGTTTAATTGGGACGATAAGACCCTATAGATCTT--AAATTTTTTTTTGT---AAATTTTATT--TTTAGTTAA--TTAG--ATTAATTAAT----ATTAAAAAAT---TTTGGTTGGGGTGATCTAATAATTTTTAAAACTTTTTATA-AAATTTTCA-TA--TTTA-AATGACTTTTT--TTTGATCTTTA-ATTTTTGATTAAAAGATTAAGATACCTTAGGGATAACAGCGTAATAAATTTGGATAGTTCATATTGATAAATTTTTGATTGCGACCTCGATGTTGAATTAATTTA-AAGTTTTGGAGTAGAAATTAAATATTTTAGGTCTGTTCGACCTTTAGTTAATTACA |
| *Bambusiphaga taibaishana* | TTTAAATAGCTGCAGTAATTTAACTGTACAAAGGTAGCATAGTAATTAGTCTTTTAATTGAGGTCTAGAATGAATGGTTCAACAAGAAAATAACTTTATTAATTTAATAAAA--TTTTAATTTAATTTTTTAGTTAAAAAGCTAAAATTTTTAAGTGGGACGATAAGACCCTATAGATCTT--TAATTTTTT-----AAATTATTTTTTT--TTTAGATAA--ATAA--AATTTATAAT----ATTAAGAAAT---TTTGGTTGGGGTGACATTAAAATTTTAAAAACTTTTTTTT-ATATCTACA-TT--TGTT-TATGGAATT----CATGATCTTAA-ATTTTTGATTAAAAGAAAAAGATACCTTAGGGATAACAGCGTAATAAATTTGGATAGTTCATATTAATAAA-TTTTGTTTACGACCTCGATGTTGAATTAATTTA-AAAATTTGAAGCAGAAATTAAA-TATTTGGGTCTGTTCGACCTTTAATTAGTTACA |
| *Belocera sinensis* | TTTAAATAGCTGCGGTAATTTAACTGTACAAAGGTAGCATAATAATTAGTCTTTTAATTGGGGTCTAGAATGAATGGTTTAACAAAATGAAAACTTTATTAATTTAATAAAT---TTTAATTTAATTTTTTAGTTAAAAAGCTAAAATAATTAATTGGGACGATAAGACCCTGTAGATCTT--TAATTATTAAAATG---TAACTTTTTT--TTGGGGT----TTAA--GATATTAAAT----TTTTAATAAT---TTTAATTGGGGTGATGTTAAAATTTTTAAAACTTTTAATT-ATTTATTCA-CA--TAAA-TAGGTGTATTGATTATGATCCTGA-TTTTTAGATTGAAAGATTAAGATACCTTAGGGATAACAGCGTTATATATTTGGATAGTACACATTGATAAA--TATGTTTACGACCTCGATGTTGAATTAATTAATAAATTTTAAAGTAGAAGTTAAA-AGTTTAGGTCTGTTCGACCTTTAATTAATTACG |
| *Carinofrons maculatipennis* | TTTAAATAGCTGCAGTAATTTAACTGTACAAAGGTAGCATAATAATTAGTCTTTTAATTGAGGTCTAGAATGAATGGTTTAACAAAATAAAAACTTTATTATTTTAATTCAA---TTTAATTTAATTTTTTAGTTAAAAAGCTAGAATAATTAGATGGGACGATAAGACCCTATAGATCTT--TAATTTTTTTTTTA---TTAAAATTTT--TTGGTTTTA--TTAA--GGTTTAATTT-----AATTAAAAT---TTCAGTTGGGGTGATTTAAAAAATTT-AAAACTTTTTAAT-ATTTTTTCA-TA--GATT-AATGTTTA-----TTTGATCTTAA-AATTTAGATTATTAGATTAAGATACCTTAGGGATAACAGCGTAATAAATTTGAATAGTTCATATTGTTAAA--TTTGTTTACGACCTCGATGTTGAATTAATTTT-AAAATTTGAAGCAGAAATTAAAGTTTTTAGGTCTGTTCGACCTTTAATTAATTACA |
| *Cemopsis griphus* | TTTAAATAGCTGCAGTATTTTAACTGTACAAAGGTAGCATAATAATTAGTCTTTTAATTGAGGTCTAGAATGAATGGTTTAACAAGAAAAAATCTTTATTTAATAAATTAAA---TTAAATTTTATTTTTTAGTTAAAAAGCTAAAATTTTTAAATGGGACGATAAGACCCTATAGATCTT--TAAATTTTTAAAAT---TAAATATATT-TTGGGAAAAA--ATAG--GGTTTTAATT-------AAAAATT---TTTTGTTGGGGTGACAGTTAAAATTTTAAACCTTTAATTA-TTTAAAACA-TG--TATT-AATGATTAA----TATGATCCTAA-ATTTTGGATTTTAAAAATGAGATACCTTAGGGATAACAGCGTAATAAATTTGTATAGTTCATATAGATAAA--TTTGTTTACGACCTCGATGTTGAATTAATAAA-AATTTTTGGAGCATAAATTAAA-ATTTTAGGTCTGTTCGACCTTTAAAATATTACA |
| *Cemus nigromaculosus* | TTTAAATAGCTGCAGTATTTTAACTGTACAAAGGTAGCATAATAAATAGTCTTTTAATTGAGGTCTAGAATGAAGGGTTTAACAAGAAAAATACTTTATT---TGATTTAAATATTTGAATTTAATTTTTTAGTTAAAAAGCTAAAATTTTTAAATGGGACGATAAGACCCTATAGATCTT--TAAATTTTTAATAT---TAAATATATT--TTAGTTAAT--TTAA--GATTTTAGAT-------AAAAATT---TTTTGTTGGGGTGACAAATAAAATTTTAAGACTTTATTTC-TTTGAAACA-TA--TATT-TATGGTTAA----GATGATCCTAATTTTTTGGATTATAAGGATAAGATACCTTAGGGATAACAGCGTTATAAATTTGTATAGTTCACATAAATAAA---TTTTTTACGACCTCGATGTTGAATTAATTAA-AATTCTTGGAGCAGAAGTTAAG-ATTTTAGGTCTGTTCGACCTTTAAAATATTACA |
| *Cemus punctatus* | TTTAAATAGCTGCAGTATTTTAACTGTACAAAGGTAGCATAGTAAGTAGTCTTTTAATTGAGGTCTAGAATGAATGGTTTAACAAGAAAAATACTTTATTGAATTGATAATA---TTGAATTTAATTTTTTAGTTAAAAAGCTTAAATTTTTAAATGGGACGATAAGACCCTATAGATCTT--AAAATTTTAATAAT---TAAACATATT--TAGGTTAAT--TTAA--GGTTTTAAAT-------GAAAATT---TTTTGTTGGGGTGACAAATAAAATTTTAAAACTTTATTTT-TTAAATACA-TA--TATT-TATGATTAAA---AATGATCCTAA-TATTTGGATTATAAGAATAAGATACCTTAGGGATAACAGCGTAATAAATTTGTATAGTTCATATAAATAAA--TTTGTTTACGACCTCGATGTTGAATTAATTAA-AATTCTTGGAGCAGAAATTAAG-GTTTTAGGTCTGTTCGACCTTTAAAATATTACA |
| *Cemus sauteri* | TTTAAATAGCTGCAGTATTTTAACTGTACAAAGGTAGCATAATAAATAGTCTTTTAATTGAGGTCTAGAATGAAGGGTTTAACAAGAAAAATACTTTATTTGATTTAAATAT---TTGAATTTAATTTTTTAGTTAAAAAGCTAAAATTTTTAAATGGGACGATAAGACCCTATAGATCTT--TAAATTTTTAATAT---TAAATATATT--TTAGTTAAT--TTAA--GATTTTAGAT-------AAAAATT---TTTTGTTGGGGTGACAAATAAAATTTTAAGACTTTATTTT-TTTGAAACA-TA--TATT-TATGGTTAA----GATGATCCTAA-TTTTTGGATTATAAGGATAAGATACCTTAGGGATAACAGCGTTATAAATTTGTATAGTTCACATAAATAAA---TTTTTTACGACCTCGATGTTGAATTAATTAA-AATTCTTGGAGCAGAAGTTAAG-ATTTTAGGTCTGTTCGACCTTTAAAATATTACA |
| *Cixius scrupeus* | CTTAAATGGCCGCGGTATTTTAACTGTGCTAAGGTAGCATAATAATTAGTCTCTTAATTAGGGTCTTGAATGAATGGTTGGACGAGAAAACAACTTTATTAAAAAAATTTTT--TCTTAATTTAATTTTTTTGTTAAAAAGCTTAAATTTTTAAATGGGACGATAAGACCCTGTAGATCTTAACAAATTATT--------TAGGTTCATT--TTTTTGTTG--TATT--AATTTGTTTC----TTAATTATTTTTGTTTTATTGGGGTGATAAATAAATTTTTTAAACTTTTTTTA-AATTAAACA-TT--TATA-TATGGCAAT----TTTGATCTTTACATTTTTGATTAAAAGAAAAAGATACCTCAGGGATAACAGCGTTATATATTTGGAGAGTTCTTATTGATAAA--TATGTTTGCGACCTCGATGTTGGATTAAAATT—ACTTGTGGAGCAGAATATACAAAGTTTAGGTCTGTTCGACCTTTAAAATTTTACA |
| *Criomorphus niger* | TTTAAATAGCTGCAGTATTTTAACTGTACAAAGGTAGCGTAATAATTAGTCTTTTAATTGAGGTCTAGAATGAAGGGTTTGACAAGAAAATACCTTTATTAAATTAACTTAA----TGAATTTTATTTTTTAGTTAAAAAGCTAAAATTTTTAAATGGGACGATAAGACCCTATAGATCTT--TAAATTTTTATTAG--ATAAAATTATT--TAAGTTAAA--TTAC--ATTGTATTTT------TAAAAATT---TTTTGTTGGGGTGACAAATAAATTTTTAAAACTTTAATAC-TTTATTCCA-TA--AATT-GATGACTC-----AATGAACCTAA-ATTTAGGGTAATTAAAATAAGATACCTTAGGGATAACAGCGTAATAAATTTGTATAGTTCAAATAGATAAA--TTTGTTTACGACCTCGATGTTGAATTAATCTT-AAGTTTTGGGGCAGCAATTAAA-ATTTTAGGTCTGTTCGACCTTTAAAAGATTACA |
| *Distantinus melichari* | TTTAAATAGCTGCAGTATTTTAACTGTACAAAGGTAGCATAGTAATTAGTCTTTTAATTGAGGTCTTGAATGAATGGTTTAACAAGAAAATTACTTTATTACATTAATTTAA----TTAATTTTATTTTTTAGTTAAAAAGCTAAAATTATTAAATGGGACGATAAGACCCTATAGATCTT--TAAATTTTTATAAA---TTAATATATT--TTAGTTA-A--ATTA--ATTTCAGTTT----ACTTAAAATT---TTTTATTGGGGTGATATACAAAATTTTTTAGCTTTGTAAT-ATATCTACA-TG--AATC-AATGA---TTTT-ATTGAACCTAA-ATTTTAGGTTACTAAAATAAGATACCTTAGGGATAACAGCGTAATAAGTTTGTATAGTTCATATAGATAAA--CTTTTTTACGACCTCGATGTTGAATTAATTTA-AAATTTTGGAGCAGAAGATGAATATTTTGGGTCTGTTCGACCTTTAAAAAATTACA |
| *Ecdelphax cervina* | TTTAAATAGCTGCAGTATTTTAACTGTACAAAGGTAGCATAGTAAATAGTCTTTTAATTGAGGTCTGGAATGAACGGTTTTACAAGAAAACCACTTTATTTAATTGATTGGG---TTGAATTTAATTTTTTAGTTAAAAAGCTAAAATTTTTAAATGGGACGATAAGACCCTATAGATCTT--TAAATTTTTAAGAT---TAAATACATT--TTAGGTTAT--ATAG--GGTTTTTAAT-------AAAAATT---TTTTGTTGGGGTGACAAATAAAATTTTAATACTTTATTTC-TTAAATACA-TA--AATT-TATGAATAAA---AATGATCCTAA-ATTTTGGAATACAAAAATAAGATACCTTAGGGATAACAGCGTAATAAATTTGTACAGTTCATATAAATAAA--TTTGTTTACGACCTCGATGTTGAATTAATTAA-AATTCTTGGAGCAGAAGTTAAG-ATTTTAGGTCTGTTCGACCTTTAAAATATTACA |
| *Ecdelphax dentata* | TTTAAATAGCTGCAGTATTTTAACTGTACAAAGGTAGCATAGTAAATAGTCTTTTAATTGAGGTCTGGAATGAACGGTTTTACAAGAAAAACACTTTATTTAATTGATTGGG---TTGAATTTAATTTTTTAGTTAAAAAGCTAAAATTTTTAAATGGGACGATAAGACCCTATAGATCTT--TAAATTTTTAAGAT---TAAATACATT--TTAGGTTAT--ATAG--GGTTTTTAAT-------AAAAATT---TTTTGTTGGGGTGACAAATAAAATTTTAATACTTTATTTC-TTAAATACA-TA--AATC-TATGAGTAAA---GATGATCCTAA-ATTTTGGAATACAAAAATAAGATACCTTAGGGATAACAGCGTAATAAATTTGTACAGTTCATATAAATAAG--TTTGTTTACGACCTCGATGTTGAATTAATTAA-AATTCTTGGAGCAGAAGTTAAG-ATTTTAGGTCTGTTCGACCTTTAAAATATTACA |
| *Elachodelphax metcalfi* | TTTAAATAGCTGCAGTATTTTAACTGTACAAAGGTAGCGTAGTAATTAGTCTTTTAATTGAGGTCTTGAATGAAAGGTTTGACAAGAGGAAAACTTTATTAAATTAATAATG----TAAATTTTATTTTTTAGTTAAAAAGCTAAAATTTTTAAATGGGACGATAAGACCCTATAGATCTT--TAATTTTTTGTTAT---TTAAATCAAT--TAATGATTA--TTTG--AGGTTTATTT-----ATAAAAATT---TTTTGTTGGGGTGACGGTTAAAATATTAAAACTTTAATGT-TTATATACATTT--AATT-AATGAATTT----TATGAACCCAA-CTTTTGGGTTGTTAAAATAAGATACCTTAGGGATAACAGCGTAATAAATTTGTATAGTTCTTATAGATAAA--TTTGTTTACGACCTCGATGTTGAATTAATTTT-AAGTTTTGGGGCAGAAATCAAA-ATTTTAGGTCTGTTCGACCTTTAAAAAATTACA |
| *Epeurysa distincta* | TTTAAATAGCTGCAGTAATTTAACTGTACAAAGGTAGCATAATAATTAGTCTTTTAATTGAGGTCTGGAATGAATGGATTAACAAAATAATAACTTTATTAGGTTAAATAAA--TTTTAACTTTCTTTTTTAGTTAAAAAGCTAAAATTTTTAAATGGGACGATAAGACCCTATAGATCTT--TAATTTTTTAAAAT---TAATTTTTTT--TTAGATTGA--TTAA--AAATTTAAAT-----TTAAAAAAT---TTTAGTTGGGGTGACTAAAAAAATTTTAAAACTTTTTTTA-ATTTTATCA-TA--AATT-AATGTTTTT----TTAGATCTTAATTATTTAGATTTTAAAAATAAGATACCTTAGGGATAACAGCGTAG-AAATTTGGATAGTTCATATTGATAAA--TTTATTTACGACCTCGATGTTGAATTAATTAT-AAATTTTAAAGCAGAAATTAAT-AATTTAGGTCTGTTCGACCTTTAATTAATTACA |
| *Epeurysa infumata* | TTTAAATAGCTGCAGTAATTTAACTGTACAAAGGTAGCATAATAATTAGTCTTTTAATTGAGGTCTGGAATGAATGGATTAACAAAATAATAACTTTATTAGGTTAAGTAAA--TTTTAATTTTATCTTTTAGTTAAAAAGCTAAAATTTTTAAATGGGACGATAAGACCCTATAGATCTT--TAATTTTTTAGGGT---TAAGGGGTTT--TTAGATGAA--TTAA--AATTTTAAGT-----TTAAAAAAT---TTTAGTTGGGGTGACTAAAAAATTTTTAAAACTTTTTTTA-ATTTTATCA-TA--AATT-AATGTCTTT----TTAGATCCTAATTATTTAGATTTTAAAAATAAGATACCTTAGGGATAACAGCGTAG-AAATTTGGATAGTTCATATTGATAAA--TTTATTTACGACCTCGATGTTGAATTAATTAT-AAATTTTAAAGCAGAAATTAAT-AATTTAGGTCTGTTCGACCTTTAATTAATTACA |
| *Epeurysa nawaii* | TTTAAATAGCTGCAGTAATTTAACTGTACAAAGGTAGCATAATAATTAGTCTTTTAATTGAGGTCTGGGATGAATGGATTAACAAAATAACAACTTTATTAGGTTAAATAAA--TTTTAATTTTCTTTTTTAGTTAAAAAGCTAAAATATTTAAATGGGACGATAAGACCCTATAGATCTT--TAATTTTTTAGGGT---TAATTTTTTT--TTAGATAAA--TTAA--AATCTTAAGT-----TTAAAAAAT---TTTAGTTGGGGTGACTAAAAAAATTTTAAAACTTTTTTTA-ATTTTATCA-TA--AATT-AATGTATTT----TTAGATCCTAATTATTTAGATTTTAAAAATAAGATACCTTAGGGATAACAGCGTAG-AAATTTGGATAGTTCATATTGATAAA--TTTATTTACGACCTCGATGTTGAATTAATTAT-AAATTTTAAAGCAGAAATTAAT-AATTTAGGTCTGTTCGACCTTTAATTAATTACA |
| *Eurybregma nigrolineata* | TTTAAATAGCTGCAGTATCTTAACTGTACAAAGGTAGCATAATAATTAGTCTTTTAATTGGGGTCTAGTATGAAGGGTTTGACAAGAAAAAATCTTTATTAATTTAATTTAA----TGAATTTTATCTTTTCGTTAAAAAGCTAAAATTTTAAAATGGGACGATAAGACCCTATAGATCTT--TAAATTTTT---GTTTATATAAGTATT--TAAGTTATA--TTTG--AATTTTATTT-----ATAAAAATT---TTTTGTTGGGGTGACAAATAAATTTTTAAAACTTTAATAC-TTAAATCCA-TT--AATT-GATGTATTT----TATGAACCTAA-ATTTGGGGTTGTTAGAATAAGATACCTTAGGGATAACAGCGTAATAAATTTGTATAGTTCATATAGATAAA--TTTGTTTACGACCTCGATGTTGAATTAATCTT-AAGTTTTGGGGCAGAAAATAAA-ATTTTAGGTCTGTTCGACCTTTAAAAGGTTACA |
| *Falcotoya lyraeformis* | TTTAAATAGCTGCAGTATTTTAACTGTACAAAGGTAGCGTAGTAATTAGTCTTTTAATTGAGGTCTTGTATGAATGGTTTGACAAGAAATATACTTTATTTGGTTAATTTAA----TAAATTTTATTTTTTAGTTAAAAAGCTAAAATATTTAAATGGGACGATAAGACCCTATAGATCTT--TAAATTTTTTTTTA---TATAGTTATT--TAAGTTG-A--TTAA--ATTTTTGTAA----TTAAAAAATT---TTTTGTTGGGGTGATATACAAAATTTTACAACTTTGTAAT-TTTCACACA-TA--AATT-AATGAT--TTCA-GTTGAACCTAA-ATTTAGGTTTGTTAAAATAAGATACCTTAGGGATAACAGCGCAATAAATTTGTATAGTTCATATAGATAGA--TTTGTTTGCGACCTCGATGTTGAATTAATTTA-AAGTTTTGGGGCAGAAAATAAA-ATTTTAGGTCTGTTCGACCTTTAAAATGTTACA |
| *Fangdelphax gongshanensis* | TTTAAATAGCTGCAGTATATTAACTGTACAAAGGTAGCGTAGTAATTAGTCTTTTAATTGAGGTCTTGTATGAATGGTTTGACAAGAAGAAGACTTTATTAAGTTAATTGTA----TAAATTTTATTTTTTAGTTAAAAAGCTAAAATTTTTAAATGGGACGATAAGACCCTATAGATCTT--TAAATTTTTGTTAT---TTAAATCGAT--TTAAGGTTA--TTTA--AATTTTATTT----GAAAAAAATT---TTTTGTTGGGGTGACAGATAAAATTTTAAAACTTTAATAT-TTATTTACA-TA--GATC-TATGGATA-----TTTGAACCCTA-ACTTTGGGTTATTAAAATTAGATACCTTAGGGATAACAGCGTAATAAATTTGAATAGTTCTTATAGATAAA--TTTGTTTACGACCTCGATGTTGAATTAATCTA-AAGTTTTGGGGCAGAAATTAGA-ATTTTAGGTCTGTTCGACCTTTAAAAGATTACA |
| Gen. nov*.* 1 | TTTAAATAGCTGCAGAATTTTGACTGTACAAAGGTAGCATAATAATTAGTCTTTTAATTGAGGTCTAGAATGAATGGTTTAACAAAATAAAAACTTTATTATTTTAATAAAAATTTTTAAATTAATTTTTTAGTTAAAAAGCTAAAATTTTTAATTGGGACGATAAGACCCTTTAGATCTT---AAATTTTGAGTAT---ATAATTTTTT--TTAGAAATT--ATTA--ATTTTTATAT-----TAATAAAAT---TTTCGTTGGGGTGATGACAAAATTCTTAAAACTTTTGTTT-AATTAAACA-TT--AATT-GATGATATTT---TGTGATCTTGA-ATTTTAGATTTTAAGATTAAGATACCTTAGGGATAACAGCGTAATAAATTTAGATAGTTCATATTGATAAA--TTTGTTTACGACCTCGATGTTGAATTAATTTA-AAGTTTTAAAGCAGAAATTAAA-AATTTGGGTCTGTTCGACCTTTAAAAAATTACA |
| *Hadeodelphax afurculus* | TTTAAATAGCTGCAGTATATTAACTGTACAAAGGTAGCATAATAATTAGTCTTTTAATTGAGGTCTGGAATGAAGGGTTTGACAAGAAAATAACTTTATTAATTTAATTTTT---TTAAAATTTAAGTTTTAGTTAAAAAGCTAAAATTTTTAAGTGGGACGATAAGACCCTATAGATCTT--TAAATTTTTTATTTATGTAAATG---T--TCAAGAGAG--GTTA--AATTTTATTT-----AAAAAAATT---TTTTGTTGGGGTGACGTTTAAATTTTTAAAACTTTAAAAT-TTATTATCA-TT--TATA-AATGAAACA----TTAGAACTTGA-ATTTTAGGTTGTTAAAATAAGATACCTTAGGGATAACAGCGTTATAAATTTGTAAAGTTCTTATAGATAAA--TTTGTTTGCGACCTCGATGTTGAATTAATTTA-AATTTTTGGAGTAGAAATTAAA-TTTTTAGGTCTGTTCGACCTTTAAAAAATTACA |
| *Harmalia aculeatus* | TTTAAATGGCTGCAGTATTTTAACTGTACAAAGGTAGCCTAGTAATTAGTCTTTTAATTGGGGTCTTGAATGAATGGTTTAACAAGAAATTAACTTTATTAGGTTAAATTTA----TGAATTTAATTTTTTAGTTAAAAACCTAAAATATTTAAGTGGGACGATAAGACCCTATAGATCCC--TAATTTTTTTGTTA---ATAAAATATT--TAAGTGAAT--ATTT--ATTTAGTTA-----GTAAGAAATT---TTTTGTTGGGGTTACATTAAAATTTTTAAGACTTTTAAAC-TATTAAACA-TT--TATA-AATGAATA-----ACTGAACCCAA-TTTTTGGGTTGTTAAAATAAGATACCTTAGGGATAACAGCGTAATAGATTTGTATAGTTCTTATAGATAAA--TTTGTTTACGACCTCGATGTTGAATTAACATA-AAGTTTTGGAGCAGAAGATAAA-AGC----------------------------CA |
| *Harmalia gayasana* | TTTAAATGGCTGCAGTATTTTAACTGTACAAAGGTAGCGTAGTAATTAGTCTTTTAATTGGGGTCTTGAATGAATGGTTTAACAAGAAATTAACTTTATTAAGTTAAATTTA----TGAATTTAATTTTTTAGTTAAAAAGCTAAAATATTTAAGTGGGACGATAAGACCCTATAGATCTT--TAATTTTTTTACTA---ATAAAATATT--TAAGTGAAG--ATTC--ATTTTATTAG-----TAAGAAATT---TTTTGTTGGGGTTACATTAAAATTTTTAAGACTTTTAAAT-TATTTTACA-TT--TATA-AATGAATA-----ACTGAACCTAA-CTTTTAGGTTGTTAAAATAAGATACCTTAGGGATAACAGCGTAATAGATTTGTATAGTTCTTATAAATAAA--TTTGTTTACGACCTCGATGTTGAATTAACTTA-AAGTTTTGGAGCAGAAGATAAA-ATTTTAGGTCTGTTCGACCTTTAAAAGGTTACA |
| *Harmalia heitensis* | TTTAAATGGCTGCAGTATTTTAACTGTACAAAGGTAGCGTAGTAATTAGTCTTTTAATTGGGGTCTTGAATGAATGGTTTAACAAGAAATTAACTTTATTAAGTTAAATTTA----TGAATTTAATTTTTTAGTTAAAAAGCTAAAATATTTAAGTGGGACGATAAGACCCTATAGATCTT--TAATTTTTTTACTA---ATAAAATATT--TAAGTGAAG--ATTC--A-TTTAATTA----GTAAGAAATT---TTTTGTTGGGGTTACATTAAAATTTTTAAGACTTTTAAAT-TATTTTACA-TT--TATA-AATGAATA-----ACTGAACCTAA-CTTTTAGGTTGTTAAAATAAGATACCTTAGGGATAACAGCGTAATAGATTTGTATAGTTCTTATAAATAAA--TTTGTTTACGACCTCGATGTTGAATTAACTTA-AAGTTTTGGAGCAGAGGATAAA-ATTTTAGGTCTGTTCGACCTTTAAAAGGTTACA |
| *Harmalia sirokata* | TTTAAATGGCTGCAGTATTTTAACTGTACAAAGGTAGCGTAGTAATTAGTCTTTTAATTGGGGTCTTGAATGAATGGTTTAACAAGAAATTAACTTTATTAAGTTAAATTTA----TGAATTTAATTTTTTAGTTAAAAAGCTAAAATATTTAAGTGGGACGATAAGACCCTATAGATCTT--TAATTTTTTTACTA---ATAAAATATT--TAAGTGAAG--ATTC--ATTTAATTAG-----TAAGAAATT---TTTTGTTGGGGTTACATTAAAATTTTTAAGACTTTTAAAT-TATTTTACA-TT--TATA-AATGAATA-----ACTGAACCTAA-CTTTTAGGTTGTTAAAATAAGATACCTTAGGGATAACAGCGTAATAGATTTGTATAGTTCTTATAAATAAA--TTTGTTTACGACCTCGATGTTGAATTAACTTA-AAGTTTTGGAGCAGAGGATAAA-ATTTTAGGTCTGTTCGACCTTTAAAAGGTTACA |
| *Himeunka tateyamaella* | TTTAAATAGCTGCAGTATTTTAACTGTACAAAGGTAGCGTAGTAATTAGTCTTTTAATTGGGGTCTTGAATGAATGGTTTGACAAGAAAAGCGCTTTATTGTGATAATTAAA----TAAATTTTATTTTTTAGTTAAAAAGCTAAAATTATTAAATGGGACGATAAGACCCTATAGATCTT--AAATTTTTTTTTAA---AATATACATT--TTAGTGTAA--TTGA--TTTTTTTTAA------CAAAAATT---TTTTGTTGGGGTGACATACAAAATTTAAAAACTTTGTAAT-AGATTTACA-TA--CGTA-AATGTTTA-----ATTGAACCTAA-ATTTTAGGTTGTTAAAATAAGATACCTTAGGGATAACAGCGTAATAAATTTGTAAAGTTCATATAGATAAA--TTTGTTTACGACCTCGATGTTGAATTAATTAA-AAGTTTTGGAGCAGAATATAAA-ATTTTGGGTCTGTTCGACCTTTAAAAAATTACA |
| *Ishiharodelphax matsuyamensis* | TTTAAATAGCTGCAGTATTTTAACTGTACAAAGGTAGCATAATAATTAGTCTTTTAATTGAGGTCTTGAATGAACGGTCTGACAAGAAAAAAACTTTATTAGGTTAATTTTA----TAAATTTTATTTTTTAGTTAAAAAGCTAAAATATTTAATTGGGACGATAAGACCCTATAGATCTT--AAAATTTTTTTTTA---AAAAATTATT--TTAGTT--A--ATTA--AATTTTTTTG----AGTAAAAATT---TTTCGTTGGGGTGATGTGTAAAATTTTATAACTTTTTAAA-TTTAATACA-TA--AATT-GATGG---TTTAAATTGAACCTAA-ATTTAGGTTTGTTAAAATAAGATACCTTAGGGATAACAGCACAATAAATTTGTATAGTTCACATAGATAAA--TTTGTTTGTGACCTCGATGTTGAATTAATTTA-AAGTTTTGGGGCAGAAAATAAA-ATTTTAGGTCTGTTCGACCTTTAAAATATTACA |
| *Javesella obscurella* | CTTAAATAGCTGCAGTATTTTAACTGTACAAAGGTAGCGTAATAATTAGTCTTTTAATTGGGGTCTAGAATGAAAGGTTTGACAAGAAAATAACTTTATTAATTTAATTAAA----TGAATTTTATTTTTTAGTTAAAAAGCTAAAATTTTAAAATGGGACGATAAGACCCTATAGATCTT--TAAATTTTTATTTA--ATAGGGGTGTT--TATGGTTTA--ATTA--ATTTTTATTT-----CAAAAAATT---TTTTGTTGGGGTGACAATTAAATTTTTAGAACTTTGATAT-TTAATTACA-TA--AATG-CATGTCTT-----ATTGAACCTGA-ATTTGGGGTTGTTAAAATAAGATACCTTAGGGATAACAGCGTAATAAATTTGTATAGTTCATATAGATAAA--TTTGTTTACGACCTCGATGTTGAATTAACTTA-AAGTTTTGGGGCAGAAATTAAA-ATTTTAGGTCTGTTCGACCTTTAAAGGGTTACA |
| *Javesella* sp. | CTTAAATAGCTGCAGTATTTTAACTGTACAAAGGTAGCGTAATAATTAGTCTTTTAATTGGGGTCTAGAATGAAAGGTTTGACAAGAAAATGACTTTTTTAATTTAATTAAG----TGAATTTTATTTTTTAGTTAAAAAGCTAAAATTTTTAAATGGGACGATAAGACCCTATAGATCTT--TAAATTTTTACTTT--ATAAAGGTGTT--TATGGTTGA--ATCA--AATTTTATTT-----TTAAAAATT---TTTTGTTGGGGTGACAATTAAATTTTTATAACTTTAATAT-TTAATTACA-TA--AATC-TATGTATTG----ATTGAACCCAA-ATTTGGGGTTGTTAAAATAAGATACCTTAGGGATAACAGCGTAATAAATTTGTATAGTTCATATAGATAAA--TTTGTTTACGACCTCGATGTTGAATTAACTTA-AAGTTTTGGGGCAGAAATTAAA-ATTTTGGGTCTGTTCGACCATTAAAAGGTTACA |
| *Kelisia xiphura* | TTTAAATAGCTGCAGTATTTTAACTGTACAAAGGTAGCATAGTAATTAGTCTTTTAATTGAGGTCTAGAATGAATGGGTTGTCAAGAAAATAACTTTATTTTTAAAATTTAA-TATTTAATTTTACTTTTAAGTTAAAAGGCTTAAATTTTTGAGTGGGACGATAAGACCCTATAGATCTT--AAAATTTTTAATTT-----TTCTTATT--TTTTGGGAG--TTTA--AAATTTAATT----ATTGAAAATT---TTTCGTTGGGGTGATGTTAAAATTTTTCAAACTTTTAAAA-TTTTAAACA-TT--TTTA-AATGGATT-----TTTGATCTTAA-ATTTTTGATTATTAGATTAAGATACCTTAGGGATAACAGCACAATAAATTTGAATAGTTCAAATTGTTAAA--TTTGTTTGTGACCTCGATGTTGAATTAATTTA-AGATATTGAAGCAGAAATTAAA-TGTCTGGGTCTGTTCGACCTTTAAATAATTACA |
| *Kusnezoviella dimidiatifrons* | TTTAAATAGCTGCAGTATTTTAACTGTACAAAGGTAGCGTAGTAATTAGTCTTTTAAATGAGGTCTCGTATGAATGGTTTGACAAGAGGAAAACTTTATTAAATTAATTATA----TAAATTTTATTTTTTAGTTAAAAAGCTAAAATTTTTAAATGGGACGATAAGACCCTATAGATCTT--TAAATTTTTATTAT---TTAAACTAAT--TAAAGGTTA--TTTA--AATTTTATTT-----ATGAAAATT---TTTTGTTGGGGTGACAGATAAAATTATAAAACTTTAATTT-TTATTTACA-TT--TATT-AATGGGCTT----CATGAACCCTA-AATTTGGGTTGCTAAAATAAGATACCTTAGGGATAACAGCGTAATAAATTTGTAAAGTTCTTATAGATAAA--TTTGTTTACGACCTCGATGTTGAATTAATTTA-AAGTTTTGGAGTAGAAATTAAA-ATTTTAGGTCTGTTCGACCTTTAAAAGATTACA |
| *Laodelphax striatellus* | TTTAAATAGCTGCAGTATTTTAACTGTACAAAGGTAGCGTAGTAATTAGTCTTTTAATTGAGGTCTCGTATGAATGGTTTGACAAGAAGGGGACTTTATTGAGTTAATTATA----TAAATTTTATTTTTTAGTTAAAAAGCTAAAATTTTTAAATGGGACGATAAGACCCTTTAGATCTT--TAAATTTTTACTAT---TTAAATTAAT--TTAAGGTTA--TTTA--AATTTTATTT----GTAAAAAATT---TTTTGTTGGGGAGACAGATAAAATTTTAAAACTTTAATAT-TTTTTTACA-TA--GATT-TATGGATT-----TTTGAACCTTA-ATTTAGGGTTATTAAAATTAGATACCTTAGGGATAACAGCGTAATAAATTTGTATAGTTCTTATAGATAAA--TTTGTTTACGACCTCGATGTTGAATTAATCTA-AAGTTTTGGGGCAGAAATTAGA-ATTTTAGGTCTGTTCGACCTTTAAAAGATTACA |
| *Lauriana senticosa* | TTTAAATAGCTGCAGTAATTTAACTGTACAAAGGTAGCATAATAATTAGTCTTTTAATTGAGGTCTAGAATGAATGGTTTAACAAGAGAAAATCTTTATTAATTTAAAAATA--TTTAAATTTAATTTTTTAGTTAAAAAGCTAAAATTTTTAAGTGGGACGATAAGACCCTATAGATCTT--AAATTTTTTTTTAT-----AAACTTTT--TTAGTTGAT--TTGA--AATTAAATTA----TTAAAAAAAT---TTTTGTTGGGGTGACATTAAAATTTTTAAAACTTTTAATT-ATTTAATCA-TT--TATA-AATGAATA-----TTTGATCCTAA-AATATGGATTGTAAGATAAAGATACCTTAGGGATAACAGCGTAATAAATTTGAATAGTTCATATTGATAAA--TTTGTTTACGACCTCGATGTTGAATTAATTTA-AGGTTTTAAAGTAGAAATTAAAAATTTTAGGTCTGTTCGACCTTTAACTAGTTACA |
| *Malaxa semifusca* | TTTAAATAGCTGCAGTAATTTAACTGTACAAAGGTAGCATAATAATTAGTCTTTTAATTGAGGTCTAGAATGAATGGTTTAACAAGAGAAAATCTTTATTAATTCAAAAATA--TTTAAATTTAATTTTTTAGTTAAAAAGCTAAAATTTTTAATTGGGACGATAAGACCCTATAGATCTT--TAATTTTTTTTTTA----CAATTCTTT--TTTGATGGT--TTTG--GATTTAATAG---TTAACAAAAAT---TTTTGTTGGGGTGACATTAAAATTTTTAAAACTTTTAATA-ATTTTAACA-TT--TATA-AGTGTAAAA----TTTGATCTCAA-AATTTGGATTTAAAGATAAAGATACCTTAGGGATAACAGCGTAATAAATTTGAATAGTTCATATTGATAAA--TTTGTTTACGACCTCGATGTTGAATTAATTTA-AGGTTTTAAAGTAGAAATTAAATATTTTAGGTCTGTTCGACCCTTAATAAATTACA |
| *Malaxella flava* | TTTAAATGGCTGCAGTAATTTAACTGTACAAAGGTAGCATAATAATTAGTCTTTTAATTGAGGTCTGGAATGAATGGTTCAACAAGAGAATAACTTTATTAATTT--TTGATAATTTGAATTTAATTTTTTGGTTAAAAAGCTAAAATTTTTAATTGGGACGATAAGACCCTATAGATCTT----ATTTTTT--------TAAATTAATTTTTTATATAAG--TTTA--TTTTTTAAAATAAAATAAAAAAAA---ATTCGTTGGGGTGACGTTGAAAACTTTAGAACTTTTAAAA-ATTTTGTCA-TT--GATT-AATGATTA-----ATTGATCTTTA-ATTTAGGATTAGTAGATTAAGATACCTTAGGGATAACAGCGTAATAAATTTGGATAGTTCATATTGATAAA--TTTGTTTACGACCTCGATGTTGAATTAATTTA-AAATTTTGGAGTAGAAATTAAA-AATTTGGGTCTGTTTGACCTTTAATTAATTACA |
| *Malaxella macracantha* | TTTAAATGGCTGCAGTAATTTAACTGTACAAAGGTAGCATAATAATTAGTCTTTTAATTGAGGTCTGGAATGAATGGTTTAACAAGAAAATAGCTTTATTAATTTTTATAAA--TTTGAATTTAATTTTTTAGTTAAAAAGCTAAAATTTTTAACTGGGACGATAAGACCCTATAGATCTT----ATTTTTTTTAAA---ATGATTTTTTATTTTGGATATTATTTA--AAAATAAGAG-----ATAAAAAAA---ATTCGTTGGGGTGACGTTAAAAATTTTAAAACTTTTGAAA-ATTTTTTCA-TT--TATT-GATGACTA-----ATTGATCTTTA-ATTTAGGATTAATAGATTAAGATACCTTAGGGATAACAGCGTAATAAATTTGGATAGTTCATATTGATAAA--TTTGTTTACGACCTCGATGTTGAATTAATTTA-AAATTTTGGAGTAGAAGTTAAA-AGTTTAGGTCTGTTCGACCTTTAATTAATTACA |
| *Malaxella tetracantha* | TTTAAATGGCTGCAGTAATTTAACTGTACAAAGGTAGCATAATAATTAGTCTTTTAATTGAGGTCTGGAATGAATGGTTTAACAAGAGAATAACTTTATTAATTT--TTAATAATTTGAATTTAATTTTTTGGTTAAAAAGCTAAAATTTTTAATTGGGACGATAAGACCCTATAGATCTT----ATTTTTT--------TAAATTAATTTTTTATATAAG--TTGA--TTTTTTAAAATAAAATAAAAAAAA---ATTCGTTGGGGTGACGTTGAAAACTTTAAAACTTTTAAAA-ATCTTATCA-TT--AATT-AATGATAA-----ATTGATCTTTA-ATTTAGGATTAGTAGATTAAGATACCTTAGGGATAACAGCGTAATAAATTTGGATAGTTCATATTGATAAA--TTTGTTTACGACCTCGATGTTGAATTAATTTA-AAATTTTGGAGTAGAAATTAAA-AATTTGGGTCTGTTCGACCTTTAATTAATTACA |
| *Megadephax cornigera* | TTTAAATAGCTGCAGTATTTTAACTGTACAAAGGTAGCGTAGTAATTAGTCTTTTAATTGAGGTCTTGTATGAATGGTTTGACAAGAAGAAAACTTTATTGAGTTAATTTTA----TTAATTTTATTTTTTAGTTAAAAAGCTAAAATTTTTAAATGGGACGATAAGACCCTATAGATCTT--TAAATTTTTACTAT---TTAAATCAAT--TTAAGGTTA--TTTA--AATTTTATTT----GTAACAAATT---TTTTGTTGGGGTGACATATAAAATTTTAAAACTTTAATAG-TTTTTTACA-TA--GATT-TATGGATT-----TTTGAACCTTA-ATTTTGGGTTATTAAAATTAGATACCTTAGGGATAACAGCGTAATAAATTTGTATAGTTCTCATAGATAAA--TTTGTTTACGACCTCGATGTTGAATTAATCTA-GAGTTTTGGGGCAGAAATTAAA-ATTTTAGGT------------------------ |
| *Megadephax kangauzi* | TTTAAATAGCTGCAGTATTTTAACTGTACAAAGGTAGCGTAGTAATTAGTCTTTTAATTGAGGTCTTGTATGAATGGTTTGACAAGAAGAAAACTTTATTGAGTTAATTGTA----TTAATTTTATTTTTTAGTTAAAAAGCTAAAATTTTTAAATGGGACGATAAGACCCTATAGATCTT--TAAATTTTTATTAT---TTAAATCAAT--TTAAGGTTA--TTTA--AATTTTATTT----GTAACAAATT---TTTTGTTGGGGTGACACATAAAATTTTAAAACTTTAATGGTTTTTTTACA-TA--GATT-TATGGATT-----TTTGAACCCTA-ATTTTGGGTTATTAAAATTAGATACCTTAGGGATAACAGCGTAATAAATTTGTATAGTTCTCATAGATAAA--TTTGTTTACGACCTCGATGTTGAATTAATCTA-GAGTTTTGGGGCAGAAATTAGA-ATTTTAGGTCTGTTCGACCTTTAAAAGATTACA |
| *Mestus cruciatus* | TTTAAATAGCTGCAGTATTTTAACTGTACAAAGGTAGCGTAGTAATTAGTCTTTTAATTGAGGTCTAGAATGAAAGGTTTGACAAGAAAATACCTTTATTAATTTGATTAGA----TGAATTTTATTTTTTGGTTAAAAAGCTAAAATTTTAAAATGGGACGATAAGACCCTATAGATCTT--TAAATTTTTGTTTT--GTAATAGTTAT--TAGGTGTTT--ATAA--AATTTTACTT-----GCAAAAATT---TTTTGTTGGGGTGACAAATAAATTTTTAAAACTTTATTAT-TTAATTTCA-TT--AATA-GATGAATTTA---ATTGAACCTAA-ATTTGGGGTTGTTAAAATAAGATACCTTAGGGATAACAGCGTAATAAATTTGTATAGTTCATATAGATAAA--TTTGTTTACGACCTCGATGTTGAATTAATCTT-AAGTTTTGGGGCAGAAAATAAA-ATTTTAGGTCTGTTCGACCTTTAAAAGATTACA |
| *Metadelphax propinqua* | TTTAAATAGCTGCAGTATTTTAACTGTACAAAGGTAGCGTAGTAATTAGTCTTTTAATTGAGGTCTAGAATGAATGGTTTAACAAGAAATTTACTTTATTAAATTAATTAAA----TGAATTTAATTTTTTGGTTAAAAAGCTAAAATTAAAAAATGGGACGATAAGACCCTATAGATCTT--TAAATTTTTTT------TAACTTTATTATTTTAGTTGA--ATAA--TTTATTTTTA-----TTTAAAATT---TTTAATTGGGGTGATAAACAAATTTTTAAAACTTTGTTAA-TTTTTTACA-TT--AATT-AATGGTTTTA---GTTGAACCTTA-ACTTTGGGTTATTAAAATAAGATACCTTAGGGATAACAGCGTAATAAATTTGTATAGTTCATATAGATAAA--TTTGTTTACGACCTCGATGTTGAATTAACTTA-AAGTTTTGGGGCAGAAAATAAA-ATTTTAGGTCTGTTCGACCTTTAAAAAGTTACA |
| *Miranus serrulatus* | TTTAAATAGCTGCAGTATTTTAACTGTACTAAGGTAGCATAATAATTAGTCTCTTAATTGGGGTCTGGAATGAATGGTTTGACAAAAGAATAACTTTATTAATAAATTTTTT---TTTAATTTTATTTTTTGGTTAAAAAGCTAAAATTTTTAAGTGGGACGATAAGACCCTATAGATCTT--TAAATTTTAATTTA---TAATTATTTT--TTAGTGTGA--AGTA--AATTTTATTT----TTAAAAAATT---TTTTGTTGGGGTGACATATAAATTTTTAAAACTTTATAAT-TTTTTTACA-TT--GATA-GATGTTTTAA---ATTGATCCAATTTTTTTTGATTATTAAAATAAGATACCTTAGGGATAACAGCGTAATAAATTTGTATAGTTCATATAGATAAA--TTTGTTTACGACCTCGATGTTGAATTAATTAA-AATTTTTGGAGCAGAAATTAAA-ATTTTAGGTCTGTTCGACCTTTAAAATATTACA |
| *Miranus varians* | TTTAAATAGCTGCAGTATTTTAACTGTACTAAGGTAGCATAATAATTAGTCTTTTAATTGAGGTCTGGAATGAATGGTTTGACAAAAGAATAACTTTATTAATAAATTTTTT---TTTAATTTTATTTTTTGGTTAAAAAGCTAAAATTTTTAAGTGGGACGATAAGACCCTATAGATCTT--TAAATTTTAATTTA---TAATTATTTT--TTAGTGCAA--AATA--AATTTTATTT-----TAAAAAATT---TTTTGTTGGGGTGACATATAAATTTTTAAAACTTTATAAT-TTTTTTACA-TT--GATA-AATGGTTAAA---ATTGATCCAAT-TTTTTTGATTATTAAAATAAGATACCTTAGGGATAACAGCGTAATAAATTTGTATAGTTCATATAGATAAA--TTTGTTTACGACCTCGATGTTGAATTAATTAA-AATTTTTGGAGCAGAAATTAAA-ATTTTAGGTCTGTTCGACCTTTAAAATATTACA |
| *Monospinodelphax dantur* | TTTAAATAGCTGCAGTATTTTAACTGTACAAAGGTAGCATAATAATTAGTCTTTTAATTGAGGTCTAGAATGAATGGTTTAACAAGAAAAAAACTTTATTTAATTAATTAAT---TTGAATTTAATTTTTTAGTTAAAAAGCTAAAATTTTTAAATGGGACGATAAGACCCTATAGATCTT--TAAATTTTTAGTGT---TAAATACATT--TTAGGTTAT--TTAT--GGTTTTAAAT-------AAAAATT---TTTTGTTGGGGTGACAATTAAAATTTTAAAACTTTATTTT-TTAGCTACA-TA--AATT-TATGG---TATACTTTGATCCTTA-ACTTAGGATTTTAAAAATAAGATACCTTAGGGATAACAGCGTAATAAATTTGTAAAGTTCATATAGATAAA--TTTGTTTACGACCTCGATGTTGAATTAATTAA-AATTTTTGGAGTAGCCGTTAAA-ATTTTAGGTCTGTTCGACCTTTAAAATATTACA |
| *Muirodelphax atratus* | CTTAAATAGCTGCAGTATTTTAACTGTACAAAGGTAGCGTAGTAATTAGTCTTTTAATTGAGGTCTTGTATGAATGGTTTGACAAGAAGAAAACTTTATTGAGTTAATTGTA----TTAATTTTATTTTTTAGTTAAAAAGCTAAAATTTTTAAATGGGACGATAAGACCCTATAGATCTT--TAAATTTTTACTAT---TTAAATCAAT--TTAAGGTTA--TTTA--AATTTTATTT----GTAACAAATT---TTTTGTTGGGGTGACATATAAAATTTTAAAACTTTAATAG-TTTTTTACA-TA--GATT-TATGGATT-----TCTGAACCCTA-ATTTTGGGTTATTAAAATTAGATACCTTAGGGATAACAGCGTAATAAATTTGTATAGTTCTCATAGATAAA--TTTGTTTACGACCTCGATGTTGAATTAATCTA-AAGTTTTGGGGCAGAAATTAAA-ATTTTAGGTCTGTTCGACCTTTAAAAGATTACA |
| *Neobelocera* sp. | TTTAAATAGCTGCGGTAATTTAACTGTACAAAGGTAGCATAATAATTAGTCTTTTAATTGAGGTCTAGAATGAATGGTTTAACAAAATATTAACTTTATTATTTTAAACATT---TTAAATTTAATTTTTTAGTTAAAAAGCTAAAATTTTTAAATGGGACGATAAGACCCTATAGATCTT--TAATTTTAGAAATT---TAATTTTTTT--TTTGATTTT--TTAA--ATTTTGATTT------TCAAAAAT---TTTTGTTGGGGTGATAATAAAAATTTTAAAACTTTTGTTT-TTATATACA-TA--TATT-TATGAATA-----ATTGATCTTGAAAATTCAGATTATAAGAATTAGATACCTTAGGGATAACAGCGTAATAATTTTGGATAGTTCATATTGATAAA--TTTGTTTACGACCTCGATGTTGAATTAATTTA-AAGTTTTGAAGTAAAAATTAAAAATTTTGGGTCTGTTCGACCTTTAGTTAATTACA |
| *Neocarinodelphax hainanensis* | TTTAAATAGCTGCAGTAATTTAACTGTACAAAGGTAGCATAATAATTAGTCTTTTAATTGGGGTCTAGAATGAATGGTTCAACAAAATAAACACTTTATTATTTTAATAAAA---TTTAATTTTATATTTTAGTTAAAAAGCTAAGATTTTTAAATGGGACGATAAGACCCTATAGATCTT--AAATTTTTTTTATC---CTGATTTAAA--TGGCTAATC--TTGA--TTTTTTGGGT----TTAAAAAAAT---TTTCGTTGGGGTGACGT-TAAAATTTTAAAACTTTTAATT-ATAATTACA-CT--TATA-AGTGTATT-----CATGATCTTAA-CTTTTAGATTACAAGAATAAGATACCTTAGGGATAACAGCGTAATAAATTTGAATAGTTCATATTGATAAA--TTTGTTTACGACCTCGATGTTGAATTAATATA-AAATTTTGAAGCAGAAATTAAA-AGTTTAGGTCTGTTCGACCTTTAAATATTTACA |
| *Nilaparvata bakeri* | TTTAAATAGCTGCAGTAATTTAACTGTACAAAGGTAGCATAATAATTAGTCTTTTAATTGAGGTCTAGAATGAATGGTTTAACAAGAAGTAAACTTTATTAATTCAATTAAG----TGAAATTTATTTTTTAGTTAAAAAGCTAAAATTTAAAAATGGGACGATAAGACCCTTTAGATCTT--TAAATTTTAATTAA--ATAACAATATT----AATGTGG--ATAT--AATTTTTATT----TTAAAAAATT---TTTTGTTGGGGTGACAGCTAAAATTTTAAACCTTTAGTGT-TTTATACCA-TT--TTTT-TATGTAAT-----TTTGAACCTTA-ATTTAGGGTTAATAAAATAAGATACCTTAGGGATAACAGCATAATAAATTTGTAAAGTTCATATATATAAA--TTTGATTGTGACCTCGATGTTGAATTAATCTA-GAATTCTGGGGCAGAAAATAGA-ATTTTAGGTCTGTTCGACCTTTAAAAGATTACA |
| *Nilaparvata lugens* | TTTAAATAGCTGCAGTAATTTAACTGTACAAAGGTAGCATAGTAATTAGTCTTTTAATTGAGGTCTAGAATGAATGGTTTAACAAGAAGTAAGCTTTATTAATTCAATTAAG----TGAAATTTATTTTTTAGTTAAAAAGCTAAAATTTAAAAATGGGACGATAAGACCCTTTAGATCTT--TAAATTTTAATTGA--ATAATAATATT----AATGTGG--ATTT--AATTTTTATT----AAATAAAATT---TTTTGTTGGGGTGACAGCTAAAATCTTAAATCTTTAGTAT-TTTACACCA-TT--TATT-TATGTATA-----TTTGAACCTTA-ATTTAGGGTTATTAAAATAAGATACCTTAGGGATAACAGCATAATAAATTTGTAAAGTTCATATATATAAA--TTTGATTGTGACCTCGATGTTGAATTAATCTA-GAATTCTGGGGCAGAAGATAGA-ATTTTAGGTCTGTTCGACCTTTAAAAGATTACA |
| *Nilaparvata muiri* | TTTAAATAGCTGCAGTAATTTAACTGTACAAAGGTAGCATAGTAATTAGTCTTTTAATTGAGGTCTAGAATGAATGGTTTAACAAGAAGTAAACTTTATTAATTCAATTAAG----TGAAATTTATTTTTTAGTTAAAAAGCTAAAATTAAAAAATGGGACGATAAGACCCTTTAGATCTT--TAAATTTTAATTAA--GTAACAATATT---AACGTGGA--TTTA--ATTTTTATTT-----TATAAAATT---TTTTGTTGGGGTGACAGCTAAAATTTTAAACCTTTAGTGT-TTTACACCA-TT--TATT-TATGTAAA-----TTTGAACCTTA-ATTTAGGGTTATTAAAATAAGATACCTTAGGGATAACAGCACAATAAATTTGTAAAGTTCATATATATAAA--TTTGATTGTGACCTCGATGTTGAATTAATCTA-GAATTCTGGGGCAGAAGATAGA-ATTTTAGGTCTGTTCGACCTTTAAAAGATTACA |
| *Numata muiri* | TTTAAATAGCTGCAGTATTTTAACTGTACAAAGGTAGCATAATAATTAGTCTTTTAATTGAGGTCTAGAATGAATGGTTTAACAAGAAAATAACTTTATTTAATTAATTGGG---TTGAATTTAATTTTTTAGTTAAAAAGCTAAAATTTTTAAACAGGACGATAAGACCCTATAGATCTT--TATATTTTAGTGAT---TATACATATT--TTAGGTTAT--GTAA--GGTTTTAAAT-------TAAAATG---ATTTGTTGGGGTGACAAATAAAATTTTTAAACTTTATTTT-TTATTTACA-TA--TATA-AATGTA--TTAA-ATTGATCTTGA-ATTTTGGATTAAAAAAATAAGATACCTTAGGGATAACAGCGTAATAAATTTGTATAGTTCATATAGATAAA--TTTGTTTACGACCTCGATGTTGAATTAATTAA-AATTTTTGGAGCAGAAATTAAA-ATTTTAGGTCTGTTCGACCTTTAAAATATTACA |
| *Opiconsiva albimarginata* | TTTAAATAGCTGCAGTATTTTAACTGTACAAAGGTAGCATAGTAATTAGTCTTTTAATTGAGGTCTTGAATGAATGGTTTGACAAGAAATTAACTTTATTATTTTAATTATA----TGAATTTAATTTTTTAGTTAAAAAGCTAAAATTTTTAAATGGGACGATAAGACCCTGTAGATCTT--TAAATTTTTATTAA---TTAGATTTTT--TTAGTAGAA--ACAA--ATTATTATTA-----GTGTAAATT---TTTTGTTGGGGTGATATTTAAATTTTTAAAACTTTAAAAT-TAATTAACATTT--TATA-GATGAGTC-----ATTGAACCTTT-ACTTTGGTTTATTAAAATAAGATACCTTAGGGATAACAGCGTAATAAGTTTGTATAGTTCTTATAGATAAA--CTTGTTTACGACCTCGATGTTGAATTAACTGA-AAGTTTTGGGGTAGAAAATAAA-ATTTTAGGTCTGTTCGACCTTTAAAAAGTTACA |
| *Opiconsiva nigra* | ATTAAATAGCTGCAGTATTTTAACTGTACAAAGGTAGCGTAGTAATTAGTCTTTTAATTGAGGTCTCGAATGAACGGTTTAACAAGAAAATAACTTTATTTAATTAATTACA----TGAATTTTATTTTTTAGTTAAAAAGCTAAAATAATTAAATGGGACGATAAGACCCTATAGATCTT--AAAATTTTTATAAA---TAAATTCATT--TAAGAAAAG--AAGG--GTTTTCTCTA------TAAAAATT---TTTTGTTGGGGTGACATTGAAAATTTTAAAACTTTTAAGT-GAGAAAACA-TT--AATA-AATGAATTT----TTTGAACTCAA-ACTTTGGGTTGTCAAAATAAGATACCTTAGGGATAACAGCGTAATAAATTTGTATAGTTCATATAATTAAA--TTTGTTTACGACCTCGATGTTGAATTAACTTA-AAGTTTTGGGGCAGAAGATAAA-ATTTTAGGTCTGTTCGACCTTTAAAAGGTTACA |
| *Palego simulator* | TTTAAATAGCTGCAGTATTTTAACTGTACAAAGGTAGCATAGTAATTAGTCTTTTAATTGAGGTCTAGAATGAATGGTTTAACAAGAGAAAAACTTTATTTAATTAATTAGG---TTGAATTTAATTTTTTAGTTAAAAAGCTGAAATTTTTAAGTGGGACGATAAGACCCTATAGATCTT--TAAATTTTAATTAG---TGGATGTATT--TTAGGTTAT--TTAA--GGTTTTTTAT-------TAAAATT---TTTTGTTGGGGTGACAAATAAATTTTTTAAACTTTATTTT-TTAATAACA-TA--AATT-AATGGGTTAG---ATTGATCCTGG-CTTCTGGACTATAAAAATAAGATACCTTAGGGATAACAGCGTAATAAATTTGTATAGTTCATATAGATAAA--TTTGTTTACGACCTCGATGTTGAATTAATTAA-AATTTTTGGAGCAGAAATTAAA-ATTTTAGGTCTGTTCGACCTTTAAAATATTACA |
| *Paradelphacodes paludosa* | TTTAAATAGCTGCAGTAATTTAACTGTACAAAGGTAGCATAATAATTAGTCTTTTAATTGAGGTCTAGAATGAATGGTTTAACAAGAAGTAAACTTTATTAATTCAATTAAG----TGAAATTTATTTTTTAGTTAAAAAGCTAAAATTTAAAAATGGGACGATAAGACCCTTTAGATCTT--TAAATTTTAATTAA--ATAACAATATT----AATGTGG--ATAT--AATTTTTATT----TTAAAAAATT---TTTTGTTGGGGTGACAGCTAAAATTTTAAACCTTTAGTGT-TTTATACCA-TT--TTTT-CATGTAAT-----TTTGAACCTTA-ATTTAGGGTTAATAAAATAAGATACCTTAGGGATAACAGCATAATAAATTTGTAAAGTTCATATATATAAA--TTTGATTGTGACCTCGATGTTGAATTAATCTA-GAATTCTGGGGCAGAAAATAGA-ATTTTAGGTCTGTTCGACCTTTAAAAGATTACA |
| *Peregrinus maidis* | TTTAAATAGCTGCAGTATTTTAACTGTACAAAGGTAGCATAATAATTAGTCTTTTAATTGAGGTCTAGAATGAATGGTTTAACAAGAAAAATTCTTTATTTAATTAATTTAG---TTAAATTTAATTTTTTAGTTAAAAAGCTAAAATTTTTAAATGGGACGATAAGACCCTATAGATCTT--TAAATTTTTATAAC---TAAATATGTT--TTAGGTTAA--ATAG--GGTTTTAAAT-------AAAAATT---TTTTGTTGGGGTGACAATTAAATTTTTAAAACTTTAATGT-TTATTTACA-TA--AATT-TATGAATTCA---ATTGATCTTAA-ATTTTAGATTAATAAAATAAGATACCTTAGGGATAACAGCGTAATAAGTTTGTATAGTCCGTATAGATAAA--TTTGTTTACGACCTCGATGTTGAATTAATTAA-AATTTTTGGAGCAGAATATAAA-ATTTTAGGTCTGTTCGACCTTTAAAATATTACA |
| *Purohita sinica* | TTTAAATAGCTGCAGTATATTAACTGTACAAAGGTAGCATAATAATTAGTCTTTTAATTGAGGTCTAGAATGAACGGTTTAACAAAGTAAAAACTTTCTTATTTTAAATATT---TTGAATTTAATTTTTTGGTTAAAAAGCCAAAATTTTTAAATGGGACGATAAGACCCTATAGATCTT--AAATTTTTTTTTAA--TTAAGTAAATT----GGTTTAA--AAAA--TGTTTAATTT----TTAAAAAAAT---TTTTGTTGGGGTGACATTAAAATTTTTAAATCTTTTAATT-ATTTTTACA-TT--TATA-AATGTATA-----TTTGATCTTAA-CTTTTAGATTAAAAAATTAAGATACCTTAGGGATAACAGCGTAATAAATTTGAATAGTTCATATTGATAAA--TTTGTTTACGACCTCGATGTTGAATTAATTAA-AAATTTTAGAGCAGGCACTTAA-AATTTAGGTCTGTTCGACCTTTAATTTTTTACA |
| *Purohita taiwanensis* | TTTAAATAGCTGCAGTATTTTAACTGTACTAAGGTAGCATAATAATTAGTCTTTTAATTGAGGTCTGGAATGAACGGTTTAACAAGGTGAAAACTTTCTTATTTAAATTACT---TTGAATTTAATTTTTTAGTTAAAAAGCTAAAATTTTTAAATGGGACGATAAGACCCTATAGATCTT--AAATTTTTTTTTAT--CTTAATTTCTTGGTTAATAAAATGTTAA--AATTTTA---------AAAAAAAT---TTTTGTTGGGGTGACATTAAAATTTTTAAATCTTTTAATC-ATTTTTACA-TA--AATA-AATGTATA-----TTTGATCTTAA-CATTTAGATTAAAAAATTAAGATACCTTAGGGATAACAGCGTAATAAATTTGAATAGTTCATATTGATAAA--TTTGTTTACGACCTCGATGTTGAATTAATATA-AAATTTTAGAGCAGAAACTTAA-AGTTTAGGTCTGTTCGACCTTTAAATATTTACA |
| *Purohita theognis* | TTTAAATAGCTGCAGTATTTTAACTGTACTAAGGTAGCATAATAATTAGTCTTTTAATTGGGGTCTAGAATGAAAGGTTTAACAAAGCAAAAACTTTCTTGATTTAATTAAA---TTTAATTTAATTTTTTGGTTAAAAAGCTAAAATTTTTAAATGGGACGATAAGACCCTATAAATCTT--TAATTTTTTTTAAAATTTAAATTTATT------GGGTT--TTAA--AATATTAATA--TTTTAAAAAAAT---TTTTGTTGGGGTGACATTAAAATTTTTTAATCTTTTATTT-ATTTTAACA-TT--AATA-AATGATTTT----TTTGATCTTAA-ATTTTAGATTAAAAAATTAAGATACTTTAGGGATAACAGCGTAATAGATTTGAATAGTTCATATTGATAAA--TTTGTTTACGACCTCGATGTTGAATTAATTTA-AAATTTTAAAGTAGAAATTAAA-AATTTGGGTCTGTTCGACCTTTAATTATTTACA |
| *Rhombotoya psedomigripennis* | TTTAAATAGCTGCAGTATTTTAACTGTACAAAGGTAGCGTAGTAATTAGTCTTTTAATTGGGGTCTAGAATGAAGGGTTTAACAAGAAAAATACTTTATTAGGTTAATAAAA----TGAATTTTATTTTTTAGTTAAAAAGCTAAAATGTATAAATGGGACGATAAGACCCTATAGATCTT--AAAATTTTTTCT-----TAAATTAATTGTTTAGGGGGA--TTTC--GGTTTCTTTA-----AGAAAAATT---TTTTGTTGGGGTGACATTCAAAAACTTAAGACTTTGAAAG-TTTCCTATA-CT--AATA-AGTAATTT-----TTTGAACCCAA-ATTTTGGGTTATTAAAATTAGATACCTTAGGGATAACAGCGTAATAAATTTGTATAGTTCTTATAGATAAA--TTTGTTTACGACCTCGATGTTGAATTAATTTA-AAATTTCGGAGCAGAAGATGGA-ATTTTGGGTCTGTTCGACCCTTAAAAGGTTACA |
| *Ribautodelphax bidentatus* | CTTAAATAGCTGCAGTATTTTAACTGTACAAAGGTAGCGTAGTAATTAGTCTTTTAATTGAGGTCTTGTATGAATGGTTTGACAAGAAGAAAACTTTATTGAGTTAATTATA----TTAATTTTATTTTTTAGTTAAAAAGCTAAAATTTTTAAATGGGACGATAAGACCCTATAGATCTT--TAAATTTTTATTAT---TTAAATCAAT--TTAAGGTTA--TTTA--AATTTTATTT----GTAACAAATT---TTTTGTTGGGGTGACACATAAAATTTTAAAACTTTAATAG-TTTTTTACA-TA--GATT-TATGGATT-----TTTGAACCCTA-ATTTTGGGTTATTAAAATTAGATACCTTAGGGATAACAGCGTAATAAATTTGTATAGTTCTCATAGATAAA--TTTGTTTACGACCTCGATGTTGAATTAATCTA-GAGTTTTGGGGCAGAAATTAGA-ATTTTAGGTCTGTTCGACCTTTAAAAGATTACA |
| *Saccharosydne procerus* | TTTAAATAGCTGCAGTATTTTAACTGTACAAAGGTAGCATAATAATTAGTCTTTTAATTGAGGTCTGGAATGAATGGTTTAACAAAAAAATAACTTTATTAATTTAATTAGT--TTTGAAATTAATTTTTTAGTTAAAAAACTAAAATATTTAAATGGGACGATAAGACCCTATAGATCTT--TAATTTTTTAATAG--ATTAATTTTAT--TTAGTTTAA--TTAA--ATAATTAATT----TTTAAAAAAT---TTTAATTGGGGTGATTATTAAACTTTTTTAACTTTAATTT-TTTTTTACA-TT--CATT-TATGATTT-----GTTGATCTTGA-ATTTTTGATTAAAAGAATAAGATACCTTAGGGATAACAGCACATATAATTTGGATAGTTCATATTGATAAA-TTTTATTTGTGACCTCGATGTTGAATTAATTTA-AACTTTTAGAGCAGAAATTAAAATAGTTAGGTCTGTTCGACCTTTAATAAATTACA |
| *Sardia rostrota* | TTTAAATAGCTGCAGTATTTTAACTGTACAAAGGTAGCATAGTAATTAGTCTTTTAATTGAGGTCTCGAATGAATGGTTTAACAAGAAAATCACTTTATTATATTAATTAAA----TTAATTTTATTTTTTAGTTAAAAAGCTAAAATTATTAAATGGGACGATAAGACCCTATAGATCTT--TAAATTTTTTTAAA---TTAATATATT--TCAGTTGAA--TT-A--ATTTTTATTT----AATAGAAATT---TTTTGTTGGGGTGACATACAAATATTTTAAGCTTTGTAAT-ATACTTACA-TA--AATT-AATGATTTA----TTTGAACCTAA-AGTAAGGGTTATTAAAATAAGATACCTTAGGGATAACAGCGTAATAAACTTGTATAGTTCAGATAGATAAG--TTTGTTTACGACCTCGATGTTGAATTAATTTA-AAATTCTGGGGCAGAAAATAGGAAATTTAGGTCTGTTCGACCTTTAAAAAATTACG |
| *Sinolacme tortilla* | TTTAAATAGCTGCAGTATTTTAACTGTACTAAGGTAGCATAATAATTAGTCTTTTAATTGAGGTCTAGAATGAATGGTTTAACAAGATAATAACTTTATTTAATTAATTAGT---TTGAATTTAATTTTTTAGTTAAAAAGCTAAAATTTTTAAATGGGACGATAAGACCCTATAGATCTT--CAAATTTTAATAAA---TAGAATGGTT--TTAGGTTAT--TTGA--GGTTTAAAAT-------TAAAATT---TTTTGTTGGGGTGACAAATAAAATTTTAAACCTTTATTTT-TAATAAACA-TT--TATT-TATGGTGAA----GTTGATCCTAA-ATTTTGGACTAAAAGATTTAGATACCTTAGGGATAACAGCGTAATAAATTTGAATAGTTCATATAGATAAA--TTTGTTTACGACCTCGATGTTGAATTAATGAA-AATTTTTGGAGTAGAAAATAAA-----------------------------ATACA |
| *Sogata hakonensis* | ATTAAATAGCTGCAGTATTTTAACTGTACTAAGGTAGCATAATAATTAGTCTTTTAATTGAGGTCTGGAATGAATGGTTTGACAAGAAAATAACTTTATTAATTAATCTTTT---TTCAATTTTATTTTTTAGTTAAAAAGCTAAAATTTTTAAATGGGACGATAAGACCCTATAGATCTT--AAAATTTTTTATAT--TTAAAATAATTTTATGGGTTAA--TTTA--AATTTTGTTT-----GGGAAAATT---TTTTGTTGGGGTGACATATAAATTTTTAAAACTTTAAAAT-TTTCTTACA-TT--TATA-GATGTATA-----AATGATCCAAA-CTTTTTGATTATTAAAATAAGATACCTTAGGGATAACAGCGTAATAAATTTGTATAGTACACATAAATAAG--TTTGTTTACGACCTCGATGTTGAATTAATATT-AATTTTTGGAGCAGAAGCTATA-ATTTTGGGTCTGTTCGACCTTTATAATATTACA |
| *Sogata nigrifrons* | ATTAAATAGCTGCAGTATTTTAACTGTACTAAGGTAGCATAATAATTAGTCTTTTAATTGAGGTCTGGAATGAATGGTTTGACAAGAAAATAACTTTATTAATTAATCTTTT---TTCAATTTTATTTTTTAGTTAAAAAGCTAAAATTTTTAAATGGGACGATAAGACCCTATAGATCTT--AAAATTTTTTATATTTAAAATAATTTT--ATGGGTTAA--TTTA--AATTTTGTTT-----GGGAAAATT---TTTTGTTGGGGTGACATATAAATTTTTAAAACTTTAAAAT-TTTCTTACA-TT--TATA-GATGTATA-----AATGATCCAAA-CTTTTTGATTATTAAAATAAGATACCTTAGGGATAACAGCGTAATAAATTTGTATAGTACACATAAATAAG--TTTGTTTACGACCTCGATGTTGAATTAATATT-AATTTTTGGAGCAGAAGCTAGA-ATTTTGGGTCTGTTCGACCTTTTTAATATTACA |
| *Sogatella furcifera* | TTTAAATAGCTGCAGTATTTTAACTGTACAAAGGTAGCATAGTAATTAGTCTTTTAATTGAGGTCTTGAATGAATGGTTTAACAAGAAAAGCACTTTATTATATTAATTAAA----TTAATTTAATTTTTTAGTTAAAAAGCTAAAATTTTTAAATGGGACGATAAGACCCTATAGATCTT--TAAATTTTTTAAAA---TAAATACATT---TCGGTTTA--TTTA--ATTTTCGTTT----AATAAAAATT---TTTTGTTGGGGTGACATACAAAATTTTTAAACTTTGTAAA-ATATCTACA-TT--AATC-AATGATTTTT---TTTGAACCTAA-AATTTAGGTTATTAAAATAAGATACCTTAGGGATAACAGCGTAATAAGTTTGTATAGTTCATATAGATAAA--CTTGTTTGCGACCTCGATGTTGAATTAATTTT-TAATTTTGGAGCAGAAAATAAAAATTTTAGGTCTGTTCGACCTTTAAAAAATTACA |
| *Specinervures basifusca* | TTTAAATAGCTGCAGTAATTTAACTGTACAAAGGTAGCATAATAATTAGTCTTTTAATTGAAGTCTGGTATGAATGGTTTAACAAGATGAAAACTTTATTAATTTAATTAAA--TTTAAATTTAATTTTTTAGTTAAAAAGCTAAAATTAATGAATGGGACGATAAGACCCTATAGATCTT--TAATTTTTTATAAA---ATAGTATTTT--TTAGTTAAT--TTTA--AATTATAATG------TAAAAAAT---TTTAGTTGGGGTGATTGTAAAATTTTTAAAACTTTTATTT-ATTTTTTCT-TA--AATT-TATGTTAA-----ATTGATCTTTA-ATTTTTGATTATAAGAATAAGATACCTTAGGGATAACAGCGTAATAAATTTGGATAGTTCATATTAATAAA--TTTGTTTACGACCTCGATGTTGAATTAATTTA-AAATTTTGAAGCAGAAATTAAA-AATTTGGGTCTGTTCGACCTTTAATTAATTACG |
| *Stenocranus montanus* | TTTAAATAGCTGCAGTATTTTAACTGTACAAAGGTAGCATAGTAATTAGTCTTTTAATTGAGGTCTAGAATGAATGGTTTGTCAAGAAAATAACTTTATTAAGTTATATATT---TTGAATTTTATTTTTAAGTTAAAAAGCTTAAATGATTAATTGGGACGATAAGACCCTATAGATCTT--AAAATTTTTGAATTTTATGAATTTTTG-GTTGGTTAAA--TTTA--ATTAGTT----------AAAAATT---TTTTGTTGGGGTGACAATTAAAATTTTAAAACTTTAATGT-TTTTAACCA-TT--TATA-AATGAAAC-----TAAGATCTTGA-ATTTTTGATTTTTAGAATAAGATACCTTAGGGATAACAGCACAATAATTTTGGATAGTTCATATTGAAAAA--TTTGATTGTGACCTCGATGTTGAATTAATTAA-AGATTAATAAGTAGAAATTTTA-AGTCTAGGTCTGTTCGACCTTTAAAAAATTACA |
| *Stiropis nigrifrons* | TTTAAATAGCTGCAGTAATTTAACTGTACAAAGGTAGCGTAATAATTAGTCTTTTAATTGAGGTCTAGAATGAAAGGTTTGACAAGAAAAAATCTTTATTAATTTAATTTAA----TGAATTTTATTTTTTAGTTAAAAAGCTAAAATTTTTAAATGGGACGATAAGACCCTATAGATCTT--TAAATCTTTGTTTT--ATAAAATTATT--TAGGTGGAA--TTTA--AGTTTTATTG-----ACAGAAATT---TTTTGTTGGGGTGACAAATAAAATTTTAAAACTTTAAAAT-TTAATTACA-TA--AATT-AGTGAATT-----TTTGAACCTAA-ATTTGGGGTTGTTAGAATAAGATACCTTAGGGATAACAGCGTAATAAATTTGTATAGTTCATATAGATAAA--TTTGATTACGACCTCGATGTTGAATTAATCTA-AAGTTTTGGAGCAGAATATAGA-ATTTTAGGTCTGTTCGACCTTTAAAAGATTACA |
| *Struebingianella detecta* | TTTAAATAGCTGCAGTATTTTAACTGTACAAAGGTAGCGTAGTAATTAGTCTTTTAATTGAGGTCTAGAATGAATGGTTTAACAAGAAATTAGCTTTATTAATTCAATTAAA----TTAATTTTATTTTTTAGTTAAAAAGCTAAAATTTAAAAATGGGACGATAAGACCCTATAGATCTT--CAAATTTTTAATTT--ATAGAGCTGTT--TAGGTTTAA--TTTT--ATTTTATTTT------TATAAATT---TTTTGTTGGGGTGACAAATAAATTTTTAAAACTTTAATAT-TTTAACACA-TA--AATT-TATGGATT---AATATGAACCTAA-TCTAAGGTGTAGTAAAATAAGATACCTTAGGGATAACAGCGTAATAGATTTGTATAGTTCTTATAGATAAA--TTTGTTTACGACCTCGATGTTGAATTAATCTA-AAGTTTTGGGGCAGAAATTAAA-ATTTTGGGTCTGTTCGACCCTTAAATGGTTACA |
| *Sulculus sulcatus* | TTTAAATAGCTGCAGTATATTAACTGTACAAAGGTAGCATAATAATTAGTCTTTTAATTGAGGTCTAGAATGAATGGTTTGACAAGAAAAAATCTTTATTTAATTAATTGGG---TTGAATTTAATTTTTTAGTTAAAAAGCTAAAATTTTTAAATGGGACGATAAGACCCTATAGATCTT--TAAATTTTTAAGAA---TAAAAATATT--TTTGGTTAA--TTAG--AGTTTAAAAT-------AGAAATT---TTTAGTTGGGGTGACAAATAAAATTTTAAAACTTTATTTT-TTAATTACA-TA--TATA-TATGTATTT----TAAGATCCTAA-ATTTTGGATTTTAAAAATAAGATACCTTAGGGATAACAGCGTAATAAATTTGTATAGTTCATATAGATAAA--TTTGTTTACGACCTCGATGTTGAATTAATTAA-AATTTTTGGAGCAGAAATTAAA-ATTTTAGGTCTGTTCGACCTTTAGAATATTACA |
| *Tarophagus colocasiae* | TTTAAATAGCTGCAGTAATTTAACTGTACAAAGGTAGCATAATAATTAGTCTTTTAATTGAGGTCTAGAATGAATGGTTTAACAAGAGAAAAGCTTTATTTAATTAATAAAA---TTTAATTTAATTTTTTAGTTAAAAGGCTAAAATTTTTAAATGGGACGATAAGACCCTATAGATCTT--TAAATTTTTAATGA---AAAATTTATT--TTAGATAAT--TTAA--AATTTTCCAT-----TAAAATATT---TTTTGTTGGGGTGACAGTCAAATCTTTAAAACTTTGAATT-TTAATTACA-TG--TATA-TATGGATGTATA-GTTGATCCTAA-ATTTTAGATTACAAGAATAAGATACCTTAGGGATAACAGCGTAATAAATTTGTATAGTTCATATAAGTAAA--TTTGTTTACGACCTCGATGTTGAATTAATAAA-GATTTTTGGAGCAGAAACTAAT-TTCTTAGGTCTGTTCGACCTTTAAAATATTACG |
| *Terauchiana singularis* | TTTAAATAGCTGCAGTATTTTAACTGTACAAAGGTAGCATAATAATTAGTCTTTTAATTGAGGTCTAGAATGAATGGTTTGTCAAGAAAAAAGCTTTATTAAGTTATATTTT---TTGAATTTTATTTTTAAGTTAAAAAGCTTAAATTTTTTATTGGGACGATAAGACCCTATAGATCTT--TAAATTTTTAAATGTTATAGGTTTTTG-GTTGGTTAAA--TTTA--AATTTTTT----------AAAATT---TTTTGTTGGGGTGACAATTAAAATTTAAGAACTTTAATGT-TTTAAACCA-TA--AATT-TATGAAAT-----TAAGATCTTTA-ATTTTTGATTTTTAGAATAAGATACCTTAGGGATAACAGCACAATAGTTTTGGAAAGTTCATATTGAAAAA--ATTGTTTGTGACCTCGATGTTGAATTAATTAA-AGATTTAGAAGCAGAAATTTAA-ATTCTAGGTCTGTTCGACCTTTAAAAAATTACA |
| *Terthron albovittatum* | TTTAAATAGCTGCAGTATTTTAACTGTACAAAGGTAGCGTAATAATTAGTCTTTTAATTGAGGTCTAGCATGAATGGTTTGACAAGAAAATAACTTTATTAATTTAATTTAA---ATGAATTTTATTTTTTAGTTAAAAAGCTAAAATTTTTAAATGGGACGATAAGACCCTATAGATCTT--TAATTTTTTTATTT---ATAAGATATT---TAAGTTAA--TTAC--ATTTATTTTA----ATTAAAAAAT---TTTTGTTGGGGTGACATATAAAATTTTAAAACTTTATAAT-TTTTTTACA-TT--TTTT-GATGATTAAAC--TTTGAACCTTA-ATTTATGTTTATTAAAATAAGATACCTTAGGGATAACAGCGTAATAAATTTGGATAGTTCATATAGATAAA--TTTGTTTACGACCTCGATGTTGAATTAATTTA-AGTTTTAGGGGCAGAAGATTAA-ATTTTAGGTCTGTTCGACCTTTAAAAGATTACA |
| *Toya terryi* | TTTAAATAGCTGCAGTATTTTAACTGTACAAAGGTAGCGTAGTAATTAGTCTTTTAATTGAGGTCTAGAATGAATGGTTTAACAAGAAACCTACTTTATTAAATTAATAAAA----TAAATTTTATTTTTTAGTTAAAAAGCTAAAATTTAAAAATGGGACGATAAGACCCTATAGATCTT--TAAATTTTCTTTAA---AAATTTTATT--TTGGTTGAA----TA--ATTTATTTTT----AAAAAAAATT---TTTTATTGGGGTGATATACAAAATTTTAAAACTTTGTAAA-TTATTTACA-TT--AATT-AATGAT--TTTA-GTTGAACCTAA-ATTTTAGGTTGTTAAAATGAGATACCTTAGGGATAACAGCGTAATAAATTTGTATAGTTCATATAGATAAG--TTTGTTTACGACCTCGATGTTGAATTAACTTA-AAGTTTTGGGGCAGAATATAAA-ATTTTGGGTCTGTTCGACCCTTAAAAGGTTACA |
| *Toyoides albipennis* | ATTTAATAGCTGCAGTATATTAACTGTACAAAGGTAGCATAGTAAATAGTCTTTTAATTGAGGTCTGGAATGAATGGTTTAACAAGAAAAATACTTTATTGATTTAATTTAA----TTAATTTTATTTTTTAGTTAAAAAGCTAAAATACTTAATTGGGACGATAAGACCCTATAGATCTT--AAAATTTTTTAAGA--TTAAATTTATT--TTGGTTTAT--TTAA--TTTTTTGTTT------AAAAAATT---TTTTATTGGGGTGATAGTTAAATTTATAAAACTTTAATTT-TTTAAATCATTA--AATT-TATGAT--TTTAATTTGATCCTGA-AGTTTGGATTGTAAAATCAAGATACCTTAGGGATAACAGCGTAATAAATTTGGATAGTTCATATAGATAAA--TTTGATTACGACCTCGATGTTGAATTAATTTA-AATTCTTGAAGCAGAAAATGAG-ATTTTAGGTCTGTTCGACCTTTAAAAAATTACA |
| *Tropidocephala brunnipennis* | TTTAAATAGCTGCAGTAATTTAACTGTACAAAGGTAGCATAATAATTAGTCTTTTAATTGAGGTCTGGAATGAATGGTTTAACAAAATATCAACTTTATTACTTTAACTTAA---TTTAATTTAATTTTTTAGTTAAAAAGCTAAAATAATTAAATGGGACGATAAGACCCTATAGATCTT--TAATTTTTTTTAAT--AAAAATTTTTT--TTGGTCTTT--TTTA--TATTTAATTT-----AATTAAAAT---TTTAATTGGGGTGATTTAAAAAATTT-AAAACTTTTTAAT-ATTTTTTCA-TA--TATT-AATGTTTA-----TTTGATCTTAA-ATTTTAGATTATTAGATTAAGATACCTTAGGGATAACAGCGTAATAAATTTGAATAGTTCATATTGTTAAA--TTTGTTTACGACCTCGATGTTGAATTAATTTA-AAAATTTGAAGTAGAAATTGAAGTTTTTAGGTCTGTTCGACCTTTAATTAATTACA |
| *Tropidocephala festiva* | TTTAAATAGCTGCAGTAATTTAACTGTACAAAGGTAGCATAATAATTAGTCTTTTAATTGAGGTCTAGAATGAATGGTTTAACAAAATATAATCTTTATTATTTTAATTTAA---TTAAATTTAATTTTTTAGTTAAAAAGCTAACATAATTAAATGGGACGATAAGACCCTATAGATCTT--TAATTTTTTTTTAA---TAAATGTTTT--TTGGTTTTA--TTAA--AATTTAATTT-----AATAAAAAT---TTTAATTGGGGTGATTTAAAAAAATTTAAAACTTTTTAAT-ATTTTTTCA-TA--TATT-AATGTTTA-----TTTGATCTTAA-AATTTAGATTATTAGATTAAGATACCTTAGGGATAACAGCACAATAAGTTTGAATAGTTCATATTGTTAAA--TTTGTTTGTGACCTCGATGTTGAATTAATTTT-AAAATTTGAAGTAGAAATTAAAGTTTTTAGGTCTGTTCGACCTTTAATTAATTACA |
| *Tropidocephala* sp. | TTTAAATAGCTGCAGTAATTTAACTGTACAAAGGTAGCATAATAATTAGTCTTTTAATTGAGGTCTAGAATGAATGGTTTAACAAAATATTAACTTTATTACTTTAATTCAA---TTTAATTTAATTTTTTAGTTAAAAAGCTAAAATAATTAAATGGGACGATAAGACCCTATAGATCTT--TAATTTTTTTTTTAATAAAAATTTTTT--TTTGGTATT--ATCA--ATGTTTAATT----TAATTAAAAT---TTTAATTGGGGTGATTTAAAAAATTT-AAAACTTTTTAAT-GTTTTTTCA-TA--TATT-AATGTTTA-----TTTGATCTTAA-ATTTTAGATTATTAGATTAAGATACCTTAGGGATAACAGCGTAATAAATTTGAATAGTTCATATTGTTAAA--TTTGTTTACGACCTCGATGTTGAATTAATTTT-AAAATTTGAAGTAGAAATTGAAGTTTTTAGGTCTGTTCGACCTTTAATTAATTACA |
| *Ulanar muiri* | TTTAAATAGCTGCAGTATTTTAACTGTACAAAGGTAGCATAATAATTAGTCTTTTAATTGAGGTCTTGAATGAATGGTTTAACAAGAAAACTACTTTATTAAGTTAATTGTG----TTAATTTTATTTTTTAGTTAAAAAGCTAAAATTTTTAAGTGGGACGATAAGACCCTATAGATCTT--TAAATTTTTTTTAA----AAAATTAAT--TTTGGTTAA--TTAA--ATTTTTATTT----AGAAAAAATT---TTTTATTGGGGTGATATATAAAATTTTTATACTTTATGGA-ATTTTTTCA-TT--TATT-AATGATTA-----AATGAACCTAA-ATTTTAGGTTGTTAAAATAAGATACCTTAGGGATAACAGCGTAATAAATTTGTATAGTTCTTATAGATAAA--TTTGTTTACGACCTCGATGTTGAATTAATTTT-AAATTTTGGAGTAGAAGATAAA-GGTTTAGGTCTGTTCGACCTTTAAAAAGTTACA |
| *Unkanodes sapporona* | TTTAAATAGCTGCAGTATATTAACTGTACAAAGGTAGCGTAGTAATTAGTCTTTTAATTGAGGTCTTGTATGAATGGTTTAACAAGAAGAAAACTTTATTAAGTTAATTTTA----TTAATTTTATTTTTTAGTTAAAAAGCTAAAATTTTTAAATGGGACGATAAGACCCTATAGATCTT--TAAATTTTTACTAT---TTAAATTAGT--TTAAGGCTA--TTTA--AATTTTATTT----GTAAAAAATT---TTTTGTTGGGGTGACAGATAAAATTTTCAAACTTTAATAG-TTATTTACA-TA--GATT-TATGGACA-----TTTGAACCTTA-ATTTTGGGTTATTAAAATTAGATACCTTAGGGATAACAGCGTAATAAATTTGTATAGTTCTTATAGATAAA--TTTGTTTACGACCTCGATGTTGAATTAATCTG-AAGTTTTGGGGCAGAAATTAAA-ATTTTAGGTCTGTTCGACCTTTAAAAGATTACA |

**Supplementary** **Table S3** The sequence alignments of *cytb*.

| Taxa | The sequence alignments of *cytb* |
| --- | --- |
| *Arcofaciella verrucosa* | ATTACAAATTTAATATCCGCCATTCCATATCTAGGAAACTCCTTAGTTACATGAGTGTGGGGAGGATTCTCTGTAGATAACCCTACCCTAACCCGATTCTTTTCACTACATTTTATTTTACCTTTCATTTTATCCCTAATAATTACCTTCCATTTAATCTTTTTACACAATAAAGGGTCCTCAAACCCACTGGGATTAAAAAACAAAATTGATAAAATCCCATTTCATCCATTTTTTTCAATTAAAGACATTTTTGGATTTATATTAACCCTAACAATGTTTTCAGTTATTCTATTAAAACTACCTTTTATACTTAACGACCCAGAAAATTTCACCCCCGCTAATTCAATAGTAACCCCCCCCCACATTCAACCTGAATGGTACTTCCTATTTGCTTACGCAATCTTACGATCAATCCCCAATAAACTAGGAGGAGTGGTAGCACTATTACTATCAATTCTAATAATTTTAACCCTACCTTTTACCGTAAATTCTAAATTTAAAAGATTTAACATATACTTTTATAAAAAAATTATATTTTTAGTGTTTATTACAACTTTCATTTTATTAACATGAATTGGAGCACGC |
| *Arcofacies maculatipennis* | ATTACAAACCTAATATCAGCAATTCCTTACCTCGGAAATTCTTTAGTAGTTTGAATCTGAGGGGGGTTTTCAGTTGATAATCCTACTTTAAATCGATTTTTTTCTTTACATTTTATTCTACCTTTTATTTTAACTGTCTTAATTCTTATTCATTTAATTTTTCTTCATGAAAAGGGTTCATCCAATCCACTAGGTTTAAAAAATAAAGTTGATAAGATTGAATTTCATCCTTATTTCACCTTAAAAGATGTCTATGGATTCATAGTTACTTTATTAGTATTCTGTATAATCTTAATAAAAACCCCCTATTTATTTAACGACCCAGAAAATTTTTCCCCAGCTAATTCAATAATTACACCTCCTCACATTCAACCTGAATGATATTTTTTATTTGCTTACGCTATTTTACGATCTATTCCTAACAAACTTGGAGGAGTAATTGCCCTACTGATATCAATTTTAATTATCCTTATTTTACCTTTTTCTATAAATCTCAAGTTTAAAAGATTAAGAATATATAATTATAAAAAATTTTTATTCTTTATATTTGTTTCAACTTTTATTTTATTAACCTGAATTGGAGCACGA |
| *Bambusiphaga fascia* | ATTACAAATTTAGTATCTGCAATTCCTTATTTAGGTTTAACTTTAGTTAATTGAATCTGAGGTGGATTTTCAGTTAATAACCCAACATTAAACCGATTTTTCTCCTTACATTTTATTTTACCATTTATTATAATAATATTAATTTTATTTCACTTAATTTTTTTACATGAAAAAGGATCATCAAATCCACTCGGATTAAAAAATAAAATGGATAAAATTGCTTTCCACCCATATTTTTCAAGAAAAGATATATTTGGATTTATAATAGTAATATTAATGTTTTCAATTATTATTCTAAAAACACCATTTATATTTAACGACCCAGAAAATTTTACCCCAGCTAACCCAATAATTACCCCTCCACATATTCAACCAGAATGATATTTTTTATTTGCTTACGCCATTTTACGGTCTATCCCTAATAAACTAGGAGGAGTTTTAGCTCTTTTATTATCAATTTTAATCATTTTAACTTTACCATTTACAATAAATAATAAATTTAAAAGTTTAAGTATATATATACATAAAAAAATTTTATTTATAATATTCACCGCAATTTTTACACTTTTAACATGAATTGGAGCACGC |
| *Bambusiphaga hainanensis* | ATCACAAATTTAATGTCAGCAATCCCTTATCTAGGATTAATGTTAGTAAACTGAGTATGAGGGGGATTTTCCGTTGAAAACCCTACCCTTAATCGATTTTTTTCTTTACATTTCATTATACCATTTATTTTAACTGTATTAATTTTGGTTCACTTAATTTTTTTGCATGAAAAAGGATCTTCTAACCCATTAGGGTTAAAAAATAAAATTGATAAAATTCCTTTCCACCCTTATTTCTCAATTAAAGATCTATATGGATTTTCCATAACCCTATTAATTTTTTCAATTATTTTATTTAAAATACCATTTTTATTTAATGATCCTGAAAATTTTACCCCAGCCAACCCTATAATAACCCCACCTCATATTCAACCAGAATGATATTTTCTTTTTGCATACGCAATTCTACGTTCTATCCCTAACAAATTAGGAGGTGTAATAGCACTTTTAATATCTATTTTAATTATTTTATCTATACCATTTACAATAAATAGAAAATTTAAAAGTCTTGGTATATACACTCATAAAAAAATTATATTTTTTATATTTATTTCAACTTTTATTTTACTTACATGAATTGGAGCACGC |
| *Bambusiphaga luodianensis* | ATCACTAATTTGATATCAGCAATTCCTTATATAGGATCAACTTTAGTCAAATGAATCTGAGGGGGATTTTCTGTAGAAAATCCAACCCTAAATCGATTCTTCTCATTACACTTTATTTTACCATTTATCTTAGCTGTATTAATTATAATTCACCTAATTTTTCTACATGAAAAAGGATCATCAAATCCTTTAGGATTAAAAAATAAAATTGATAAAATTTGTTTCCACCCTTACTTTACTATTAAAGATTTATTTGGATTTTTAATAACTCTACTAATATTTTCAATTGTACTGTTAAAAACACCATTTTTATTCAATGACCCTGAAAATTTCACCCCAGCTAACCCTATAGTAACACCACCCCACATTCAACCCGAATGATATTTTCTTTTTGCGTACGCAATTTTACGTTCAATTCCTAATAAACTAGGAGGAGTCTTAGCACTCTTAATATCCATTTTAATCATTTTAACTCTACCATTCACTATAAAAAGAAAATTTAAAAGTTTAGGAATATACACTTATAAAAAAATTTTATTTTTTATTTTTGTATCAACATTTATTTTATTAACATGAATTGGAGCACGA |
| *Bambusiphaga maculata* | ATTACAAATTTAGTGTCAGCAATTCCCTATTTAGGAGTATCAGTGGTTAACTGAGTATGAGGAGGATTTTCAGTTGAAAACCCAACTTTAAACCGATTTTTTTCATTACATTTTATATTACCTTTTATTTTAACTGTATTAATTTTGTTTCACTTAATTTTTTTACATGAAAAAGGGTCATCAAATCCCCTTGGGTTAAAAAATAAAATAGATAAAATTTCGTTTCACCCTTATTTTTCTATTAAAGATTTATTTGGGTTTTTATTGACTTTAATGTTATTTTCAGTAATTTTATTTAAAACACCATTTTTATTTAATGACCCTGAAAACTTTACTCCAGCAAATCCAATGGTTACCCCACCTCATATTCAACCAGAATGATATTTTCTGTTTGCTTACGCTATTTTACGCTCAATCCCTAATAAACTAGGTGGAGTTATAGCTTTATTACTATCAATTTTAATTATTTTAACATTGCCATTTACCATAAATGCTAAATTTAAAAGTCTTAACATATATATACATAAAAAAATTTTATTCTTTATTTTTATTTCAATTTTTCTGTTATTAACATGAATTGGAGCACGA |
| *Bambusiphaga membranacea* | ATTACAAATTTAATATCTGCAGTTCCTTATCTAGGATTAACATTAGTAAACTGAGTATGGGGAGGGTTTTCAGTTGAAAACCCTACACTAAATCGATTTTTCTCATTACACTTTATTATACCATTTATTTTAACTGTATTAATTTTAATTCACTTAATTTTTTTACATGAAAAAGGGTCATCAAACCCACTAGGGTTAAAAAATAAAATTGATAAAATTTCTTTTCACCCCTATTTCTCAATTAAAGATTTATTCGGATTTTTATTAACTCTTTTAGTTTTCTCAATTATTTTATTCAAAACACCATTTTTATTTAATGATCCTGAAAATTTTACCCCAGCTAATCCTATAGTAACCCCCCCCCATATTCAACCTGAATGATACTTTCTTTTTGCATATGCAATCTTACGCTCTATCCCTAATAAGTTAGGAGGAGTATTAGCATTATTAATATCTATCCTAATTATTTTATCTTTACCATTTACAATAAATAGAAAATTTAAAAGTCTAGGTATATACACTCACAAAAAAATTTTATTTTTTATATTCATTTCAACTTTCATTTTACTTACATGAATTGGAGCACGC |
| *Bambusiphaga mirostylis* | ATCACAAATTTAATATCAGCTATTCCTTATCTTGGTCAAAATTTAGTAAACTGAATTTGAGGGGGGTTCTCAGTAGAAAACCCAACACTTAACCGATTCTTTTCTTTGCACTTTATCCTACCATTTATTTTAATATTTATAATTTTGTTCCATCTAATTTTTCTTCATGAAAAAGGTTCTTCAAACCCCATAGGTTTAAAAAATAAAATTGATAAAATCTCATTTCACCCTTATTTTTCTATTAAAGATATTTTTGGATTTTTATTAACCCTATTAATATTCGTAATTATTTTATTTAAAACACCATTTCTATTTAATGATCCTGAAAATTTCACCCCCGCTAATTCCATAGTTACCCCCCCCCATATTCAACCAGAATGATATTTTCTATTTGCCTATGCTATTTTACGTTCTATCCCTAACAAATTAGGTGGTGTATTAGCATTACTAATATCTATCTTAATTATTTTAACTTTACCATTTACAATAAAAAGAAAATTTAAAAGTTTAGGCATATACACACATAAAAAATTTTTGTTTTTTACTTTTGTGTCTATTTTTGTTTTATTAACCTGAATTGGAGCACGC |
| *Bambusiphaga taibaishana* | ATTACAAATTTAATATCCGCAATTCCATATTTAGGTTTAACTTTAGTTAACTGAATCTGAGGGGGATTTTCAGTAAATAACCCAACATTAAACCGTTTTTTCTCATTACATTTCATTTTACCATTTATTATAATAATATTAATTTTATTCCACTTAATTTTCTTGCATGAAAAAGGTTCATCAAACCCCCTTGGCTTAAAAAATAAAATAGATAAAATTACTTTTCACCCTTATTTTTCAAGAAAAGACTTATTTGGATTTATATTAGTTATATTTTTATTTTCAATTATTATTTTAAAAACACCATTTTTATTTAATGACCCAGAAAATTTTACTCCAGCTAACCCCATAATTACTCCCCCACACATTCAACCTGAATGATATTTTTTGTTTGCTTACGCTATCCTACGATCTATCCCTAATAAATTAGGAGGAGTTTTAGCTCTTTTATTTTCAATCTTAATTATTTTAACTTTACCATTTACAATAAATAGTAAATTTAAAAGTTTAAGTATATACACACATAAAAAAATTTTATTTATTTTATTTACTTCAATTTTTATACTTTTAACATGAATTGGAGCACGC |
| *Carinofrons maculatipennis* | ATTACAAATTTAATATCAGCTATTCCCTATTTAGGTGATATAATGGTTACATGGGTATGGGGGGGATYCTCAGTTGATAACCCAACGCTGAATCGATTCTTTTCTCTACATTTTATATTGCCTTTTATTTTAACAGTTTTAATTTTAATACACTTAATTTTTCTACATGAAAACGGCTCATCTAACCCATTAGGGCTAAAAAATAAAATTGACAAAGTTTCCTTTCACCCTTATTTCTCAGTAAAAGATATTTACGGATTTATTGTAACCTTGTTTATTTTTTCATTTATTATTTTCAAAACCCCGTTTCTATTTAATGACCCAGAAAATTTTATACCAGCTAATCCTATAGTGACACCCCCTCACATTCAGCCTGAATGATATTTTTTATTTGCATATGCGATTTTACGTTCGATTCCCAATAAATTGGGAGGTGTTTTAGCATTATTAATATCAATTTTGATCATTTTAACACTTCCATTTACTATAAACTCAAAATTTAAAAGTTTAAGAATATACATACACAAAAAAATTTTATTTTTTATGTTTGTGTCAATCTTCATTCTATTAACCTGAATTGGAGCACGA |
| *Cemopsis griphus* | ATTACAAATCTTCTTTCTGCCTTTCCATATATTGGAAATATTTTAGTTAAATGAATTTGAGGGGGATTCTCAGTTGAAAATCCCACCCTTAATCGATTTTTTTCTTTCCACTTTATTTTACCTTTTATCCTTTTAACCTTAATCTTATTTCACCTAATTTTTTTACATGAAAAAGGTTCATCTAACCCGTTAGGGTTAAAAAATAAAATTGATAAAATTAGTTTTCATCCATATTTCACCCTAAAAGATATTTATGGATTTATAATAATTATACTTGTATTTATTTTTATCAATTTAAAAACACCATTTTTATTTAATGATCCAGAAAATTTTAGACCAGCAAACCCAATAATTACTCCCCCCCATATTCAACCAGAGTGATATTTTCTTTTTGCTTATGCTATTCTACGTTCAATCCCTAATAAACTGGGGGGGGTTCTAGCTTTAGTTTTCTCAATTTTAATTATTTTAATTTTACCTTTTACAATAAATTTAAAATTTAAAAGTTCTAGAATATATTTAACTAAAAAAATTTCATTTTTTATTTTCTCTTCAACATTTATTTTATTAACATGAATTGGAGCACGA |
| *Cemus macaoensis* | ATTACAAACCTACTTTCTGCTATTCCTTATTTAGGAGACACATTAGTGAAATGAATTTGAGGTGGGTTTTCAGTAGAAAACCCTACCCTTAACCGATTTTTTTCTTTCCACTTTATCTTACCTTTCACATTATCTTTATTAATTTTATTTCACTTAATTTTTCTACATGAAAGGGGTTCTTCAAATCCCCTTGGACTAAAAAATAAAGTTGATAAAATCAGATTCCACCCATACTTTACTTTAAAGGATATCTACGGATTTATAGTAATTTTACTTATATTTATTTTTATTAACTTTAAAACCCCATTCTTATTTAATGACCCAGAAAATTTTACTCCTGCAAACCCCATAATTACACCACCCCATATCCAGCCAGAATGATATTTTCTTTTTGCTTACGCCATTTTACGTTCTATTCCCAATAAACTAGGTGGAGTTCTCGCCCTAATTTTTTCTATCTTAATTATTTTAATACTCCCATTTACAATAAATCTAAAATTTAAAAGATCAAGAATATATTTAATTAAAAAGATCACATTTTGTTTATTTTGTTCAACCTTCATTTTACTAACATGAATTGGAGCACGC |
| *Cemus nigromaculosus* | ATTACAAATTTATTGTCTGCTATCCCCTATTTAGGAAACACTTTAGTTACATGGATCTGAGGAGGATTTTCAGTAGAAAACCCCACACTAAACCGATTCTTTTCTTTCCATTTTATCTTACCATTTATGCTAGTATTATTAATTTTAGTTCATTTAATTTTTCTACATGAAAAAGGATCCTCTAACCCCCTAGGTTTAAAAAATAAAACCGACAAAATCAGTTTTCATCCATATTTTACCCTAAAAGATATTTTTGGATTTACTATGATCCTACTCATTTTTATCTTCATTAACTTTAAAACACCATTTCTATTTAATGATCCAGAAAATTTCACACCAGCAAACCCCATAATTACACCCCCTCACATCCAGCCTGAATGATATTTCCTATTTGCCTATGCTATCCTACGTTCTATCCCCAATAAGTTGGGGGGAGTGTTAGCTTTAATTTTTTCAATCTTAATTATTTTGACACTACCATTTACAATAAATTTAAAATTTAAAAGATCAAGTATATATATAACAAAAAAAATTTCATTTTATATATTCTGTTCAACATTTATTTTATTAACATGAATTGGAGCACGA |
| *Cemus punctatus* | ATTACTAATCTTTTGTCCGCCATCCCTTACTTAGGTAATACAATAGTAACATGAATTTGAGGGGGATTTTCCGTAGAAAATCCTACCCTTAACCGATTCTTTTCTTTTCATTTTATTTTACCTTTTGCTTTATCTTTATTAATTTTATTTCATTTAATTTTCCTCCATGAAAAGGGCTCTTCCAACCCCCTTGGTTTAAAAAATAAAATTGATAAGATTAGATTTCATCCATATTTTACTTTAAAGGATATTTATGGATTTATTATAATTATATTGGTATTTATATTTATTAACTTTAAGACCCCATTTTTATTTAATGATCCAGAAAATTTTACACCAGCAAACCCAATAATTACTCCCCCTCATATTCAACCAGAATGATACTTTCTTTTCGCTTATGCTATTTTACGTTCTATCCCTAACAAACTGGGGGGAGTACTGGCCTTAATTTTTTCAATCTTAATTATCTTAATTCTCCCCTTTACAATAAATTTAAAATTTAAAAGATCAAGAATATATATTACTAAGAAAATCACATTTTTTATGTTATGTTCAACCTTTATCTTACTAACGTGAATTGGAGCACGC |
| *Cemus sauteri* | ATTACAAATTTATTGTCTGCTATCCCCTATTTAGGAAACACTTTAGTTACATGGATCTGAGGAGGATTTTCAGTAGAAAACCCCACACTAAACCGATTCTTTTCTTTCCATTTTATCTTACCATTTATGCTAATATTATTAATTTTAGTTCATTTAATTTTTCTACATGAAAAAGGATCCTCTAACCCCCTAGGTTTAAAAAATAAAACCGATAAAATCAGTTTTCATCCATATTTTACCCTAAAAGATATTTTTGGATTTACTATGATCCTACTCATTTTTATCTTTATTAACTTTAAAACACCATTTCTATTTAATGATCCAGAAAATTTCACACCAGCAAACCCTATAATTACACCCCCCCACATCCAGCCTGAATGATATTTCCTATTTGCCTATGCTATCCTACGTTCTATCCCCAATAAGTTGGGGGGAGTGTTAGCTTTAATTTTCTCAATCTTAATTATTTTGACACTACCATTTACAATAAATTTAAAATTTAAAAGATCGAGTATATATGTAACAAAAAAAATTTCATTTTATATATTCTGTTCAACATTTATTTTATTAACATGAATTGGAGCACGC |
| *Cixius scrupeus* | ATTACAAACTTAATTTCTGCTGTACCATATTTAGGAAAAATAATTGTCAATTGAATCTGAGGAGGATTTTCAGTTGATAACCCAACATTAAACCGATTTTTTACATTACACTTTTTACTCCCTTTTATTTTAGCTTTAATAATTATTATACATTTAAATTTTCTTCATAGAACAGGATCCTCTAACCCATTAGGATTAAAAAATAATGTTGATAAAATTCCATTCCATCCATTTTTTACAATTAAAGATGTTATAGGTTTTATTTTTACACTTATATTATTTTCAATTGTAATTCTTAAAGAACCTTTTATTTTAATAGATCCAGAAAATTTTATACCAGCTAATCCAATAATTACCCCCCCACATATTCAACCTGAATGATATTTTTTATTTGCTTATGCAATTCTACGTTCAATTCCTAATAAATTAGGTGGTGTAATTGCTTTATTAATATCAATTTTAATTTTATTAACTTTACCATTTTCAATAAAATCAAAATTTAAAAGTTCATCATTTTACCCCTTAAACAAATTTATATTTTGAATTTTAGTAATAAGAACTATTTTATTAACATGAATTGGAGCACGC |
| *Ecdelphax dentata* | ATTACAAATCTTCTATCTGCAGTCCCCTATATAGGGAATACATTAGTAACATGAATTTGGGGGGGGTTTTCAGTTGAAAATCCCACCCTTAATCGATTTTTTTCTTTCCATTTTATTTTACCTTTTATTATTTCTGTATTAATTTTATTTCACCTAATTTTATTACATGAAAAAGGTTCATCTAACCCCCTTGGGTTAAAAAATAAAATTGATAAAATTAGATTCCACCCATATTTTACATTAAAAGACATTTTTGGATTTACATTAATTTTTTTAGTATTCATTTTTATCAATATAAAAACTCCTTTTCTATTTAATGACCCTGAAAATTTTTCACCAGCAAATCCAATAATTACTCCTCCCCATATTCAACCAGAATGATACTTTTTATTTGCTTATGCTATTCTACGGTCTATCCCTAATAAATTAGGGGGAGTTATAGCCTTATTATTTTCAATTCTTATTATTTTAATCCTACCATTTACAATAAATTTAAAATTTAAAAGATCAAGAATATATATTTCAAAAAAAATTCTTTTTTATATATTTTGTTCAACTTTTATTTTATTAACATGAATTGGAGCACGC |
| *Epeurysa distincta* | ATTACAAATTTAATATCAGCTATTCCCTATTTAGGTGATATAATGGTTACATGGGTATGGGGGGGATTCTCAGTTGATAACCCAACGCTGAATCGATTCTTTTCTCTACATTTTATATTGCCTTTTATTTTAACAGTTTTAATTTTAATACACTTAATTTTTCTACATGAAAACGGCTCATCTAACCCATTAGGGCTAAAAAATAAAATTGACAAAGTTTCCTTTCACCCTTATTTCTCAGTAAAAGATATTTACGGATTTATTGTAACCTTGTTTATTTTTTCATTTATTATTTTCAAAACCCCGTTTCTATTTAATGACCCAGAAAATTTTATACCAGCTAATCCTATAGTGACACCCCCTCACATTCAGCCTGAATGATATTTTTTATTTGCATATGCGATTTTACGTTCGATTCCCAATAAATTGGGAGGTGTTTTAGCATTATTAATATCAATTTTGATCATTTTAACACTTCCATTTACTATAAACTCAAAATTTAAAAGTTTAAGAATATACATACACAAAAAAATTTTATTTTTTATGTTTGTGTCAATCTTCATTCTATTAACCTGAATTGGAGCACGC |
| *Epeurysa infumata* | ATTACAAATTTAATATCAGCCATTCCCTATTTAGGAAATATAATAGTTACTTGAGTGTGGGGGGGGTTTTCAGTTGATAGCCCAACACTTAACCGATTTTTTTCACTACATTTTATACTACCATTTATTTTAACAGTTTTTATCTTAATACACTTGATTTTCCTACATGAAAATGGGTCATCTAATCCACTAGGATTAAAAAATAAAATTGACAAAATCTCTTTTCACCCCTATTTCTCAGTAAAAGACATTTATGGATTTGTTATAACCTTGTTTATTTTTTCAATTATTCTCTTCAAAACCCCGTTTCTATTTAATGACCCAGAAAATTTTACACCAGCTAATTCTATAGTAACCCCACCACATATTCAACCTGAGTGATACTTTTTATTTGCATATGCTATTCTACGTTCAATTCCCAATAAATTGGGAGGAGTTTTAGCATTATTAATATCAATTTTGATTATTTTAACACTACCATTTACTATAAATTCAAAATTTAAAAGTTTAAGAATATACACACACAAAAAAATTTTATTCTTTATATTTGTGGCAATCTTCATCTTATTAACATGAATTGGAGCACGC |
| *Epeurysa nawaii* | ATTACTAATTTAATATCAGCCATTCCCTATTTAGGAAATATAATAGTTACTTGAGTGTGGGGGGGGTTTTCAGTTGATAACCCAACACTTAACCGATTTTTTTCACTACATTTTATACTACCATTTATTTTAACAGTTTTTATCTTAATACACTTGATTTTCCTACATGAAAATGGGTCATCTAATCCACTAGGATTAAAAAATAAAATTGACAAAATCTCTTTTCACCCCTATTTCTCAGTAAAAGACATTTATGGATTTGTTATAACCTTGTTTATTTTTTCAATTATTCTCTTCAAAACCCCGTTTCTATTTAATGACCCAGAAAATTTTACACCAGCTAATTCTATAGTAACCCCACCACATATTCAACCTGAGTGATACTTTTTATTTGCATATGCTATTCTACGTTCAATTCCCAATAAATTGGGAGGAGTTTTAGCATTATTAATATCAATTTTGATTATTTTAACACTACCATTTACTATAAATTCAAAATTTAAAAGTTTAAGAATATACACACACAAAAAAATTTTATTCTTTATATTTGTGGCAATCTTCATCTTATTAACATGAATTGGAGCACGC |
| *Falcotoya lyraeformis* | ATTACAAATTTATTATCTGCAATTCCTTATTTAGGACAAATATTGGTTAATTGAATTTGAGGGGGATTTTCAGTTGAAAATCCAACTCTTAACCGTTTTTTTTCTTTTCATTTCATTTTGCCTTTCATTTTATGCTTAATAATTATATTTCACTTAATTTTTTTACATGAAAAAGGTTCTTCAAACCCCCTAGGACTCAAGAATAAGATTGACAAAATCAGTTTTCATCCTTATTTCTCTATTAAAGACATTTATGGTTTTTTTGTAGTTTTAATATTATTTTTATTTATTAATTTTAAAACTCCATTCATATTTAATGATCCTGAGAACTTTACCCCTGCTAATCCTATAGTTACACCACCACATATCCAACCTGAATGATACTTCCTTTTTGCTTATGCAATTTTACGATCTATCCCTAATAAACTTGGTGGTGTATTAGCTCTTTTAATATCAATTCTAATTATTTTAACTTTACCTTTTACCTCTAATTTTAAATTTAAAAGTGCTAGAATATACACAACTAAAAAGATTATTTTTTCAGTATTTTGTGGAACATTTATTTTATTAACTTGAATTGGAGCACGC |
| Gen. nov*.* 1 | ATTACAAATTTAGTTTCTGCCATTCCTTATCTAGGTAATTCTGTGGTAACTTGAATTTGAGGGGGATTTTCAGTTGATAACCCTACTTTAAATCGATTTTTTTCTTTACATTTTATGTTACCATTTTTATTAATAATATTAATCATTTTTCACCTAATTTTTTTACATGAAAGGGGATCATCTAACCCTCTTGGTTTAAAAAATAAAATTGATAAAATTAATTTTCACCCTTATTTTTCCTTGAAAGACTTATATGGATTTATTTTAACTTTAATACTATTTACAATGATTTTAATAAACACACCGTATTTATTCAATGACCCAGAAAATTTTATACCAGCTAATTCAATAGTTACACCCCCTCATATTCAACCTGAATGATATTTTCTATTTGCTTATGCCATTTTACGTTCTATCCCCAATAAGCTGGGGGGCGTTATAGCTTTAGTACTATCAATTTTAATTATTTTAACTTTATCTTTTTCTATTAACCACAAATTTAAAAGAACCAGTATATATCTATACAAGAAAATTATATTTTTTATATTTGTTTCTATTTTCATTTTATTAACATGAATTGGAGCACGA |
| *Hadeodelphax afurculus* | ATCACAAATTTACTTTCTGCTATCCCCTACATTGGAATGATACTTGTTACATGAATTTGAGGAGGGTTTTCAGTTGAAAACCCCACACTAAATCGATTTTTTACATTTCATTTTATTCTACCCTTTATATTAACATTTATAATTTTAATTCATTTAGTACTTCTACATGAAAAAGGTTCATCAAACCCATTAGGTTTAAAAAATAAAATTGATAAAATTAGATTTCATCCTTATTTTACTCTAAAAGATTTATATGGATTTTTAATTGTTGTTTCAGTTTTCTTTCTTATCAATTTTAAAATACCATTTTTATTTAATGACCCAGAAAATTTCACACCTGCTAACCCAATAGTAACTCCTCCCCACATCCAACCAGAATGATACTTTCTTTTCGCTTACGCTATTTTACGCTCTATCCCCAACAAACTCGGCGGAGTTTTAGGCCTTGTAATATCAATTTTAATTATTTTGAGTCTTCCATTCTCCTATAACTTAAAATTTAAAAGTTCATCAATATACTTAATTAAAAAAATTTTATTTTTTATATTCTGCCTAACTTTCATTTTATTAACTTGAATTGGAGCACGA |
| *Harmalia gayasana* | ATTACTAATCTCTTATCTGCAATCCCTTACGTAGGGGTCACTTTAGTAACTTGAATCTGAGGGGGGTTTTCAGTAGAAAATCCTACACTAAATCGATTTTTTTCATTCCATTTTATTTTACCTTTCATTTTATGTATAATAATCATAATACACCTAATCTTTTTACATGAAAAAGGTTCATCAAACCCTTTAGGGTTAAAAAATAAAATTGATAAAATTAGTTTTCACCCCTACTTCTCTATTAAAGATATATATGGATTTTTAATTGTAATAGTCTTATTTCTTTTTATTAACTTTAAAACCCCCTTTCTTTTTAATGATCCAGAAAATTTTACCCCCGCTAACCCAATAATTACTCCCCCTCACATTCAACCCGAATGATACTTCCTTTTTGCTTACGCAATTTTACGATCTATCCCTAATAAATTAGGGGGAGTTGTAGCTCTTTTAATATCAATTTTAATTATCTTAATTTTACCTTTTACAATAAACATAAAATTCAAAAGAACAAGAATATACAATTTTAAAAAAATCTATTTCTTAATCTTTTGCAGAACTTTTATTATTTTAACTTGAATTGGAGCACGA |
| *Harmalia sirokata* | ATTACTAATCTCTTATCTGCAATCCCTTACGTAGGGGTCACTTTAGTAACTTGAATCTGAGGGGGGTTTTCAGTAGAAAACCCTACACTAAATCGATTTTTTTCATTCCATTTTATTTTACCTTTCATTTTATGTGTAATAATCATAATACACCTAATCTTTTTACATGAAAAAGGTTCATCAAACCCTTTAGGGTTAAAAAATAAAATTGATAAAATTAGTTTTCACCCCTACTTCTCTATTAAAGATATATATGGATTTTTAATTGTGACAGTTTTATTTCTTTTTATTAACTTTAAAACCCCCTTTCTTTTTAATGATCCAGAAAATTTTACCCCCGCTAACCCAATAATTACTCCCCCTCACATTCAACCCGAATGATACTTCCTTTTTGCTTACGCAATTTTACGATCTATCCCTAATAAATTAGGGGGAGTTGTAGCTCTTTTAATATCAATTTTAATTATCTTAATTTTACCTTTTACAATAAACATAAAATTTAAAAGAACAAGAATATACAATTTTAAAAAGATCTATTTCTTAATCTTTTGCAGAACTTTTATTATTTTAACTTGAATTGGAGCACGA |
| *Himeunka tateyamaella* | ATTACTAATCTATTATCAGCTATTCCTTACTTAGGAACCACCCTAGTAACTTGAATTTGAGGGGGCTTTTCAGTAGAAAACCCTACCCTTAATCGATTCTTTTCATTTCATTTTATCTTACCATTTATTTTATGTTTAATAATTTTATTTCACTTAATTTTTTTACATGAAAGGGGCTCATCTAACCCCCTGGGACTGAAAAACAAAGTTGATAAAATTAGTTTCCACCCGTATTTTTCAATTAAAGATATTTATGGGTTTATAATTGTAATTTTAGTATTTTTATTTATTAACTTTAAAACCCCCTTCCTCTTTAATGACCCTGAAAATTTTACACCAGCTAACCCAATAGTAACTCCACCTCATATTCAACCTGAATGGTATTTCCTTTTTGCTTACGCAATCCTACGGTCAATCCCCAATAAACTAGGGGGTGTAGTAGCTCTAGTAATATCAATTCTTATTATTCTTACTTTACCATTAACAATAAATTTAAAATTCAAAAGATCAAGAATATATATCATTAAAAAAATTTTATTTATAACTTTCTGTAGAACATTTTTACTTTTAACTTGAATTGGAGCACGC |
| *Ishiharodelphax matsuyamensis* | ATTACAAATTTATTATCTGCAATTCCTTATTTAGGACAGACTATAGTAAATTGAATTTGAGGTGGATTTTCAGTTGAAAACCCTACACTTAACCGTTTTTTCTCTTTCCATTTTATTTTACCTTTCATTTTATGTTTAATAATTATACTTCACTTAATTTTTTTACACGAAAAAGGTTCATCAAACCCTTTAGGACTAAAAAATAAAGTTGATAAAATCAGTTTTCATCCTTATTTTTCTATTAAAGATATATATGGGTTTGTTGTAGTATTAATATTATTTATGTTTATCAACTTCAAAATCCCATTTTTATTTAATGACCCTGAAAATTTTACCCCTGCTAACCCTATAACTACACCCCCCCACATTCAACCTGAATGATATTTCCTTTTTGCTTATGCAATTTTACGGTCTATCCCCAATAAACTTGGGGGGGTGTTAGCTCTTTTAATATCAATTTTAATTATTTTAACATTACCCTTTAATTCAAATTTTAAATTTAAAAGATCTAGAATATATTTAACTAAAAAGATTCTTTTTTCTATATTTTGTGGAACATTTATTTTATTAACGTGAATTGGAGCACGA |
| *Laodelphax striatellus* | ATTACTAATCTTTTATCAGCTATTCCCTATTTAGGAAATACATTAGTTTCATGAATTTGAGGAGGATTCTCCGTAGAAAATCCTACTTTAAACCGATTTTTTTCATTTCACTTCATTTTACCCTTTATTTTATGTTTAATAATTATATTTCACTTAATTTTTTTACACGAAAAAGGATCATCAAATCCCCTTGGCTTAAAAAATAAAATTGACAAAATTTCCTTCCATCCTTACTTTACAATTAAAGATTTATACGGATTTATATTAGTTTTTATTATATTTTTATTTATTAACTTTAAAACACCATTCCTATTGAATGACCCCGAAAATTTTACACCAGCTAACCCTATAGTAACCCCCCCACATATTCAACCTGAATGATATTTTCTATTTGCCTACGCTATTTTACGGTCAATCCCTAACAAACTAGGGGGAGTTTTAGCATTATTTATATCAATTATTATTATTTTAACTTTACCATTTACTATAAATTTAAAATTTAAAAGATCAAGAATATATTTAGTTAAAAAAATCTTATTTTTAATTTTCTGTAGAACATTTATATTATTAACATGAATTGGAGCACGA |
| *Latistria placitus* | ATTACAAATTTAATATCAGCTGTTCCTTATTTAGGTTACACCTTAGTAAATTGAATTTGAGGGGGATTTTCTGTTGAAAATCCTACCCTTAACCGATTTTTTTCGTTTCATTTTATTTTACCTTTTGTTTTGTGTTTAATAATTTTATTTCACTTAATTTTTTTACATGAAAAAGGTTCTTCTAACCCCCTGGGATTAAAAAATAAAGTTGATAAAATCAGTTTCCACCCTTATTTTTCGATCAAGGATATTTATGGATTTATAATTGTTATTATAATATTTTTATTTGTTAATTTTAAAACTCCTTTTCTATTTAATGACCCTGAAAATTTTACACCAGCAAATCCATTAGTAACTCCCCCTCACATTCAACCTGAATGATACTTTCTATTTGCTTACGCAATTTTACGGTCTATCCCCAACAAATTAGGGGGAGTTTTAGCTCTTATTATATCAATTGTTATTATTTTGAGTTTACCATTAACAATAAACCTAAAATTTAAAAGATCAAGTATATACACTCTTAAAAAAATTTTGTTTTTAGTATTTTGTTTAACATTTGTACTTTTAACCTGAATTGGAGCACGA |
| *Malaxa semifusca* | ATTACTAATTTAATGTCAGCTATCCCTTACTTGGGTGTAACTTTAGTTAGTTGAATTTGAGGGGGGTTTTCAGTAGAAAACCCAACCTTAAACCGTTTTTTTTCATTACATTTTATTTTACCATTTATTTTAACTATATTAGTTATAATACACCTAATTTTTTTACATGAAAAAGGTTCTTCAAATCCTCTCGGGCTTAAAAATAAAATTGATAAAATTCCTTTCCATCCATATTTCTCTATAAAAGATTTATTTGGATTCATTATAACCATAATAATTTTTACACTTATTTTATTAAAATCACCTTATTTATTTAGAGATCCAGAAAATTTTACCCCGGCTAATTCTATAATAACACCCCCCCATATTCAACCAGAATGATATTTTCTTTTCGCTTACGCTATTTTACGCTCTATTCCTAATAAATTAGGAGGAGTTTTAGCACTTTTAATATCAATTTTTATTATTTTAATTTTACCATTTTCTGTCAATTCAAAATTTAAGAGTTTATCTATATATACACATAAAAAAATTTTATTTTATATTTTTGTAACCACATTTATTTTATTAACATGAATTGGAGCACGC |
| *Metadelphax propinqua* | ATTACTAATTTACTGTCAGCTATCCCTTACTTAGGAACAACACTTGTCAACTGAATTTGAGGAGGATTCTCAGTAGAAAACCCTACACTTAACCGATTTTTTTCATTTCATTTTATTTTACCTTTTATTCTATGTATTATAATTTTAATACATTTAATTTTTCTACACGAAAAAGGTTCCTCAAACCCCCTAGGATTAAAAAATAAAGTTGACAAAATTAGTTTTCACCCATATTTCTCTATTAAAGATATTTATGGATTCATTGTAGTTATAACATTATTTATATTTATTAATTTTAAAGCTCCGTTTCTATTCAACGACCCTGAAAATTTTACATCGGCTAACCCAATAGTAACACCACCACATATTCAACCTGAATGATATTTTCTTTTTGCTTATGCTATTTTACGTTCAATTCCTAACAAACTAGGAGGAGTTTTGGCTCTTTTAATGTCTATTCTTATCATTTTAACATTACCCTTAACAATAAACTTGAAATTTAAAAGATCAAGTATATATATAATTAAAAAAATTTCTTTTTCAATCTTCTTAAGAACATTTGTATTATTAACTTGAATTGGAGCACGC |
| *Miranus serrulatus* | ATTACAAATTTATTATCTGCAGTACCATATTTAGGAGTTACACTTGTAACATGAATTTGAGGTGGATTCTCAGTAGAAAACCCAACACTTAACCGATTTTTTACTTTTCACTTTATTTTACCTTTTATATTATCATTAATAATTATTATTCACTTAACTTTTCTTCATGAAAAAGGTTCATCTAATCCTCTTGGATTAAAAAATAGAATTGATAAAATTAGATTCCACCCCTACTTTACTATTAAAGACCTACTAGGATTTATTATAATACTAATATTATTTTTACTAATCAATTTTAAAACTCCATATTTATTTAATGATCCAGAAAATTTTTCACCAGCTAATCCTATAGTTACCCCCCCCCATATTCAACCAGAATGATATTTTCTTTTTGCCTATGCAATTTTACSTTCTATTCCTAACAAATTAGGGGGAGTAATAGCCCTTTTAATATCAATTTTGATTATTTTAAGATTACCATTAACTAGAAATTTAAAATTTAAAAGTTCTTCAATATATCCAATTAAAAAATTGTTATTCATGATATTTGTTAGAATTTTTATTTTATTAACATGAATTGGAGCACGA |
| *Monospinodelphax dantur* | ATTACAAATTTAGTATCTGCTATTCCCTATTTAGGAAATACCCTGGTAACATGAATTTGGGGAGGATTTTCAGTAGAAAATCCAACCCTCAACCGATTTTTCTCATTTCACTTTATTTTACCCTTCATTCTCTCTTTATTAATTCTTTTTCACTTAATTTTTTTACATGAAAAAGGATCCTCAAACCCGTTAGGATTAAAAAATAATGTGGATAAAATTAGATTCCATCCTTATTTCACCTTAAAAGATATTTATGGGTTTTTAGTAATTTTATTAATTTTTATAGTTATCAACTTAAAAACACCATTTATATTTAATGATCCAGAAAATTTTACACCTGCAAATCCAATAATTACTCCACCCCATATCCAACCAGAGTGGTATTTCCTTTTTGCTTATGCTATTTTACGTTCTATTCCTAATAAATTAGGGGGGGTTATAGCTCTAGTCTTCTCTATCATTATTATTTTAATTTTACCTTTTACAATAAATTTAAAATTTAAAAGATCAAGTATATATATCCTTAAAAAAATTTTATTCTTTATATTTTGTTCAACTTTTATTATATTAACATGAATTGGAGCACGA |
| *Neocarinodelphax hainanensis* | ATCACAAATTTAATGTCAGCTATCCCCTATCTAGGAAGATTTTTAGTAACTTGAATTTGGGGGGGATTTTCAGTTGATAACCCAACATTAAACCGGTTTTTTTCTTTACACTTTATATTACCTTTTGTGTTAACAGTTATTATTATAATTCACTTAATTTTTTTACATGAAAAGGGGTCATCTAACCCCCTAGGTCTATTAAATAAGATTGATAAAGTATCATTTCATCCCTATTTTTCAATTAAAGATTTATACGGATTTATTGTAGTAATTTTTGGTTTATCCTTAATTTTAATAAAAAGACCCTATATATTTAATGACCCAGAAAATTTTTCCCCCGCTAACTCAATGGTAACTCCTCCCCATATTCAACCTGAATGATATTTTTTATTTGCATACGCTATTTTACGTTCTATTCCTAATAAATTGGGGGGAGTAATTGCACTTCTTATATCAATTTTAATTATTTTAACTCTTCCATTTTCAATAAATTCTAAGTTTAAAAGATTAAGAATATACAGATACAAAAAAAATATGTTTTTTTTATTTGTAGTTATTTTTATATTATTAACATGAATTGGAGCACGC |
| *Nilaparvata lugens* | ATCACAAATTTACTCTCAGCTATCCCCTACTTGGGTAATAGATTAGTTACATGAATTTGAGGGGGATTTTCAGTAGAAAATCCCACTTTAAACCGGTTTTTTTCATTTCATTTCATTTTACCTTTCATCCTTTGTGTAATTATTATATTTCACTTGATCTTTTTACATGAAAAAGGATCATCTAACCCTCTAGGATTAATTAATAAAGTTGATAAAATTAGATTTCACCCCTACTTCACTATTAAAGATATCTTTGGGTTTATAATTATAATATTATTTTTTTTCTTTATTAATATAAAAACCCCCTTTATATTTAATGATCCTGAAAATTTTATGCCGGCAAACCCCATAGTGACACCCCCTCACATTCAACCAGAGTGATATTTTCTATTTGCTTATGCTATTTTACGATCTATTCCTAACAAATTAGGGGGGGTTGTAGCTCTTTTCCTCTCTATTGTTATTATTACAACTTTACCATTTACTTCAAATCTTAAGTTTAAAAGTTCAAGTATATACCCAATTAAAAAAATTTTATTTATTATATTTTGTATAACATTTATATTGTTAACTTGAATTGGAGCACGA |
| *Oecleopsis* sp. | ATTACAAATTTACTTTCAGCAATCCCTTATCTAGGACAAACAATCGTAAAATGAATTTGAGGAGGGTTTTCAGTAGAAAACCCTACATTAAACCGATTTTTCACCCTACACTTCATTTTACCATTTATCTTATTAATAATAGTCATAATACACTTAAATTTTTTACATGAAATAGGATCATCAAACCCCCTAGGTATTAAAAATAATATTGATAAAATTCCATTTCACCCATTCTTCATTAATAAAGACGTTATAGGTTTAGTTTTAATAATATTAATTTTCTCAATTCTTATTCTTAAGGAACCTTTTGTTCTTATAGATCCAGAAAATTTTATTTCAGCAAACCCAATAGTAACTCCACCCCATATTCAACCAGAATGATATTTTTTATTTGCATATGCTATTTTACGTTCAATTCCTAATAAATTAGGAGGAGTAATTGCCCTGTTATTATCAATTTTAATTTTAGTTTCATTACCCTTTTCTATAAAACCAAAATTTAAAAGCATATCATTCTATCCATTAAATAAAATTATATTCTGAACTATAGTTTCAACAACAACTTTATTAACATGAATTGGAGCACAC |
| *Oliarus* sp. | ATTACAAATTTACTTTCAGCAATTCCTTATCTAGGACAAACAATCGTAAAATGAATTTGAGGAGGATTTTCAGTAGAAAACCCTACATTAAATCGATTTTTCACCCTACACTTCATTTTACCATTTATTTTATTAATTATAGTCATGATACACTTAAATTTTTTACATGAAACAGGATCATCAAACCCCCTAGGTATTAAAAATAATATTGATAAAATTCCATTCCACCCATTCTTTATTAATAAAGACGTTATAGGTTTAGTTTTAATAATATTAATTTTCTCAATTATTATTCTTAAGGAACCTTTTATTCTTATAGATCCAGAAAATTTTATTTCAGCAAATCCAATAGTAACTCCACCCCATATTCAACCAGAATGATATTTTTTATTTGCATATGCTATTTTACGCTCAATTCCTAACAAATTAGGAGGAGTAATTGCCCTATTATTATCAATTTTAATTTTAATTTCATTACCCTTTTCCATAAAACCAAAATTTAAAAGTATATCGTTCTATCCATTAAATAAAATTATATTTTGAACTATAGTTTCAACAACAACTTTATTAACATGAATTGGAGCACAC |
| *Opiconsiva albicollis* | ATTACTAACCTCTTATCTGCAATTCCTTATGTAGGAATCACCTTAGTTAATTGAATCTGAGGGGGGTTTTCAGTTGAAAACCCTACATTAAATCGATTTTTTTCATTCCATTTTATTTTACCTTTCATCTTATGTTTAATAATTTTAATACACTTAATTTTTTTACATGAAAAAGGTTCATCAAACCCCTTGGGATTAAAAAATAAAATTGATAAAATTAGATTCCACCCCTACTTCTCTATTAAGGATATATTCGGATTTTCAATTGTAACAATTGTATTTATTTATGTTAACTTTAAAACCCCCTTTATTTTTAATGATCCAGAAAATTTTAATCCTGCTAACCCTATGATTACTCCCCCACACATTCAACCCGAATGATACTTCCTTTTTGCTTACGCAATTTTACGATCTATTCCAAACAAGCTAGGGGGAGTTGTGGCTCTTTTAATATCAATTTTAATTATTTTAATTTTACCTTTTACAATAAACCTAAAATTTAAAAGAACTAGAATATATAATTTTAAAAAAATTTATTTTTTAGTTTTTTGCAGAACTTTTATTATTTTAACTTGAATTGGAGCACGA |
| *Palego simulator* | ATTACTAATCTTTTATCTGCCATTCCATATCTAGGTAACATATTAGTAACATGGATTTGGGGGGGATTCTCAGTTGAAAATCCAACTCTAAATCGATTTTTCTCCTTTCACTTTATTTTACCGTTTATTTTAACCACTTTAATTTTGTTTCATTTGATTTTTTTACATGAAAAAGGTTCCTCAAACCCCCTTGGACTAAGGAATAAAGTTGATAAAATCAGTTTTCACCCATACTTTACACTAAAAGATTTATATGGATTCACAATCATTTGTATAGTATTTATTTTCATCAATTTAAAGACTCCGTTTTTATTCAATGACCCAGAAAATTTTACGCCTGCAAATCCAATAATTACACCTCCTCATATTCAACCAGAATGATACTTTCTTTTTGCCTACGCAATTTTACGTTCAATTCCTAATAAATTGGGGGGTGTATTAGCACTAGTTTTTTCAATTTTAATTATCTTAATTTTACCATTTTCAATAAACCTAAAATTTAAAAGATCAAGAATATACACTTTTAAAAAAATTTTATTTTACATATTTTGCTCTACATTCATTTTATTAACCTGAATTGGAGCACGC |
| *Pentastiridius* sp. | ATTACCAATTTACTTTCTGCAATTCCATATATTGGTCAAATAATTGTAAAATGAATCTGAGGAGGATTTTCAGTTGAAAATCCAACCTTAAATCGATTTTTTACTCTTCACTTCATTTTACCATTTATTTTATTAACTCTGATTATTCTACACTTAAATTTTTTACATGAAACAGGTTCATCAAACCCATTAGGATTAAAAAATAATATTGATAAAATTCCTTTTCATCCATTTTTTATTTATAAAGATATTATAGGAATAATTTTAACCCTAATAATTTTCTCATTTATAATTTTAAAAGAACCATTTATCCTAATAGATCCAGAAAACTTTACCCCAGCAAATCCAATAGTTACACCACCCCATATTCAACCAGAATGATATTTTTTATTTGCCTATGCAATTTTACGATCTATTCCTAATAAATTAGGAGGAGTAATTGCTCTACTTATATCAATTCTAATTTTATTAACCCTTCCATTTTCAATAAACCCTAAATTTAAAAGATTATATTTTTATCCAATAAACAAAATAATATTTTGATTATTAATTTCATCTACAATTTTATTAACATGAATTGGAGCACGC |
| *Purohita sinica* | ATCACTAATTTGTTATCAGCAGTCCCTTATCTTGGATTAAATTTAGTAAACTGAATTTGAGGAGGATTTTCAGTTGATAACCCTACTTTAAATCGATTTTTCTCTTTACATTTTATTTTACCATTTATTTTAATTGTTATAATTATTTTTCATTTAATTTTTTTACATGAAAGGGGTTCATCTAATCCCCTTGGTTTAAAAAATAAAGTTGATAAAGTTTCTTTTCATCCTTACTTTTTAATTAAAGATGTATACGGATTTATAGTTATATTTCTATTTTTCTCAATTGTTATTTTAAAAACACCTTTTATCTTTAATGACCCAGAAAATTTTTCTCCAGCCAATGCTATAATGACACCACCTCATATTCAACCTGAATGATATTTTTTGTTTGCTTACGCTATTTTACGTTCTATTCCTAATAAACTAGGTGGTGTTGTGGCTCTTTTAATATCAATTTTAATTATTTTAACTTTGTCTTTAACTATAAATTTAAAATTTAAAAGATTAAGAATATATTTCTATAAAAAAATTATATTTTTTTCATTTTTATCAATTTTTTTTTTATTAACTTGAATTGGAGCACGA |
| *Purohita taiwanensis* | ATTACTAATTTAATATCAGCAGTTCCTTATTTTGGGTTAAATTTAGTAAACTGAGTTTGAGGAGGGTTTTCAGTTGATAACCCCACTTTAAACCGATTTTTTTCTTTACATTTTATTTTACCATTTATCTTAACTGTTTTAATTATTTTTCATTTAATTTTTCTACATGAAAATGGATCTTCAAATCCTCTTGGTTTAAAAAATAAAATTGATAAAATTTCTTTTCACCCTTACTTTTCTATTAAAGATTTATACGGATTTATATTATTATTTCTTTTTTTTTCATCAGTAATTTTAAAAGCACCCTATATTTTTAATGACCCAGAAAATTTTTCACCAGCAAATTCTATAATTACCCCCCTTCATATTCAACCTGAATGATACTTTTTATTTGCTTACGCTATTTTACGTTCTATCCCTAACAAGCTGGGGGGAGTTGTAGCTCTTTTAATATCAATTTTAATTATCTTATCATTATCATTTACAATAAATTTAAAGTTTAAAAGTTTAAGAATGTATTTTTATAAAAAAATTTTATTTTTCTCCTTTTTATCAATTTTTTTTTTATTAACATGAATTGGAGCACGA |
| *Purohita theognis* | ATTACAAATCTTATATCAGCTATTCCTTACTTAGGAACCAATTTAGTAACCTGAATTTGAGGGGGTTTTTCTGTTGATAACCCTACATTAAATCGATTTTTTTCCCTACATTTCTTGATACCATTCATTTTAACAGTAATAATTTTGTTTCATTTAATTTTTTTACATGAAAAAGGATCATCTAATCCTCTTGGATTAAAAAATAAAATTGATAAAATTTCATTTCATCCGTATTTCTCAATTAAAGACTTATATGGATTTATTTTTATATTTTTAATATTCTCAATTATTGTATTAAAAATACCCTATATATTTAATGATCCAGAAAATTTTACTCCTGCTAACTCAATAATTACACCACCCCATATTCAACCTGAATGATATTTTCTATTTGCTTATGCTATTTTACGATCTATTCCTAATAAATTAGGTGGTGTTTTAGCACTTTTATTATCAATTCTTATTATTTTAACTTTACCATTTACAATAAATTTAAAATTTAAAAGTTTAAGTATATATAAACATAAAAAAATTTTATTCTTTTTATTTATTTCAATTTTTTTCTTATTAACTTGAATTGGAGCACGC |
| *Reptalus squadricinctus* | ATTACAAACTTATTATCTGCAATTCCCTATATTGGTCAGACAATTGTAAAATGAATCTGAGGAGGGTTTTCAGTTGAAAACCCAACTTTAAACCGATTTTTTACACTACATTTTATTCTACCATTCATTTTATTAACAATAATTATTCTTCATTTAAACTTTTTACATGAAACAGGTTCCTCTAACCCTATTGGAATAAAAAATAATGTAGATAAAATCCCATTCCACCCATTTTTCATTTATAAAGATTTAATAGGTATAGTTATTCTTCTTTCAACCTTCTCAATTATAATTTTAAAAGAACCATTCATTTTAATAGATCCAGAAAATTTCACTCCAGCTAATCCAATAGTTACACCTCCACATATTCAACCTGAATGATACTTCTTATTTGCCTACGCAATTTTACGATCAATTCCCAATAAGTTAGGAGGAGTAATTGCCCTACTTATATCAATTTTAATTTTATTAACCTTACCATTTTCAATAAAACCAAAATTCAAAAGAACATCATTTTACCCCCTAAATAAAATTATATACTGAATATTAATTTCTTCAACAATTTTACTAACTTGAATTGGAGCACAC |
| *Rhombotoya psedomigripennis* | ATTACAAATTTACTTTCAGCCATTCCATATTTAGGGAATACATTAGTGACATGAATTTGGGGAGGATTTTCAGTAGAGAATCCTACCCTTAATCGTTTCTTTTCATTTCATTTTATTTTACCTTTTATTTTAACTGTAATAATTCTTATACACTTAATTTTCCTTCATGAAAAAGGATCATCTAACCCCCTTGGATTAAAAAATAAAGTTGATAAAATTAGATTTCATCCATACTTCACACTAAAAGATATCTTTGGTTTTATAATTATTATTTCTACATTTATTTTTATCAACTGCAAAACTCCATTTTTATTTAATGACCCAGAAAATTTTACTCCAGCTAATCCCATAATCACACCTCCACATATTCAACCTGAATGATATTTTCTTTTTGCATATGCCATTCTACGTTCTATTCCTAATAAATTGGGAGGAGTTATTGCACTTTTATTATCAATTTTAATCATTTTAATTCTTCCTTTCACAATAAATTTTAAATTTAAAAGTTCTAGAATATACTTATCTAAAAAAATTTTATTCTTCTTATTTTGTTCAACTTTTATTTTATTAACCTGAATTGGAGCACGA |
| *Saccharosydne procerus* | ATTACTAATTTAATATCAGCAATCCCCTATTTAGGAATATCATTAGTAAATTGAATTTGAGGAGGATTTTCCGTAGAAAATCCTACACTTAATCGATTTTTTTCTATTCATTTTATTTTACCATTTATTTTAACTCTAATAATTTTAATTCATTTATTATTTTTACATGAAAAAGGTTCTTCCAATCCTCTTGGATTAAAAAATTCAATTGATAAAATTTCTTTTCACCCTTATTTTACTACAAAAGATATTTTAGGATTTTTAATTGTTCTAATAATATTTTCAATAATTTTATTTTATAGACCTTTTATATTCAACGACCCAGAAAACTTTACCCCCGCAAATTTTATAAATACACCACCCCATATTCAACCCGAATGATATTTTTTATTTGCCTATGCAATTCTACGCTCAATTCCTAATAAATTAGGGGGAGTGATTGCCCTAGTAATATCTATTCTTATTATTTTAACTCTCCCATTTACTAATAACCTTAAATTCAAAAGATCAAGTATATATACTATTAAAAAAACTATTTTTCTAATATTTATTTCAATTTTTATTTTATTAACATGAATTGGAGCACGC |
| *Sardia rostrota* | ATTACTAACCTATTATCAGCTATTCCTTACTTAGGGGGAAGAATAGTTAACTGAATTTGGGGGGGGTTCTCGGTAGATAACCCTACCCTAAATCGATTCTTTTCATTTCATTTTATTTTACCTTTTATTTTATGCCTAATAGTTATATTCCACTTAATCTTTTTACATGAAAGGGGGTCTTCAAACCCCCTTGGGTTAAGAAATAAAGTTGATAAAATTAGTTTCCACCCATATTTCTCTATTAAAGATATTTACGGGTTCATACTTGTAATTTTAATATTTTTATTTGTTAATTTTAAAACACCTTTCATATTTAATGACCCTGAAAACTTTTCACCAGCTAATCCAATAGTGACTCCCCCCCATATCCAACCTGAATGGTATTTTTTGTTTGCTTACGCAATTTTACGTTCTATCCCTAACAAACTGGGGGGGGTTTTAGCTCTTATTATATCAATTATCATTATCTTAAGATTACCTCTTACAATAAACCTAAAATTTAAAAGATCAAGAATATACATTTTAAAAAAAATTTTCTTTCTAATTTTTTGCAGAACATTTTTGCTTTTAACTTGAATTGGAGCACGC |
| *Shadelphax eforiae* | ATCACCAACCTTCTTTCTGCTATTCCTTACCTAGGAGAAACTTTAGTTACATGAATCTGGGGGGGATTCTCCGTTGAAAACCCCACTCTAAACCGATTTTTCTCATTCCACTTTATTCTCCCTTTCATTCTGAGAGGCATGATCTTATTTCATCTAATCTTTTTACACGAAAAGGGATCTTCAAATCCCTTAGGTCTCAAAAATAAAATTGATAAAATTAGATTCCACCCTTATTTTTCTGTGAAAGATATTTATGGTTTTATAATTTTGATACTGATTTTCATTTTAATTAACTTAAAAACTCCCTTTCTATTTAATGATCCTGAAAATTTTACCCCAGCCAACCCAATAGTAACCCCCCCTCATATTCAACCTGAATGATATTTTTTATTTGCCTATGCAATCTTACGGTCTATCCCCAACAAACTTGGGGGAGTCGTAGCGTTATTTATATCAATTATTATTATTTTAACACTCCCCCTTACTATAAATTTAAAATTTAAAAGCTCCAGAATATACTTAACAAAAAAAATCACATTCATAATTTTTTGTTTAACATTTATGATATTAACATGAATTGGAGCACGC |
| *Sogata hakonensis* | ATTACTAACCTTTTATCAGCAATCCCATATGTAGGAATTACCCTAGTAACATGAATCTGAGGAGGCTTCTCAGTAGAAAACCCAACACTTAACCGATTTTTTACGTTTCATTTCATTTTACCCTTTATGTTAATATTAATAATTTTAATCCACTTAATTTTACTTCATGAAAAAGGATCTTCTAATCCTCTTGGGTTAAAAAATAAAATTGATAAGATTAGTTTTCACCCATATTTTTCAATTAAGGACTTACTGGGATTCATTATAATAATAATTTTATTTTTATTAATTAATTTTAAAACACCCTATTTATTTAATGACCCAGAAAATTTCACCCCAGCAAATCCCATAATTACACCTCCCCATATTCAACCAGAATGATATTTCCTTTTTGCTTACGCAATTTTACGTTCAATTCCTAACAAATTAGGGGGTGTAGTAGCTCTTTTAATGTCAATTTTAATTATTTTAACTTTACCATTAACAATAAATTTAAAATTTAAAAGATCATCTATATACACAACAAAAAAAATTTTATTTTTTATATTTTGTAGCACATTCATTTTATTAACATGAATTGGAGCACGA |
| *Sogata nigrifrons* | ATTACTAACCTTTTATCAGCAATCCCATATATAGGAATTACCCTAGTAACATGAATCTGGGGGGGCTTCTCAGTAGAAAACCCAACACTTAACCGATTTTTCACGTTTCATTTCATTTTACCCTTTATGTTAATATTAATAATTTTAATTCACTTAATTTTACTTCATGAAAAAGGGTCTTCTAATCCTCTTGGATTAAAAAATAAAATTGATAAGATTAGTTTCCACCCATATTTTTCAATTAAAGACTTACTGGGATTCATAATAATAATAATTTTATTTTTATTAATTAATTTTAAAACACCCTATTTATTTAATGACCCAGAAAATTTCACCCCAGCAAATCCCATAATTACACCTCCCCATATTCAACCAGAATGATATTTCCTTTTTGCTTACGCAATTTTACGTTCAATTCCTAATAAATTAGGGGGTGTGGTAGCTCTTTTAATGTCAATTTTAATCATTTTAACTTTACCATTAACAATAAATTTAAAATTTAAAAGATCATCTATATACACAATAAAAAAAATTTTATTTTTCATATTTTGTAGCACATTCATTTTATTAACATGAATTGGAGCACGC |
| *Sogatella furcifera* | ATCACTAACCTATTATCGGCTGTTCCTTATTTAGGTGATACTTTAGTGAGTTGAATTTGAGGGGGGTTCTCTGTTGAAAATCCTACTCTTAACCGATTCTTTTCTTTTCATTTTATTTTACCTTTTATTTTATGTTTAATAATTTTAATTCACTTAATCTTCTTACATGAAAAAGGGTCATCAAACCCCCTTGGGTTAAAGAATAAAGTAGACAAAATTAGTTTTCACCCTTATTTTTCAATTAAAGACATTTATGGATTTATAATTGTTATTTTAATATTTTTATTTGTTAATTTTAAAACACCTTTTCTATTCAATGACCCTGAAAATTTCACACCAGCCAACCCTATAGTGACTCCTCCTCATATCCAGCCTGAATGATACTTTTTGTTTGCTTATGCAATTTTACGTTCTATCCCTAACAAACTAGGAGGAGTTTTAGCTCTTGTAATGTCAATTGTTATTATTTTAAGATTACCATTAACTATAAATTTAAAATTTAAAAGATCTAGAATATATTTTATAAAAAAAATTTTGTTTATAATTTTTTGCAGAACATTTTTTCTTCTCACCTGAATTGGAGCACGA |
| *Specinervures basifusca* | ATTACAAATTTAATATCTGCTATTCCCTATTTAGGCTCATCAGTAGTTAATTGAGTATGAGGCGGGTTTTCAGTTGATAACCCTACATTAAATCGATTCTTTTCACTTCATTTTATTTTACCATTTGTATTAATAATATTAATTTTATTACACCTTATTTTTCTTCATGAAAAAGGTTCATCAAATCCACTAGGACTAAAAAATAAAATAGATAAAATTTCATTCCACCCATATTTTTCTATTAAGGACCTATTAGGATTTTTATTAACTTTATTTTTTTTTTCAATTATTTTATTCAAAACACCATTTTTATTTAATGATCCAGAAAATTTTACACCAGCTAATCCTATATTAACTCCCCCTCATATTCAACCAGAATGATATTTTCTATTTGCTTATGCTATTTTACGATCAATTCCTAACAAACTGGGAGGGGTTTTAGCATTACTATTATCAATCTTAATTATTTTAACTTTACCATTTACTATAAAATCAAAATTTAAAAGAAGAAGAATATATTTACATAAGAAAATCTTATTCTTAATTTTTATTTCAATTTTTATTTTATTAACATGAATTGGAGCACGA |
| *Stenocranus montanus* | ATTACAAATTTAATATCGGCAATTCCCTATCTAGGACAAACAATAGTAAATTGAGTATGAGGGGGATTCTCAGTAGATAATCCCACACTTAACCGATTTTTCTCTTTACATTTCATTTTACCATTCATATTAACAGTAATAATTTTATTCCACCTAATTTTTCTTCATGAAAGGGGGTCTTCAAATCCTTTAGGGTTAAAAAATAAAATTGATAAAATTCCCTTCCACCCATTTTTCACTGTTAAAGACATTTTTGGGTTTATTTTAATTTTTCTAGGATTTTCAGTTATTTTATTTAAAACACCATTTTTATTTAATGACCCAGAAAATTTTTCTCCTGCTAACCCTATAATTACACCTCCTCATATTCAACCAGAATGATACTTCTTATTTGCTTATGCTATTCTTCGTTCAATCCCAAATAAACTAGGAGGAGTTATAGCTCTTTTATTGTCAATTCTTGTAATTTTAACTTTACCATTTTTAACCAACCAAAAATTTAAAGGATCATACATGTACATAACAAAAAAAATCACTTTTTGAATTTTCATTTGTTCATTCATTTTACTAACATGAATTGGAGCACAC |
| *Terauchiana singularis* | ATTACAAACTTAATGTCAGCAATTCCTTACATAGGATTAACTCTAGTAAATTGAGTCTGGGGGGGATTTTCAGTTGATAACCCTACCTTAAACCGATTCTTTTCATTACACTTTATTTTACCATTTTTATTAATGTTAATAATTATATTTCACCTAATTTTTCTCCATGAAAAAGGATCATCAAATCCCTTAGGATTAAAAAATAAAATTGATAAAATTCCCTTCCACCCATTCTTTACTATTAAGGATATTTTTGGATTTATAATTGTATTATTTTTATTTTCAATTATCTTACTAAAAACCCCATTTTTATTTAACGACCCAGAAAATTTCACTCCAGCTAATCCAATAGTAACACCCCCCCATATTCAACCAGAATGATATTTTTTATTTGCTTATGCTATTCTACGGTCTATTCCAAATAAACTAGGGGGWGTAATTGCTTTAGTTTTTTCTATTCTTGTAATCTTAACATTACCCTTATTAACTAAACAAAAATTTAAGGGATCAAACATATATTTAACAAAAAAAATTACTTTCTGAATATTTATTTGCTCATTTATTATATTAACATGAATTGGAGCACGA |
| *Terthron albovittatum* | ATTACCAATTTATTGTCAGCTATCCCATATTTAGGTATAACTCTTGTCAATTGAATCTGAGGGGGCTTTTCGGTTGAAAACCCTACTTTAAATCGATTTTTTTCTTTTCATTTTATTTTACCTTTTATTTTATGTGTAATAATTTTATTTCATTTAATTTTTCTACATGAGAAAGGGTCTTCCAATCCCTTAGGGTTAAAAAACAAGATTGATAAAATTACCTTCCACCCGTATTTCTCAATTAAGGATATTTATGGGTTTATTGTAATTTTATTATTGTTTATATTTATTAATTTTAAAACACCATTTTTATTTAATGATCCTGAAAACTTTACACCAGCTAACCCAATGGTTACCCCCCCTCATATCCAACCTGAATGATATTTTTTATTTGCTTACGCTATCTTACGTTCTATTCCTAACAAATTAGGAGGAGTTTTAGCTCTATTTTTATCTATTATTATTATTTTAACATTGCCATTAACAACAAATCTTAAATTTAAGAGATCAAGTATATACCCTTTAAAAAAAATTTTTTTTTCAATATTTTGTTGTACTTTTGTTCTCTTGACCTGAATTGGAGCACGA |
| *Toya terryi* | ATTACAAATTTACTGTCAGCTATCCCTTATTTAGGAACAACTCTTGTAAACTGAATTTGAGGTGGATTTTCAGTTGAAAATCCTACCTTAAATCGATTTTTTTCATTTCACTTTATTTTACCCTTTATTTTGTGTGTTATAATTATGTTTCACTTAATTTTTTTACATGAGAAGGGTTCATCAAACCCCTTAGGGTTAAAAAATAAAGTTGATAAAATTAGTTTTCATCCATATTTTTCTATTAAAGATATTTATGGATTTGTAGTAGTAGTCACATTATTTCTATTTATTAATTTTAAAACTCCATTCCTATTTAACGACCCGGAAAATTTTTCTCCTGCTAACCCAATAGTAACACCCCCCCACATTCAACCTGAATGATATTTTCTTTTTGCTTATGCTATTTTACGGTCTATCCCCAACAAGCTAGGAGGAGTTTTAGCTCTTTTAATATCTATTTTAATTATTTTAACATTGCCATTTACTATAAATATAAAATTTAAAAGATCAAGAATATACATAGTTAAAAAAATTTCATTTTCAATTTTTTGTAGTACGTTTGTACTTTTAACTTGAATTGGAGCACGA |
| *Toyoides albipennis* | ATCACTAATTTACTTTCAGCAATTCCATATTTAGGCAATCTTTTAGTTACATGAATTTGGGGGGGCTTCTCAGTAGAAAACCCTACTCTTAACCGATTTTTTTCTTTACATTTTATTTTACCATTTATACTTTGTTTAATAATTATATTTCACTTAATTATACTGCATGAAAAAGGATCTTCAAACCCACTAGGTTTAATTAACAAAATTGATAAAATCAGTTTCCATCCTTATTTTACATTAAAAGATTTAATTGGATTTATTATTGTATTTATAACCCTTACTTTTATTAATTTAAAAACACCATTCTTATTCAATGATCCAGAAAATTTCACTCCTGCTAACCCTATAATTACACCTCCCCATATTCAACCTGAATGATACTTCCTTTTTGCTTACGCAATTTTACGTTCTATTCCTAATAAATTAGGAGGGGTTATAGCACTATTTTTCTCAATTCTAATCATTTTAACACTTCCTTTTTCATCAAATTTAAAATTTAAAAGCTCAAGAATATACTTAACTAAAAAAATTTTATTTTATTTATTTACTGCAAATTTTATTTTATTAACCTGAATTGGAGCACGA |
| *Tropidocephala brunnipennis* | ATTACAAATTTATTATCAGCAATTCCATACTTAGGAAAATTTTTAGTTAATTGAATTTGAGGAGGATTTTCTGTAGATAACCCAACATTAAATCGATTTTTTTCATTACATTTTTTATTACCTTTTATATTAACAATAATAATTTTAATACACCTTATTTTTTTACATGAAAAAGGTTCATCTAATCCTCTTGGTTTAAAAAATAAAATTGATAAAATTTCATTCCATCCATATTTTTCAATTAAAGACATTTATGGATTTTGTATAACATTATTAATATTCACAATCATTTTATTTAAATTTCCATTTATGTTTAATGATCCAGAAAATTTTAGCCCAGCAAATTCTATAGTAACTCCACCACATATTCAACCAGAATGATATTTTTTATTTGCTTATGCTATTTTACGCTCAATTCCAAATAAACTGGGGGGGGTAATAGCATTATTAATATCAATCTTAATTATTTTAACTCTTCCATTTACAATAAATGATAAATTTAAAAGACTTAATATATATATTCATAAAAAAATTTTATTTTTATTATTCACCGCAATTTTCATTTTATTAACATGAATTGGAGCACGA |
| *Tropidocephala festiva* | ATTACAAACTTAATATCAGCTATTCCATATTTAGGAAAATTTTTAGTTAATTGAATTTGAGGGGGATTTTCTGTAGATAACCCAACATTAAATCGATTTTTCTCTTTACATTTTTTATTACCTTTTATCTTAACTATAATAATTTTAATACACCTTATTTTTTTACATGAAAGGGGGTCATCCAATCCACTTGGATTAAAAAATAAAATTGACAAAATTATATTCCATCCATATTTTTCAATTAAAGATATTTATGGATTTTCTATAACATTACTTATATTTACAATTATTTTATTTAAATTTCCATTTGTATTTAATGACCCAGAAAATTTTACCCCAGCAAATTCAATAGTTACCCCCCCCCACATTCAACCTGAATGATATTTTCTATTTGCTTACGCCATTTTACGTTCAATTCCTAACAAACTAGGTGGAGTAATAGCATTATTAATATCAATTTTAATTATTTTAACTCTTCCATTTACAATAAATGATAAATTTAAAAGACTTCACATATATATTCATAAAAAAATTTTATTTTTATTATTTATTACAACTTTCATTTTATTAACATGAATTGGAGCACGA |
| *Tropidocephala* sp. | ATTACAAATTTATTATCAGCAATCCCATATTTAGGAAAATTTTTAGTTAACTGAATTTGAGGAGGATTTTCTGTAGATAACCCAACATTAAATCGATTTTTTTCATTACATTTTTTGTTACCTTTCATATTAACAATAATAATTTTAATACACCTTATTTTTTTACATGAAAAAGGGTCATCTAATCCACTTGGTTTAAAAAATAAAATTGATAAAATTTCATTTCATCCATATTTTTCAATTAAAGATATTTATGGATTTTGTGTAACATTATTAATATTCACAATCATTTTATTTAAATTTCCATTTTTATTTAATGATCCAGAAAATTTTACTCCAGCAAATTCTATAGTTACCCCACCACATATTCAACCAGAATGATACTTCTTATTTGCTTATGCTATTTTACGCTCAATTCCAAATAAACTGGGGGGAGTAATAGCATTATTAATATCAATTTTAATTATTTTAACTCTTCCATTTACAATAAATGATAAATTTAAAAGACTTAACATGTACATTCATAAAAAAATTTTATTTTTATTATTTACCGGAATTTTTATTTTATTGACATGAATTGGAGCACGC |

**Supplementary** **Table S4** The sequence alignments of *cox1*.

| Taxa | The sequence alignments of *cox1* |
| --- | --- |
| *Aoyuanus furcatus* | GAAGTTTACATCTTAATCCTACCAGGATTTGGATTAATTTCACATATTATTATACAAGAAAGAGGAAAAAAGGAAACATTTGGATCAATCGGAATAATTTATGCAATAATCGCTATTGGAATTTTAGGATTTATTGTTTGAGCCCACCATATATTTACAGTAGGTATAGATATCGACACACGAGCTTATTTTACTTCAGCAACTATAATTATTGCTGTACCTACAGGTATTAAAATTTTTAGATGAATAGCAACAATCTATGGCTCTAAAATTTTACTCTCACCCCAAATAATTTGAGCCATAGGATTTATTTTACTATTTACAGTAGGAGGATTAACAGGAGTAATACTAGCAAATTCATCAATCGACATTGTATTACATGATACCTACTATGTAGTAGCTCACTTCCACTATGTTTTATCTATAGGAGCAGTTTTTACAATCATTGCAAGATTCATTCACTGATACCCCCTAATTACAGGAACCTCAATAAACAATAAATGACTAAAAATTCAATTTTTTTCAATGTTCTTAGGA |
| *Arcofaciella verrucosa* | ---------------------------------------------------------------------------ACATTTGGTAATATCGGAATAATTTATGCAATAATTGCTATTGGTGTTTTAGGATTTATTGTATGAGCCCACCATATATTCACTGTTGGAATAGATATTGATACACGAGCCTATTTTACTTCAGCCACAATAATTATTGCAGTCCCGACAGGAATCAAAATTTTTAGATGACTAGCCACAATTCACGGATCCAAAATTGTATTTTCACCTCAAATAATTTGATCAACAGGATTTATTTTTCTCTTCACAATTGGAGGATTAACAGGAATTATTTTAGCTAACTCATCAATTGACATTATTTTACATGATACATACTATGTTGTTGCCCACTTCCACTATGTATTATCAATAGGAGCAGTATTCACAATTATTTCAAGATTTATCAACTGATACCCCGTACTGACAGGTGTTTCAATAAATTATAAATGACTAAAAATTCAATTTTTATCAATATTTACAGGT |
| *Arcofacies maculatipennis* | GAAGTTTACATCTTAATTTTACCAGGATTTGGATTAATTTCACATATTATTATACAAGAAAGAGGAAAAAAGGAAGCATTTGGAACTATCGGGATAATTTACGCAATAATCGCTATTGGAATTCTTGGATTCATTGTTTGAGCTCACCATATATTTACAGTTGGAATAGACATCGACACTCGAGCTTACTTCACTTCAGCAACTATAATTATTGCTGTACCAACAGGTATTAAAATTTTTAGCTGATTAGCAACAATCCACGGATCAAAAATACTATTTTCACCACAAATAATTTGATCAACAGGATTCATTTTTCTTTTTACAATTGGGGGTTTAACCGGAGTAATTCTAGCTAATTCATCAATCGATATTATTTTACATGATACCTATTATGTAGTTGCTCACTTTCATTATGTTCTTTCCATAGGAGCTGTATTCACAATCATCTCAAGATTTATTAACTGATATCCAACATTTACAGGATTAATAATAAACAATAAATGACTAAAAATTCAATTTATATCAATATTTATCGGA |
| *Arcofacies strigatipennis* | GAAGTTTACATCTTAATTTTACCTGGATTCGGATTAATTTCACATATTATTATACAAGAAAGAGGTAAAAAGGAAACTTTTGGTTCAATTGGGATAATTTACGCTATAATTGCTATTGGAATTTTAGGATTTATTGTATGAGCCCACCACATATTCACAGTAGGTATAGACATTGATACACGAGCTTACTTTACCTCCGCAACTATAATCATTGCAGTCCCCACTGGAATTAAAATTTTTAGATGACTAGCCACAATTCACGGTTCAAAAATAAATTTTTCACCTCAAATAATTTGATCAACAGGATTTATTTTTCTATTCACTATTGGAGGCTTAACAGGAGTAATTTTAGCCAATTCATCAATTGACATCATTTTACATGATACATATTATGTTGTAGCCCACTTCCATTATGTACTATCAATAGGAGCTGTTTTTACTATTATTTCAAGATTCATTAATTGATACCCCCTATTTACAGGATTAATAATAAATAATAAATGACTAAAAATCCAATTTACATCAATATTCATTGGA |
| *Bakerella minuta* | GAAGTTTACATCCTAATTTTACCAGGATTTGGTTTAATTTCACACATTATCATACAAGAAAGAGGAAAAAAAGAAACTTTTGGTTCAATTGGAATAATTTACGCAATAATTTCTATCGGAGTATTAGGTTTCATCGTTTGAGCCCACCATATATTTACTGTCGGAATAGATATTGACACCCGTGCTTATTTTACCTCAGCAACCATAATTATTGCAGTCCCAACTGGAATTAAAATTTTCAGATGAATAGCAACAATCTATGGAACTAAAATCTACTTTTCCCCTCAAATAATTTGATCAATGGGGTTCATTTTATTATTCACAATTGGTGGATTAACAGGTGTAATATTAGCAAATTCATCTATTGATGTAATTCTACATGACACATATTATGTAGTAGCACACTTCCACTATGTACTTTCGATAGGTGCAGTATTCACCATCATTGCAAGATTTATCCACTGATACCCCCTAATTACAGGGGTAAAAATAAATAATAAATGATTAAAAATTCAATTTATTTCAATATTTACAGGA |
| *Bambusiphaga fascia* | GAAGTTTACATTTTAATTTTACCAGGATTTGGATTAATTTCACATATTATTATACAAGAAAGAGGAAAAAAGGAAACATTTGGTACAATCGGAATAATCTACGCAATAATCGCCATTGGAATTTTAGGATTTATTGTATGAGCTCACCATATATTCACTGTAGGTATAGATATCGACACACGAGCTTATTTTACCTCAGCTACTATAATTATTGCTGTACCCACAGGGATTAAAATTTTTAGATGATTAGCAACTATCCATGGATCAAAAATAACATTTTCACCCCAAATAATTTGATCAACCGGATTTATTTTCCTTTTTACAATCGGAGGACTAACAGGAGTAATTTTAGCTAATTCATCTATTGATATTATTTTACACGATACATATTATGTAGTAGCACATTTTCATTACGTATTATCAATAGGAGCTGTATTTACAATTATTGCAAGATTTATTAATTGATACCCAGTATTTACAGGAATTTCAATAAACAATAAATGATTAAAAATTCAATTTTTTTCAATATTTATGGGA |
| *Bambusiphaga hainanensis* | GAAGTCTACATTCTAATCCTCCCAGGATTTGGATTAATTTCACATATTATTATGCAAGAAAGAGGAAAAAAAGAAACATTTGGAACAATTGGAATAATCTATGCTATAATTGCTATTGGTATCCTAGGATTTATTGTGTGAGCCCATCACATATTTACTGTAGGAATAGATATTGATACACGAGCCTACTTTACTTCAGCCACCATAATTATTGCTGTCCCCACAGGAATTAAAATTTTTAGATGACTAGCAACAATTCACGGGTCAAAAATTTCATTTTCACCCCAAATAATTTGATCGACAGGATTTATTTTTCTATTTACAATCGGGGGATTAACCGGAGTAATCCTAGCAAATTCATCAATTGATATTATTTTACATGATACATACTATGTAGTTGCCCATTTTCACTATGTACTATCTATAGGAGCAGTATTTACAATCATTGCAAGATTTATTAACTGATACCCAGTAATTACAGGAATCTCAATAAACAATAAATGACTTAAAATCCAATTTTTATCCATATTTATAGGA |
| *Bambusiphaga kunmingensis* | GAAGTTTACATTTTAATTTTACCAGGATTCGGGTTAATTTCACATATCATTATGCAAGAAAGAGGTAAAAAAGAAACATTTGGAACAATTGGAATAATCTACGCAATAATCTCCATCGGGATTTTAGGATTTATTGTTTGGGCCCATCATATATTCACCGTTGGTATAGACATCGATACGCGAGCTTATTTCACCTCAGCAACTATAATTATTGCTGTACCAACCGGGATTAAAATTTTTAGTTGACTAGCAACTATCCATGGATCAAAAATATCATTTTCACCTCAAATAATTTGGTCAACCGGATTCATTTTTCTTTTCACAATCGGCGGATTAACGGGAGTAATTTTAGCTAACTCCTCTATTGATATTATTTTACACGACACATATTATGTTGTAGCACATTTTCATTATGTTTTATCAATAGGAGCTGTTTTTACAATTATCGCAAGATTTATCAATTGATACCCAGTGTTCACGGGGATACAGATAAATAATAAATGACTAAAAATTCAATTTTTTTCAATATTTATCGGA |
| *Bambusiphaga luodianensis* | GAAGTTTACATCTTAATCTTACCAGGATTTGGATTAATTTCCCATATCATTATGCAAGAGAGAGGAAAAAAAGAAACATTTGGAACAATTGGTATGATTTACGCAATAATAGCTATTGGTATCCTAGGATTCATCGTATGAGCACATCACATATTTACCGTAGGTATAGACATTGACACCCGAGCTTATTTCACTTCAGCCACTATAATTATTGCTGTCCCCACAGGAATTAAAATTTTTAGTTGACTGGCAACTATTCACGGTTCAAAAATCCTTCTTTCACCCCAAATAATCTGATCAACAGGATTTATTTTCCTATTTACAATTGGAGGATTAACTGGGGTTATCCTAGCAAATTCATCAATTGATATTATTTTACATGACACATACTATGTAGTAGCCCATTTCCATTATGTTTTATCCATAGGAGCTGTATTCACCATTATCGCCAGATTTATTAACTGATATCCAGTGATTACTGGTGTAACAATAAACAAAAAATGACTAAAAATCCAATTTTTAACAATATTTATAGGA |
| *Bambusiphaga maculata* | GAAGTTTATATTTTAATTTTACCAGGATTCGGAYTAATCTCACATATTATTATACAAGAAAGAGGAAAAAAAGAAACATTTGGTACAATTGGAATAATTTATGCAATAATTGCTATTGGAGTTTTAGGATTTATTGTATGAGCCCATCATATATTTACTGTAGGTATAGATATTGATACACGAGCTTATTTTACTTCAGCAACTATAATTATTGCTATTCCAACGGGAATTAAAATTTTTAGTTGACTCGCAACAATTCATGGATCAAAAATTTCGCTTACACCCCAAATAATTTGATCAACTGGATTTATTTTCCTTTTTACTGTAGGGGGGTTAACAGGAGTAATTTTAGCTAATTCTTCTATTGATATTATTCTTCATGATACATATTATGTAGTAGCCCACTTCCACTATGTATTATCAATAGGAGCTGTATTTACAATTATTGCCAGTTTTATTAATTGATATCCAGTGTTTACTGGAATTGTAATAAACCTAAAATGACTAAAAATTCAATTTTTATCCATATTTATTGGA |
| *Bambusiphaga mirostylis* | GAAGTATACATTTTAATTCTACCCGGATTCGGACTTATTTCACATATTATTATACAAGAAAGAGGAAAAAAAGAAACATTTGGAACTATTGGAATAATCTATGCAATAATCGCCATTGGTGTTCTAGGATTTATTGTGTGAGCTCATCACATATTCACAGTAGGTATAGATATTGACACACGAGCTTATTTTACTTCAGCAACTATAATTATTGCTGTCCCCACAGGAATTAAAATTTTTAGTTGATTAGCTACAATCCATGGATCAAAAATATATTTTTCACCGCAAATAATTTGATCAACAGGATTTATTTTTTTATTCACCATCGGTGGTTTAACAGGAGTAATTTTAGCAAACTCATCCATTGATATTATCTTACACGATACATACTATGTAGTAGCACATTTCCATTACGTTCTCTCTATAGGAGCAGTTTTCACTATTATTGCTAGATTTATTAATTGATACCCCGTATTTACAGGAGTTACAATAAATAATAAATGATTAAAAATTCAATTTTTTACCATATTTATAGGA |
| *Bambusiphaga nigropunctata* | GAAGTATACATTTTAATTTTACCAGGATTTGGATTAATTTCACATATTATTATACAGGAAAGAGGAAAAAAAGAAACATTTGGAACAATTGGAATAATCTACGCAATAATTGCTATTGGAATCTTAGGATTTATTGTTTGAGCACATCACATATTCACCGTAGGTATAGATATCGATACACGAGCTTACTTTACTTCAGCTACTATAATTATTGCTGTCCCCACAGGAATTAAAATTTTTAGTTGATTAGCCACCATTCATGGATCAAAAATTAAATTTTCCCCCCAAATAATTTGATCAACAGGATTTATTTTTCTGTTCACAATTGGTGGATTAACAGGAGTAATTCTAGCAAATTCCTCAATTGATATTGTTTTACATGACACATATTATGTAGTTGCACATTTTCACTATGTACTTTCAATAGGAGCTGTCTTTACTATTATTGCCAGATTTATCAACTGATTTCCCGTATTTACAGGAATATTAATAAACAATAAATGACTAAAAATTCAATTTTTAACCATATTTATAGGA |
| *Bambusiphaga taibaishana* | GAAGTTTACATTTTAATCTTACCAGGATTTGGATTAATTTCCCATATTATTATACAAGAAAGAGGAAAAAAAGAAACATTTGGTACAATCGGAATAATTTATGCAATAATTGCAATTGGAATTTTAGGTTTTATTGTATGAGCACATCATATATTTACTGTTGGTATAGACATTGATACCCGAGCTTACTTTACTTCAGCCACTATAATCATTGCTGTACCTACAGGAATCAAAATTTTTAGATGACTAGCAACTATTCACGGATCAAAAATAACTTTTTCACCACAAATAATTTGATCAACTGGATTTATTTTTCTATTTACAATTGGAGGATTAACAGGAGTAATTTTAGCTAATTCATCCATTGATATTATTTTACATGATACATATTATGTAGTAGCACATTTTCATTATGTATTATCAATAGGAGCTGTATTTACAATTATTGCAAGATTCATTAATTGATACCCAGTATTTTCAGGAATTTCAATAAACAATAAATGACTAAAAATTCAATTTTTTTCAATATTTATAGGA |
| *Belocera sinensis* | ---------------------------------------------------------------------------ACATTTGGTACTATTGGAATAATCTACGCAATAATTGCTATCGGAATTCTAGGATTTATTGTATGAGCCCATCATATATTTACGGTTGGAATAGATATTGATACACGAGCTTACTTTACTTCAGCAACTATAATTATCGCTGTGCCAACAGGAATCAAAATTTTTAGATGACTGGCCACAATCCATGGATCAAAAATTAACTTTTCACCTCAAATAATTTGATCTACAGGATTTATTTTTCTATTTACAATTGGGGGGCTGACTGGAGTAATTCTGGCCAATTCATCAATTGACATTATTTTACACGATACATACTATGTAGTAGCACATTTCCACTATGTATTATCAATAGGAGCTGTATTCACTATCATTTCTAGATTTATTAACTGGTATCCAGTATTTACAGGATTCACAATAAATAACAAATGACTAAAAATTCAATTCTTTTCCATATTCACAGGA |
| *Bostaera nasuta* | ------TATATTTTGATTTTACCAGGTTTTGGTTTAATTTCACATATTATTATGCAGGAAAGAGGAAAAAAAGAAACATTTGGTTCAATTGGAATAATCTATGCAATAATAGCAATTGGAATTCTAGGGTTTATTGTTTGAGCACATCATATATTTACAGTAGGTATAGATATCGATACACGAGCATATTTCACTTCAGCTACTATAATTATCGCTGTACCAACAGGAATTAAAATTTTTAGATGAATGGCAACAATTTATGGATCAAAAATTTTTTATTCTCCTCAAATAATTTGATCTATGGGGTTCATTTTACTTTTTACAATTGGTGGATTAACAGGAGTAATATTAGCAAATTCATCAATTGATATTATTTTACATGATACATATTATGTAGTTGCACATTTTCATTATGTATTATCAATAGGAGCTGTATTTACTATTATTGCAAGTTTTATCCACTGATATCCAATTTTTACAGGAACATCATTAAATAAAAAGTGATTAAAAATTCAATTTTTTTCTATATTCACAGGA |
| *Caenodelphax teapae* | GAAGTTTATATTCTAATTCTCCCCGGATTTGGATTAATTTCACATATTATTATACAAGAAAGAGGAAAACGAGAAACTTTTGGATCTTTAGGTATAATTTATGCTATACTAGCAATTGGAATTTTAGGATTTATTGTGTGAGCCCACCATATATTTACAGTTGGTATAGACATTGATACTCGAGCCTACTTCACCTCAGCAACTATAATTATTGCAGTACCTACAGGTATTAAAATTTTTAGATGAATAGCAACAATCTATGGGTCAAAAATTAATTTCTCACCCCAAATGGTATGGTCCATAGGATTTATTTTGTTATTTACCATTGGTGGTTTAACCGGAGTTATACTAGCCAATTCATCTATTGATATTGTTCTTCACGACACATACTATGTTGTTGCTCACTTCCATTATGTTTTATCTATAGGAGCTGTATTTACAATTGTAGCCAGTTTTATCCATTGATACCCTCTATTTACAGGTGTTACAATAAACAAAAAATGATTAAAAATTCAATTTTCATCTATATTTATTGGA |
| *Carinofrons maculatipennis* | GAAGTATACATTCTTATTCTCCCAGGATTTGGTTTAATTTCACACATTATTATACAGGAAAGAGGAAAAAAAGAAACATTTGGATCTATCGGTATAATCTACGCAATAATAGCAATTGGAATTTTAGGTTTCATTGTTTGAGCCCATCATATGTTTACAGTAGGAATAGATATTGACACACGGGCCTATTTTACTTCAGCTACCATAATTATTGCAGTCCCAACTGGCATCAAAATTTTTAGATGAATAGCAACAATTTTTGGATCAAAAATTTTAAATTCACCTCAAATAATTTGATCCATAGGATTCATTTTACTTTTTACTATTGGGGGATTAACAGGTGTTATACTAGCTAATTCATCAATTGATATTGTTCTACACGATACATATTATGTAGTTGCACACTTTCATTATGTACTTTCTATAGGAGCTGTTTTTACCATTATTGCAAGATTTATCCATTGATACCCCTTATTCACAGGTACTTCATTAAATATAAAATGACTTAAAATTCAATTTTTTTCTATATTTACAGGA |
| *Cemopsis griphus* | GAAGTTTATATCCTAATTTTACCTGGATTTGGTTTAATTTCCCATATTATTATACAAGAAAGAGGAAAAAAAGAAACATTTGGGTCAATCGGAATAATCTATGCCATAATTGCCATTGGAATTCTTGGATTTATTGTTTGAGCCCATCATATATTTACTGTAGGTATAGATATCGACACACGAGCATACTTCACTTCAGCAACAATAATTATTGCAGTTCCTACTGGCATCAAAATCTTTAGATGAATAGCAACCATTTTTGGTTCTAAAATTTCATTTTCCCCACAAATAATTTGGTCAATAGGATTCATTATACTTTTTACAATTGGTGGTCTTACCGGTGTAATATTGGCAAATTCCTCAATTGATATTATTTTACATGACACCTACTATGTAGTAGCACACTTCCACTATGTACTATCAATAGGAGCAGTTTTTACAATTATTGCTAGATTTATCCACTGATATCCATTATTTACAAGAACAATAATAAACAACAAATGACTTAATATCCAA--------------------- |
| *Cemus macaoensis* | GAAGTTTATATTTTAATCCTACCAGGATTTGGATTAATTTCCCATATTATTATACAAGAAAGAGGAAAGCGAGAAACTTTTGGTTCTATTGGTATAATTTATGCTATATTAGCAATTGGTATCTTAGGGTTTATTGTGTGAGCTCACCACATATTTACTGTTGGTATAGATATCGATACACGAGCTTACTTTACTTCAGCCACTATAATTATTGCTGTTCCTACAGGAATTAAAATCTTTAGATGAATCGCTACAATTTATGGATCTAAAATTAACTTCTCTCCTCAAATAATTTGATCAATAGGATTTATCTTATTATTTACTGTAGGGGGACTAACTGGGGTTATATTAGCCAATTCATCAATTGATATTATTCTACATGATACCTATTATGTAGTAGCACACTTTCATTATGTTCTTTCTATAGGTGCTGTATTTACTATTGTTGCTAGATTCATCCATTGATACCCCCTATTTACAGGTGTTTCCATAAACAAAAAATGATTAAAAATTCAATTTTATTCAATATTTATAGGA |
| *Cemus nigromaculosus* | GAAGTATATATATTAATTTTACCTGGATTTGGGTTAATTTCACACATTATTATACAAGAAAGAGGAAAAAAAGAAACATTTGGTTCAATTGGAATAATTTATGCAATAATTGCTATTGGAATTCTAGGATTTATTGTTTGAGCTCACCACATATTCACTGTAGGAATAGATATTGATACACGAGCTTACTTCACCTCAGCCACTATAATTATTGCTGTACCAACTGGTATTAAAATTTTTAGATGAATAGCAACAATATATGGATCTAAAATTTTGTTTTCCCCTCAAATAATTTGATCCATAGGATTTATCTTACTTTTTACAGTTGGGGGACTTACTGGTGTTATACTAGCAAACTCATCAATTGACATTATTTTACACGACACATATTATGTCGTTGCCCATTTCCATTATGTATTATCCATAGGAGCAGTATTTACAATTATTGCAAGATTTATTCATTGATACCCTCTACTCACAGGTATCTCAATAAACAAAAAATGACTCAAAATTCAATTTTTTTCAATGTTCATAGGA |
| *Cemus punctatus* | GAAGTGTATATTCTAATTTTACCAGGTTTTGGTTTAATCTCACATATCATTATGCAAGAAAGAGGTAAAAAAGAAACATTTGGATCAATTGGAATAATTTACGCAATAATTGCTATCGGAATCTTAGGATTCATCGTCTGAGCACATCACATGTTTACTGTAGGAATAGATATTGATACTCGAGCCTATTTTACCTCAGCAACCATAATCATCGCTGTACCAACAGGAATTAAAATTTTTAGATGATTAGCCACAATTTATGGATCTAAAATTTCATTTTCACCACAAATAATCTGATCACTAGGATTTATTCTACTCTTTACTATTGGAGGATTAACTGGTGTAATTTTAGCAAATTCATCAATTGATATTATTTTACATGACACATACTATGTTGTTGCCCATTTCCATTATGTACTCTCTATAGGAGCAGTATTTACAATTATTGCAAGATTCATCCACTGATACCCCCTAATTACAGGAAATTCAATAAATAAAAAATGACTAAAAATACAATTCTTTTCTATATTTATAGGA |
| *Cemus sauteri* | GAAGTATATATCTTAATTTTACCTGGATTTGGGTTAATTTCACACATTATTATACAAGAAAGAGGAAAAAAAGAAACATTTGGTTCAATTGGAATAATTTATGCAATAATTGCTATTGGAATTCTAGGATTTATTGTTTGAGCTCACCACATATTCACTGTAGGAATAGATATTGATACACGAGCTTACTTCACCTCAGCCACTATAATTATTGCTGTACCAACTGGTATTAAAATTTTTAGATGAATAGCAACAATATATGGATCTAAAATTTTGTTTTCCCCTCAAATAATTTGATCCATAGGATTTATCTTACTTTTTACAGTTGGGGGACTTACTGGTGTTATACTAGCAAACTCATCAATTGACATTATTTTACACGACACATATTATGTCGTTGCCCATTTCCATTATGTATTATCCATAGGAGCAGTATTTACAATTATTGCAAGATTTATTCATTGGTACCCCCTACTCACAGGTATCTCAATAAACAAAAAATGACTCAAAATTCAATTTTTTTCAATGTTCATAGGA |
| *Cixius scrupeus* | GAAGTTTATATTTTAATCTTACCAGGATTTGGATTAGTTTCACATATCATTATACAAGAAAGAGGGAAAAAAGAAACATTTGGATCAATCGGAATAATTTATGCAATAATTGCAATTGGATTATTAGGATTTGTGGTTTGGGCACATCATATATTTACAGTAGGTATAGATATTGATACACGTGCTTACTTCACTGCAGCAACTATAGTAATTGCTGTACCAACTGGTATTAAAATCTTTAGATGAATAGCAACTATTCACGGATCAAAAATTAAATTTACACCTCAAACAATATGAGCCATAGGCTTCATTTTACTTTTTACGATAGGTGGATTAACAGGAGTAGTTCTTGCCAATTCATCAATTGATATTATTCTTCATGATACATATTATGTAGTTGCCCACTTTCATTATGTATTATCAATAGGAGCAGTATTTGCTATTATAGGAAGATTTATTCAATGATACCCTTTATTTACAGGACTTCAACTAAATAATAAATGATTAAAAATTCAATTTATAACTATATTTACAGGA |
| *Criomorphus niger* | GAAGTATATATCTTAATCCTCCCCGGATTTGGATTAATCTCACACATTATTATACAAGAAAGAGGTAAAAAAGAAACATTTGGATCAATCGGTATAATTTACGCAATAATTTCAATTGGTATTCTAGGATTCATCGTTTGAGCTCACCACATATTTACAGTTGGAATAGACATCGATACACGAGCCTACTTTACATCAGCAACCATAATTATTGCCGTTCCTACAGGGATTAAAATTTTTAGATGAATTGCCACAATCTACGGTTCAAAAATTAATTTTTCACCTCAAATAATTTGGGCAATAGGATTTATTTTTCTATTTACGATTGGTGGCCTCACCGGAGTTATACTAGCAAACTCATCTGTTGATATTACACTACACGACACCTACTACGTAGTGGCTCATTTCCACTATGTACTATCAATAGGGGCAGTTTTCACTATCATTGCAAGTTTTATTCACTGATACCCCCTATTTACAGGAACTTCAATACATAATAAATGACTAAACATCCAATTTATTACTATATTTACAGGA |
| *Delphacodes penedetecta* | ------------------------------GGATTAATTTCACACATCATTATACAAGAAAGAGGAAAAAAGGAAACCTTTGGATCAATTGGTATAATTTATGCAATAATAGCTATTGGTATTTTAGGGTTTATTGTTTGAGCTCATCATATGTTCACAGTAGGAATAGATATCGACACACGGGCCTACTTTACTTCAGCAACTATAATTATTGCAGTCCCTACAGGAATTAAGATTTTCAGATGAATTGCAACAATTTACGGTTCTAAAATTAAATTTTCTCCCCAAATAATTTGATCTATAGGATTCATTTTATTATTTACAATTGGTGGATTAACAGGAGTAATACTTGCAAATTCTTCAATTGATATTGTACTACATGACACCTATTATGTTGTAGCCCATTTCCACTACGTACTTTCTATGGGAGCAGTTTTTACTATTATCGCAAGATTTATTCATTGATACCCAATTTTTACAGGGCTTTCCTTAAATATAAAATGATTAAAAATTCAATTTTACTCTATATTCATTGGA |
| *Delphacodes puella* | ---GTTTACATTTTAATTTTACCTGGATTTGGTTTAATTTCACATATTATCATGCAAGAAAGAGGAAAGCGAGAAACTTTTGGATCAATCGGAATAATTTACGCTATAATTGCTATTGGAATTTTAGGGTTTATTGTATGAGCACATCATATATTCACAGTTGGTATAGATATTGATACACGAGCCTACTTTACATCAGCAACTATAATCATTGCAGTCCCCACCGGAATTAAAATTTTCAGATGAATCGCCACAATTTACGGGTCCAAAATTAAATTTTCACCCCAAATAATTTGATCAATAGGGTTCATTTTATTATTTACTATTGGTGGATTAACAGGAGTTATACTTGCTAATTCATCAATTGATATTGTACTTCATGATACATACTATGTAGTAGCCCATTTTCATTATGTTTTATCCATAGGAGCTGTATTTACAATTGTAGCTAGATTTATCCACTGATTTCCCCTATTCACAGGGGTTTCCATAAATAAAAAATGATTAAAAATTCAATTTTCATCAATATTTATTGGA |
| *Delphax orientalis* | GAAGTTTATATTCTTATTCTACCAGGGTTTGGCTTAATCTCCCACATTATTATACAAGAAAGAGGAAAAAAAGAAACATTTGGATCAATCGGAATAATTTATGCAATAATTGCAATTGGAATATTAGGGTTTGTAGTTTGAGCTCATCACATATTCACCGTAGGAATAGATATTGACACACGTGCATACTTTACATCAGCAACAATAATTATTGCTGTGCCAACCGGAATTAAAATTTTTAGATGAATAGCCACAATATATGGATCAAAAATTAAGTTTTCAATTCAAACCCTCTGAGCCACAGGATTCGTTTTCTTATTTACATTAGGTGGATTAACAGGAGTAATACTAGCAAATTCATCAATTGATGTAATTATACATGATACCTATTACGTAGTAGCCCATTTCCATTATGTATTATCCATAGGAGCAGTATTTGCAATTTTAGGAAGATTTATTCAATGATACCCATTAATTACAGGATTTTCAATAAACAAAAAATGATTAAAAATTCAATTTTCATCCATGTTTCTAGGA |
| *Dicranotropis hamata* | GAAGTCTATATTCTCATCCTTCCGGGATTTGGACTAATTTCCCACATTATTATACAAGAAAGAGGTAAAAAAGAAACTTTCGGTTCAATTGGAATAATCTACGCTATAATCGCCATTGGAATTCTAGGGTTTATTGTATGAGCACATCATATATTTACTGTAGGTATAGATATTGATACACGAGCCTATTTTACCTCAGCAACCATAATTATTGCTGTACCAACAGGAATTAAAATTTTTAGATGAATCGCCACAATCTACGGATCGAAAATTAACTTTTCTCCTCAAATAATTTGATCTGTGGGATTCATTTTGCTGTTCACAATTGGGGGATTAACCGGAGTAATATTGGCCAACTCATCTATTGATATTATTTTACACGATACATATTATGTAGTAGCACATTTTCACTACGTACTTTCTATAGGGGCTGTTTTCACTATTGTAGCCAGTTTCATCCATTGATACCCCCTTTTTACAGGTGTTTCATTAAACAATAAATGATTAAAAATTCAATTTACTTCAATATTCATCGGA |
| *Distantinus melichari* | GAAGTTTACATTCTAATCCTCCCCGGATTCGGTTTAATTTCACATATTATTATACAAGAAAGAGGAAAACGTGAAACTTTTGGGTCTATTGGAATAATCTACGCTATATTAGCAATTGGAATTTTAGGATTTATTGTATGAGCCCACCATATATTTACAGTAGGTATAGATATTGATACACGAGCCTATTTTACTTCAGCGACTATAATTATTGCAGTACCCACAGGTATCAAAATCTTCAGTTGAATCGCCACCATTTATGGATCAAAAATTAGATTTTCCCCCCAAATAATTTGATCGATAGGATTCATTTTACTTTTTACAATTGGTGGATTAACAGGAGTTATACTAGCTAACTCATCAATTGATGTTGTCCTCCACGACACTTATTATGTGGTAGCACACTTTCACTACGTCCTATCTATAGGAGCAGTTTTTACAATCATTGCCAGATTTATCCACTGATACCCAGTCTTTACCGGAGTTTCAATAAACAAAAAATGACTAAAAATTCAATTCTTTTCTATATTCATAGGA |
| *Ecdelphax cervina* | GAAGTTTATATTCTAATTTTACCAGGATTTGGCTTAATTTCACATATCATTATACAAGAAAGAGGAAAAAAAGAAACATTTGGTTCAATTGGAATAATCTACGCAATAATTGCTATTGGAGTATTAGGATTTATTGTCTGAGCCCACCATATATTTACTGTAGGTATAGATATTGATACCCGAGCATATTTCACTTCAGCAACTATAATTATTGCAGTGCCCACTGGTATTAAAATTTTTAGATGAATAGCAACAATCTATGGTTCTAATATTTCATTTTCACCTCAAATAATTTGATCTATAGGGTTCATTTTACTTTTCACTATTGGGGGGTTAACAGGAGTAATATTAGCAAATTCATCAATTGATATTATCCTTCATGACACTTATTATGTTGTGGCTCACTTCCACTATGTTCTTTCAATAGGAGCAGTTTTTACAATTATTGCAAGATTTATTCATTGATACCCACTATTTAGAGGAACCTCTATAAACAAAAAATGACTTAAAATTCAATTCATATCAATATTTATAGGA |
| *Ecdelphax dentata* | GAAGTTTATATTCTAATTCTACCAGGATTTGGCTTAATTTCACACATCATTATACAAGAAAGAGGAAAAAAAGAAACATTTGGTTCAATTGGAATAATCTACGCAATAATTGCTATTGGAGTATTAGGGTTTATTGTCTGAGCCCACCATATATTTACTGTAGGTATAGATATCGATACCCGAGCGTATTTCACTTCAGCAACTATAATTATTGCAGTGCCCACTGGAATTAAAATTTTTAGATGAATAGCAACAATCTATGGTTCTAACATTTCATTTTCACCTCAAATAATTTGATCTATAGGGTTTATTTTACTTTTCACTATTGGGGGGTTAACAGGAGTAATATTAGCAAATTCATCAATTGATATTATCCTTCATGACACTTATTATGTTGTGGCTCACTTCCACTATGTTCTTTCAATAGGAGCAGTTTTTACAATTATTGCAAGATTTATTCATTGATACCCACTATTTAGAGGAACCTCTATAAACAAAAAATGACTTAAAATTCAATTCATATCAATATTTATAGGA |
| *Elachodelphax metcalfi* | GAAGTTTACATCTTAATCCTTCCAGGATTTGGATTAATTTCACACATTATTATACAAGAAAGAGGTAAGAAAGAAACTTTTGGTTCAATTGGAATAATTTACGCTATAATCGCTATTGGAATTTTAGGATTCATCGTATGAGCTCACCACATATTCACTGTAGGGATAGATATCGACACACGAGCCTATTTTACTTCAGCAACTATAATTATTGCTGTACCTACAGGAATCAAAATTTTTAGATGAATCGCAACTATTTATGGATCAAAAATTAGATTTTCTCCTCAAATAATTTGATCAATAGGATTCATTTTATTATTCACAATTGGAGGACTAACAGGAGTAATACTTGCTAATTCTTCAATTGATATTATTTTACACGATACATATTATGTAGTAGCACATTTTCATTATGTACTTTCTATGGGAGCTGTATTTACTATTGTAGCTAGTTTTATTCATTGATACCCCCTATTTACAGGTGTTTCCTTAAATAATAAATGACTAAAAATCCAATTCTCTTCTATATTTATCGGA |
| *Epeurysa distincta* | GAAGTGTATATTTTAATTTTACCCGGGTTTGGCTTAATCTCCCACATCATTATGCAAGAAAGAGGAAAAAAAGAAACATTTGGGTCAATTGGAATAATTTACGCTATAATTGCTATTGGGGTTTTAGGATTTATTGTATGAGCACACCACATATTTACTGTAGGAATAGACATCGATACACGAGCTTATTTTACATCTGCCACAATAATCATCGCTGTACCTACTGGTATTAAAATTTTTAGATGACTAGGAACAATTCATGGTTCTAAAATTTCATTTTCACCACAAATAATTTGATCTACAGGGTTCATCTTTTTGTTTACAATTGGGGGATTAACTGGAGTTATTCTAGCTAATTCATCAATTGATATTATTCTTCATGATACATATTATGTAGTTGCACATTTCCACTACGTTCTGTCAATAGGCGCTGTTTTTACAATTATCGCAAGTTTTATTAATTGATACCCCGTATTTACAGGTCTAACCATAAACTTAAAATGATTAAAAATTCAATTTATTTCAATATTTATTGGA |
| *Epeurysa infumata* | GAAGTGTATATTTTAATTTTACCAGGCTTTGGCTTAATTTCCCATATCATTATACAAGAAAGAGGAAAAAAGGAAACATTTGGATCAATTGGAATAATTTACGCTATAATTGCCATTGGGGTCTTGGGGTTTATTGTATGAGCACACCACATATTTACTGTTGGAATAGATATTGACACACGAGCTTATTTTACATCTGCTACAATAATTATCGCTGTACCTACAGGTATTAAAATTTTTAGATGACTAGCAACAATTCACGGATCAAAAATTTCATTTTCACCTCAAATGATTTGATCTACAGGATTTATCTTTTTATTCACAATTGGTGGATTAACCGGAGTTATTCTAGCTAACTCATCAATTGATATTATCCTTCATGATACATATTATGTGGTTGCCCATTTCCACTACGTTTTATCAATAGGGGCTGTTTTTACAATTATTGCAAGTTTTATCAACTGATACCCTGTATTTACAGGATTAACCATAAACTTAAAGTGATTAAAAATCCAATTCATTTCCATATTTATTGGA |
| *Epeurysa nawaii* | GAAGTGTATATTTTAATCTTGCCAGGATTTGGATTAATTTCCCATATTATTATACAAGAAAGAGGGAGGAAAGAAACATTTGGGTCAATCGGAATAATTTACGCCATAATTGCTATCGGAGTTTTAGGATTTATTGTATGGGCACACCACATATTTACCGTTGGTATAGATATTGACACACGAGCTTACTTTACATCAGCCACAATAATCATTGCTGTACCCACCGGTATCAAAATTTTTAGATGGCTGGCAACAATTCATGGTTCTAAAATTTCATTTTCCCCACAAATAATTTGATCTACAGGGTTTATCTTTTTATTTACTATTGGAGGATTAACTGGAGTTATTCTAGCTAATTCGTCTATTGATATTATTCTTCATGACACATACTATGTGGTTGCACATTTTCACTACGTTTTATCAATAGGAGCTGTTTTTACAATTATTGCAAGATTCATTAACTGATATCCAGTATTCACAGGGCTAACCATAAATCTAAAATGATTAAAAATTCAATTTATTTCAATATTTATCGGA |
| *Eurybregma nigrolineata* | GAAGTTTACATCCTAATCCTCCCCGGATTTGGATTAATTTCACACATCATTATACAAGAAAGAGGAAAAAAAGAAACATTTGGATCAATCGGTATAATTTATGCAATAATTTCTATCGGTATCTTAGGTTTCATCGTATGAGCCCATCATATATTTACAGTCGGAATAGATATTGATACACGAGCTTACTTCACTTCAGCAACAATAATTATTGCCGTACCTACAGGTATCAAAATTTTTAGATGAATCGCCACGATCTATGGGTCTAAAATTAAATTTTCCCCTCAAATAATTTGATCAATAGGATTCATTTTACTTTTTACAGTTGGTGGATTGACAGGAGTAATACTAGCCAATTCATCGATCGACATCGTATTACATGACACTTACTATGTAGTAGCCCATTTCCACTATGTACTTTCAATGGGTGCTGTATTCACTATTATTGCAAGATTTATTCACTGATACCCCCTATTTACAGGAGTATCAATAAACAATAAATGACTGAAAATTCAATTTATTTCAATATTTACAGGA |
| *Falcotoya lyraeformis* | GAAGTTTACATTCTCATTCTTCCAGGATTTGGACTAATTTCACATATTATTATACAAGAAAGAGGAAAACGAGAAACATTTGGATCAATTGGTATAATTTATGCTATAATAGCAATTGGTATTTTAGGATTTATTGTTTGAGCTCATCATATATTCACGGTAGGAATAGACATCGACACCCGGGCTTACTTCACTTCAGCTACAATAATTATTGCAGTACCAACCGGAATTAAAATTTTTAGTTGAATTGCCACAATTTATGGTTCCAAAATTAATTTTTCTCCTCAAATAATTTGATCCATAGGATTTATTTTACTTTTCACAATTGGGGGATTAACTGGTGTAATACTAGCTAATTCATCTATTGATATTGTTTTACATGATACATATTATGTTGTAGCCCACTTCCATTATGTACTGTCGATAGGAGCTGTATTTACGATTGTAGCAAGATTTATTCATTGATATCCTCTATTCACAGGTGTATCAATAAATAAAAAATGACTTAAAATCCAATTTTCTTCTATATTTATAGGA |
| *Fangdelphax gongshanensis* | GAAGTATATATTTTAATTCTACCGGGATTTGGGCTAATTTCACACATCATCATGCAAGAAAGAGGAAAAAAAGAAACTTTCGGCTCAATTGGTATAATTTATGCAATAATCGCTATTGGAATTTTAGGGTTTATTGTATGGGCACATCACATATTTACTGTTGGTATAGATATTGACACACGAGCCTACTTTACATCAGCAACGATAATTATTGCTGTACCAACAGGAATTAAAATCTTTAGATGAATTGCAACAATTTATGGGTCTAAAATTAACTTTTCACCACAAATAATTTGATCTATTGGATTCATTTTATTATTTACGATTGGTGGATTAACAGGAGTAATATTAGCAAATTCATCTATCGACATTATTTTACACGATACCTATTATGTAGTAGCCCATTTCCATTATGTACTTTCCATAGGAGCTGTCTTCACTATTGTAGCCAGTTTTATTCACTGATACCCTCTATTTACAGGTGTTTCATTAAACAATAAATGACTAAAAATTCAGTTCAATTCTATGTTTATTGGA |
| *Flavoclypeus nigrifacies* | ---------------------------------------------------------------------------------------ATTGGTATAATTTATGCAATATTAGCTATCGGAATTTTAGGATTTATTGTATGAGCCCACCACATATTCACTGTTGGGATAGATATTGATACTCGAGCATATTTTACTTCAGCAACTATAATTATTGCGGTACCCACTGGTATCAAAATCTTTAGATGAATCGCTACCATTTACGGTTCTAAAATAAACTTTTCACCTCAAATAATTTGATCTATAGGATTCATTTTACTTTTTACAATTGGTGGGTTAACAGGTGTAATATTAGCCAATTCATCAATTGATGTTATTCTTCATGACACTTATTATGTAGTAGCACACTTCCATTATGTTCTTTCAATAGGAGCAGTATTCACAATTGTAGCTAGA------------------------------------------------------------------------------------ |
| Gen. nov*.* 1 | GAAGTTTACATTTTAATCCTCCCAGGATTTGGATTAATTTCACATATCATCATACAAGAAAGAGGAAAAAAAGAAACTTTTGGGACTATTGGAATAATTTATGCTATAATTGCAATCGGAATTTTAGGTTTTATCGTATGGGCCCACCACATATTTACTGTAGGTATAGATATTGACACACGAGCATATTTTACCTCAGCCACTATAATTATTGCAGTCCCCACTGGAATTAAAATTTTTAGATGATTGGCCACAATCCACGGATCAAAAATCAACTTTTCTCCTCAAATAATTTGATCTACAGGATTTATTTTCTTATTTACAATTGGTGGATTAACTGGAGTAATTTTAGCTAATTCTTCTATTGATATTATTTTACATGATACATACTATGTAGTTGCCCATTTTCACTACGTATTATCAATAGGAGCTGTATTTACAATTATTTCAAGATTTATTAATTGATATCCAGTATTTACAGGATTAATAATAAACAACAAATGATTAAAAATTCAATTTTTTTCAATGTTTATGGGA |
| *Hadeodelphax afurculus* | GAAGTTTATATTTTAATTTTACCAGGATTTGGGTTAATCTCACATATCATTATACAAGAAAGAGGAAAAAGGGAAACTTTTGGATCAATTGGGATGATTTATGCAATAATCGCTATTGGAATCTTAGGCTTTATCGTTTGAGCCCATCATATATTTACAGTAGGAATAGATATTGACACTCGAGCATATTTTACCTCAGCAACCATAATTATCGCTGTACCTACTGGTATTAAAATTTTTAGATGAATAGCAACAATTTTTGGTTCCAAAATTAATTACACCCCCCAAATAATTTGATCAATAGGATTTATTCTACTTTTCACAATTGGAGGACTAACCGGAGTAATATTAGCAAATTCATCGATTGATATTATTCTTCACGATACATACTATGTTGTAGCTCATTTTCACTATGTTTTATCCATAGGTGCTGTATTTACAATCATTGCAAGTTTTGTACACTGATACCCCCTATTCACAGGACTATCAATAAACATAAAATGACTAAAAATTCAATTTTTTTCAATATTCATAGGA |
| *Harmalia gayasana* | GAAGTTTATATTTTAATCCTCCCGGGATTCGGATTAATTTCTCATATTATTATACAAGAAAGAGGAAAAAAAGAAACATTTGGTTCAATCGGTATAATTTACGCAATATTAGCCATCGGGATTCTAGGATTTATCGTTTGAGCTCACCACATATTCACGGTTGGAATAGATATTGACACCCGAGCTTATTTTACATCGGCCACTATAATTATTGCAGTCCCAACTGGTATCAAAATTTTTAGATGAATAGCAACAATTTACGGTTCTAAAATCAACTTTTCACCTCAAATAATTTGATCAATAGGATTCATTTTACTTTTTACTATTGGTGGATTAACCGGTGTAATATTAGCTAATTCTTCAATCGATATTGTTCTTCACGACACATACTATGTGGTAGCTCACTTTCACTATGTTTTATCTATAGGTGCTGTATTTACTATTATAGCAAGATTTATCCATTGATACCCCCTTTTTACCGGAGTTTCCATAAACAAAAAATGATTAAAAATTCAATTTTCATCTATATTTATTGGA |
| *Harmalia heitensis* | GAAGTTTACATTTTAATCCTCCCAGGATTCGGATTAATTTCTCATATTATTATACAAGAAAGAGGAAAAAAAGAAACGTTTGGTTCAATCGGTATAATTTACGCAATATTAGCCATCGGGATTCTAGGATTTATCGTTTGAGCTCACCACATATTCACGGTTGGAATAGATATTGACACCCGAGCTTATTTTACATCAGCCACTATAATTATTGCAGTCCCTACTGGTATCAAAATTTTTAGATGAATGGCAACAATTTACGGTTCTAAAATCAACTTTTCACCTCAAATAATTTGATCAATAGGATTCATTTTACTTTTTACTATTGGTGGATTAACCGGTGTAATATTGGCTAATTCCTCAATCGATATTGTTCTTCACGACACATACTATGTGGTAGCTCACTTTCACTATGTTTTATCTATAGGTGCTGTATTTACTATTATAGCAAGATTTATCCATTGATACCCCCTTTTTACCGGAGTTTCCATAAACAAAAAATGATTAAAAATTCAATTTTCATCTATGTTTATTGGA |
| *Harmalia ostorius* | GAAGTCTACATTTTAATTCTTCCCGGATTTGGATTAATTTCTCATATTATTATACAAGAAAGAGGAAAAAAAGAGACATTTGGTTCAATTGGTATAATTTACGCTATATTAGCTATCGGAATTCTAGGATTTATTGTTTGAGCTCACCACATATTTACGGTTGGGATAGATATTGATACCCGAGCTTATTTCACATCAGCTACTATAATTATTGCAGTGCCAACTGGGATTAAAATTTTTAGATGAATAGCAACAATCTATGGTTCTAAAATCAACTTTTCACCTCAAATAATTTGATCAATAGGATTCATTTTACTTTTTACTATTGGTGGGTTAACCGGTGTAATACTAGCTAATTCTTCAATTGATATTGTTCTTCACGACACATACTATGTGGTAGCTCACTTTCACTACGTTTTATCAATAGGTGCTGTATTTACTATTATAGCAAGATTTATCCATTGATACCCTCTTTTTACAGGAGTTTCTTTAAATAAAAAATGATTAAAAATTCAATTTTCATCTATATTTATTGGA |
| *Harmalia sirokata* | GAAGTTTACATTTTAATCCTCCCGGGATTCGGATTAATTTCTCATATTATTATACAAGAAAGAGGAAAAAAAGAAACGTTTGGTTCAATCGGTATAATTTACGCAATATTAGCCATCGGGATTCTAGGATTTATCGTTTGAGCTCACCACATATTCACGGTTGGAATAGATATTGACACCCGAGCTTATTTTACATCAGCCACTATAATTATTGCAGTCCCTACTGGTATCAAAATTTTTAGATGAATGGCAACAATTTACGGTTCTAAAATCAACTTTTCACCTCAAATAATTTGATCAATAGGATTCATTTTACTTTTTACTATTGGTGGATTAACCGGTGTAATATTAGCTAATTCCTCAATCGATATTGTTCTTCACGACACATACTATGTGGTAGCTCACTTTCACTATGTTTTATCTATAGGTGCTGTATTTACTATTATAGCAAGATTTATCCATTGATACCCCCTTTTTACCGGAGTTTCCATAAACAAAAAATGATTAAAAATTCAATTTTCATCTATGTTTATTGGA |
| *Harmalia tiphys* | GAAGTCTACATCTTAATTTTACCAGGATTTGGTTTAATTTCTCATATTATTATGCAAGAAAGTGGAAAAAAAGAAACATTTGGTTCTATCGGAATAATTTATGCCATGCTAGCTATTGGAATCCTAGGATTTATTGTATGAGCCCACCATATATTTACAGTAGGAATAGATATTGACACCCGAGCTTATTTTACATCAGCAACTATAATCATCGCTGTGCCAACAGGAATTAAAATCTTTAGATGAATAGCAACAATCTACGGATCTAAAATTAACTTTTCACCTCAAATAATTTGATCTATAGGATTCATTTTACTTTTCACAATTGGGGGATTAACAGGAGTTATACTTGCAAATTCATCAATTGATATTATTTTACATGACACCTACTATGTCGTAGCACACTTTCATTATGTATTATCTATAGGTGCTGTATTTACCATTGTAGCAAGTTTCATTCACTGATACCCACTTTTTACAGGTACAATAATAAACAAAAAATGATTAAAAATTCAATTTTCATCTATATTTATCGGA |
| *Himeunka tateyamaella* | GAAGTTTATATCCTAATTTTACCGGGATTCGGATTAATTTCACACATCATTATACAAGAAAGAGGAAAGCGTGAAACTTTTGGTTCAATTGGTATAATCTACGCCATGCTAGCAATTGGAATCTTAGGGTTTATCGTTTGGGCCCACCATATATTTACTGTGGGTATAGATATCGATACACGAGCCTACTTTACGTCAGCGACAATAATTATTGCGGTCCCCACCGGAATCAAAATCTTCAGTTGAATTGCCACAATTTATGGATCAAAAATTAATTTGTCTCCCCAAATAATTTGATCCATAGGGTTCATTTTACTTTTTACAATCGGTGGGTTAACAGGAGTAATACTAGCTAATTCATCAATTGATATTGTTTTACATGACACTTACTATGTAGTGGCCCACTTTCATTACGTTCTATCAATAGGAGCAGTATTTACAATTGTTGCAAGATTCATCCACTGATACCCTATTTTTACTGGAGTTTCAATAAACAAAAAATGATTAAAAATCCAATTCATTTCTATATTTATAGGA |
| *Ishiharodelphax matsuyamensis* | GAAGTTTATATTCTCATCCTCCCGGGATTTGGATTAATTTCACACATTATTATACAAGAAAGAGGAAAACGAGAAACATTTGGATCAATTGGTATAATTTATGCTATGTTAGCAATTGGGATTTTAGGATTTATTGTTTGAGCTCACCATATATTTACAGTAGGAATAGATATTGATACACGAGCTTACTTTACTTCAGCCACTATAATTATCGCAGTACCAACTGGAATTAAAATCTTTAGTTGAATCGCCACAATTTACGGTTGCAAAATTAACTTTTCCCCCCAAATAATTTGATCCATAGGATTCATTTTACTTTTTACAATTGGGGGATTAACCGGTGTAATACTTGCTAATTCATCTATTGATATTGTTTTACACGATACCTACTATGTCGTAGCTCACTTTCACTATGTTTTATCAATGGGAGCTGTATTTACAATCGTAGCAAGTTTTATCCACTGGTATCCGCTATTTACAGGAGTATCCATAAATAAAAAATGACTAAAAATTCAATTTTCTTCTATATTTTTAGGA |
| *Isodelphax basivitta* | GAAGTGTATATTTTAATTTTACCCGGATTTGGTTTGATTTCACATATTATTATACAAGAAAGAGGAAAAAAAGAAACTTTTGGATCTATCGGAATAATTTATGCTATAATAGCTATCGGTGTTCTAGGGTTTATTGTTTGAGCTCACCATATATTTACTGTAGGTATAGATATCGACACACGAGCTTACTTTACATCAGCGACTATAATTATTGCTGTCCCAACAGGTATCAAAATTTTTAGTTGAATAGCAACAATTTACGGATCAAAAATCAATTTTTCCCCTCAAATAATTTGATCTATAGGATTTATCTTCCTATTTACAATCGGTGGATTAACCGGAGTTATATTAGCTAATTCATCTATTGACATTGTTTTACATGACACGTATTATGTAGTGGCCCATTTTCACTATGTTCTATCTATAGGAGCGGTATTCACTATTATTGCAAGATTTATTCACTGGTACCCTCTGTTTACAGGTGTTTCAATAAATAATAAATGACTAAAAATTCAGTTTATTTCTATATTTCTAGGA |
| *Javesella dubia* | GAAGTGTATATTCTTATTCTTCCAGGGTTTGGATTAATTTCACACATTATTATACAAGAAAGAGGAAAAAAAGAAACATTTGGATCTATCGGTATAATCTACGCAATAATCGCTATCGGAATTTTAGGTTTCATTGTTTGAGCACATCACATATTTACGGTTGGAATAGATATCGATACACGAGCTTACTTTACATCTGCCACCATAATCATTGCAGTACCTACTGGTATCAAAATCTTTAGTTGAATCGCAACAATCTATGGATCAAAAATTAATTTTTCACCTCAGACGATCTGATCAATAGGATTTATTTTACTTTTCACAATTGGTGGATTAACAGGAGTAATATTAGCCAACTCGTCTATTGACATTGTGTTACACGACACATACTACGTAGTAGCACATTTCCACTATGTGTTATCTATGGGAGCTGTATTCACTATTATCGCAAGATTCATTCATTGATACCCCCTATTCACTGGAACATCACTAAATCATAAATGATTAAAAATTCAATTTATTTCTATATTCACCGGA |
| *Javesella obscurella* | GAAGTATATATTCTCATCCTCCCGGGGTTTGGATTAATTTCGCATATTATCATACAAGAAAGAGGGAAAAAAGAAACATTTGGATCAATTGGTATAATTTACGCAATAATAGCTATCGGGATTATGGGTTTCATTGTTTGGGCACATCACATATTTACGGTTGGGATAGATATCGATACACGAGCTTACTTTACATCTGCCACTATAATTATCGCAGTACCTACTGGTATCAAAATTTTCAGCTGAATTGCAACAATCTACGGGTCAAAAATTAATTTTTCCCCACAAACGATCTGATCCATAGGATTTATCTTACTTTTCACAATTGGCGGATTAACTGGAGTGATACTGGCCAACTCCAGCATTGACATTATCCTACACGACACATACTATGTAGTAGCCCATTTCCACTATGTACTATCTATGGGGGCTGTATTTACTATTATCGCCAGATTCATCCATTGATACCCCCTATTTACTGGCACATCATTAAATAATAAATGATTAAAAATTCAATTTATATCAATATTCACTGGA |
| *Javesella pellucida* | GAAGTCTATATTCTTATTCTTCCAGGGTTTGGTTTAATTTCACACATTATTATACAAGAAAGAGGAAAAAAAGAAACATTTGGATCTATTGGTATAATCTACGCAATAATTGCTATCGGAATTTTAGGTTTTATTGTTTGAGCACATCACATATTTACGGTTGGAATAGATATCGATACACGAGCTTACTTTACATCCGCCACCATAATCATTGCAGTACCTACTGGTATCAAAATCTTTAGTTGAATCGCAACAATCTATGGATCAAAAATTAATTTTTCACCACAGACGATCTGATCTATAGGATTTATTTTACTTTTCACAATTGGTGGATTAACGGGAGTAATATTAGCCAACTCATCTATTGACATTGTTTTACACGACACATACTACGTAGTAGCACATTTCCACTATGTTTTATCTATGGGGGCTGTATTCACTATTATCGCAAGATTCATTCATTGATACCCCCTATTCACTGGAACATCACTAAATCATAAATGATTAAAAATTCAATTTATCTCAATATTCACCGGA |
| *Javesella* sp. | GAAGTCTATATTCTTATCCTCCCTGGGTTTGGATTAATCTCCCATATTATTATGCAAGAAAGAGGAAAAAAAGAAACATTTGGATCAATTGGTATAATTTACGCAATAATCGCTATCGGAATTCTAGGATTCATTGTTTGAGCTCATCATATATTTACAGTTGGAATAGATATTGATACACGAGCTTATTTTACATCAGCCACCATAATTATTGCAGTTCCTACAGGTATCAAAATTTTTAGTTGAATCGCAACAATTTATGGATCAAAAATCAATTTCTCCCCACAAACAATCTGATCTATGGGGTTTATTTTACTTTTTACAATTGGAGGATTAACAGGAGTAATACTAGCTAATTCATCTATTGACATTGTTTTACACGATACATATTATGTAGTGGCTCATTTTCATTATGTACTATCTATAGGTGCTGTATTCACTATTATTGCAAGATTTATCCATTGATACCCCTTATTCACTGGTATATCCTTAAATCATAAATGATTAAAAATTCAATTTATCTCAATATTTACTGGA |
| *Kelisia xiphura* | GAAGTTTACATTTTAATTCTCCCAGGATTTGGTTTAATCTCCCATATTATTATACAAGAAAGAGGAAAAAAAGAAACATTTGGTTCTATCGGTATAATTTATGCAATAATTGCAATTGGGATTCTAGGATTCATCGTTTGAGCACATCACATATTCACCGTAGGTATAGATATTGATACACGAGCTTACTTTACTTCAGCAACCATAATCATCGCTGTACCAACAGGAATTAAAGTGTTTAGATGACTAGCAACTATTTACGGATCTAAAATTAATTATTCCCCCCAAATAATTTGATCAATTGGATTTATTTTCTTATTTACTATCGGAGGATTAACAGGTGTAATCCTAGCAAATTCATCAATTGATATTGTTTTACATGACACCTACTATGTAGTAGCCCATTTCCACTATGTACTTTCTATAGGTGCAGTATTTACAATTATTTCAAGATTTATTCATTGATATCCACTATTTACAGGTACATTAATAAATAAAAAATGACTAAAAATTCAATTTTTTTCAATATTTACAGGA |
| *Laodelphax striatellus* | GAAGTGTACATTTTAATTCTCCCAGGATTTGGATTAATTTCACATATTATTATACAAGAAAGAGGTAAGAAAGAAACTTTTGGGTCAATTGGTATGATTTATGCAATAATCGCTATTGGAATTTTAGGGTTTATTGTATGAGCCCATCATATATTTACTGTTGGTATAGATATTGATACACGAGCTTACTTTACATCAGCAACTATAATTATCGCAGTACCTACTGGAATTAAAATCTTTAGTTGAATTGCCACAATTTATGGATCCAAAATTAACTTTTCACCTCAAATAATCTGATCTATTGGATTCATTTTGTTATTTACAATTGGTGGATTAACAGGAGTTATATTAGCAAATTCATCTATCGATATCATTTTACACGATACATATTATGTAGTGGCTCATTTCCACTATGTACTTTCAATGGGAGCTGTTTTTACTATTGTAGCCAGATTCATTCATTGATACCCCCTATTTACAGGTGTTTCATTAAACAATAAGTGACTAAAAATCCAATTCACTTCTATATTTATCGGA |
| *Latistria placitus* | GAAGTTTATATCCTTATTCTCCCCGGATTTGGATTAATCTCCCACATTATCATACAAGAAAGAGGAAAACGTGAAACTTTTGGATCAATCGGTATAATTTACGCTATAATGGCAATTGGAATTTTAGGTTTTATCGTTTGAGCACACCACATGTTTACGGTAGGTATAGATATCGACACACGAGCCTACTTCACTTCAGCAACTATAATCATTGCTGTACCTACAGGAATCAAAATTTTTAGTTGAATCGCCACCATTTATGGATCAAAAATTAAATTTTCCCCACAAATAATTTGGTCCATAGGATTTATTTTTCTTTTTACAATCGGTGGATTAACCGGAGTAATACTAGCTAATTCCTCAATTGATATTGTTTTACACGACACCTACTATGTTGTTGCTCACTTTCACTACGTTTTATCTATAGGAGCCATTTTTACAATTGTTGCTAGATTTATCCACTGATACCCAGTTTTTACAGGAGTTTCAATAAACAAAAAATGGCTAAAAATTCAATTCTTTTCTATATTTTTAGGA |
| *Lauriana senticosa* | GAAGTATATATTTTAATTCTACCCGGATTTGGATTAATTTCACATATTATTATACAAGAAAGTGGAAAAAAAGAAACTTTTGGATCTATCGGAATAATTTATGCAATAATTGCTATCGGAATTTTAGGGTTTATTGTTTGAGCACATCATATATTTACTGTAGGAATAGATATTGACACACGTGCATATTTTACTTCAGCAACTATAATTATTGCTGTACCTACAGGAATTAAAATTTTTAGTTGAATTGCTACAATTCATGGATCAAAAATATCATTTTCACCTCAAATAATTTGGTCAACAGGATTTATTTTTCTTTTTACTATTGGTGGATTAACCGGAGTAATTTTAGCAAATTCCTCAATTGACATTATCTTACATGATACATATTATGTTGTAGCCCATTTTCATTATGTGTTATCTATAGGAGCTGTTTTCACAATTATTGCAAGTTTTATTAACTGATACCCAGTTTTTACAGGATTAACCATAAATATAAAATGACTAAAAATTCAATTTTTTTCAATATTTATAGGA |
| *Liburniella ornata* | GAAGTGTATATTCTAATTTTACCAGGGTTTGGATTAATTTCACATATTATTATACAAGAAAGAGGAAAAAAAGAAACATTTGGTTCAATTGGAATAATCTATGCTATAATTGCCATTGGTATTTTAGGTTTCATTGTATGAGCACACCATATATTTACTGTAGGAATAGATATTGATACACGAGCTTACTTTACTTCAGCCACAATAATCATTGCAGTACCAACAGGAATCAAAATTTTTAGATGATTAGCAACAATTTTTGGATCTAAAATTAACTTTTCTCCCCAAATAATTTGATCAATAGGATTTATTATATTATTCACCATTGGTGGATTAACAGGAGTTATATTAGCTAATTCATCAATTGATATTGTTCTTCATGACACTTACTATGTAGTCGCTCACTTCCACTATGTGTTATCTATAGGAGCTGTATTTACTATTATCGCAAGATTTATTCACTGATATCCTTTATTTACAGGAACTTCAATAAATAATAAATGACTCAAAATTCAATTCTTTTGTATATTTACAGGA |
| *Malaxa semifusca* | GAAGTATACATTTTAATTCTCCCCGGATTTGGATTAGTTTCACATATTATTATACAAGAAAGAGGAAAAAAAGAAACATTTGGATCTATTGGAATAATTTATGCCATAATTGCTATTGGAATTTTAGGGTTTATTGTTTGAGCCCATCACATATTTACTGTAGGAATAGATATTGATACACGAGCTTATTTTACTTCAGCAACTATAATTATTGCTGTCCCCACAGGTATCAAAATTTTTAGATGGTTAGCAACTATTCATGGCTCAAAAATTAAATTTTCCCCCCAAATAATTTGATCAACCGGATTTATTTTCCTTTTCACAATTGGAGGATTAACCGGAGTAATCCTAGCAAATTCATCAATTGACATTGTTTTACATGATACCTACTATGTTGTAGCTCATTTTCACTATGTTTTATCTATAGGAGCTGTATTTACAATTATTTCAAGATTTATCAATTGATACCCAATATTTACTGGATTAATTATAAATTTAAAATGATTAAAAATTCAATTTATTTCAATATTTATTGGA |
| *Malaxella flava* | ---------------------------------------------------------------------------ACATTCGGTACAATCGGAATAATTTATGCAATAATCGCTATCGGAATTCTAGGATTCATTGTATGAGCACACCATATATTTACAGTAGGTATAGATATCGATACACGAGCTTATTTTACTTCGGCCACTATAATCATTGCAGTCCCTACAGGAATTAAAATCTTTAGATGATTAGCAACAATTCATGGATCAAAAATATCCACCTCACCCCAAATAATTTGATCTACAGGATTTATTTTCCTTTTCACAATCGGGGGACTAACCGGAGTAATCTTAGCAAATTCTTCAATTGATGTAATTCTTCATGATACATACTATGTAGTTGCACATTTTCACTATGTATTATCCATAGGAGCTGTATTCACAATCATTTCAAGATTTATCAACTGATACCCAGTATTCACAGGAATTTTAATAAATTCAAAATGACTAAAAATTCAATTTTTATCAATATTTATTGGG |
| *Malaxella macracantha* | GAAGTTTACATTTTAATCCTACCAGGATTCGGATTAATTTCACATATCATTATACAAGAAAGAGGTAAAAAAGAAACATTCGGCACAATTGGAATAATTTATGCAATAATTGCTATTGGAATTCTAGGATTCATTGTATGAGCACATCACATATTTACTGTGGGTATAGACATTGATACACGAGCCTATTTTACTTCAGCAACCATAATTATTGCAGTCCCTACAGGAATTAAAATCTTTAGTTGACTAGCAACAATTCATGGATCAAAAATATCTACTTCCCCCCAAATAATTTGATCAACCGGATTCATCTTCCTTTTCACAATTGGAGGATTAACTGGAGTAATTTTAGCCAACTCCTCAATCGACGTAATTCTTCACGATACATATTATGTAGTTGCACATTTTCATTATGTTCTATCAATAGGAGCTGTATTCACAATCATTTCAAGATTCATTAACTGATACCCACTATTAACAGGAATTATAATAAATTCTAAATGATTAAAAATTCAATTTTTATCAATATTTATAGGA |
| *Malaxella tetracantha* | GAAGTTTATATCTTAATTTTACCAGGATTCGGATTAATTTCACATATTATTATACAAGAAAGAGGTAAAAAAGAAACATTTGGCACAATTGGAATAATTTACGCAATGATTGCTATTGGAATTCTAGGATTTATTGTTTGAGCACATCATATGTTTACTGTAGGTATAGATATTGACACACGAGCTTATTTTACTTCAGCAACTATAATTATTGCAGTCCCTACAGGAATTAAAATTTTTAGATGATTAGCAACAATCCACGGATCAAAAATATCTACCTCCCCCCAAATAATTTGATCAACAGGATTCATTTTCCTTTTTACAATTGGAGGATTAACCGGAGTAATCTTAGCAAACTCCTCAATTGATGTAATTCTTCATGACACATATTATGTAGTTGCACATTTCCATTATGTTCTATCTATAGGAGCTGTATTTACAATTATTTCAAGATTCATCAACTGATACCCAGTATTCACAGGAATTTTAATAAACTCTAAATGATTAAAAATTCAATTTTTATCAATATTTATTGGA |
| *Megadephax cornigera* | GAAGTGTACATCTTAATCCTCCCAGGATTTGGCTTAATTTCACATATTATTATACAGGAAAGAGGAAAAAAGGAAACTTTTGGTTCAATTGGAATAATTTACGCAATAATCGCTATTGGAATTTTAGGGTTTATTGTATGAGCCCATCATATGTTTACTGTTGGTATAGATATTGATACACGAGCCTACTTTACATCAGCGACTATAATTATTGCTGTACCAACAGGAATTAAAATCTTTAGATGAATTGCAACAATCTATGGGTCTAAAATTAACTTATCACCACAAATAATTTGATCTATTGGATTCATTTTATTATTTACAATTGGTGGATTAACGGGAGTTATATTAGCCAACTCCTCTATTGATATCATTTTACACGATACATACTACGTAGTAGCTCATTTCCACTATGTACTCTCAATAGGAGCTGTTTTCACTATTGTAGCCAGTTTTATCCATTGATACCCCCTATTTACAGGTGTTTCACTAAACAATAAATGACTAAAAATTCAATTCACTTCTATATTTATTGGA |
| *Megadephax kangauzi* | ------------------------GGATTTGGATTGATTTCACATATTATTATACAGGAAAGAGGAAAAAAAGAAACTTTTGGTTCAATTGGAATAATTTATGCAATAATCGCTATTGGAATTTTAGGGTTTATTGTATGAGCCCACCATATGTTTACTGTTGGTATAGATATTGATACGCGAGCCTATTTTACATCAGCGACTATAATTATTGCTGTACCAACAGGAATTAAAATTTTTAGATGAATTGCAACAATCTATGGGTCTAAGATTAACTTCTCACCCCAAATAATTTGATCTATCGGATTCATTTTATTATTTACAATTGGTGGATTAACGGGAGTTATATTAGCCAACTCGTCTATTGATATCATTTTACACGATACATACTATGTAGTAGCCCATTTCCACTATGTACTCTCAATAGGAGCTGTTTTCACTATTGTAGCCAGTTTTATCCATTGATACCCCCTATTTACAGGCGTTTCACTAAACAATAAATGACTAAAAATTCAATTCACTTCCATATTTATTGGA |
| *Mestus cruciatus* | GAAGTGTATATTCTTATCCTCCCAGGATTTGGATTAATTTCACATATTATTATACAAGAAAGAGGAAAAAAAGAAACATTTGGCTCAATTGGTATAATTTACGCAATAATTGCTATCGGAATTTTAGGATTCATTGTATGAGCTCATCATATATTTACAGTTGGTATAGATATTGATACGCGAGCTTACTTCACTTCAGCCACTATAATCATCGCCGTGCCCACAGGAATTAAAATTTTTAGTTGAATCGCTACAATCTACGGATCAAAAATTAATTTTTCCCCTCAAATAATTTGAACAATGGGATTTATTTTCTTATTCACAATTGGGGGATTAACTGGAGTTATGCTAGCTAACTCATCAATTGACATTGTTTTACATGATACTTACTATGTAGTAGCTCACTTCCATTATGTGTTATCTATAGGAGCTGTATTCACCATTGTTGCAAGATTTATTCATTGATACCCACTATTTACAGGATCTTCAATAAACAGTAAATGACTTAAAATCCAATTTATTGCAATATTTACTGGA |
| *Metadelphax propinqua* | GAAGTTTACATCCTTATCCTACCTGGATTTGGACTAATTTCACACATTATTATACAAGAAAGAGGAAAACGAGAAACCTTTGGATCAATTGGTATAATTTATGCCATATTAGCTATTGGAATTTTAGGATTTATTGTTTGAGCACACCACATATTCACAGTTGGTATAGATATTGATACTCGAGCTTACTTTACATCAGCAACTATAATTATTGCTGTCCCCACAGGAATTAAAATTTTTAGTTGAATCGCCACAATCTATGGGTCTAAAATTAGATTTTCACCCCAAATAATTTGATCTATAGGATTTATTTTACTTTTTACTATTGGAGGATTAACAGGAGTTATACTTGCTAACTCATCTATTGATATTGTACTTCATGACACATATTATGTAGTAGCACATTTTCATTATGTCCTTTCTATAGGAGCAGTATTTACAATTGTAGCTAGATTTATCCACTGATACCCAATTTTTACTGGATTATCCATAAATAAAAAATGACTCAAAATTCAATTTAGATCAATATTTATCGGA |
| *Metadelphax propinqua* | GAAGTTTATATCCTTATCCTCCCTGGATTTGGACTAATTTCACATATTATTATACAAGAAAGAGGAAAACGAGAAACCTTTGGGTCAATCGGTATAATCTATGCTATATTAGCTATTGGGATTTTAGGATTTATTGTTTGAGCACACCACATATTTACAGTTGGTATAGATATTGATACTCGAGCTTACTTTACATCAGCAACTATAATTATTGCTGTACCTACAGGAATTAAAATTTTCAGTTGGATTGCAACAATTTACGGGTCAAAAATTAAATTTTCACCCCAAATAATTTGATCTATAGGATTTATTTTACTTTTTACTATTGGAGGGTTAACAGGAGTTATACTTGCTAATTCATCTATTGATATTGTTCTTCATGATACATATTATGTAGTAGCACATTTTCATTATGTTCTTTCTATAGGAGCAGTATTTACAATTGTCGCTAGATTTATTCACTGATACCCAATTTTTACTGGATTATCCATAAATAAACAATGACTCAAAATTCAATTTAGATCAATATTTATTGGA |
| *Miranus serrulatus* | GAAGTTTACATTTTAATTCTTCCTGGATTTGGATTAATTTCACATATTATTATGCAAGAAAGAGGAAAAAAAGAAACTTTTGGATCAATTGGAATAATTTACGCAATAATCGCTATTGGTATTCTAGGATTTATTGTTTGAGCTCATCATATATTTACAGTAGGTATAGATATTGATACACGAGCATATTTTACATCTGCTACAATAATTATTGCTGTACCTACAGGAATTAAAATCTTTAGATGAATAGCAACAATCTTTGGATCCAAAATTAATGGTTCCCCCCAAATAATTTGATCTATAGGATTCATTTTACTATTTACAATTGGAGGATTAACAGGTGTTATATTAGCTAATTCTTCAATTGACATCATTCTTCATGACACTTATTATGTAGTTGCGCATTTCCATTATGTTCTCTCAATAGGAGCTGTTTTTACTATTATTGCAAGTTTTATTCATTGATACCCTCTATTCACAGGATTATCACTTAATAATAAATGACTAAAAATTCAATTTTTTTGCATATTCACAGGA |
| *Miranus varians* | GAAGTTTACATTTTAATTCTTCCTGGATTTGGATTAATTTCACATATTATTATACAAGAAAGAGGAAAAAAAGAAACTTTTGGATCAATTGGAATAATTTACGCAATAATCGCTATTGGTATTTTAGGATTTATTGTTTGAGCTCATCATATATTTACAGTAGGTATAGATATTGATACACGAGCATACTTTACATCTGCTACAATAATTATTGCTGTACCTACAGGAATTAAAATTTTTAGTTGAATAGCAACAATCTTTGGATCTAAAATTAATGGTTCCCCCCAAATAATTTGATCCATAGGATTCATTTTACTATTTACAATTGGAGGATTAACAGGTGTTATATTAGCTAATTCTTCAATTGACATTATCCTTCACGACACCTATTATGTAGTTGCACATTTCCATTACGTTCTCTCAATAGGAGCTGTTTTTACTATTATTGCAAGTTTTATCCATTGATACCCGCTATTTACAGGATTATCACTTAATAATAAATGACTTAAAATTCAATTCTTTTGTATATTCACAGGA |
| *Monospinodelphax dantur* | GAAGTTTACATCTTAATTTTACCCGGATTTGGATTAATTTCACATATTATTATACAAGAAAGTGGAAAAAAGGAAACTTTTGGATCTATCGGAATAATCTACGCAATAATTGCTATCGGAATTTTAGGATTTATTGTTTGAGCACACCATATATTTACAGTAGGTATAGATATCGATACACGAGCTTACTTCACCTCAGCAACCATAATTATTGCTGTGCCTACTGGTATCAAAATTTTTAGTTGAATAGCAACAATTTATGGGTCTAAAATTTCATTTTCACCTCAAATAATTTGATCGATAGGATTTATTTTACTTTTTACAGTAGGAGGATTAACAGGTGTAATACTAGCAAATTCATCAATTGATATCATCCTTCATGACACCTACTATGTTGTTGCACACTTCCATTATGTTCTATCAATAGGAGCAGTATTTACAATTATTGCAAGATTTATCCATTGATACCCCCTATTTACAGGGACCTCAATAAATATTAAATGATTAAAAACACAATTTTTCTTAATATTTTTAGGA |
| *Muirodelphax arvensis* | ---------------ATCCTTCCAGGATTTGGATTAATTTCACACATTATTATACAAGAAAGAGGTAAAAAAGAAACTTTTGGTTCAATTGGAATAATTTACGCTATAATCGCTATTGGAATCTTAGGATTCATCGTATGAGCTCACCACATATTTACCGTAGGAATAGATATCGATACACGAGCCTATTTTACCTCAGCAACTATAATTATTGCTGTCCCTACAGGAATCAAAATTTTTAGATGAATCGCAACTATTTATGGATCAAAAATTAGATTTTCCCCTCAAATAATTTGATCAATAGGATTCATTTTATTATTCACAATTGGAGGATTAACAGGAGTAATACTCGCTAATTCTTCAATTGATATTATTTTACATGATACGTACTATGTAGTAGCACATTTTCACTATGTACTTTCTATGGGAGCTGTATTTACTATTGTAGCTAGTTTTATTCATTGATACCCCCTATTTACAGGTGTTTCCCTAAATAATAAATGGCTAAAA--------------------------- |
| *Muirodelphax atratus* | GAAGTGTACATCTTAATCCTCCCAGGATTTGGCTTAATTTCACATATTATTATACAGGAAAGAGGAAAAAAAGAAACTTTTGGTTCAATTGGAATAATCTATGCAATAATCGCTATTGGAATTTTAGGGTTTATTGTATGAGCTCATCATATATTTACTGTTGGTATGGATATTGATACACGAGCCTACTTTACATCAGCGACTATAATTATTGCTGTGCCAACAGGAATTAAAATCTTTAGATGAATTGCAACAATCTATGGATCTAAAATTAACTTCTCACCCCAAATAATTTGATCTATTGGATTCATTTTATTATTTACAATTGGTGGATTGACGGGAGTTATATTAGCCAACTCCTCTATTGATATCATTTTACACGATACATACTACGTAGTAGCCCATTTCCACTATGTACTATCAATAGGAGCTGTTTTCACTATTGTAGCCAGTTTTATCCATTGATATCCCCTATTTACAGGTGTTTCACTAAACAATAAATGACTAAAAATTCAATTCACTTCCATATTTATTGGA |
| *Neobelocera* sp. | ---------------------------------------------------------------------------ACATTTGGAACAATTGGAATAATTTACGCCATAATTGCTATTGGAATTTTAGGATTCATCGTATGAGCACACCATATATTCACAGTAGGAATAGATATCGACACACGAGCTTATTTTACATCAGCCACTATAATTATCGCAGTACCCACAGGTATCAAAATCTTTAGATGACTAGCTACTATACACGGGTCTAAAATAAAACTTTCACCCCAAATAATTTGAGCTACAGGGTTTATTTTTCTATTCACAATTGGAGGATTAACAGGAGTAATTTTAGCAAATTCATCAATTGATATCATTTTACATGACACTTATTATGTCGTTGCTCATTTCCATTATGTTTTATCCATAGGAGCTGTATTCACAATCATTGCAAGATTTATCAATTGATACCCCCTATTTACAGGAATTATAATAAACTCAAAATGATTAAAAATCCAATTTTTATCAATATTTATTGGA |
| *Neocarinodelphax hainanensis* | GAAGTTTACATTTTAATTTTACCAGGGTTTGGTTTAGTGTCACATATTATTATACAAGAAAGAGGAAAAAAAGAAACATTCGGTACAATTGGAATAATCTATGCAATAATTGCTATTGGTATTCTAGGGTTTATTGTATGAGCACATCATATATTTACTGTAGGAATAGATATTGATACACGAGCTTATTTTACATCCGCTACTATGATTATTGCAGTCCCTACAGGAATTAAAATTTTTAGTTGATTAGCTACAATTCACGGATCAAAAATTTTATTTTCGCCTCAAATAATTTGATCAACAGGATTTATTTTTTTATTTACTATTGGAGGATTAACAGGAGTTATTTTAGCAAATTCATCTATTGATATTATTTTACATGATACCTACTATGTTGTAGCTCACTTTCACTATGTGTTATCTATAGGTGCTGTATTTACAATTATTGCAAGATTCATTAATTGATATCCTGTGTTTACAGGAATTCTAATAAATAATAAATGATTAAAAATTCAATTTTTAGCAATATTTATGGGA |
| *Neomegamelanus elongatus* | ---------------------------GTTGGATTAATTTCTCATATTATTATACAAGAAAGAGGAAAAAAAGAAACCTTTGGATCAATTGGGATAATTTTTGCTATATTAGCTATTGGGATTTTAGGGTTTATTGTCTGAGCTCATCACATATTCACAGTAGGAATAGATGTTGATACACGAGCTTATTTCACCTCAGCAACAATAATTATTGCGGTACCCACTGGAATTAAAATTTTTAGTTGAATTGCTACAATTTATGGTTCAAAAATTAAATTTTCTCCCCAAATAATTTGATCTATAGGATTTATTTTATTATTTACAATTGGAGGACTAACAGGAGTAATATTAGCCAACTCATCAATTGATATTGTACTTCATGACACATATTATGTGGTAGCCCATTTCCATTATGTACTTTCCATAGGAGCAGTATTTACTATCATTGCAAGATTTATCCATTGATATCCTCTATTTACAGGAATTCATTTAAATTATAAATGACTA------------------------------ |
| *Neometopina orientalis* | GAAGTTTATATTTTAATTTTACCAGGATTTGGATTAATCTCACATATTATTATACAAGAAAGTGGTAAAAAAGAAACTTTTGGATCAATTGGTATAATTTACGCAATAATTGCCATTGGAATCCTAGGGTTTATTGTATGAGCCCATCATATATTTACAGTAGGAATAGATATTGATACACGAGCTTATTTTACATCCGCAACTATAATTATCGCTGTACCTACAGGTATTAAAATCTTCAGATGAATAGCAACAATTTTTGGTTCAAAAATTAATTTTTCCCCTCAAATAATCTGATCAATAGGATTTATTTTATTATTCACTATTGGAGGTTTAACAGGAGTTATACTAGCAAATTCATCAATTGATGTAGTGCTACATGACACTTACTATGTAGTTGCACACTTCCATTATGTATTATCTATGGGGGCTGTATTCACTATTATTGCAAGATTTATTCACTGATTTCCACTTTTTACAGGTGTTTCAATAAATAACAAATGATTAAAAATCCAATTTTTTTCAATATTCACTGGA |
| *Nilaparvata bakeri* | GAAGTTTATATCCTTATTCTTCCTGGATTTGGATTAATTTCTCATATTATTATACAGGAAAGAGGAAAAAAAGAAACTTTTGGATCTATTGGAATAATCTATGCAATGATTGCAATCGGAATTTTAGGATTTATTGTTTGAGCCCACCATATGTTTACTGTAGGTATAGATATTGATACCCGAGCCTATTTTACATCTGCTACTATAATTATTGCAGTCCCCACTGGAATTAAAATTTTTAGCTGAATTGCAACCATTTATGGTTCCAAAATAAATTTTTCCCCACAAATAATTTGATCATTAGGATTCATTTTACTTTTTACTATTGGTGGGTTAACAGGTGTTATACTAGCAAATTCATCAATTGATATTATCTTACATGATACTTACTATGTAGTTGCTCATTTTCACTATGTTCTTTCCATAGGAGCTGTATTTACTATCATCGCTAGATTTATTCACTGATTTCCATTATTTACAGGTAGAACTATAAATAATAAATGACTAAAAATTCAATTTTACTCAATATTTTTAGGA |
| *Nilaparvata lugens* | GAAGTTTACATCCTTATTCTTCCAGGATTTGGATTAATTTCTCATATTATTATACAAGAAAGAGGAAAAAGGGAAACTTTCGGATCTATTGGAATAATTTATGCAATAATTGCAATTGGAATTTTAGGTTTTATTGTTTGAGCTCACCATATATTTACTGTAGGTATAGATATTGATACCCGAGCCTATTTTACGTCAGCTACTATAATTATTGCGGTCCCCACCGGAATCAAAATTTTTAGATGAATCGCAACAATTTACGGTTCCAAAATGAACTTTTCCCCCCAAATAATTTGATCATTAGGATTCATTTTACTTTTTACTATTGGAGGATTAACAGGTGTAATATTATCAAATTCCTCAATTGATATTATTCTACATGATACCTATTATGTAGTGGCTCATTTTCACTATGTCCTTTCCATAGGAGCAGTATTCACCATTATCGCTAGATTTATCCATTGATACCCCTTATTTACAGGTAGAAACATAAATAATAAATGACTAAAAATTCAATTTTATTCCATATTTCTAGGA |
| *Nilaparvata muiri* | GAAGTTTATATTCTTATTCTCCCAGGGTTTGGATTAATTTCTCACATTATTATGCAAGAAAGAGGAAAAAAAGAAACTTTTGGGTCTATCGGAATAATCTATGCAATAATTGCAATCGGAATTCTAGGGTTTATTGTTTGAGCCCACCACATATTTACTGTAGGTATAGATATTGACACCCGAGCCTATTTTACATCAGCTACTATGATTATTGCAGTCCCCACTGGAATTAAAATCTTTAGCTGAATTGCAACAATTTATGGTTCCAAAATAAATTTTTCCCCACAAATAATTTGATCTTTAGGATTCATTTTACTTTTCACTATTGGAGGATTGACAGGTGTGATACTAGCAAATTCCTCAATTGATATTATTTTACATGACACTTATTATGTAGTAGCTCATTTTCATTATGTTCTTTCTATAGGAGCAGTATTTACTATTATCGCCAGATTTATCCACTGATTTCCATTATTTACAGGTAGAACCATAAATAATAAATGACTAAAAATTCAATTCTATTCAATATTTTTAGGA |
| *Nothodelphax neocclusa* | GAAGTGTACATCTTAATTTTACCTGGGTTTGGATTAATTTCCCATATTATCATACAAGAAAGTGGGAAAAAAGAAACCTTTGGTTCTGTAGGAATAATCTATGCTATAATCTCTATTGGTGTTTTAGGGTTCATCGTGTGAGCCCACCATATGTTCACAGTAGGAATAGATATTGATACGCGAGCTTACTTTACTTCAGCTACTATAATTATTGCTGTCCCCACAGGAATTAAAATTTTTAGCTGAATCGCTACAATTTACGGATCAAAAATTAATTTTTCCCCCCAAATAGTGTGATCTATGGGATTCATTTTACTATTTACAATTGGTGGATTAACAGGAGTTATGTTAGCCAACTCATCCATTGATATCGTTTTACATGATACCTACTATGTAGTAGCCCATTTCCATTACGTACTTTCCATGGGAGCGGTATTCACTATTATTGCAAGATTTATCCACTGATACCCGTTATTTACGGGAATCAGATTAAAT------------------------------------------ |
| *Numata muiri* | GAAGTCTATATCTTAATCCTCCCAGGATTTGGACTAATTTCCCACATTATCATACAGGAAAGAGGTAAAAAAGAAACATTTGGATCAATCGGAATAATTTATGCAATAATTGCTATTGGAATCTTAGGTTTTATTGTTTGGGCCCACCACATATTTACAGTAGGAATAGATATTGATACACGTGCATATTTTACTTCAGCAACTATAATTATTGCAGTCCCCACTGGCATCAAAATTTTTAGATGAATAGCAACAATTTATGGCTCTAAAATTTCATTTTCACCCCAAATAATTTGATGTATAGGATTCATTTTACTTTTTACGATTGGAGGATTAACAGGAGTAATACTAGCAAATTCATCAATTGATATCATCCTCCATGATACATATTATGTTGTTGCTCACTTCCACTATGTTCTTTCTATGGGAGCAGTTTACACTATTATTGCAAGATTTATCCATTGATTTCCCCTGTTCACAGGCCTATGCATAAATAATAAATGATTAAAAATTCAATTTTTTTCAATATTTTTAGGA |
| *Oecleopsis* sp. | GAAGTATATATTTTAATTTTACCAGGATTTGGTTTAATTTCTCATATTATTATACAAGAAAGAGGAAAAAAAGAAACTTTCGGCTCAATCGGAATAATTTATGCAATAATTGCTATTGGAGTATTAGGATTTGTAGTTTGAGCTCATCATATATTCACTGTTGGAATAGATATTGACACACGAGCTTATTTTACCTCAGCAACCATAATTATTGCTGTTCCAACAGGAATTAAAATTTTTAGATGACTAGCAACAATTTACGGAACTAAAATAAAATTTAATCCACAAACCTTATGAGCAAGAGGATTTGTATTCTTATTTACAATAGGAGGTATTACAGGAGTAATTTTATCCAATTCATCTATTGATATTATTCTTCACGATACATATTATGTAGTTGCACACTTTCATTATGTATTATCCATAGGAGCAGTATTTGCAATTATAGGAAGATTTATTCAATGATACCCTCTAATAACAGGATTATATTTAAATAGAAAATGACTAAAAATTCAATTTATAACCATATTTACAGGA |
| *Oliarus* sp. | GAAGTATATATTTTAATTTTACCAGGATTTGGTTTAATTTCTCATATTATTATACAAGAAAGAGGAAAAAAAGAAACTTTCGGTTCAATTGGAATAATTTACGCAATAATTGCTATTGGAGTATTAGGATTTGTAGTTTGAGCTCATCATATATTCACTGTTGGGATAGATATTGATACACGAGCTTATTTCACCTCAGCAACCATAATTATTGCTGTTCCAACAGGCATTAAAATTTTTAGATGACTAGCAACAATTTACGGAACTAAAATAAAATTTAATCCACAAACCTTATGAGCAAGAGGATTTGTATTCCTATTTACAATAGGAGGTATCACAGGAGTAATTTTATCCAATTCATCTATTGATATTATCCTTCACGATACATATTATGTAGTTGCACACTTTCATTACGTATTATCTATAGGAGCAGTATTTGCAATTATAGGAAGATTTATTCAATGATATCCTCTAATAACAGGATTATATTTAAATAGAAAATGACTAAAAATTCAATTTATAACCATATTTACAGGA |
| *Opiconsiva albicollis* | GAAGTCTACATTTTAATCCTTCCCGGATTTGGATTAATTTCTCATATTATTATACAAGAAAGAGGAAAAAAAGAGACATTTGGTTCAATTGGTATAATTTACGCTATATTAGCTATCGGAATTCTAGGATTTATTGTTTGAGCTCACCACATATTTACGGTTGGGATAGATATTGATACCCGAGCTTATTTCACATCAGCTACTATAATTATTGCAGTGCCAACTGGGATTAAAATTTTTAGATGAATAGCAACAATCTATGGTTCTAAAATCAACTTTTCACCTCAAATAATTTGATCAATAGGATTCATTTTACTTTTTACTATTGGTGGGTTAACCGGTGTAATACTAGCTAATTCTTCAATTGATATTGTTCTTCACGACACATACTATGTGGTAGCTCACTTTCACTACGTTTTATCAATAGGTGCTGTATTTACTATTATAGCAAGATTTATCCATTGATACCCTCTTTTTACAGGAGTTTCTTTAAATAAAAAATGATTAAAAATTCAATTTTCATCTATATTTATTGGA |
| *Opiconsiva albimarginata* | GAAGTTTACATCTTAATTCTACCTGGATTTGGTTTAATTTCTCACATCATTATACAAGAAAGAGGCAAAAAAGAAACATTTGGATCTATCGGTATAATTTATGCTATGTTAGCCATCGGAATTCTAGGATTTATTGTATGAGCCCACCATATATTTACAGTAGGAATAGATGTTGATACTCGAGCCTACTTTACTTCAGCAACCATAATTATCGCTGTGCCAACGGGTATCAAAATTTTTAGATGAATAGCAACAATTTACGGGTCTAAAATTAATTTTTCTCCTCAAATAATTTGATCTATGGGATTTGTTTTACTTTTCACAATTGGGGGACTCACAGGTGTTATACTAGCAAATTCCTCAATTGATATTGTTTTACACGATACCTACTATGTAGTAGCACATTTTCATTATGTCTTATCTATAGGTGCTGTATTCACCATTGTAGCCAGATTCATTCACTGATACCCTCTTTTTACAGGTATATCAATAAATAAAAAATGATTAAAAATTCAATTTTCAACCATGTTTATCGGA |
| *Opiconsiva nigra* | GAAGTTTATATTCTTATTTTACCAGGATTCGGTCTTATTTCACACATTATTATACAAGAAAGAGGAAAAAAAGAAACATTTGGTTCAATTGGAATAATCTACGCTATATTAGCTATTGGAATTTTAGGATTTATTGTATGAGCACATCACATATTCACAGTAGGAATAGATATTGATACACGAGCTTATTTTACATCTGCTACAATAATTATTGCTGTCCCCACAGGAATTAAAATTTTCAGATGAATAGCAACAATTTATGGTTCCAAAATTAATTATTCTCCTCAAATAATTTGATCAATAGGATTTATTTTACTTTTCACAATTGGGGGATTAACCGGAGTAATATTAGCCAACTCATCAATTGATATTGTTCTTCATGATACATATTATGTTGTAGCTCATTTTCATTATGTACTGTCAATAGGAGCTGTATTCACTATTGTAGCTAGATTTATCCATTGATTTCCTTTATTTACAGGTGTAGCATTAAACAATAAATGATTAAAAATTCAATTTTCCTCCATGTTTATTGGA |
| *Opiconsiva* sp. | GAAGTTTATATTCTTATTTTACCTGGATTTGGTCTTATTTCACACATTATTATACAAGAAAGAGGAAAAAAAGAAACATTTGGTTCAATTGGAATAATCTACGCTATATTAGCTATTGGGATTTTAGGATTTATTGTATGAGCACATCACATATTTACAGTAGGAATAGATATTGATACACGAGCTTACTTTACATCTGCTACAATAATTATTGCTGTCCCTACAGGAATTAAAATTTTCAGATGAATAGCAACAATTTATGGTTCCAAAATTAATTTTTCTCCTCAAATAATTTGATCAATAGGATTTATTTTACTTTTCACAATTGGGGGATTAACCGGAGTAATATTAGCCAACTCATCAATTGATATTGTTCTTCATGATACATATTATGTTGTAGCTCATTTTCATTATGTCCTGTCTATAGGAGCTGTATTCACTATTGTAGCTAGCTTTATCCATTGATTTCCTTTATTTACAGGTGTAGCATTAAACAATAAATGATTAAAAATTCAATTCTCCTCCATGTTT------ |
| *Palego simulator* | GAAGTCTACATCTTGATCCTCCCAGGATTTGGCTTAATCTCCCATATTATTATACAAGAAAGAGGTAAAAAGGAAACATTCGGATCAATTGGTATAATTTATGCAATAATTGCTATTGGTATTTTAGGATTTATCGTATGAGCTCACCACATATTTACAGTAGGAATAGACATCGATACACGTGCCTACTTCACTTCAGCTACTATAATTATTGCTGTACCAACTGGTATTAAAATTTTTAGATGAATAGCAACAATTTATGGTTCTAAAATTTTATTTTCACCTCAAATAATTTGATCTATAGGGTTCATTTTACTTTTCACAGTTGGGGGACTTACAGGTGTTATATTAGCTAACTCATCAATTGATATCATTTTACATGACACTTACTATGTTGTTGCTCATTTCCATTATGTTCTATCCATAGGAGCAGTTTTTACTATCATCGCTAGATTTATTCACTGATACCCATTATTTACAGGACTCTCCATAAATAATAAATGACTAAAAATTCAGTTTTCTTCTATATTTTTAGGA |
| *Paradelphacodes paludosa* | GAAGTTTACATCCTTATCCTCCCTGGATTTGGATTAATCTCACACATCATTATACAAGAAAGAGGGAAAAAAGAAACATTTGGATCAATCGGTATAATCTACGCCATAATCGCTATCGGTATCCTAGGATTTATTGTATGAGCACACCACATATTCACAGTAGGTATAGACATCGATACACGAGCCTACTTCACCTCAGCAACCATAATCATCGCTGTACCTACAGGAATTAAAATTTTCAGTTGAATCGCTACAATTTATGGTTCTAAAATTTCGTTTTCCCCCCAAATAATTTGATCCATAGGATTCATTTTACTTTTCACTGTTGGGGGCCTAACAGGAGTTATACTAGCCAATTCATCTATCGACATTGTTTTACATGACACTTACTACGTAGTAGCCCACTTCCATTATGTACTCTCAATAGGTGCCGTATTCACTATTATTGCAAGATTCATCCACTGATACCCCCTTCTTACAGGAACGTCAATAAACAAAAAATGACTAAAAATTCAATTTGTTTCAATATTCGCAGGA |
| *Paradelphax nigrostriata* | GAAGTCTATATTCTTATCCTTCCAGGATTTGGACTAATTTCCCACATTATTATACAAGAAAGAGGTAAAAAAGAAACTTTTGGTTCAATTGGAATAATCTATGCAATAATCGCCATTGGAATTCTAGGATTTATTGTATGAGCACATCATATATTTACGGTGGGTATAGATATTGATACCCGAGCCTATTTTACCTCAGCAACCATAATTATTGCTGTACCAACAGGAATCAAAATTTTTAGATGAATCGCTACAATTTACGGATCTAAAATTAACTTTTCACCTCAAATAATTTGATCAGTGGGATTCATTTTACTATTCACAATTGGAGGGTTAACCGGAGTGATATTAGCCAACTCATCTATTGATATTATTTTACACGACACATATTATGTAGTAGCACATTTTCACTACGTCCTTTCCATAGGAGCTGTTTTCACCATTGTAGCCAGTTTTATCCATTGATACCCCCTTTTTTCAGGTGTTTCATTAAACAATAAATGATTAAAAATTCAATTTACTTCTATATTCATCGGA |
| *Paranectopia lasaensis* | GAAGTATACATCTTAATCCTACCAGGATTTGGATTAGTTTCACATATCATTATACAAGAAAGAGGAAAAAAGGAAACCTTCGGTTCAATTGGCATAATCTATGCCATAATTGCAATTGGTATCTTAGGGTTTATCGTATGGGCCCACCACATATTTACAGTGGGAATAGATATTGATACACGAGCCTATTTTACTTCAGCTACTATAATCATTGCTGTCCCGACAGGAATTAAAATCTTTAGATGAATCGCTACAATTTACGGGTCAAAAATTAACCTTTCACCCCAAATAATCTGAACAATAGGATTCATTTTCTTATTCACTATTGGAGGTTTAACCGGAGTGATGTTAGCTAATTCATCTATTGATATTGTTCTACACGACACTTACTATGTAGTAGCCCACTTTCACTATGTTCTTTCAATAGGAGCTGTATTCACTATTATTGCCAGATTTATTCACTGATACCCTTTATTTACAGGCACATCAATAAACAATAAATGATTAAAAATCCAATTTATTGCAATGTTTACAGGA |
| *Pentastiridius* sp. | GAAGTTTATATTTTAATCTTACCAGGATTTGGGCTAATTTCACACATTATTATAAAAGAAAGAGGTAAAAAAGAAACATTTGGATCAATTGGAATAATTTATGCAATAATCGCTATTGGTGTACTTGGATTTGTAGTTTGAGCACATCATATATTCACTGTAGGTATAGATATTGATACACGAGCATATTTCACATCAGCAACAATAATTATTGCAGTACCAACAGGTATTAAAATTTTTAGATGAATAGCAACAATCTATGGAACAAAAATTAAATTCACACCCCAAATAATATGAGCTATAGGATTTATTTTCCTTTTTACTATAGGTGGATTAACAGGTGTTATTCTTGCAAATTCATCTATTGATATTATTCTTCATGATACATATTATGTAGTAGCCCACTTTCATTATGTTTTATCAATAGGAGCAGTTTTTGCAATTATAGGAAGATTTATCGAATGATATCCATTGATAACGGGTTTATCAATAAATCAAAAATGATTAAAAATTCAATTCTTTACTATATTTACAGGA |
| *Pissonotus albovenosus* | GAAGTTTACATTTTAATTCTACCAGGGTTTGGATTAATTTCCCATATTATTATACAAGAAAGnGGTAAGAAAGAAACATTTGGTTCAATCGGTATAATTTATGCAATAATCGCAATTGGAATTTTAGGATTTATCGTGTGAGCACATCACATATTnACTGTAGGAATAGATATCGACACACGAGCATATTTTACTTCAGCGACTATAATTATTGCAGTACCAACAGGAATCAAAATTTTTAGATGAWTRGCCACTATTTACGGAGCTAAAATTATTTATTCACCACAAATAATTTGATCTATAGGATTCATTTTACTTTTCACAATAGGTGGATTAACGGGAGTAATATTAGCCAATTCATCTATTGATGTTATTTTACATGATACTTACTATGTAGTTGCACATTTTCATTATGTTCTTTCAATAGGAGCAGTATTTACTATTATTGCAAGTTTTATCCACTGATACCCCATATTTACAGGTTTATATATAAATAAAAAATGATTAAAAACTCAATTTTTCTCAATATTTATGGGA |
| *Pissonotus brunneus* | GAAGTTTATATTTTAATTTTACCAGGGTTTGGTTTAATTTCCCATATTATTATACAAGAAAGAGGTAGGAAAGAAACCTTTGGTTCAATCGGTATAATTTACGCAATAATCGCAATCGGAATCCTGGGGTTTATCGTATGAGCTCATCATATATTTACTGTGGGAATAGACATTGATACACGAGCATATTTTACCTCAGCAACCATAATTATTGCAGTACCGACAGGGATTAAAATTTTTAGATGAWTAGCAACTATCTACGGATCCAAAATTATTTATTCACCTCAAATAATTTGATCTATAGGATTCATTTTGCTTTTTACAATAGGTGGATTAACAGGTGTAATATTGGCTAATTCCTCTATTGATATCATTTTACATGACACTTATTATGTAGTTGCCCACTTCCATTATGTTCTTTCAATGGGAGCAGTATTTACTATTATTGCAAGATTTATCCACTGATACCCTGTATTTACAGGATTTTATATGAATAAAAAATGATTAAAAACTCAATTCTTTTCTATATTTTTAGGA |
| *Prokelisia dolus* | GAAGTGTATATTTTGATTCTCCCCGGATTCGGACTAATTTCTCATATTATTATACAGGAAAGAGGAAAAAAAGAAACATTTGGCTCAATTGGGATAATCTATGCCATGTTAGCTATTGGGATTCTAGGATTCATCGTTTGAGCACACCATATATTTACAGTGGGAATAGATATTGATACACGAGCCTACTTCACATCTGCCACAATAATTATTGCAGTACCTACAGGTATCAAAATTTTTAGATGAATTGCCACTATCTATGGATCAAAAATTAAATTTTCTCCACAAATAATTTGATCAATTGGATTCATTTTATTATTCACAATTGGGGGATTAACGGGAGTAATATTAGCAAATTCCTCTATTGACATTATCTTACACGACACTTATTATGTAGTTGCCCATTTTCACTATGTTCTTTCTATAGGAGCGGTATTTACTATTATTGCAAGATTTATCCACTGATATCCTCTGTTTACTGGAGTTTCAATAAACAGTAAATGACTTAGGGTA------------------------ |
| *Prokelisia marginata* | ---------ATTTTAATTCTCCCCGGATTCGGATTAATTTCTCATATTATTATACAGGAAAGAGGGAAAAAAGAAACATTTGGCTCAATTGGAATAATCTATGCCATGTTAGCTATTGGGATTCTAGGATTCATCGTTTGAGCACACCATATATTTACAGTAGGAATAGATATTGATACACGAGCCTACTTCACATCTGCCACAATAATTATTGCAGTACCTACTGGTATCAAAATTTTTAGATGAATTGCCACTATCTATGGATCAAAAATTAAGTTTTCTCCACAAATAATTTGATCAATTGGATTCATTTTATTATTCACAATTGGGGGACTGACGGGAGTAATGTTAGCAAATTCCTCTATTGACATTATCTTACACGACACTTATTATGTAGTTGCCCATTTTCATTATGTTCTTTCTATAGGAGCAGTATTTACTATTATTGCAAGATTTATCCATTGATATCCTCTATTTACTGGAGTTTCAATAAACAGTAAATGATTAAAGGTACAATTCTATGCCATATTTATTAGA |
| *Purohita sinica* | ---------------------------------------------------------------------------ACTTTTGGAACTATCGGAATAATTTACGCTATAATTGCTATTGGAATTCTAGGGTTTATTGTTTGAGCACATCATATATTTACTGTAGGAATAGATATTGATACTCGAGCATACTTTACTTCAGCTACAATAATTATTGCAGTCCCAACTGGAATTAAAATTTTTAGATGAATTGCTACAATTCACGGATCTAAAATTGTGTATTCACCTCAAATAATTTGATCAACAGGATTTATTTTCTTATTTACTATTGGAGGATTAACAGGGGTTATTCTAGCTAATTCTTCAATTGATATTATTTTACATGATACATATTATGTTGTTGCACATTTTCATTATGTTCTATCTATAGGAGCTGTATTTACAATTATTTCAAGATTTATTAATTGATACCCAGTATTTACAGGATTAGTATTAAATAATAAATGATTAAAAATTCAATTTTTTTGTATATTCATAGGA |
| *Purohita taiwanensis* | ---------------------------------------------------------------------------ACGTTTGGAACTATTGGAATAATTTACGCTATAATTGCCATCGGAATCCTAGGATTTATTGTTTGAGCTCATCATATATTCACAGTAGGTATAGATATTGATACACGAGCTTATTTTACATCAGCCACAATAATTATTGCAGTACCAACAGGAATTAAAATTTTTAGTTGAATCGCTACAATTCATGGATCTAAAATTATATTTACACCTCAAATAATTTGATCTACAGGATTTATTTTTCTATTCACGGTAGGTGGATTAACAGGAGTAATTTTAGCTAATTCATCAATTGATATTATTTTACATGATACATATTATGTTGTTGCACATTTTCATTATGTTCTATCTATAGGAGCCGTATTCACAATTATTTCAAGATTTATTAACTGATATCCAGTTTTTACAGGGCTAGTTATAAATAATAAATGATTAAAAATTCAATTTTTTTCCATATTTATAGGA |
| *Purohita theognis* | GAAGTTTACATTCTAATTCTTCCAGGATTTGGATTAATTTCCCATATTATTATACAAGAAAGAGGAAAAAAAGAAACATTTGGAACTATTGGAATAATTTACGCAATAATTGCTATTGGAATTTTAGGATTTATTGTTTGAGCACATCATATGTTTACTGTAGGAATAGATATTGATACACGAGCTTATTTTACTTCAGCTACAATAATTATTGCAGTTCCTACTGGAATTAAAATTTTCAGATGACTAGCCACAATTCATGGATCAAAAATCTTATTTTCACCCCAAATAATTTGATCTACCGGATTTATTTTCTTATTTACAATCGGAGGACTAACTGGAGTAATTTTAGCAAATTCCTCAATTGATATTATTTTACACGATACATATTATGTAGTTGCACATTTTCATTATGTCCTATCTATAGGAGCTGTATTCACAATTATTGCAAGATTTATTAATTGATATCCAGTATTTACAGGACTAATAATAAATATAAAATGACTAAAAATTCAATTTTTGTCAATATTTATTGGA |
| *Reptalus squadricinctus* | GAAGTTTACATTTTAATTCTACCAGGATTTGGATTAATTTCTCATATTATTATAAAAGAAAGAGGAAAAAAAGAAACATTTGGATCAATCGGAATAATTTATGCAATAATTGCTATTGGAATCCTAGGATTTGTAGTATGAGCTCATCATATATTTACAGTCGGAATAGATATTGATACACGAGCTTATTTTACATCAGCTACAATAATTATTGCAGTTCCAACAGGAATTAAAATTTTCAGATGAATAGCAACAATTTATGGAACAAAAATTAAATTCACACCACAAACTATATGAGCAATGGGATTCATTTTTTTATTTACCATAGGAGGATTAACAGGAGTAATTCTTGCAAATTCATCAATTGATATTATTTTGCACGATACATATTATGTAGTAGCTCATTTCCATTACGTTTTATCAATAGGAGCAGTATTTGCTATTATAGGAAGATTTATTGAATGATATCCATTAATAACAGGCTTATCAATAAACCAAAAATGACTAAAAATTCAATTCCTTACTATATTTACGGGA |
| *Rhombotoya psedomigripennis* | GAAGTGTACATCCTAATCTTGCCCGGATTTGGATTAATCTCACATATCATTATGCAAGAAAGAGGGAAAAAAGAAACATTTGGTTCAATTGGAATAATCTACGCAATAATCGCTATCGGGATTCTGGGATTTATCGTCTGAGCTCATCATATATTCACTGTAGGTATAGATATTGATACACGAGCATACTTCACTTCAGCAACAATAATCATTGCTGTGCCAACAGGTATTAAAATTTTTAGATGACTAGCAACAATTTATGGATCAAAAATTTCATTTTCCCCCCAAATAATTTGATCAATGGGATTTATCCTACTTTTTACAATTGGGGGACTTACTGGTGTAATACTAGCAAACTCATCGATTGACATCATTTTACATGACACCTATTATGTAGTTGCTCACTTCCACTATGTACTTTCCATAGGAGCAGTATTCACAATTATCGCAAGATTTATTCACTGATATCCTATCTTCACAAGTACATCAATAAATCTAAAATGATTAAAA--------------------------- |
| *Ribautodelphax bidentatus* | GAAGTGTACATCCTAATCCTCCCAGGATTTGGATTGATTTCACATATTATTATACAGGAAAGAGGAAAAAAGGAAACTTTTGGTTCAATTGGAATAATTTACGCAATAATAGCTATTGGAATTTTAGGGTTTATTGTATGAGCCCACCATATATTTACTGTTGGTATAGATATTGATACGCGAGCCTATTTTACATCAGCGACTATAATTATTGCTGTACCAACCGGAATTAAAATTTTTAGATGAATTGCAACAATCTATGGGTCTAAAATTAACTTCTCACCCCAAATAATTTGATCTATCGGATTCATTTTATTATTTACAATTGGTGGATTGACAGGAGTTATATTAGCCAACTCCTCTATTGATATCATTTTACACGATACATACTACGTAGTAGCCCATTTCCACTACGTACTCTCAATAGGAGCTGTTTTCACTATTGTAGCCAGTTTTATCCATTGATACCCCCTATTTACAGGTGTTTCACTAAACAATAAATGACTAAAAATTCAATTCACTTCCATATTTATTGGA |
| *Ribautodelphax tuvinus* | GAAGTGTATATCCTAATCCTCCCAGGATTTGGATTGATTTCACATATTATTATACAGGAAAGAGGAAAAAAGGAAACTTTTGGTTCAATTGGAATAATTTACGCAATAATAGCTATTGGAATTTTAGGGTTTATTGTATGAGCCCACCATATATTTACTGTTGGTATAGATATTGATACGCGAGCCTATTTTACATCAGCGACTATAATTATTGCTGTACCAACCGGAATTAAAATTTTTAGATGAATTGCAACAATCTATGGGTCTAAAATTAACTTTTCACCCCAAATAATTTGATCTATCGGATTCATTTTATTATTTACAATTGGTGGATTAACAGGAGTTATATTAGCCAACTCCTCTATTGATATCATTTTACACGATACATACTACGTAGTAGCCCATTTCCACTACGTACTCTCAATAGGAGCTGTTTTCACTATTGTAGCCAGTTTTATCCATTGATACCCCCTATTTACAGGTGTTTCACTAAACCATAAATGACTAAAAATTCAATTCACTTCCATATTTATTGGA |
| *Saccharosydne procerus* | GAAGTTTATATTTTAATTTTACCAGGATTTGGACTAATTTCACATATTATTATACAAGAAAGAGGGAAAAAAGAAACATTTGGATCTATTGGAATAATTTATGCTATAATTGCCATTGGAATTTTAGGATTTATTGTATGAGCACATCACATATTCACAGTAGGAATAGATATTGATACTCGAGCATATTTTACCTCAGCCACAATAATTATTGCAGTACCTACTGGGATTAAAATTTTTAGATGACTAGCAACAATTCACGGGTCAAAAATAATTTATTCACCCCAAATAATTTGATCTACGGGATTTATTTTTCTATTTACAGTAGGAGGATTAACTGGTGTAATCTTAGCAAATTCATCTATTGATATTATTCTTCATGATACTTATTATGTAGTAGCCCACTTCCATTATGTATTATCCATAGGAGCAGTCTTCACTATTATTGCAAGATTTATTAATTGATATCCAATTATAACAGGAATATTAATAAACAAAAAATGATTAAAAATTCAATTTATAACTATATTTTTAGGA |
| *Sardia rostrota* | GAAGTTTATATCCTTATTCTCCCCGGGTTTGGACTAATTTCACACATTATTATACAAGAAAGAGGAAAACGTGAAACCTTTGGTTCAATCGGTATAATTTATGCCATATTAGCAATTGGGATTTTAGGATTCATCGTTTGAGCACACCACATATTCACTGTAGGGATAGATATTGATACACGAGCATATTTTACTTCAGCCACTATAATTATTGCTGTACCTACCGGAATTAAAATTTTTAGATGAATTGCCACTATTTACGGATCTAAAATTAAATTTTCCCCCCAAATAATTTGATCAATAGGATTCATTTTACTCTTTACAATTGGTGGATTAACTGGAGTAATACTAGCCAATTCATCAATTGATATTGTTCTTCATGATACTTATTATGTAGTAGCCCACTTTCACTATGTACTATCTATAGGAGCAGTTTTTACCATCATTGCTAGATTTATTCATTGATACCCTGTATTTACAGGACTTTCAATAAACAAAAATTGATTAAAAATTCAATTTTTTTCTATATTTATGGGA |
| *Shadelphax eforiae* | ---------------------------TTTGGATTAATCTCTCACATTATCATGCAAGAGAGAGGAAGGAAAGAAACATTTGGATCTATTGGGATAATCTACGCCATAATTGCCATCGGTATATTAGGATTTATTGTATGAGCCCACCACATATTTACTGTAGGTATAGATATCGATACACGAGCCTATTTTACATCAGCAACTATAATTATTGCTGTACCCACAGGTATTAAAATCTTTAGATGAATCGCCACAATCCATGGATCAAAAATTAATTATTCCCCCCAAATAATCTGATCTTTAGGATTCATTTTATTATTCACAATTGGTGGACTAACCGGAGTTATATTAGCCAATTCTTCAATTGATGTAATTTTACACGACACCTATTACGTAGTGGCCCATTTCCATTACGTCCTCTCAATAGGAGCTGTATTTACAATTATTGCAAGATTTATCCACTGATTCCCCCTTTTTACAGGAGTGACACTAAATAATAAATGACTAAAAATCCAATTTACCTCAATGTTTAGAGGA |
| *Sogata hakonensis* | GAAGTTTACATTCTGATCCTACCAGGATTTGGATTAATTTCACATATCATTATACAAGAAAGAGGGAAAAAAGAAACTTTTGGATCTATCGGGATAATTTATGCAATAATCTCAATTGGAATTTTAGGTTTCATCGTGTGAGCCCACCATATATTTACAGTTGGAATAGACATTGATACACGAGCATACTTTACTTCTGCTACAATAATTATTGCTGTCCCCACAGGAATTAAAATTTTTAGTTGAGTAGCAACAATTTTTGGATCAAAAGTAAATAATTCCCCTCAAATAATTTGATCAATAGGGTTTATTTTACTATTTACTATAGGTGGATTAACAGGAGTAATATTAGCTAATTCATCAATCGATATTGTTCTTCACGACACCTATTATGTAGTAGCCCACTTCCATTATGTTCTTTCTATAGGTGCTGTATTCACTATTATTGCAAGATTTATCCACTGATACCCTCTATTTACAGGATCCTCATTAAATAAAAAATGACTAAAAATTCAATTTTTTTCAATATTTACAGGA |
| *Sogata nigrifrons* | GAAGTTTACATTCTGATCCTACCAGGATTTGGATTAATTTCACATATCATTATACAAGAAAGAGGGAAAAAAGAAACTTTTGGATCTATCGGGATAATTTATGCAATAATCTCAATTGGAATTTTAGGTTTCATCGTGTGAGCCCACCATATATTTACAGTTGGAATAGACATTGATACACGCGCATACTTTACTTCTGCTACAATAATTATTGCTGTCCCCACAGGAATTAAAATTTTTAGTTGAGTAGCAACAATTTTTGGATCAAAAGTAAATAATTCCCCTCAAATAATTTGATCAATAGGGTTTATTTTACTATTTACTATAGGTGGATTAACAGGAGTAATATTAGCTAATTCATCAATCGATATTGTTCTTCACGACACCTATTATGTAGTAGCCCACTTCCATTATGTTCTTTCTATAGGTGCTGTATTCACTATTATTGCAAGATTTATCCACTGATACCCTCTATTTACAGGATCCTCATTAAATAAAAAATGACTAAAAATTCAATTTTTTTCAATATTTACAGGA |
| *Sogatella furcifera* | GAAGTTTATATCCTGATTCTCCCCGGATTTGGATTAATTTCCCATATCATTATACAAGAAAGAGGTAAACGTGAAACCTTTGGATCAATTGGTATAATCTACGCCATACTAGCTATTGGAATCCTAGGATTTATCGTTTGAGCACACCATATATTTACAGTAGGAATAGATATTGATACACGAGCGTACTTTACTTCAGCGACAATAATTATTGCTGTACCTACAGGAATTAAAATTTTTAGATGGATCGCCACCATTTACGGATCAAAAATTAATTTTTCCCCCCAAATAATTTGGTCTATAGGATTCATTTTGCTTTTTACAATTGGTGGTCTAACAGGAGTAATACTAGCAAACTCCTCAATCGATGTTGTTCTTCATGATACCTACTATGTAGTTGCTCACTTTCACTATGTTTTGTCTATAGGAGCCGTTTTTACAATTGTTGCCAGTTTCATCCACTGGTACCCAATTTTTACTGGAGTTGCCTTAAACAATAAATGACTAAAAATTCAATTTTTTTCTATATTTTTAGGA |
| *Spartidelphax detectus* | GAAGTGTACATTCTTATCCTCCCAGGATTTGGATTAATTTCACACATCATTATACAAGAAAGAGGAAAAAAGGAAACCTTTGGATCAATTGGTATAATTTATGCAATAATAGCTATTGGTATTTTAGGGTTTATTGTTTGAGCTCATCATATGTTCACAGTAGGGATAGATATCGACACACGGGCCTACTTTACTTCAGCAACTATAATTATTGCAGTTCCTACAGGAATTAAAATTTTCAGATGAATTGCAACAATTTACGGTTCTAAAATTAAATTTTCTCCCCAAATAATTTGATCTATAGGATTCATTTTATTATTTACAATTGGTGGATTAACAGGAGTAATACTTGCAAATTCTTCAATTGATATTGTATTACATGACACCTATTATGTTGTAGCCCATTTCCACTACGTACTTTCTATGGGAGCAGTTTTTACTATTATCGCAAGATTTATTCATTGATACCCAATTTTTACAGGGCTTTCCTTAAATATAAAATGATTAAAAATTCAATTTTACTCTATATTCATTGGA |
| *Specinervures basifusca* | GAAGTTTATATTCTAATTCTACCCGGATTTGGATTAATTTCACATATTATTATGCAAGAAAGAGGAAAAAAAGAAACATTTGGTACAATTGGTATAATCTACGCTATAATTGCTATCGGAGTCTTAGGATTTATTGTATGAGCCCATCATATATTTACAGTAGGTATAGATATTGATACACGAGCTTACTTCACTTCAGCTACAATAATTATTGCAGTCCCCACTGGAATTAAAATTTTTAGATGACTGGCAACAATTCACGGATCAAAAATTTCATTTTCCCCACAAATAATTTGATCCACCGGATTCATTTTCCTTTTTACAATCGGTGGACTAACAGGAGTAATTTTAGCTAATTCATCTATTGACATTATCCTTCATGATACATATTATGTAGTTGCACACTTCCACTATGTTTTATCTATAGGTGCTGTATTCACAATTATTGCAAGATTTATTAACTGATATCCAGTCTTCACCGGATTATCAATAAATTTAAAATGATTAAAAATTCAATTTTCATCAATATTTATAGGA |
| *Stenocranus montanus* | GAAGTATACATTTTAATTTTACCTGGATTTGGATTAATTTCACATATTATTATACAAGAAAGAGGAAAAAAAGAAACTTTTGGATCTATTGGAATAATTTACGCAATAATTGCTATTGGAGTACTAGGATTTATTGTTTGAGCACATCATATATTTACAGTAGGAATAGATATTGATACACGTGCTTACTTTACATCAGCAACTATAATTATTGCTGTACCTACAGGAATTAAAGTATTTAGATGACTAGCTACCATTTACGGATCAAAAATTAAATTTTCACCTCAAATAATTTGATCAATTGGTTTCATTTTCCTTTTCACAATTGGTGGATTAACAGGTGTAATTTTAGCCAATTCATCAATTGACACTATTTTACACGACACATATTATGTAGTTGCTCACTTTCACTATGTTTTATCAATAGGAGCAGTATTTACAATTATTTCCAGATTTATTCATTGATACCCATTATTTACAGGTACAATAATAAACAAAAAATGATTAAAAATTCAATTTTTATCAATATTCACAGGA |
| *Stiropis nigrifrons* | GAAGTTTACATCCTAATCCTCCCCGGATTTGGATTAATTTCACATATTATTATGCAAGAAAGAGGAAAAAAAGAAACATTTGGTTCAATTGGGATAATTTACGCAATAATCGCAATTGGTATTTTAGGATTCATTGTTTGAGCTCATCACATATTTACAGTTGGAATAGATATTGATACACGAGCCTACTTTACCTCAGCAACCATAATTATTGCTGTACCCACAGGTATCAAAATTTTTAGTTGAATCGCCACAATCTACGGGTCTAAAATTAACTTTTCACCCCAAATAATTTGATCAATAGGATTCATTTTATTATTTACAATCGGTGGACTGACAGGAGTTATACTGGCTAATTCATCCATTGATATTGTATTACATGACACTTACTATGTAGTAGCCCATTTCCACTATGTACTTTCCATAGGTGCTGTATTCACTATTATTGCAAGATTTATCCACTGATACCCACTATTTACAGGTGTATCGATAAATAATAAATGACTAAAAATCCAATTTATTTCAATATTCACAGGA |
| *Struebingianella detecta* | GAAGTTTATATCCTGATTCTCCCCGGATTCGGATTAATCTCCCACATTATTATACAAGAAAGAGGTAAAAAAGAAACATTCGGCTCAATCGGTATAATTTACGCAATAATTGCAATTGGTATCCTAGGGTTCATTGTATGGGCCCACCACATATTTACAGTTGGTATAGACATCGATACACGAGCTTATTTCACATCAGCAACGATAATTATTGCTATCCCGACAGGGATCAAAATTTTTAGATGAATCGCAACAATCTACGGGTCAAAAATTAATTTTTCCCCTCAAATAATTTGATCTATAGGATTCATTTTACTGTTTACAATTGGTGGACTAACAGGAGTTATATTAGCTAATTCCTCCATTGACATTATCCTACATGATACCTATTATGTTGTAGCACACTTTCACTATGTGTTGTCAATAGGAGCTGTATTCACCATTATTGCAAGATTTATTCATTGATACCCCCTATTTACAGGAGCCTCATTAAATAAAAAATGATTAAAAATTCAATTTATTTCAATATTTACAGGA |
| *Sulculus sulcatus* | GAAGTATATATTCTAATCCTACCAGGATTTGGACTAATCTCCCATATTATTATACAAGAAAGAGGAAAAAAAGAAACATTTGGGTCAATTGGAATAATTTATGCAATAATGGCTATTGGAGTTCTAGGATTTATTGTATGAGCTCATCATATATTTACTGTAGGAATAGATATTGATACGCGAGCCTACTTTACTTCAGCAACTATAATCATTGCGGTACCTACCGGTATTAAAGTATTTAGATGAATAGCAACTATCTATGGATCCAAAATCTCATTCTCACCACAAATAATTTGATCAATAGGATTCATTTTACTTTTTACAATTGGTGGTTTAACAGGTGTAATACTAGCAAATTCATCAATTGATATTATTTTACATGATACATATTATGTAGTTGCTCATTTTCACTACGTTCTTTCAATAGGAGCAGTTTTTACAATTATTGCAAGATTTATCCACTGATACCCCCTTCTTACAGGTATATCAATTAACAATAAATGATTAAAAATTCAATTTTATTCAATATTTCTAGGA |
| *Tagosodes orizicolus* | GAAGTTTATATTCTCATTCTTCCAGGGTTTGGATTAATTTCACACATTATTATACAAGAAAGAGGAAAACGAGAAACTTTCGGTTCAATTGGAATAATTTACGCCATACTAGCAATTGGTATCCTAGGATTTATTGTGTGAGCACATCACATATTTACTGTAGGAATAGATATTGATACAAAAGCCTACTTTACCTCAGCAACAATAATTATTGCTGTTCCAACAGGAATTAAAATCTTCAGTTGAATTGCCACAGTTTATGGATCTAAAATTAATTTATCTCCCCAAATAATTTGATCTATAGGATTCATTTTACTATTCTCAATTGGTGGACTTACTGGAGTTATATTAGCTAATTCATCAATTGATATTGTCCTCCATGACACTTACTATGTAGTAGCACACTTCCACTACGTTTTATCCATAGGAGCTGTATTTACAATTATTGCAAGATTTATCCATTGATACCCTATTTTTACAGGAGTTTTAATAAACAAAAAATGATTAAAAATTCAATTTATTTCAATATTTGTGGGA |
| *Tarophagus colocasiae* | GAAGTATACATTCTTATTCTCCCAGGATTTGGATTAATTTCACATATCATTATAAGAGAAAGAGGCAAAAAAGAAACATTTGGATCAATCGGAATAATTTATGCAATAATCGCTATTGGAATCTTAGGTTTTATTGTTTGAGCTCACCATATATTCACTGTAGGTATGGATATTGACACTCGAGCCTACTTCACCTCAGCTACTATAATTATTGCAGTTCCAACTGGCATCAAAATCTTTAGATGAATAGCAACAATTTATGGATCAAAAATTATATTTTCACCTCAAATAATCTGATCCATAGGATTTATTTTACTATTTACAATTGGGGGGTTAACAGGAGTAATACTAGCAAATTCCTCAATTGATATTGTATTACACGATACATACTATGTAGTTGCACACTTTCACTATGTTCTCTCAATAGGAGCAGTTTTTACAATTATTGCAAGATTTATTCATTGATACCCCCTATTTACAGGAACTTTAATAAATAATAAATGACTAAAAATTCAATTTTTCTCAATATTTACAGGA |
| *Terauchiana singularis* | GAAGTTTACATTTTAATTTTACCTGGATTTGGCTTAATTTCACATATTATTATACAAGAAAGAGGAAAAAAAGAAACATTTGGATCAATTGGAATAATTTACGCAATAATTGCAATTGGAATTTTAGGATTCATTGTTTGAGCTCACCACATATTTACTGTAGGAATAGATATTGATACACGTGCTTACTTTACATCAGCCACCATAATTATTGCCGTACCTACAGGAATTAAAGTATTTAGATGACTAGCAACTATTTACGGATCAAAAATTAATTTTTCACCACAAATAATTTGATCGATTGGATTTATTTTCCTTTTCACTATTGGTGGACTAACAGGAGTTATTCTTGCTAACTCATCAATTGACACAATTTTACATGATACATATTATGTAGTTGCTCACTTTCACTATGTTTTATCAATAGGAGCAGTTTTCACAATCATTTCAAGTTTTATTCATTGATATCCCTTATTTACAGGAACAATAATAAACAAAAAATGACTAAAAATTCAATTTTTTTCAATATTTACAGGA |
| *Terthron albovittatum* | GAAGTTTATATTTTAATTTTACCCGGATTCGGTTTAATTTCACACATTATTATACAAGAAAGAGGAAAACGAGAGACATTTGGATCAATTGGAATAATTTATGCAATGTTAGCTATTGGAATTTTAGGATTTATTGTATGAGCTCATCATATATTTACGGTTGGTATAGACATTGATACACGAGCATATTTCACTTCAGCCACTATAATTATTGCTGTACCAACAGGAATCAAAATCTTTAGATGAATTGCAACAATCTACGGATCCAAAATTAAATTCTCACCCCAAATAATTTGATCTCTAGGATTCATTTTACTTTTTACAATTGGAGGATTAACAGGAGTAATATTAGCTAATTCATCTATTGATATTGTTATACATGACACATATTATGTAGTAGCCCATTTTCACTATGTATTATCAATAGGGGCAGTATTCACTATTGTAGCAAGTTTTATTCACTGATATCCATTATTTACAGGAGTAACCATAAATAATAAATGATTAAAAATTCAATTTACTGCAATATTTATAGGA |
| *Toya terryi* | GAAGTTTATATTTTAATTCTACCAGGATTTGGATTAATTTCACATATTATTATACAGGAAAGAGGTAAGCGAGAAACCTTTGGATCAATCGGTATAATTTACGCTATACTAGCTATTGGAATTCTAGGATTTATTGTGTGAGCCCACCACATATTTACTGTTGGTATAGACATTGATACTCGAGCTTACTTTACATCAGCAACTATAATTATTGCTGTCCCCACAGGAATTAAAATTTTTAGTTGAATTGCAACGATTTATGGATCTAAAATTAGATTTTCCCCTCAAATAATTTGATCAATAGGATTCATTTTACTTTTCACTATTGGTGGTTTAACAGGAGTAATACTTGCTAATTCATCAATTGATATCGTTCTTCATGATACGTATTATGTTGTAGCTCACTTTCACTATGTACTTTCTATAGGTGCTGTATTTACAATTGTAGCCAGATTTATTCACTGATACCCAATTTTTACTGGTTTATCAATAAATAAAAAATGATTAAAAATTCAATTTAGTTCAATATTTATTGGA |
| *Toyoides albipennis* | GAAGTCTATATTTTAATTTTACCAGGATTTGGATTAATTTCACATATTATTATAAGAGAAAGAGGTAAAAAAGAAACATTTGGATCAATCGGAATAATCTATGCTATAATTGCCATTGGAATTCTAGGATTTATTGTTTGAGCACATCATATATTCACAGTTGGGATAGATATTGATACACGAGCCTATTTTACTTCAGCTACAATAATTATTGCTGTTCCTACAGGAATTAAAATTTTTAGTTGAATAGCTACAATTTATGGATCAAAAATTATTTATTCACCCCAAATAATTTGATCAATAGGTTTCATTTTACTTTTTACAATCGGAGGATTGACTGGTGTAATACTAGCAAATTCATCAATTGACATTGTATTACATGATACATACTATGTAGTAGCCCACTTCCATTACGTACTTTCAATAGGAGCAGTATTCACCATTATTGCAAGTTTTATTCAATGATACCCACTTTTTTCAGGTACATCAATAAACAACAAATGATTAAAAATTCAATTCTTAACAATATTTACAGGA |
| *Tropidocephala brunnipennis* | GAAGTTTACATTTTAATTTTACCAGGATTTGGACTAATTTCACATATCATTATACAAGAAAGAGGTAAAAAAGAAACTTTTGGATCAATTGGAATAATCTATGCAATAATCGCAATTGGTATTTTAGGTTTCATTGTTTGAGCTCATCACATATTTACAGTTGGAATAGATATTGATACACGAGCTTATTTTACTTCAGCCACTATAATTATTGCTGTACCTACAGGAATCAAAATCTTTAGATGAATTGCTACAATTCACGGATCAAAAATTAATTTTTCTCCTCAAATAATTTGATCTACTGGATTCATTTTCCTATTTACAATTGGAGGATTAACAGGAGTTATTTTAGCTAATTCATCAATCGATATTATTTTACATGACACTTATTATGTAGTTGCTCATTTTCATTATGTACTCTCAATAGGAGCAGTATTTACTATTATTTCTAGATTTATCAATTGATACCCTGTACTTACAGGCATTTCAATAAATAATAAGTGATTAAAAATTCAATTCATAACCATATTTATAGGA |
| *Tropidocephala festiva* | GAAGTGTATATCTTAATTCTACCAGGATTTGGATTAATTTCACATATCATTATACAAGAAAGAGGAAAAAAAGAAACTTTTGGATCAATTGGAATAATTTATGCAATAATCGCAATTGGCATTTTAGGATTCATTGTATGAGCTCATCATATATTTACAGTTGGAATAGATATTGATACACGAGCCTACTTTACATCAGCTACTATAATTATTGCTGTACCCACCGGAATCAAAATCTTTAGTTGAATTGCTACAATTCACGGATCAAAAATTAACTTTTCCCCCCAAATAATTTGATCTACTGGATTCATTTTCCTATTTACAATTGGAGGATTAACAGGAGTAATTTTAGCTAATTCATCAATTGATATTGTTTTACATGACACTTACTATGTAGTTGCACACTTTCACTATGTACTTTCAATGGGAGCAGTATTCACCATCATTTCAAGATTTATTAATTGATACCCTGTACTTACAGGTACCTCAATAAATATCAAATGATTAAAAATTCAATTTATAGCTATATTTATAGGA |
| *Tropidocephala* sp. | GAAGTTTATATTTTAATTTTACCAGGATTTGGACTAATTTCACATATCATTATACAAGAAAGAGGAAAAAAAGAAACTTTTGGATCAATTGGAATAATCTATGCAATAATCGCAATTGGTATCTTAGGTTTTATTGTTTGAGCTCATCACATATTCACAGTTGGAATAGATATTGATACACGAGCCTACTTCACTTCGGCCACTATAATTATTGCTGTACCTACAGGAATCAAAATCTTTAGCTGAATTGCTACAATTCACGGATCAAAAATTAATTTTTCTCCCCAAATAATTTGATCCACTGGATTCATTTTTCTATTTACAATTGGAGGATTAACGGGAGTTATTTTAGCTAATTCATCAATTGATATTATTTTACATGACACTTATTATGTAGTTGCTCACTTTCATTATGTACTCTCAATAGGAGCAGTATTTACTATTATTTCCAGATTTATCAATTGATACCCTGTACTTACAGGTATTTCAATAAACAATAAATGATTAAAAATTCAATTCATAACTATATTTATAGGA |
| *Ulanar muiri* | GAAGTGTACATTTTAATTCTACCTGGATTTGGATTAATTTCACACATCATTATACAAGAAAGAGGTAAACGAGAAACTTTTGGAAATATCGGTATAATTTATGCCATAATAGCTATTGGAGTTTTAGGTTTTATTGTATGAGCACACCATATATTTACTGTTGGAATAGATATTGATACTCGAGCTTACTTCACCTCAGCAACTATAATTATTGCTGTACCTACTGGAATTAAGATTTTTAGATGAATTGCAACAATCTATGGATCAAAAATTAATTATTCTCCCCAAATAATATGATCAATAGGATTCATTTTACTTTTTACAATTGGTGGATTAACAGGAGTTATATTAGCTAATTCATCAATTGATGTAGTTCTACATGACACTTACTACGTAGTAGCTCACTTCCATTATGTACTTTCAATAGGAGCTGTGTTTACAATTATTGCTAGATTTATCCACTGATACCCAATCCTTACAGGCTTAATAATAAACAAAAAATGATTAAAAATTCAATTTTGTTCAATATTTATAGGA |
| *Unkanodes sapporona* | GAAGTATACATTTTAATCCTACCAGGGTTTGGATTAATTTCACATATCATTATACAAGAAAGAGGTAAAAAAGAAACTTTTGGCTCAATTGGTATAATTTACGCAATAATCGCTATTGGGATTTTAGGGTTTATTGTATGAGCTCACCATATGTTTACTGTTGGCATAGATATTGACACACGAGCCTACTTTACATCAGCAACTATAATTATTGCTGTACCAACAGGAATTAAAATCTTTAGATGAATTGCAACGATTTATGGATCTAAAATTAACTTTTCACCTCAAATAATTTGATCCATTGGATTCATTTTATTATTCACTATTGGTGGATTAACAGGGGTAATGTTAGCAAATTCATCTATCGATATCATTTTACATGACACCTACTATGTAGTAGCCCATTTCCACTACGTACTTTCCATAGGAGCTGTTTTCACTATTGTAGCCAGTTTTATTCACTGATACCCTCTATTTACAGGTGTTTCATTAAACAGTAAATGACTAAAGATTCAATTTACTTCTATATTTATTGGA |

**Supplementary** **Table S5** List of species investigated and their related information.

| Taxa | Collected location | Date | GenBank accession numbers | | | |
| --- | --- | --- | --- | --- | --- | --- |
|  |  |  | *cox1* | *cytb* | 16S rDNA | 28S rDNA |
| Delphacinae |  |  |  |  |  |  |
| Saccharosydnini |  |  |  |  |  |  |
| *Saccharosydne procerus* (Matsumura) | China (LN) | 31. vii. 2009 | KX246193 | KX246124 | KX245925 | KX246024 |
| Tropidocephalini |  |  |  |  |  |  |
| *Arcofaciella verrucosa* Fennah | China (GD) | 21. viii. 2010 | KX246175 | KX246110 | KX245907 | KX246003 |
| *Arcofacies maculatipennis* Ding | China (GZ) | 31. vii. 2012 | KX246171 | KX246107 | KX245903 | KX246000 |
| *Arcofacies strigatipennis* Ding | China (FJ) | 29. vii. 2009 | KX246184 |  | KX245916 | KX246014 |
| *Bambusiphaga fascia* Huang & Tian | China (GZ) |  | KX246167 | KX246104 | KX245899 | KX245996 |
| *Bambusiphaga hainanensis* Hou & Chen | China (HN) | 30. vii. 2010 | KX246172 | KX246108 | KX245904 | KX246001 |
| *Bambusiphaga kunmingensis* Yang & Chen | China (YN) | 08. viii. 2011 | KX246164 |  |  | KX245993 |
| *Bambusiphaga luodianensis* Ding | China (GZ) | 29. vii. 2012 | KX246176 | KX246111 | KX245908 | KX246004 |
| *Bambusiphaga maculata* Chen & Li | China (HN) | 26. vii. 2013 | KX246179 | KX246113 | KX245910 | KX246007 |
| *Bambusiphaga membranacea* Yang & Yang | China (GZ) | 27. vii. 2012 |  | KX246114 | KX245911 | KX246008 |
| *Bambusiphaga mirostylis* Huang & Ding | China (YN) | 22. v. 2011 | KX246180 | KX246115 | KX245912 | KX246010 |
| *Bambusiphaga nigropunctata* Huang & Ding | China (SC) | 19. vii. 2011 | KX246170 |  | KX245902 | KX245999 |
| *Bambusiphaga* *taibaishana* Qin | China (SX) | 15. viii. 2011 | KX246183 | KX246117 | KX245915 | KX246013 |
| *Belocera sinensis* Muir |  |  | HM233889 |  | HM233801 | HM233832 |
| *Carinofrons maculatipennis* Chen & Li | China (YN) | 01. viii. 2010 | KX246163 | KX246102 | KX245896 | KX245992 |
| *Epeurysa distincta* Huang & Ding | China (HN) | 26. vii. 2013 | KX246188 | KX246120 | KX245920 | KX246018 |
| *Epeurysa infumata* Huang & Ding | China (GZ) | 08. viii. 2012 | KX246191 | KX246122 | KX245923 | KX246022 |
| *Epeurysa nawaii* Matsumura | China (GZ) | 06. vii. 2013 | KX246166 | KX246103 | KX245898 | KX245995 |
| Gen. nov. 1 | China (HN) | 30. vii. 2010 | KX246189 | KX246121 | KX245921 | KX246019 |
| *Lauriana senticosa* Ren & Qin | China (SC) | 07. vii. 2013 | KX246187 |  | KX245919 | KX246017 |
| *Malaxa semifusca* Yang & Yang | China (HN) | 26. vii. 2013 | KX246162 | KX246101 | KX245895 | KX245991 |
| *Malaxella* *macracantha* Ren & Qin | China (YN) | 22. v. 2011 | KX246190 |  | KX245922 | KX246020 |
| *Malaxella flava* Ding & Hu | China (FJ) | 29. vii. 2009 | KX246173 |  | KX245905 |  |
| *Malaxella tetracantha* Qin & Zhang | China (FJ) | 01. ix. 2008 | KX246181 |  | KX245913 | KX246011 |
| *Neobelocera* sp. |  |  | HM233922 |  | HM233813 | HM233875 |
| *Neocarinodelphax hainanensis* (Qin & Zhang) | China (HN) | 30. vii. 2010 | KX246177 | KX246112 | KX245909 | KX246005 |
| *Purohita sinica* Huang & Ding | China (GZ) | 27.vii. 2012 | KX246192 | KX246123 | KX245924 | KX246023 |
| *Purohita taiwanensis* Muir | China (HN) | 04. viii. 2010 | KX246185 | KX246118 | KX245917 | KX246015 |
| *Purohita theognis* Fennah | China (HN) | 25. viii. 2002 | KX246186 | KX246119 | KX245918 | KX246016 |
| *Specinervures basifusca* Chen & Li | China (SC) | 19. vii. 2011 | KX246174 | KX246109 | KX245906 | KX246002 |
| *Tropidocephala brunnipennis* Signoret | China (HN) | 25. vii. 2010 | KX246169 | KX246106 | KX245901 | KX245998 |
| *Tropidocephala festiva* (Distant) | China (GZ) | 06. viii. 2012 | KX246168 | KX246105 | KX245900 | KX245997 |
| *Tropidocephala* sp. | China (HN) | 27. vii. 2013 | KX246182 | KX246116 | KX245914 | KX246012 |
| Delphacini |  |  |  |  |  |  |
| *Aoyuanus furcatus* Ding & Chen | China (FJ) | 16. viii. 2008 | KX246205 |  | KX245936 | KX246040 |
| *Bakerella minuta* Beamer | USA (FL) |  | HM017488 |  |  | HM017360 |
| *Bostaera nasuta* Ball | USA (UT) |  | HM017489 |  |  | HM017361 |
| *Caenodelphax teapae* (Fowler) | USA (FL) |  | HM017471 |  |  | HM017321 |
| *Cemopsis griphus* Fennah | China (YN) | 21. v. 2011 | KX246245 | KX246149 | KX245974 | KX246085 |
| *Cemus macaoensis* (Muir) | China (YN) | 21. v. 2011 | KX246195 | KX246125 |  | KX246027 |
| *Cemus nigromaculosus* (Muir) | China (FJ) | 05. ix. 2008 | KX246221 | KX246139 | KX245951 | KX246058 |
| *Cemus punctatus* (Muir) | China (YN) | 21. v. 2011 | KX246203 | KX246128 | KX245931 | KX246033 |
| *Cemus sauteri* (Muir) | China (YN) | 21. v. 2011 | KX246247 | KX246150 | KX245976 | KX246086 |
| *Criomorphus niger* Ding & Zhang | China (NX) | 10. vii. 2008 | KX246226 |  | KX245956 | KX246063 |
| *Delphacodes puella* (Van Duzee) | USA (PA) |  | HM017484 |  |  | HM017348 |
| *Delphax orientalis* (Linnavuori) | Kyrgyzstan |  | HM017496 |  |  | HM017390 |
| *Dicranotropis hamata* (Boheman) | Germany |  | HM017498 |  |  | HM017403 |
| *Distantinus melichari* (Kirkaldy) | China (HN) | 15. v. 2008 | KX380848 |  | KX380844 | KX380846 |
| *Ecdelphax cervina* (Muir) | China (JX) | 19. vii. 2013 | KX246209 |  | KX245940 | KX246045 |
| *Ecdelphax dentata* Yang | China (FJ) | 30. vii. 2013 | KX246206 | KX246132 | KX245937 | KX246042 |
| *Elachodelphax metcalfi* (Kusnezov) | China (NX) | 10. vii. 2008 | KX246237 |  | KX245967 |  |
| *Eurybregma nigrolineata* Scott | China (HB) | 24. vi. 2009 | KX246220 |  | KX245950 | KX246057 |
| *Falcotoya lyraeformis* (Matsumura) | China (JX) | 26. vii. 2013 | KX246244 | KX246148 | KX245973 | KX246083 |
| *Fangdelphax gongshanensis* Ding | China (SC) | 15. vii.2011 | KX246213 |  | KX245943 | KX246050 |
| *Flavoclypeus nigrifacies* Muir (as *Delphacodes nigrifacies*) | USA (FL) |  | HM017473 |  |  | HM017328 |
| *Hadeodelphax afurculus* Guo & Liang | China (GD) | 24. viii. 2010 | KX246249 | KX246151 | KX245978 | KX246088 |
| *Harmalia aculeatus* (Yang) | China (HN) | 03. viii. 2010 |  |  | KX245960 | KX246069 |
| *Harmalia gayasana* (Kwon) | China (SC) | 17. vii. 2011 | KX246234 | KX246145 | KX245964 | KX246071 |
| *Harmalia heitensis* (Matsumura & Ishihara) | China (HN) | 27. v. 2007 | KX246216 |  | KX245946 | KX246053 |
| *Harmalia ostorius* (Kirkaldy) | Australia |  | HM017505 |  |  | DQ532596 |
| *Harmalia sirokata* (Matsumura & Ishihara) | China (SC) | 17. vii. 2011 | KX246201 | KX246130 | KX245933 | KX246036 |
| *Harmalia tiphys* Fennah | China (HN) | 31. vii. 2009 | KX246224 |  |  | KX246061 |
| *Himeunka tateyamaella* (Matsumura) | China (JX) | 23. vii. 2013 | KX246210 | KX246135 | KX245941 | KX246046 |
| *Ishiharodelphax matsuyamensis* (Ishihara) | China (FJ) | 30. vii. 2013 | KX246250 | KX246152 | KX245979 | KX246089 |
| *Isodelphax basivitta* (Van Duzee) | USA (PA) |  | HM017485 |  |  | HM017349 |
| *Javesella dubia* (Kirschbaum) | China (SX) | 05. vii. 2010 | KX246252 |  |  | KX246091 |
| *Javesella obscurella* (Boheman) | China (LN) | 31. vii. 2009 | KX246194 |  | KX245926 | KX246026 |
| *Javesella pellucida* (Fabricius) | USA (PA) |  | HM017472 |  |  | HM017325 |
| *Javesella* sp. | China (HLJ) | 27. v. 2007 | KX246215 |  | KX245945 | KX246051 |
| *Kusnezoviella dimidiatifrons* (Kusnezov) | China (NX) | 01. vii. 2008 |  |  | KX245935 | KX246038 |
| *Laodelphax striatellus* (Fallén) | China (FJ) | 07. viii. 2013 | KX246228 | KX246143 | KX245958 | KX246065 |
| *Latistria placitus* (Van Duzee) | China (YN) | 05. viii. 2011 | KX246243 | KX246147 |  | KX246082 |
| *Liburniella ornata* (Stål) | USA (PA) |  | HM017477 |  |  | HM017337 |
| *Megadelphax cornigera* (Kusnezov) | China (NX) | 01. vii. 2008 | KX246232 |  | KX245962 |  |
| *Megadelphax kangauzi* Anufriev | China (SX) | 09. vii. 2006 | KX246231 |  | KX245961 | KX246070 |
| *Mestus cruciatus* Ren & Qin | China (YN) | 12. viii. 2010 | KX380847 |  | KX380843 | KX380845 |
| *Metadelphax propinqua* (Fieber) | USA (UT) |  | HM017486 |  |  | HM017350 |
| *Metadelphax propinqua* (Fieber) | China (JX) | 23. vii. 2013 | KX246218 | KX246137 | KX245948 | KX246055 |
| *Miranus serrulatus* Dong & Qin | China (YN) | 01. viii. 2011 | KX246207 | KX246133 | KX245938 | KX246043 |
| *Miranus varians* (Kuoh) | China (HN) | 27. vii. 2009 | KX246241 |  | KX245971 | KX246080 |
| *Monospinodelphax dantur* (Kuoh) | China (YN) | 21. v. 2011 | KX246211 | KX246136 | KX245942 | KX246047 |
| *Muirodelphax arvensis* (Fitch) (as *Delphacodes campestris* (Van Duzee)) | USA (PA) |  | HM017478 |  |  | HM017339 |
| *Muirodelphax atratus* Vilbaste | China (NX) | 12. vii. 2008 | KX246233 |  | KX245963 |  |
| *Neomegamelanus elongatus* (Ball) | USA (DE) |  | HM017482 |  |  | HM017345 |
| *Neometopina orientalis* Qin | China (HN) | 30. iv. 2008 | KX246212 |  |  | KX246048 |
| *Nilaparvata bakeri* (Muir) | China (JX) | 19. vii. 2013 | KX246240 |  | KX245969 | KX246078 |
| *Nilaparvata* *lugens* (Stål) | China (FJ) | 07. vii. 2013 | KX246217 | KX246140 | KX245947 | KX246054 |
| *Nilaparvata muiri* China | China (JX) | 19. vii. 2013 | KX246248 |  | KX245977 | KX246087 |
| *Nothodelphax neocclusa* (Muir & Giffard) | USA (OR) |  | HM017497 |  |  | HM017391 |
| *Numata muiri* (Kirkaldy) | China (HN) | 12. v. 2008 | KX246242 |  | KX245972 | KX246081 |
| *Opiconsiva albicollis* (Motschulsky) | China (YN) | 21. v. 2011 | KX246200 | KX246129 |  | KX246035 |
| *Opiconsiva albimarginata* Chen & Li | China (FJ) | 17. viii. 2008 | KX246197 |  | KX245928 | KX246030 |
| *Opiconsiva nigra* Ding & Tian | China (YN) | 30. vii. 2010 | KX246227 |  | KX245957 | KX246064 |
| *Opiconsiva* sp. | Australia |  | HM017504 |  |  | DQ532595 |
| *Palego simulator* Fennah | China (YN) | 21. v. 2011 | KX246208 | KX246134 | KX245939 | KX246044 |
| *Paradelphacodes paludosa* (Flor) | China (SX) | 13. viii. 2010 | KX246254 |  | KX245982 | KX246093 |
| *Paradelphax nigrostriata* (Kusnezov) | China (NX) | 06. vii. 2008 | KX246239 |  |  | KX246075 |
| *Paranectopia lasaensis* Ding & Tian | China (XZ) | 28. viii. 2010 | KX246178 |  |  | KX246006 |
| *Peregrinus maidis* (Ashmead) | China (YN) | 21. v. 2011 |  |  | KX245981 | KX246092 |
| *Pissonotus albovenosus* Osborn | USA (DE) |  | HM017480 |  |  | HM017342 |
| *Pissonotus brunneus* Van Duzee | USA (DE) |  | HM017491 |  |  | HM017366 |
| *Prokelisia dolus* Wilson | USA (DE) |  | HM017483 |  |  | HM017346 |
| *Prokelisia marginata* (Van Duzee) | USA (DE) |  | HM017474 |  |  | HM017330 |
| *Rhombotoya pseudonigripennis* Fennah | China (YN) | 21. v. 2011 | KX246219 | KX246138 | KX245949 | KX246056 |
| *Ribautodelphax bidentatus* Anufriev | China (HLJ) | 28. vii. 2007 | KX246246 |  | KX245975 | KX246084 |
| *Ribautodelphax tuvinus* Anufriev | China (HB) | 12. vii. 2006 | KX246204 |  |  | KX246039 |
| *Sardia rostrota* Melichar | China (YN) | 29. vii. 2011 | KX246229 | KX246144 | KX245955 | KX246066 |
| *Shadelphax eforiae* (Dlabola) | China (GS) | 30. vii. 2009 | KX246253 | KX246153 |  |  |
| *Sinolacme tortilla* (Kuoh) | China (HN) | 01. v. 2008 |  |  | KX245970 | KX246079 |
| *Sogata hakonensis* (Matsumura) | China (ZJ) | 26.vii. 2011 | KX246198 | KX246127 | KX245929 | KX246031 |
| *Sogata nigrifrons* (Muir) | China (FJ) | 28. vii. 2013 | KX246222 | KX246141 | KX245952 | KX246059 |
| *Sogatella furcifera* (Horváth, 1899) | China (FJ) | 07. viii. 2013 | KX246196 | KX246126 | KX245927 | KX246029 |
| *Spartidelphax detectus* (Van Duzee) (as *Delphacodes detecta*) | USA (DE) |  | HM017475 |  |  | HM017331 |
| *Spartidelphax penedetectus* Beamer (as *Delphacodes penedetecta*) | USA (FL) |  | HM017487 |  |  | HM017359 |
| *Stiropis nigrifrons* (Kusnezov, 1929) | China (NX) | 22. viii. 2011 | KX246223 |  | KX245953 | KX246060 |
| *Struebingianella detecta* (Linnavnori) | China (HLJ) | 27. v. 2007 | KX246235 |  | KX245965 | KX246072 |
| *Sulculus sulcatus* Ding | China (YN) | 21. v. 2011 | KX246214 |  | KX245944 | KX246052 |
| *Tagosodes orizicolus* (Muir) | Venezuela |  | HM017494 |  |  | HM017381 |
| *Tarophagus colocasiae* (Matsumura) | China (HN) | 01. v. 2008 | KX246251 |  | KX245980 | KX246090 |
| *Terthron albovittatum* (Matsumura) | China (GZ) | 20. viii. 2012 | KX246202 | KX246131 | KX245934 | KX246037 |
| *Toya terryi* (Muir) | China (YN) | 21. v. 2011 | KX246225 | KX246142 | KX245954 | KX246062 |
| *Toyoides albipennis* Matsumura | China (YN) | 21. v. 2011 | KX246236 | KX246146 | KX245966 | KX246073 |
| *Ulanar muiri* (Metcalf) | China (YN) | 01. viii. 2011 | KX246230 |  | KX245959 | KX246067 |
| *Unkanodes sapporona* (Matsumura) | China (LN) | 08. viii. 2009 | KX246199 |  | KX245932 | KX246034 |
| Outgroups |  |  |  |  |  |  |
| Cixiidae |  |  |  |  |  |  |
| *Cixius scrupeus* Fennah | China (GZ) | 02. viii. 2013 | KX246154 | KX246094 | KX245891 | KX245983 |
| *Oecleopsis* sp. | China (SX) | 01. viii. 2014 | KX246157 | KX246097 |  | KX245986 |
| *Oliarus* sp. | China (GZ) | 01. viii. 2012 | KX246158 | KX246098 |  | KX245987 |
| *Pentastiridius* sp. | China (SX) | 08. viii. 2013 | KX246155 | KX246095 |  | KX245984 |
| *Reptalus squadricinctus* (Matsumura) | China (GZ) | 28. vii. 2013 | KX246156 | KX246096 |  | KX245985 |
| Delphacidae |  |  |  |  |  |  |
| Kelisiinae |  |  |  |  |  |  |
| *Kelisia xiphura* Vilbaste | China (NX) | 22. viii. 2011 | KX246161 |  | KX245894 | KX245990 |
| Stenocraninae |  |  |  |  |  |  |
| *Stenocranus montanus* Huang & Ding | China (GZ) | 23. viii. 2012 | KX246160 | KX246100 | KX245893 | KX245989 |
| *Terauchiana singularis* Matsumura | China (SX) | 06. ix. 2013 | KX246159 | KX246099 | KX245892 | KX245988 |
